# Numerical simulation of quantum dots as a buffer layer in CIGS solar cells: a comparative study

**DOI:** 10.1038/s41598-022-12234-0

**Published:** 2022-05-16

**Authors:** Zuhair R. Abdulghani, Asmaa Soheil Najm, Araa Mebdir Holi, Asla Abdullah Al-Zahrani, Khaled S. Al-Zahrani, Hazim Moria

**Affiliations:** 1 Department of Mechanical Engineering Technology, Yanbu Industrial College, Yanbu Al-Sinaiyah City, 41912 Kingdom of Saudi Arabia; 2 Department of Electrical Electronic and Systems Engineering, Faculty of Engineering and Built Environment, Universiti Kebangsaan Malaysia (UKM), 43600 Bangi, Selangor Malaysia; 3 Department of Physics, College of Education, University of Al-Qadisiyah, Al-Diwaniyah, Al-Qadisiyah 58002 Iraq; 4 Imam Abdulrahman Bin Faisal University, Eastern Region, Dammam, Kingdom of Saudi Arabia

**Keywords:** Electronic devices, Energy

## Abstract

Quantum bandgap buffer layers can improve sunlight absorption in the short wavelength region, hence improving the performance of CIGS solar cells. In this study, we use numerical modelling to determine the impact of various buffer layers' electrical characteristics on the performance of CIGS thin film photovoltaic devices, particularly, carrier concentration and the quantum effect. As well Ag_2_S buffer layer has been experimentally examined to fulfilment its effect in term of bulk and quantum bandgap. Experimental results depicted that, Ag_2_S QDs has polycrystalline nature of films, with smooth surface roughness, and average diameter 4 nm. Meanwhile, a simulation revealed that the Fermi level of the (*n*-buffer layer) material shifts closer to the conduction band with an increase in carrier concentration. The findings indicate that, a buffer layer with a wider bandgap and carrier concentration is an essential demand for achieving a device with a higher conversion efficiency and a broader bandgap-CBO window. It was attributed to beneficial synergistic effects of high carrier concentration and narrower depletion region, which enable carriers to overcome high CBO barrier. Most importantly, modelling results indicate that the optic-electrical characteristics of the buffer layer are critical in determining the progress of a CIGS solar cell.

## Introduction

The substantial climate changes are a good reason to look for alternative resources of energy. The huge challenge for the global community is to research alternative energy sources that are renewable, abundant, environmentally friendly and cost-effective and at the same time, the current energy system prevents any negative consequences^1^. Solar electricity is expected to play an essential role in all alternative energy resources in terms of sustainable and fossil-fuel-free energy production^2^. Photovoltaic (PV) devices are semiconductor devices with the ability to convert energy in sunlight into electricity. Presently, global PV installations are comprised of monocrystalline silicon (c-Si), multi-crystalline silicon (mc-Si), thin film technologies and emerging PV^3,4^.Furthermore, the increasing need for low-cost photovoltaic (PV) modules has revealed some inherent drawbacks of c-Si technology, like as a lack of raw materials, high costs of material processing and device fabrication steps, and an inability to form monolithic interconnections^5^. Among thin-film photovoltaics, the copper indium gallium di-selenide (CIGS) have garnered tremendous interest in the PV research community, which eventually translated into systematic theoretical and experimental studies and consequently attained a power conversion efficiency (PCE) of 23.35%^6^.

Although of that, CIGS performance is still far behind that of other photovoltaic platforms. One of the fundamental reasons for this is choosing the appropriate buffer layer^7^. Cadmium sulphide (CdS) is a significant compound semiconductor (II-VI), n-type conductivity characterized by its high transparency, direct bandgap transition (2.4 eV), and efficient electron affinity (4.2 eV),^8,9^. CdS as well improves the lattice heterojunction interface match, enhances the additional carrier lifetime, and optimizing the device's band alignment^10^. CdS thin film deposition can be done by various methods, for instance, chemical vapour transport (CSVT), chemical bath deposition (CBD), magnetron sputtering, and thermal evaporation^11–13^. Most of the techniques are very complex and hard to control, and thus costly. CBD consider as the most common fabrication techniques to deposit the buffer layer in solar cells because of its simple deposition process, low costs, high yield, and eco-friendly. Besides, this process can be controlled easily through pH, salt concentration, and temperature variations, thereby obtaining a high quality of thin film with desired thickness and crystallinity^14^. Different materials with a wider bandgap, also the non-toxic materials for example ZnS, Ag_2_S, PbS, as well as In_2_S_3_ also investigated as a potential buffer layer^15–18^. However, these buffer layers suffer from complicated reaction mechanism and light soaking effects, presenting a potential cell durability and reproducibility issues^19^. The identification of the optical, electrical, and structural characteristics of CdS films is critical for an assortment of scientific, technological, and commercial uses, most notably in the field of semiconductor applications such as solar cells.

Further, the buffer layer's role in a hetero-junction is to create a junction with the absorber layer, enabling the absorber layer to absorb as much light as possible. Ideally, the buffer layer must have the lowest possible absorption losses, surface recombination, and electrical resistance in order to allow the most effective transmission of photogenerated carriers to the outside circuit. It is also responsible for strengthening the cell's band alignment and electrical properties, as well as forming a wide depletion zone with the p-type absorber layer^20^. Thus, to achieve a desirable characteristic, the buffer layer must have higher carrier concentrations with a wider bandgap. In our previous work, we have proven that buffer layer with higher carrier mobility and carrier concentration is a vital necessity as it is led to enhance *J*_sc_ due to a higher carrier diffusivity, which enables carriers to overcome a high CBO barrier which is followed with higher conversion efficiency^21^.

Besides, compare with the bandgap value of bulk buffer, the increasing in this value reveals quantum confinement induced by the limited particle size (less than 10 nm)^22^. CdSe, CdS, CdTe, PbS, Ag_2_S, and PbSe, etc. are the most often utilized quantum dots^23–26^.

A significant feature of QDs is that their bandgap may be adjusted by altering the size of the quantum dot^27^. This is a considerable advantage over other nanocrystals since it allows for more control over the bandgap. This characteristic of QD has a significant influence on the design of solar cells because the optoelectronic performance of the solar cell can be controlled by changing the bandgap of the semiconductor. This quantum confinement effect is connected with the alteration of the electronic properties by displacing the conduction and valence bands' energy level position to more negative and positive values, respectively. This redox potential shift favors the mechanism of electron transfers and increases photoactivity^28^.

Hence, in this study, we have focused in our numerical simulation to elucidate the interdependence of carrier concentration and bandgap of various types of buffer layer on PCE of the CIGS solar cell. Intentionally, to accurate the simulated results, Ag_2_S as buffer layer has been experimentally selected to investigated its structure and morphology in terms of bulk and quantum bandgap. In order to maximize the applicability of simulation outcomes, all the possible permutations and combinations of buffer bandgap for bulk and quantum effect has been taken into consideration in terms of solar cell parameters. The fundamental target of this study is to highlight the importance of having high-quality buffer layer, from an electrical and optical property point of view, focusing on the effect of using a high carrier concentration and higher bandgap during the initial development period of photovoltaic solar cells.

## Methodology

### Preparation of Ag_2_S

**Figure 1:**
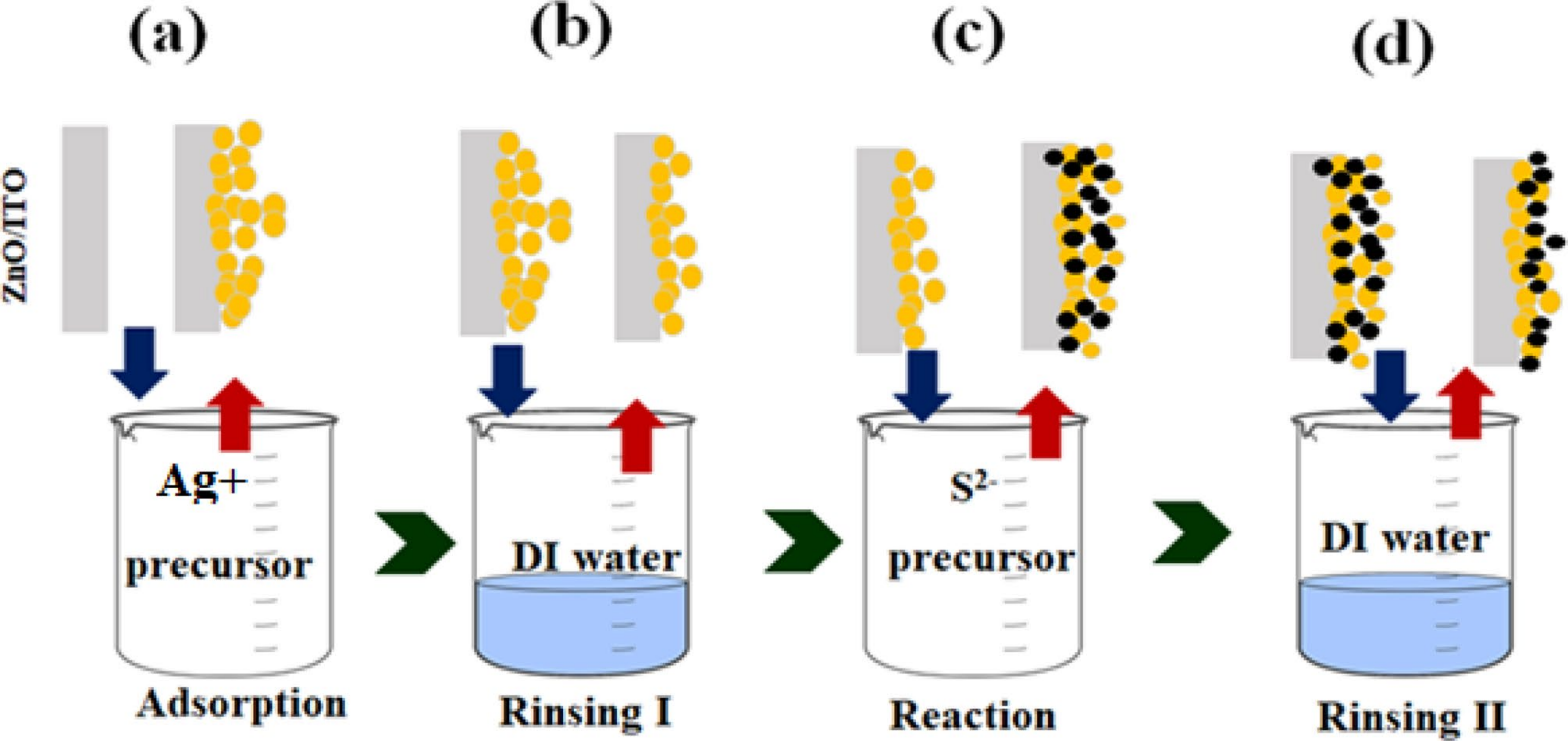
Preparation of Ag_2_S nanoparticles/ZnO NRAs nanoparticles using SILAR technique (yellow circles = Ag^+^ and black circles = S^2−^).

Ag_2_S/ZnO NRAs films were prepared by two different methods, once for obtaining the nanoparticles, and the second to achieve the quantum dot size. The Ag_2_S nanoparticle/ZnO NRAs photoanode was prepared using the SILAR technique. This approach was adapted from our previous research^29^. At ambient temperature, a thin film of Ag_2_S was deposited upon ZnO NRAs/ITO using 0.02 M AgNO_3_ and 0.02 M Na_2_S as cationic and anionic precursor, respectively. For the deposition procedure, a ZnO NRAs substrate was submerged in a cationic precursor solution including Ag^+^ ions. Following that, the substrate was washed with DI to eliminate any unabsorbed Ag^+^ ions. Additionally, the substrate was submerged in an anionic precursor, that enables S^2−^ ions to flow from the solution in the diffusion layer to the solid solution interface, where they react with Ag^+^ ions to form Ag_2_S. After that, a second rinse with DI was used to remove any remaining material from the substrate, as seen in Fig. 1. The durations of immersion and rinsing were determined experimentally to be 100 s. Likewise, Ag_2_S QDs/ZnO NRAs films were made by submerging ZnO NRAs/ITO in an equivalent volume of an aqueous solution of 30 mM CS (NH_2_)_2_ and 5 mM AgNO_3_ in a sealed glass container. The container was then put in an oil bath and heated for 25 min at 60 °C on a programmable hot plate. The substrate was repeatedly cleaned with DI to eliminate any remaining impurities. Following that, the samples were heated to 400 °C for one hour at a rate of 2 °C per minute in a N_2_. Both procedures' samples were allowed to cool first, prior to characterize. The experimental data for Ag_2_S QDs were obtained with permission from our previously published article^30^.

### Simulation

**Figure 2:**
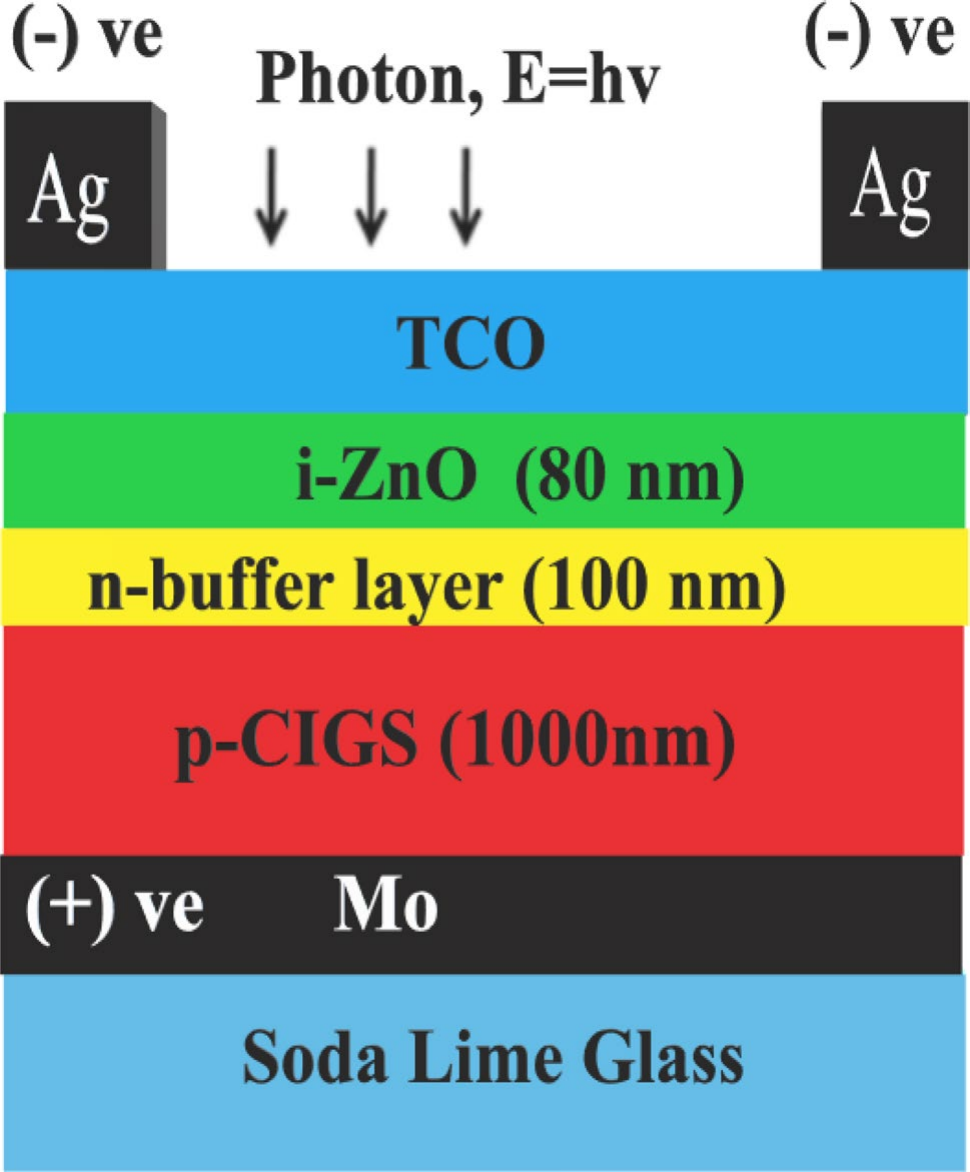
Device configuration for CIGS solar cell.

Numerical modeling will aid in comprehending the behavior of solar cells and will provide more insight into how to adjust fabrication parameters to increase cell performance. In this numerical simulation study, the SCAPS-1D (version 3.3.07) software has been utilized to simulate the effects of a variable carrier concentration of various buffer layers on the overall performance of the substrate-type thin-film photovoltaic device. SCAPS-1D is one-dimensional computer software for simulating the DC and AC electrical properties of thin-film heterojunction solar cells. It was designed and maintained by a research team at the University of Gent lead by Marc Burgelman^31^. The modelling capabilities of SCAPS is specifically designed to mimic the characteristics of CIGS solar cells, however, it has been observed and shown to be effective in a range of different cell types. The generic device structure that has been adopted in this study and subsequently modelled in SCAPS is as shown in Fig. 2.

Brief literature from the material property perspective for each layer and pertinent theoretical framework is given in the subsequent paragraphs, with the aim to derive credible justification for the selection of material and hetero-interface electronic parameters, which have been used in this study as shown in Tables 1, 2 and 3.

**Table 1:** List of physical and electronic properties for each layer parameter^15,17,18,32–40^.

Layer parameters	*i*-ZnO	*n*-CdS	*n*-ZnS	*n*-Ag_2_S	*n*-In_2_S_3_	*n*-PbS	*p*-CIGS
Thickness (nm)	80	100	100	100	100	100	1000
Dielectric constant, ε_r_	10	10	10	10	13.5	10	10
Electron mobility, µ_n_ (cm^2^/V s)	50	50	50	50	50	50	50
Hole mobility, µ_p_ (cm^2^/V s)	20	20	20	20	20	20	20
Acceptor concentration, *N*_A_ (cm^−3^)	0	0	0	0	0	0	5.5 × 10^15^
Donor concentration, *N*_D_ (cm^−3^)	5 × 10^17^	10^15^–10^19^	10^15^–10^19^	10^15^–10^19^	10^15^–10^19^	10^15^–10^19^	0
Bandgap, *E*_g_ (eV)	3.4	2.4, 2.65	3.54, 3.95	1.1, 1.82	2.32, 2.98	1.22, 1.61	1.2
Electron affinity, χ (eV)	4.55	4.45	4.5	4.5	4.7	4.35	4.5
Effective density of states in Conduction band, *N*_C_ (cm^−3^)	4 × 10^18^	2 × 10^18^	2 × 10^18^	2 × 10^18^	2 × 10^18^	2 × 10^18^	2 × 10^18^
Effective density of states in Valence band, *N*_V_ (cm^−3^)	9 × 10^18^	1.5 × 10^19^	1.5 × 10^19^	1.5 × 10^19^	1.5 × 10^19^	1.5 × 10^19^	2 × 10^18^
Electron thermal velocity (cm s^−1^)	1 × 10^7^	1 × 10^7^	1 × 10^7^	1 × 10^7^	1 × 10^7^	1 × 10^7^	1 × 10^7^
Hole thermal velocity (cm s^−1^)	1 × 10^7^	1 × 10^7^	1 × 10^7^	1 × 10^7^	1 × 10^7^	1 × 10^7^	1 × 10^7^

**Table 2:** List of physical and electronic properties for interface parameters.

Interface parameters	Front	–	Back
Metal work function (eV)	4.47 (Ag)	–	4.95 (Mo)
Majority carrier barrier height Φ_b_ (eV)	Φ_bn_ = 0	–	Φ_bp_ = *E*_g-Absorber_ − Φ_bn_
Electron surface recombination velocity, *S*_e_ (cm s^−1^)	10^7^	–	10^7^
Hole surface recombination velocity, *S*_h_ (cm s^−1^)	10^7^	–	10^7^
Reflectivity	0.05	–	0.80

**Table 3:** Defect characteristics of all layers employed in this work.

Layer parameters	*i*-ZnO	*n*-buffer	CIGS
Defect type	Neutral	Neutral	Neutral
Electron capture cross section (cm^2^)	10^−12^	10^−13^	10^−15^
Hole capture cross section (cm^2^)	10^−12^	10^−13^	10^−13^
Energetic distribution	Gauẞ	Gauẞ	Gauẞ
Reference for defect energy level *E*_t_	Above *E*_v_	Above *E*_v_	Above *E*_v_
Energy level with respect to a reference (eV)	1.650	1.200	(0.6/1.1) × *E*_g-Absorber_
Characteristic energy (eV)	0.100	0.100	0.100
Total defect density, *N*_T-total_ (cm^−3^)	1.772 × 10^16^	1.772 × 10^17^	0–1.772 × 10^17^
Peak defect density *N*_T-peak_ (eV^−1^·cm^−3^)	1 × 10^17^	1 × 10^18^	0–1 × 10^18^

The effects of the SLG substrate on the heterojunction band energy layout were not taken into consideration in the simulation because of the limitations posed by SCAPS software used in this study. Conventionally, in a substrate-type device architecture, the molybdenum (Mo) thin film is the primary select back contact due to its chemical inertness, good thermal stability and suitable electrical and optical (reflectivity) properties^41,42^. The work function of Mo back contact is set to 4.95 eV^43^. The absorber layer thickness was fixed at 1000 nm throughout the entire simulation. Table 3 summarizes the defect properties for all the relevant layers adopted. In all instances, the buffer layer thickness was kept constant at 100 nm. A 80 nm thick *i*-ZnO layer was incorporated as a (TCO) layer on the top of the buffer layer and was followed by a front contact metal electrode (Ag front electrode). All numerical simulations were achieved at a constant temperature of 300 K. No additional series resistance and shunt resistance parameters were defined for simplicity. Built-in standard solar spectrum (AM1.5G-1 Sun) with an integrated power density of 1000 W/m^2^ was selected as an illumination bias. Due to the scope of this study, which focuses primarily on the optical and electrical characterizations of the buffer layer and its suitability for CIGS, the interfacial recombination mechanism was not incorporated. Also, the following equations are obtained from the literature to using in this simulated work^44–49^.

1 $$E_{F} = \frac{{E_{c} + E_{v} }}{2} + \frac{k T}{2}\ln \left( {\frac{{N_{v} }}{{N_{c} }}} \right)$$

2 $$Ev = kTln\frac{{N_{V} }}{{N_{A} }}$$

3 $$Ec = kTln\frac{{N_{C} }}{{N_{D} }} = { }Ev + Eg$$

where is; *K*, *T*, *N*_V_/*N*c, and *N*_A_/*N*_D_ is Boltzmann constant, the absolute temperature in Kelvin, the effective density of states at the valence/conductive band edges, acceptor/donor density, respectively.

4 $$N_{c} = 2 \left( {\frac{{2\pi m^{*} kT}}{{h^{2} }}} \right)^{\frac{3}{2}}$$

5 $$\mu = \frac{e \tau }{{m^{*} }}$$

where m*, $h$, e, τ is the effective mass, and Planck’s constant, the electron charge, and relaxation time.

6 $$J_{sc} = J_{0} \left( {e^{{\frac{q Voc}{{AKT}}}} - 1} \right)$$

7 $$J_{0} = A N_{C} N_{V} e^{{ - \frac{Eg}{{KT}} }} \left( {\frac{{D_{n} }}{{L_{n} N_{A} }} + \frac{{D_{p} }}{{L_{p} N_{D} }}} \right)$$

where *J*_0_, *A*, *q*, *D*_n/p_, and *L*_n/p_, is, saturation current density, the Illuminated device area, charge of an electron, the diffusion coefficient of electron and hole, and the diffusion length of electron and hole.

8 $$\upeta = \frac{{FF V_{oc } J_{sc } }}{{P_{in} }}$$

whereby $\upeta$, *FF*, *V*_oc_, *J*_*sc*_, and *P*_*in*_ is the conversion efficiency, fill factor, open circuit voltage, short circuit current and input power.

9 $$D_{n} = \frac{{\mu_{n} k T}}{q}$$

10 $$L_{n} = \sqrt {D_{n} \tau_{n} }$$

11 $$I_{ph} = qAG\left( {L_{n} + W + L_{p} } \right)$$

12 $$W_{d} = \left[ {\frac{{2_{{\epsilon_{1} \epsilon_{2} }} \left( {V_{bi} - V} \right)\left( {N_{A}^{2} + N_{D}^{2} } \right)}}{{q\left( {\epsilon_{1} N_{D} + \epsilon_{2} N_{A} } \right)N_{D} N_{A} }} } \right]^{1/2}$$

whereby *µ*_n_, *A*, *G*, *W*, $\epsilon_{1} \epsilon_{2}$, *V*_bi_, *V*, is electron mobility, the cross-sectional area, the carrier generation rate, the depletion width of the heterojunction, dielectric permittivity of buffer layer/absorber layer, built-in voltage, and applied voltage.

## Results and discussion

### Ag_2_S experimental results

**Figure 3:**
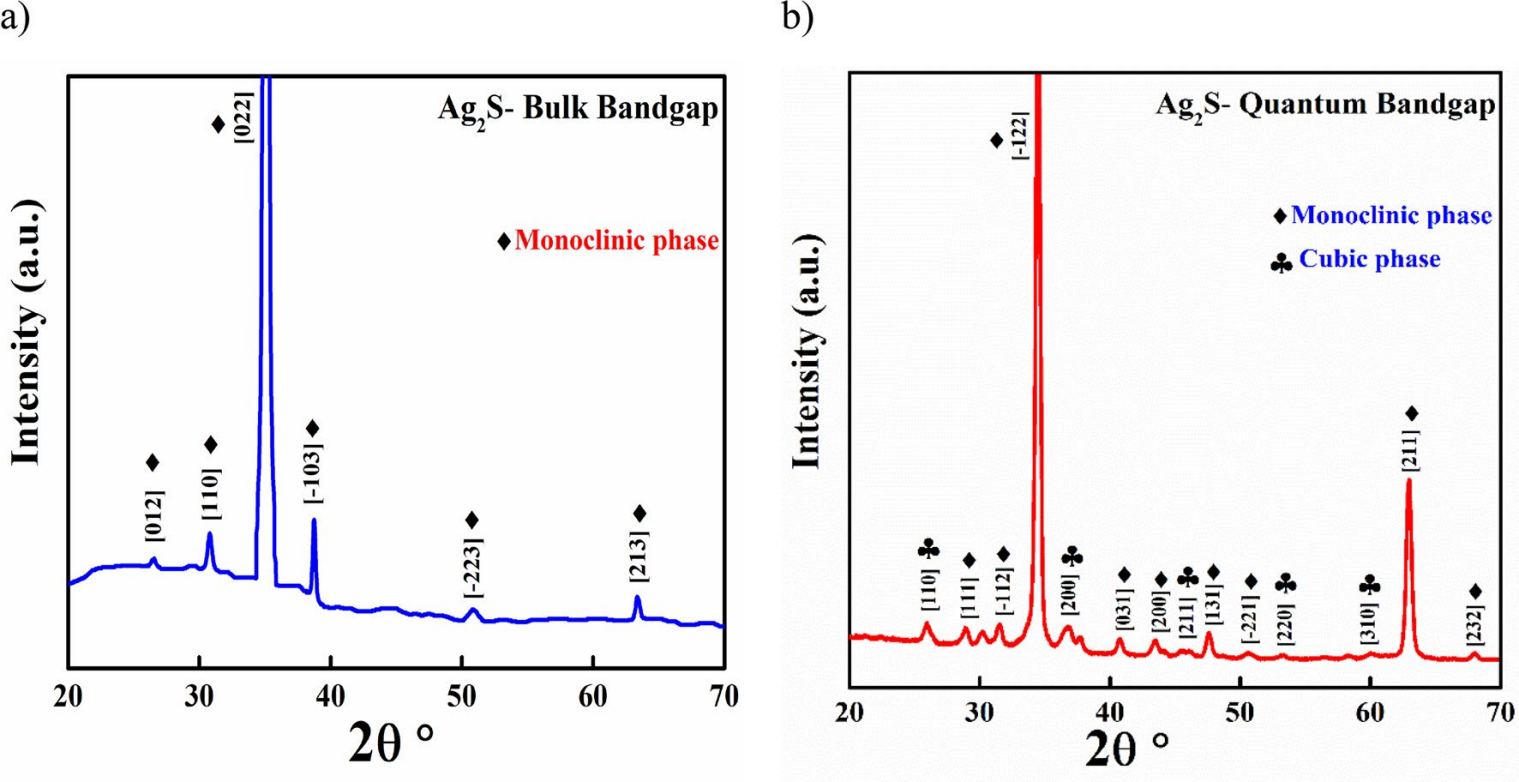
XRD patterns of: (**a**) Ag_2_S nanoparticles, (**b**) Ag_2_S QDs.

To obtain more detailed structural information regarding the sample, XRD analysis were presented. Figure 3 illustrates X-ray patterns of Ag_2_S at both nanoparticle and quantum dot. The films were scanned from 20° to 70°. Ag_2_S mainly forms cubic and monoclinic phases based on deposition conditions. Besides, the specific identification of the crystal structure of Ag_2_S, whether the film is primarily monoclinic or essentially cubic or a mixture of both, is difficult to determine since both of the film phases have the same XRD diffraction peak angles. It was found that Ag_2_S nanoparticle has a monoclinic phase only (JCPDS-01-075-1061), based on the peaks corresponding to (012), (110), (022), (− 103), (− 223) and (213) crystal planes in the diffraction pattern. Indeed, Ag_2_S QDs display presence of many strong diffraction peaks orientation along (110), (200), (211), (220), and (310) plane indicate the polycrystalline nature of films, which has been belong to the cubic phase, (JCPDS-00-001-1151). This can be explaining since Ag_2_S is stable in a monoclinic structure at room temperature, but suffers a thermo-induced phase shift into a cubic structure at 450 K^50^. Silver ions are randomly dispersed across the interstitial sites of a sulphur lattice in this high-temperature structure, resulting in a favourable ionic conductivity owing to the quantum effect of particle size.

A particle, or grain, is composed of one or more united crystals that are fused together. Although the size of such a particle cannot be determined using XRD, it can be measured using light microscopy, light scattering methods, or high resolution scanning electron microscopy (HR-TEM)^51^.

**Figure 4:**
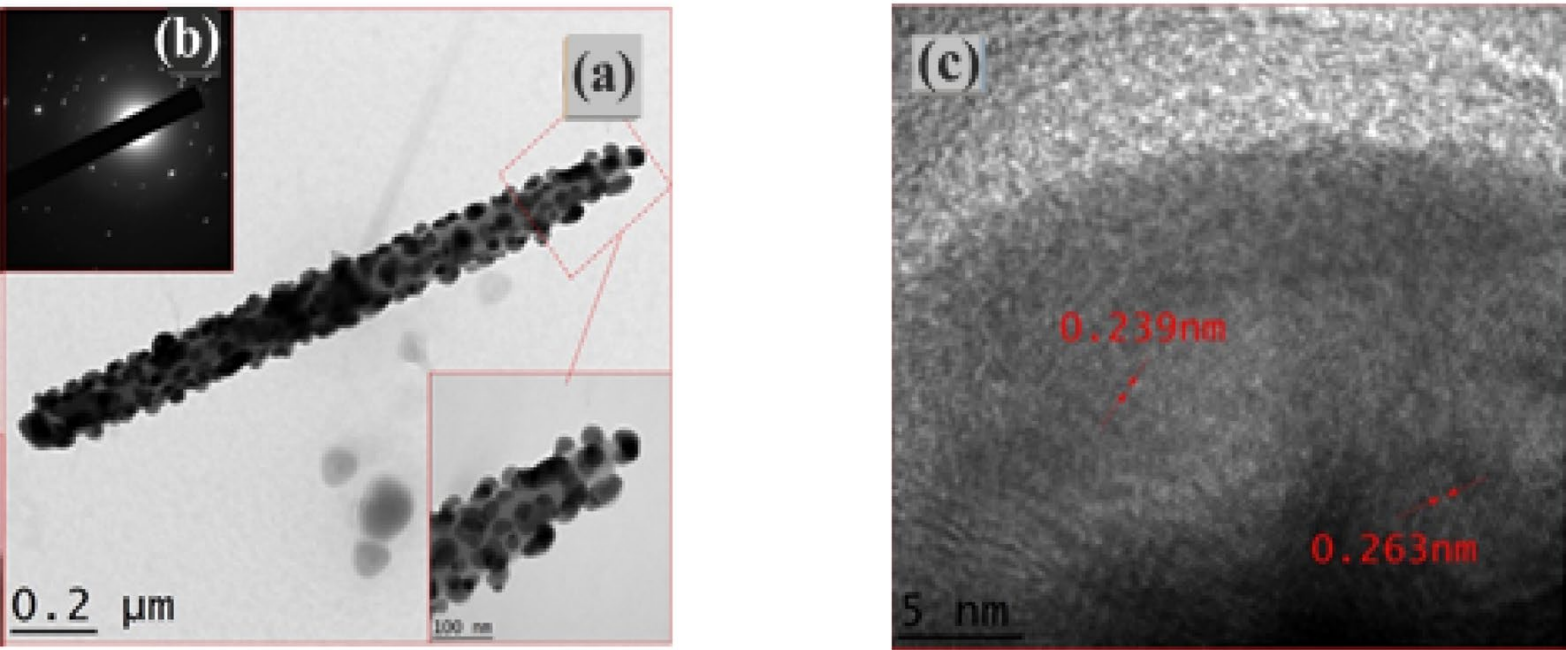
Ag_2_S nanoparticle/ZnO NRAs (**a**) TEM image, (**b**) SAED and (**c**) HRTEM image.

To examine the effect on the surface morphology further, an illustrative sample of Ag_2_S nanoparticles/ZnO NRAs/ITO was analyzed under TEM as shown in Fig. 4a. As realized, spherical Ag_2_S nanoparticles with an average diameter of 20.31 ± 0.2 nm were distributed onto the ZnO NR surface, resulting in a relatively rough surface. HR-TEM was used to verify the crystal structure and interplanar distances of single Ag_2_S nanoparticles. The area of electron diffraction in a particular portion of the HR-TEM image was calculated using conventional FFT. Figure 4b depicts the (SAED) pattern of Ag_2_S/ZnO/ITO, which shows some sets of diffraction spots verifying the binary hetero-structure's polycrystalline nature. Additionally, to corroborate the interplanar spacing of the binary hetero-structure Ag_2_S/ZnO NRAs/ITO, Fig. 4c displays an HR-TEM image of Ag_2_S nanoparticles. The plane fringes with a crystalline plane spacing of 0.239 nm were roughly assigned to the (002) and $\text{(}\stackrel{\mathrm{-}}{1}{\text{03}}$) planes of the hexagonal wurtzite structure of ZnO NRAs and monoclinic Ag_2_S phase, respectively (as confirmed by reference data JCPDS 00-003-0888, JCPDS 00-014-0072, and other related reports^30,52^.

**Figure 5:**
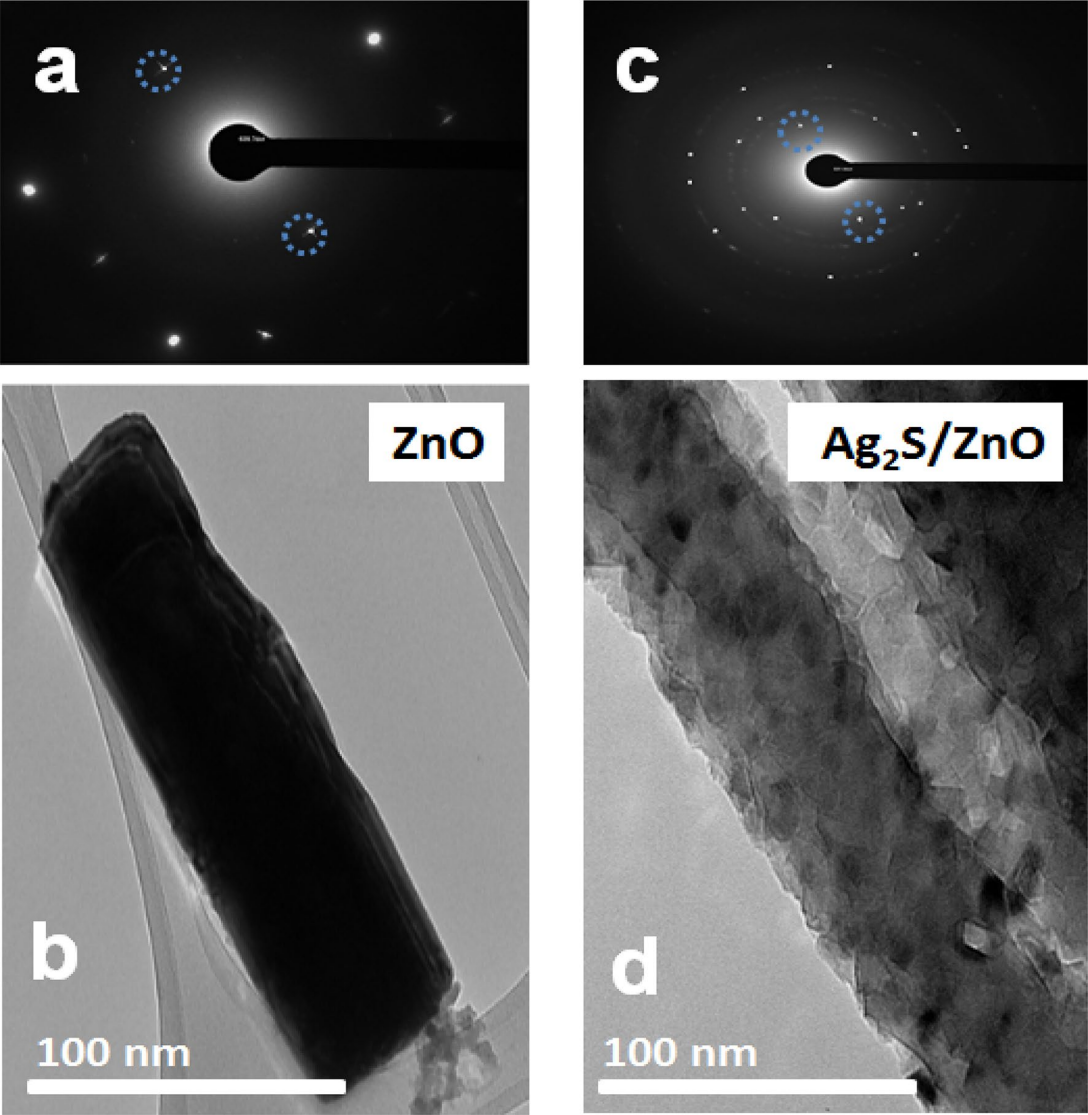
The TEM images and the SAED pattern of pure ZnO; (**a**,**b**) and Ag_2_S/ZnO (**c**,**d**).

**Figure 6:**
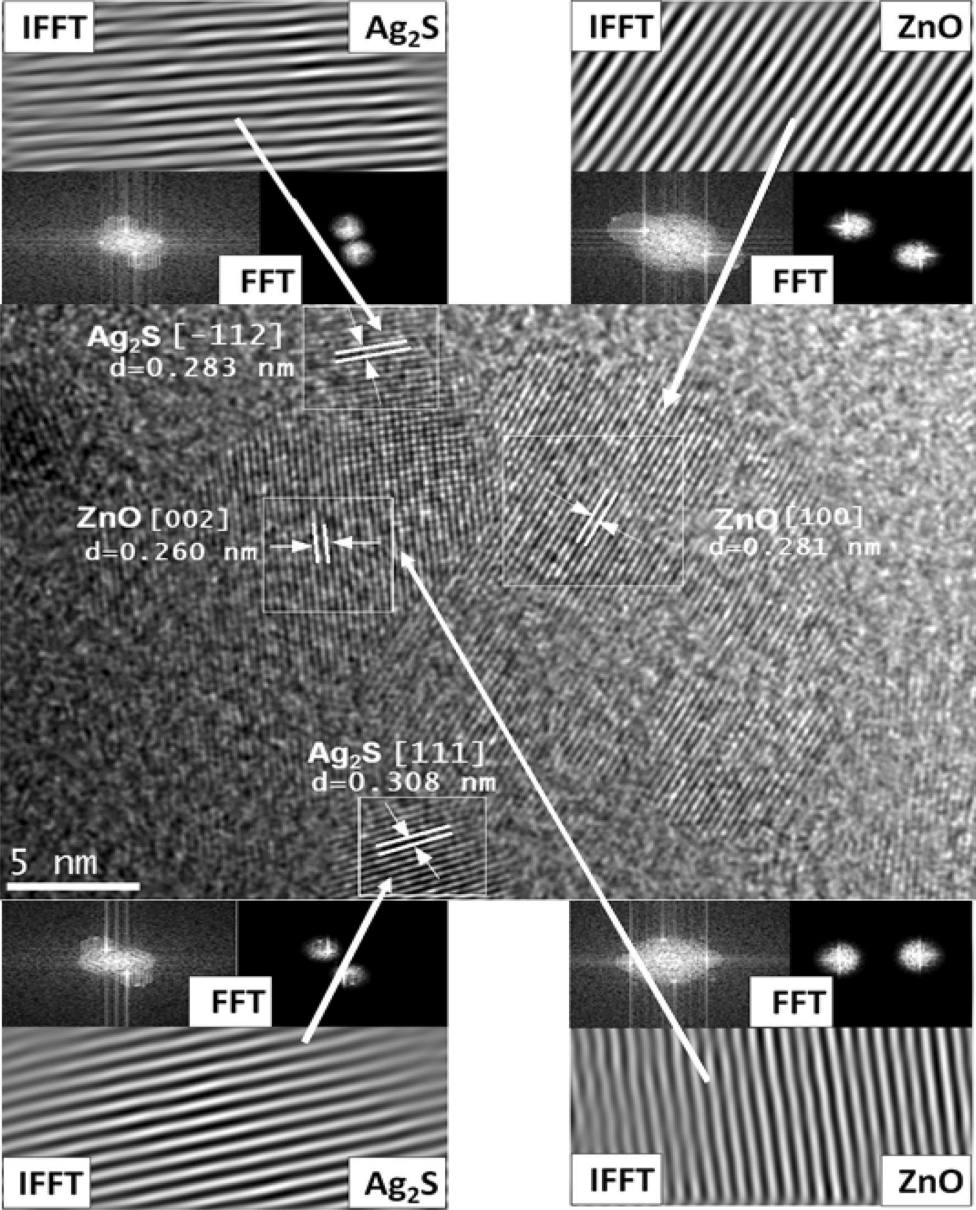
HRTEM image of the Ag_2_S/ZnO and diffraction patterns via FFT and IFFT.

The SAED patterns of ZnO and Ag_2_S/ZnO are presented in Fig. 5a,c, respectively. Pure ZnO, Fig. 5a, exhibited the (2 0 0) plane of hexagonal wurtzite ZnO with d-spacing of approximately 0.260 nm, whereas Fig. 5c exhibited some sets of diffraction spots identified as polycrystalline Ag_2_S/ZnO with d-spacing of approximately 0.343 nm that might be assigned to the monoclinic Ag_2_S's (1 1 1) plane. Figure 5b,d show TEM images of pure ZnO NR and Ag_2_S/ZnO. The rods' surfaces were not particularly smooth in contrast. Figure 5d depicts the homogeneous distribution of Ag_2_S nanoparticles (with a mean diameter of about 4 nm) over the surface of ZnO NR. The diffraction patterns obtained from this image using FFT and IFFT in Fig. 6 revealed plane fringes with crystalline plane spacing of 0.308 and 0.283 nm, respectively, which were attributed to the (1 1 1) and (1 1 2) planes of the monoclinic phase of Ag_2_S.

### Study of the influence of energy band alignment

In the application of solar cells, a cell model is a theoretical structure designed to simulate real processes and characteristics that may have an impact on cell performance. They may vary from device to device due to the fact that many of them are reliant on fabrication procedures and deposition methods. The numerical simulation work presented herein is primarily focused on assessing the impacts of optical and electrical characterizations of buffer layers, namely the bandgap and carrier concentration on the performance of CIGS photovoltaic devices. There is a misunderstanding about the relation between the electron affinity (χ), bandgap (*E*_g_), and both the conduction band edge (*E*_c_) with the valence band edge (*E*_v_), as it is depending on the donor density (*N*_D_) and the voltage and illumination conditions^53^. Thus, the impact of using different donor densities with different buffer layers in two cases: bulk-bandgap and quantum dot-bandgap buffer layers, has been investigated. The density of states (DOS) is considered as a function of the lattice parameters and temperature. Therefore, these parameters principally affect the *E*_g_. The density of states and energy level spacing alters with the reduction in particle size, owing to quantum confinement effects and high surface area to volume ratio. Briefly, the density of states at the valence band effects on the properties of any photovoltaic material, namely the absorption coefficient, the lifetime or recombination rate, and the mobility^54^.

In equilibrium, in a neutral, as the main layer of device configuration is *p*-type (CIGS), the Fermi level (*E*_F_) equal to Fermi Level in the *p*-type material (*E*_Fp_), and the valence band edge (*E*_v_) is fixed amount, regardless the bandgap and/or electron affinity grading, Eq. (1). This is because of the valence band edge (*E*_v_) is depending mainly on the acceptor density (*N*_A_). Thus, with supposing that both N_A_ and N_V_ are uniform and not graded, Eq. (2). The conduction band (*E*_c_) is then placed at a distance with *E*_g_, and above *E*_v_, and will thus be sloped when *E*g is graded, Eq. (3). The next layer in device configuration is the *n*-type buffer layer. Various buffer layers have been applied in a constant circumstance (applied *V*, illumination, in a depletion layer, grading of the doping *N*_A_ or of the densities of states (*N*_C_/*N*_V_). Only, densities *N*_D_ have changed three times (10^15^, 10^17^, and 10^19^ cm^−3^) in each buffer layer in two cases bulk-bandgap and quantum dot-bandgap.

**Figure 7:**
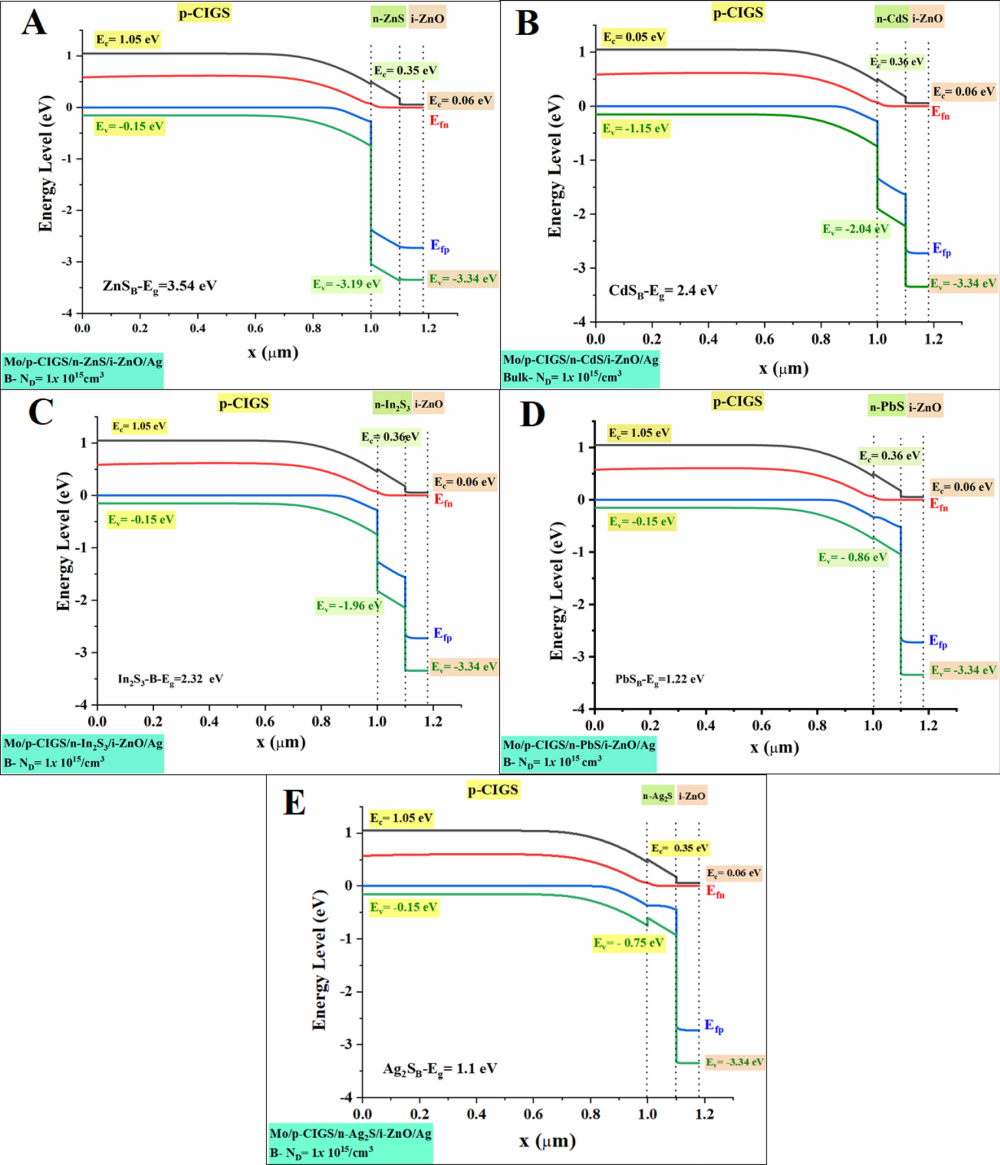
Band diagram at Bulk bandgap at *N*_D_ = 10^15^.

**Figure 8:**
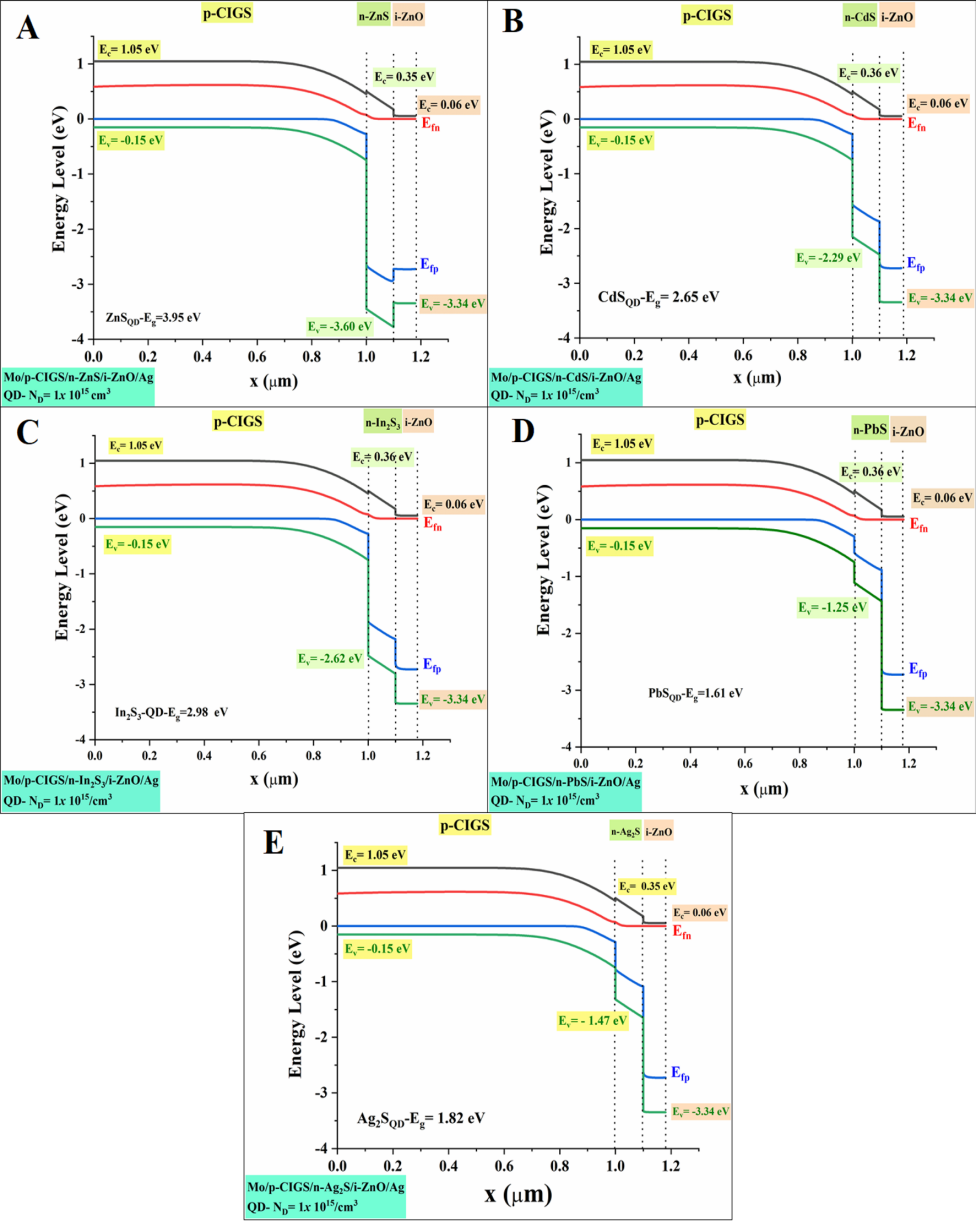
Band diagram at Quantum bandgap at *N*_D_ = 10^15^.

Figure 7a–e depicts the proposed energy band diagram for buffer layers. The suggested structure is modelled using experimentally measured values for electrical and optical characteristics that are provided into the software. Figures 7, 8 and supplementary A1, B1, A2 and B2, show shifting in the Fermi energy level as a function of donor concentration (n-buffer layer) at T = 300 K. This could be illustrated accordingly to Fermi energy of an intrinsic semiconductor formula, Eq. (1). Indeed, when the doping levels increase, causing in the drop of the conduction band. As a result, the Fermi level shifts downward into the valence band, while the Fermi level electrons jump into the conduction band^46^. This can be noticeable from the results, as the maximum shifting can be achieved is for the Ag_2_S buffer layer when the conduction band edge (*E*_c_) is jumped from 0.35 to − 0.05, for *N*_D_ 10^15^ to 10^19^ cm^−3^ respectively. Numerical results as well showed that, with increasing the N_D_, the buffer layer displayed lower Ec in all simulated cases. Equations (3), (4), and (5) can be used to explain the results. According to Eq. (3), when the donor doping concentration (*N*_D_) grows, the (*N*c) declines, which is consistent with a low effective mass Eq. (4). Due to the fact that a reduced effective mass results in increased charge carrier mobility and low exciton separation energy, the *J*_sc_ and efficiency will rise, as shown in Eqs. (6) and (7). For example, parallel to the quantum bandgap of Ag_2_S and PbS buffer layers, low *N*c results in an increase in *J*_sc_ from 9.53 to 31.46 V and 11.72 to 31.64 V, respectively, as shown in Table 4.

**Table 4:** CIGS solar cells' photovoltaic performance characteristics at various buffer layers.

*N*_D_	*V*oc	*J*_sc_	*FF*	*eff*	*V*_oc_	*J*_sc_	*FF*	*eff*
	**Ag**_**2**_**S (bulk bandgap)**				**Ag**_**2**_**S (quantum bandgap)**			
1.00E+15	0.338	6.466	43.359	2.021	0.560	29.787	60.551	10.101
1.00E+17	0.494	9.438	63.280	2.950	0.5482	30.053	63.969	10.541
1.00E+19	0.495	9.536	63.574	3.001	0.551	31.465	64.778	11.238
	**PbS (bulk bandgap)**				**PbS (quantum bandgap)**			
1.00E+15	0.510	11.435	62.396	3.644	0.554	27.235	59.989	9.062
1.00E+17	0.504	11.595	63.616	3.717	0.545	28.269	64.147	9.899
1.00E+19	0.504	11.728	63.694	3.770	0.551	31.642	64.794	11.312
	**In**_**2**_**S**_**3**_** (bulk bandgap)**				**In**_**2**_**S**_**3**_** (quantum bandgap)**			
1.00E+15	0.686	29.926	33.541	6.890	0.673	30.798	33.748	6.996
1.00E+17	0.663	31.034	46.300	9.536	0.657	31.963	46.714	9.818
1.00E+19	0.551	31.738	64.014	11.205	0.553	32.668	64.370	11.631
	**CdS (bulk bandgap)**				**CdS (quantum bandgap)**			
1.00E+15	0.558	31.597	61.472	10.843	0.560	31.977	61.105	10.945
1.00E+17	0.552	32.087	64.797	11.479	0.552	32.529	64.936	11.677
1.00E+19	0.553	32.765	65.040	11.794	0.553	32.970	65.093	11.884
	**ZnS (bulk bandgap)**				**ZnS (quantum bandgap)**			
1.00E+15	0.566	32.287	59.503	10.877	0.566	32.277	59.452	10.867
1.00E+17	0.553	32.921	65.068	11.855	0.553	32.954	65.075	11.870
1.00E+19	0.553	33.108	65.149	11.949	0.554	33.141	65.152	11.962

Unlike the bulk-buffer layer, where electrons are more delocalized that is spread out over a larger volume, electrons in the buffer layer-quantum dots are confined to a much smaller volume due to the QD's tiny size. The suitable electron energies in the valence and conduction bands become quantized that is discrete, rather than continuous, therefore, this so-called Quantum Confinement Effect^55^. Only the size of the QD of the applied buffer layer may "tune" the bandgap to the desired value.

### Influence of the donor concentration of the varying buffer layers

**Figure 9:**
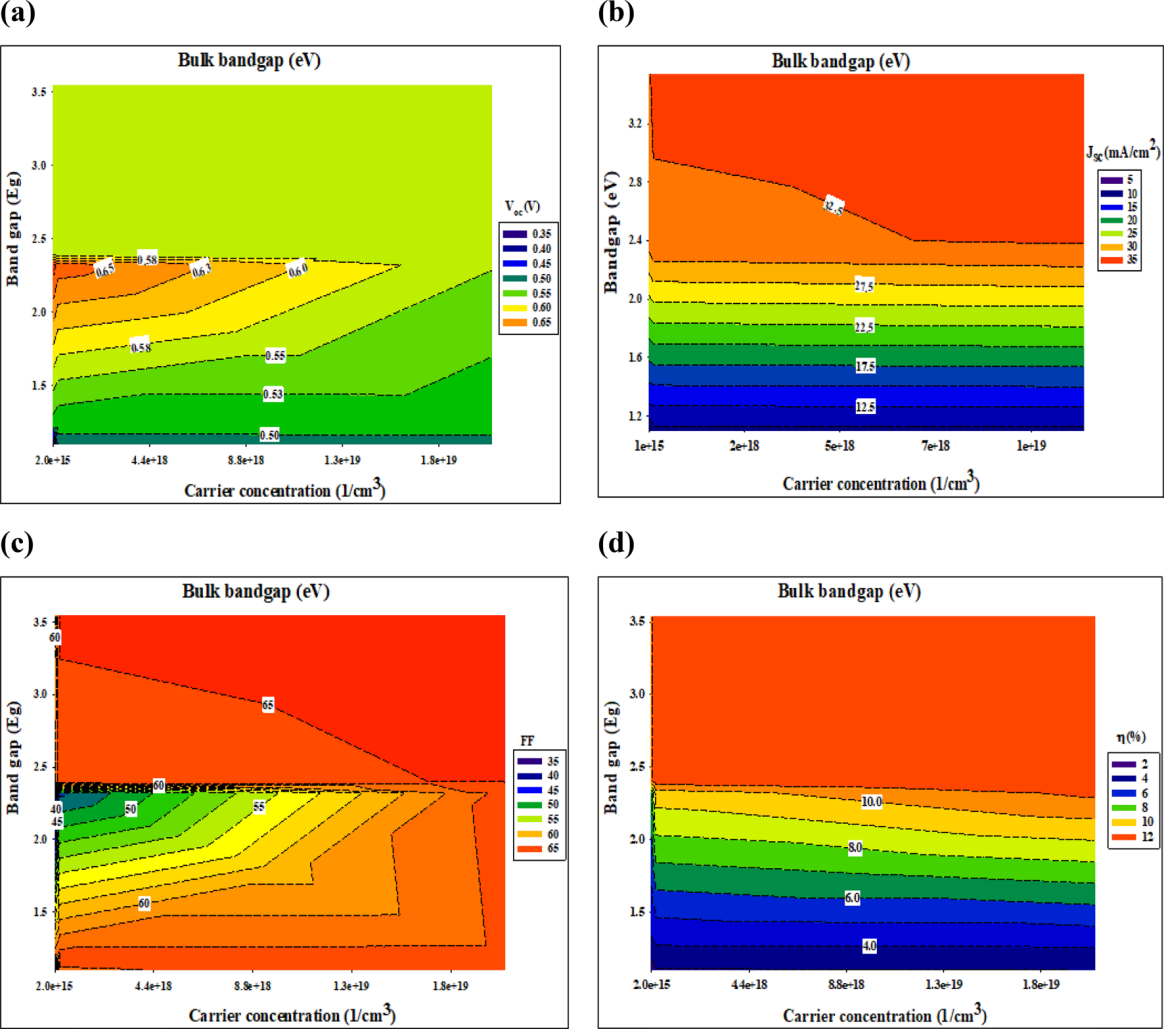
Photovoltaic performance parameters at bulk bandgap.

**Figure 10:**
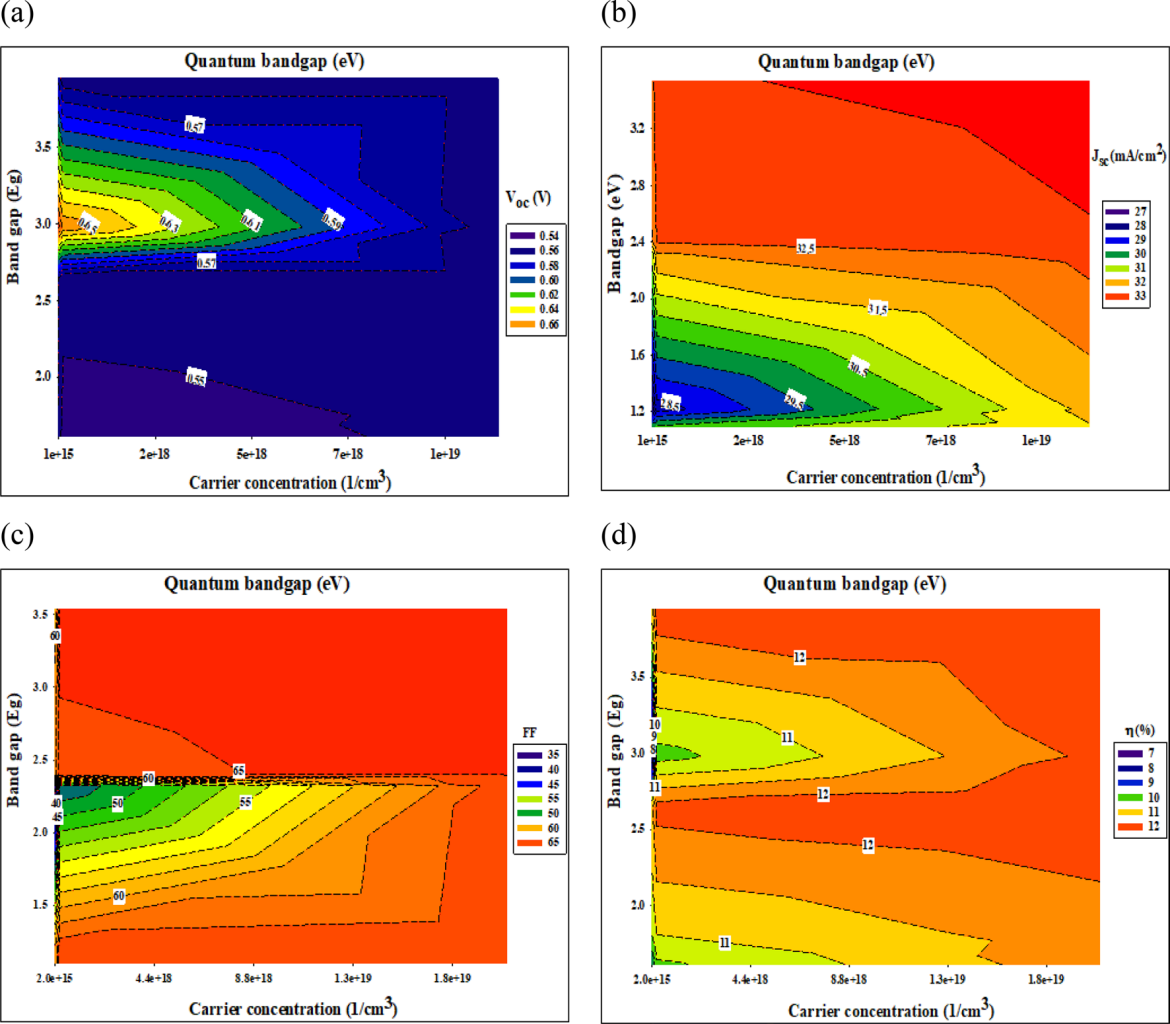
Photovoltaic performance parameters at quantum bandgap.

The attempt has been considering here to delineate the trend impact of the different buffer layers by dissecting the relevant photovoltaic performance parameters, which ultimately govern the solar cell conversion efficiency. Generally, the conversion efficiency is obtained according to Eq. (8). For clarity and simplicity, simulation outcomes pertaining to the buffer layers that is represents the lower and upper limit in terms of bandgap and carrier concentration values were chosen for comparative analysis. Figures 9 and 10 illustrate the *V*_oc_, (a) *J*_sc_, (b) *FF*, (c) *FF,* and (d) *η* for a thin film photovoltaic device with different buffer layer.

When it comes to the transfer of carriers in thin-film solar cells and the recombination of those carriers, the conduction band offset (CBO) at the absorber/buffer interface is one of the most critical issues to consider. Once the absorber layer's electron affinity energy is more or less than the buffer layer's electron affinity energy, the conduction band offset is equal to the difference between the two layers' values. At the interface of the layers' interfaces, it may find configurations of the cliff-type configuration and the spike-type configuration. As soon as the buffer layer's minimum conduction band is lower than (or higher than) the absorber layer's minimum conduction band, the cliff-type (or spike-type) configuration occurs. Those cliffs would operate as a barrier to electron injection from the absorber layer to the photon-generated buffer layer. These cliffs would improve electron accumulation and recombination at the interface between the majority carriers (holes) in the absorber layer and the accumulated electrons. As a consequence, the buffer layer must have a substantial bandgap so as to ensure proper band alignment at the buffer/absorber interface and to enhance the open-circuit voltage (*V*_*oc*_). Once the buffer layer has a higher electron affinity than the absorber layer, the band alignment at the absorber/buffer interface shifts from cliff to spike. Nevertheless, the spike-like band alignment results in less *V*_oc_ reduction, and *V*_oc_ remains almost constant despite an increase in CBO. This observed occurrence is contrary to the outcome of practical measurements whereby a small positive CBO in the range of 0–0.15 eV (at Ag_2_S and PbS buffer layer) conventionally results in higher conversion efficiency and a negative CBO is expected to yield slightly high efficiency (In_2_S_3_, CdS, and ZnS buffer layer). However, these phenomena are not reflected in this study due to the fact that the beneficial effects of a small positive CBO and detrimental effects of a negative CBO only come into play if a *n*-buffer layer/p-absorber hetero-interface recombination mechanism is taken into account.

On the other hand, the *J*_sc_ parameter differs considerably as illustrated in Figs. 9 and 10, mainly in the range between 6 and 33 (mA/cm^2^). Low carrier concentration yields to lower carrier collection at the front contact and thus a lower *J*_sc_. However, buffer layer with a higher carrier concentration could retain a high *J*_sc_ value in the same CBO region. This trend can be noticeable in the Ag_2_S buffer layer. While PbS buffer layer depicted that, even with higher carrier concentration there is no significant enhanced. Therefore, an appropriate band alignment at the buffer/absorber interface (higher carrier concentration and bandgap) for effective solar cells is very important to rise the *J*_sc_. This could be due to the increased diffusivity (*D*_n_) of carriers, induced by higher carrier mobility as governed by the Eq. (9)^48^. In return, increased diffusivity is responsible for longer carrier diffusion length and subsequently higher photogenerated current, *I*_ph_ as evident in the following relationship as shown in Eqs. (10) and (11)^49^. Based on Eq. (12) below, we note that the depletion region width, *W* for a heterojunction consisting of different buffer layers with a higher carrier concentration (*N*_D_: 10^19^ cm^−3^) and optimized bandgap should be lower compared to the depletion width for the buffer layer with a lower carrier concentration (*N*_D_: 10^15^ cm^−3^)^56^.

However, a higher *J*_sc_ value was recorded for both CdS and ZnS, even with the wide depletion width, which was supposed to decrease the photogenerated current according to Eq. (8). This observed occurrence is in agreement with the outcome of practical measurements whereby a small positive CBO conventionally results in higher conversion efficiency^57^. This could be due to the beneficial synergistic effects of the typical bandgap, which enables carriers to overcome a high CBO barrier. This is supported by the fact that only CdS, and ZnS, which represents a buffer layer with the highest bandgap, exhibits almost the same photovoltaic performance parameters across all investigated values. The fill factor varies according to the open-circuit voltage.

We argue that if the optical properties of the ensuing buffer layer are not properly fine-tuned and characterized, it may lead to incorrect assumptions particularly on the true potential of the investigated *p*-absorber material. For example, let us say that CIGS with a bandgap of 1.2 eV and CBO in the range of 0–0.15 eV (obtained according to using Ag_2_S and PbS as a buffer layer) is being investigated. In the preliminary stage of development, it is highly likely for the absorber thin film to possess a bulk defect density, due to its poly-crystalline nature and non-optimized deposition process. If the deposited buffer layer possesses a low bandgap, the corresponding device is predicted to yield efficiency below 2% (see Fig. 9, Table 4). However, the device efficiency can be boosted above by employing a buffer layer with a higher bandgap. It is also evident that a rise in the carrier concentration and bandgap of In_2_S_3_, CdS, and ZnS buffer layer yields a conversion efficiency that seems to be inconspicuously small, Fig. 10.

### Performance of quantum efficiency (QE) %

**Figure 11:**
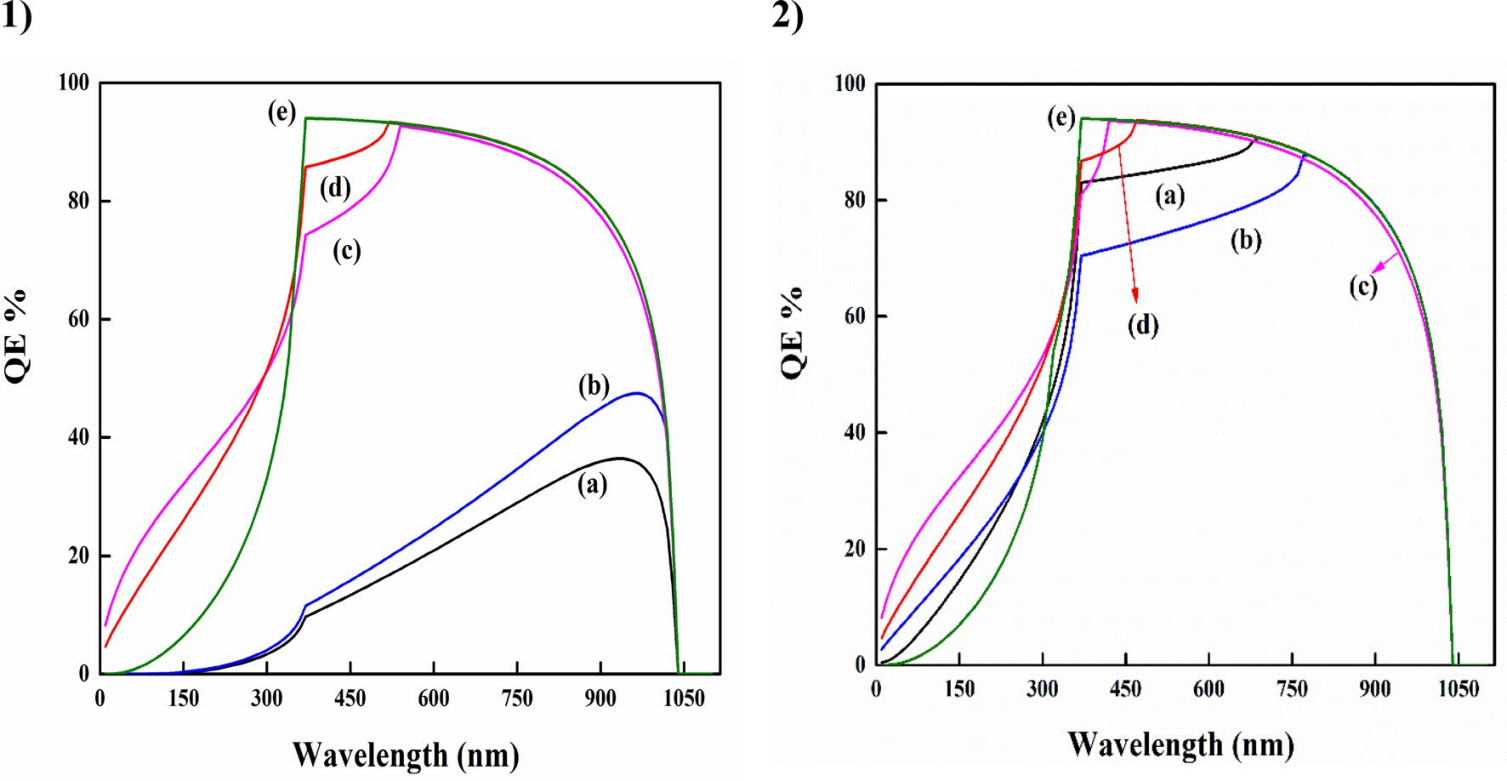
QE% of CIGS solar cells at (**1**) Bulk bandgap, (**2**) Quantum bandgap for; (a) Ag_2_S. (b) PbS, (c) In_2_S_3_, (d) CdS, and (e) ZnS buffer layer.

The quantum efficiency (QE) of an external circuit is defined as the ratio of the current flowing to it to the number of charge carriers incident on it^58,59^. Since the motivation for this study is to simulated high efficiency, a high QE% based on CIGS solar cells is a requirement. Thus, here to investigate the highest possible QE% values, the QE% has been obtained at *N*_D_ = 10^19^ cm^−3^. Figure 11, depicted the performance of QE% versus wavelength with different buffer layers.

Except for Ag_2_S and PbS in the bulk bandgap situation, all devices exhibit a comparable QE response throughout the depletion region at wavelengths below 800 nm. It can see that, the impact of the optical properties for buffer layers on the cell response. At a higher wavelength between 800 and 1000 nm, represents the absorber bulk's quality, the simulated QE % curve is shifted upward. When the bandgap of the buffer layer is raised from 1.1 to 3.54 eV, the ZnS buffer layer exhibits the highest carrier collection efficiency of all the solar cells, with a response of around 93%, indicating less photocurrent interface recombination. This is owing to the increased capture of photons by these buffer layers.

The CdS sample exhibits characteristic absorption in the 400–500 nm short wavelength region of the spectrum. The QE % of both CdS cells (bulk and quantum) is almost the same in the range of 520–1030 nm. The quantum efficiency of CdS begins to decrease below 517 and 468 nm for bulk and quantum, respectively, suggesting that it contributes less to electron generation. By employing ZnS as the buffer material, this current loss may be eliminated. High-energy photons may also create charge carriers in the absorber owing to its larger bandgap and hence higher transmittance. At long wavelengths, the In_2_S_3_ buffer layer shows a reduced response and light absorption losses below 600 nm, respectively.

The buffer layers of both (Ag_2_S and PbS) give interesting responses. Ag_2_S buffer at bulk bandgap shows by far the lowest absorption, Fig. 10. While PbS buffer layer suffers from the interface and bulk recombination^60^. On the contrary, Ag_2_S buffer exhibits significantly enhanced absorption with a larger bandgap, enabling a response comparable to that of In_2_S_3_ above 750 nm and an enhanced response below 750 nm due to decreased light absorption. Thus, the recombination loss of photogenerated minority carriers (i.e., electrons) reduces as well in the CIGS region. The same is true for the PbS buffer layer^61^.

This has been supported by an enhance in carrier concentration, photo-generated minority carrier current density, and depletion layer width. As a result, both J_SC_ and QE % rise up to 800 nm wavelength. These buffer layers could provide the optimal combination of optical and electrical characteristics^62^. Related findings were reported by Priya and Singh, 2021^47^. However, the overall QE for Ag_2_S and PbS levels below 80% is relatively low, which may be due to reflection losses. Besides that, a small spike at the buffer/absorber interface that obstructs electron transport cannot be excluded.

Additionally, solar cells based on CdS, In_2_S_3_ (at the bulk bandgap), and Ag_2_S, PbS, CdS, and In_2_S_3_ (at the quantum bandgap) have the cut-off QE, which contributes to electron generation throughout the visible spectrum. As a result, solar cells with a higher bandgap of buffers achieve better efficiency than bulk solar cells^63^.

During certain wavelengths, all the curves begin to converge on zero, since each material be able to absorb photons only in a narrow region of the visible light spectrum^64^. Further, Fig. 10 demonstrates that the spectral response above 800 nm, named the red response, declines with increasing bulk bandgap values (for example ZnS). This scenario is describable by decreased absorption and a short diffusion length. This behaviour explains why the efficiency does not improve proportionately as the quantum bandgap of ZnS increases, since the bandgap also saturates at a certain point.

## Conclusion

The numerical simulation work presented herein is primarily focused on assessing the impacts of optic-electrical characterizations of the various buffer layer, namely the bandgap and carrier concentration on the performance of SLG/Mo/*p*-CIGS/*n*-buffer layer/*i*-ZnO/Al configured thin-film photovoltaic devices. In general, it was exposed that for the CBO more than 0.15 eV, *J*_sc_ is dependent on the bandgap and carrier concentration of the buffer layer, due to a higher carrier diffusivity and efficient carrier transport across a spike-like CBO barrier. Whereas, *V*_*oc*_ is predominantly dependent on the band structure of different buffer layers, as an appropriate bandgap could avoid the recombination process at the interface between the absorber and buffer layer. Owing to the improved both *J*_sc_ and *V*_oc_, the conversion efficiency of CIGS based on Ag_2_S quantum dot as a buffer layer showed better performance with 11.23% as compared with the CIGS-based ones 3%. While the highest efficiency of 11.96% could be reached due to the ZnS quantum bandgap and the appropriate conduction band offset (CBO). Further investigations on the QE% suggest a suitable bandgap could be used in order to obtain better quantum efficiency. Our visions into the device performance show that, the CIGS solar cells might be tuned via adjusted bandgap and carrier concentration, which is consistent with previous findings. Important design considerations for buffer layers containing highly effective CIGS solar cells may be derived from the findings of this simulation.

## Supplementary Information

Supplementary Information 1.

Supplementary Information 2.

Supplementary Information 3.

Supplementary Information 4.

Supplementary Information 5.

Supplementary Information 6.

Supplementary Information 7.

Supplementary Information 8.

Supplementary Information 9.

Supplementary Information 10.

Supplementary Information 11.

Supplementary Information 12.

Supplementary Information 13.

Supplementary Information 14.

Supplementary Information 15.

Supplementary Information 16.

Supplementary Information 17.
