# Supplementary material for: Numerical simulation of quantum dots as a buffer layer in CIGS solar cells: a comparative study
Source: Sci Rep. 2022 May 16;12:8099. doi: 10.1038/s41598-022-12234-0 (PMC9110751; doi:10.1038/s41598-022-12234-0)
Supplement: Supplementary file 16 — Supplementary Information 16. [file 41598_2022_12234_MOESM16_ESM.docx]

# **CdS nanoparticle (Eg = 2.4 eV)**

SCAPS 3.3.07 ELIS-UGent: Version scaps3307.exe, dated 27-01-2018, 10:11:02

Simulation of I-V curves

Single shot simulation # 1

Calculation started : 16-9-2021 at 14:02:17

problem definition file : c:\Program Files (x86)\Scaps3307\def\example CIGS.def

last saved: 01-09-2015 at 07:44:06

Calculation under illumination

Optical generation from internal SCAPS calculation

Illumination spectrum read from file

spectrum file :

C:\Program Files (x86)\Scaps3307\spectrum\AM1_5G 1 sun.spe last saved: 22-05-2009 at 09:59:48

Neutral density filter: ND 0.0000

Transmission of ND filter: 1.0000e+00

Power from spectrum: 100.0000 mW/cm2

Incident power on solar cell structure 100.0000 mW/cm2

Working point conditions

Temperature 300.00 K

Workpoint bias voltage 0.0000 Volt

Frequency 1.0000e+06 Hz

Voltage reference convention: voltage referred to right contact, voltage V applied to left contact

Current reference convention: current positive when entering the left contact

Power generated by the cell: = -V*I; power consumed by the cell = +V*I

v(V) jtot(mA/cm2) j_total_rec(mA/cm2) j_total_gen(mA/cm2) jbulk(mA/cm2) jifr(mA/cm2) jminor_left(mA/cm2) jminor_right(mA/cm2) j_SRH(mA/cm2) j_Radiative(mA/cm2) j_Auger(mA/cm2)

0.000000 -3.15970770e+01 6.77540545e+00 3.83718858e+01 5.16252081e+00 1.31627930e+00 2.13865013e-01 8.27403307e-02 5.16252081e+00 0.00000000e+00 0.00000000e+00

0.020000 -3.14819263e+01 6.88919808e+00 3.83718858e+01 5.26463076e+00 1.32754392e+00 2.14272710e-01 8.27507008e-02 5.26463076e+00 0.00000000e+00 0.00000000e+00

0.040000 -3.13627249e+01 7.00784031e+00 3.83718858e+01 5.37134824e+00 1.33905178e+00 2.14679115e-01 8.27611666e-02 5.37134824e+00 0.00000000e+00 0.00000000e+00

0.060000 -3.12389346e+01 7.13167185e+00 3.83718858e+01 5.48299776e+00 1.35081809e+00 2.15084197e-01 8.27717976e-02 5.48299776e+00 0.00000000e+00 0.00000000e+00

0.080000 -3.11096212e+01 7.26103215e+00 3.83718858e+01 5.59990000e+00 1.36286164e+00 2.15487771e-01 8.27827329e-02 5.59990000e+00 0.00000000e+00 0.00000000e+00

0.100000 -3.09743268e+01 7.39637399e+00 3.83718858e+01 5.72249661e+00 1.37519345e+00 2.15889933e-01 8.27939955e-02 5.72249661e+00 0.00000000e+00 0.00000000e+00

0.120000 -3.08325332e+01 7.53821814e+00 3.83718858e+01 5.85129665e+00 1.38782509e+00 2.16290786e-01 8.28056088e-02 5.85129665e+00 0.00000000e+00 0.00000000e+00

0.140000 -3.06836277e+01 7.68717266e+00 3.83718858e+01 5.98689591e+00 1.40076872e+00 2.16690438e-01 8.28175981e-02 5.98689591e+00 0.00000000e+00 0.00000000e+00

0.160000 -3.05268862e+01 7.84396706e+00 3.83718858e+01 6.13001093e+00 1.41403714e+00 2.17088998e-01 8.28299939e-02 6.13001093e+00 0.00000000e+00 0.00000000e+00

0.180000 -3.03614017e+01 8.00950504e+00 3.83718858e+01 6.28153180e+00 1.42764383e+00 2.17486580e-01 8.28428281e-02 6.28153180e+00 0.00000000e+00 0.00000000e+00

0.200000 -3.01860140e+01 8.18494927e+00 3.83718858e+01 6.44260689e+00 1.44160294e+00 2.17883302e-01 8.28561363e-02 6.44260689e+00 0.00000000e+00 0.00000000e+00

0.220000 -2.99991622e+01 8.37186138e+00 3.83718858e+01 6.61478284e+00 1.45592930e+00 2.18279284e-01 8.28699579e-02 6.61478284e+00 0.00000000e+00 0.00000000e+00

0.240000 -2.97986504e+01 8.57242760e+00 3.83718858e+01 6.80023013e+00 1.47063847e+00 2.18674655e-01 8.28843373e-02 6.80023013e+00 0.00000000e+00 0.00000000e+00

0.260000 -2.95807002e+01 8.78971519e+00 3.83718858e+01 7.00199896e+00 1.48574750e+00 2.19069308e-01 8.28994268e-02 7.00199896e+00 0.00000000e+00 0.00000000e+00

0.280000 -2.93419175e+01 9.02861885e+00 3.83718858e+01 7.22496890e+00 1.50127099e+00 2.19463892e-01 8.29150698e-02 7.22496890e+00 0.00000000e+00 0.00000000e+00

0.300000 -2.90746129e+01 9.29605993e+00 3.83718858e+01 7.47604212e+00 1.51722807e+00 2.19858299e-01 8.29314420e-02 7.47604212e+00 0.00000000e+00 0.00000000e+00

0.320000 -2.87682848e+01 9.60253215e+00 3.83718858e+01 7.76569403e+00 1.53363680e+00 2.20252704e-01 8.29486155e-02 7.76569403e+00 0.00000000e+00 0.00000000e+00

0.340000 -2.84073879e+01 9.96357202e+00 3.83718858e+01 8.10944240e+00 1.55051562e+00 2.20647313e-01 8.29666751e-02 8.10944240e+00 0.00000000e+00 0.00000000e+00

0.360000 -2.79692276e+01 1.04018812e+01 3.83718858e+01 8.52996988e+00 1.56788325e+00 2.21042383e-01 8.29857130e-02 8.52996988e+00 0.00000000e+00 0.00000000e+00

0.380000 -2.74205538e+01 1.09500260e+01 3.83718858e+01 9.05982646e+00 1.58575560e+00 2.21438059e-01 8.30059123e-02 9.05982646e+00 0.00000000e+00 0.00000000e+00

0.400000 -2.67159340e+01 1.16549932e+01 3.83718858e+01 9.74597336e+00 1.60415737e+00 2.21835253e-01 8.30272145e-02 9.74597336e+00 0.00000000e+00 0.00000000e+00

0.420000 -2.57884403e+01 1.25821360e+01 3.83718858e+01 1.06537522e+01 1.62309963e+00 2.22234212e-01 8.30499326e-02 1.06537522e+01 0.00000000e+00 0.00000000e+00

0.440000 -2.45472962e+01 1.38237103e+01 3.83718858e+01 1.18753923e+01 1.64260756e+00 2.22636432e-01 8.30740568e-02 1.18753923e+01 0.00000000e+00 0.00000000e+00

0.460000 -2.28655414e+01 1.55067323e+01 3.83718858e+01 1.35378879e+01 1.66270111e+00 2.23043615e-01 8.30997452e-02 1.35378879e+01 0.00000000e+00 0.00000000e+00

0.480000 -2.05685892e+01 1.78035794e+01 3.83718858e+01 1.58136168e+01 1.68337713e+00 2.23458054e-01 8.31274202e-02 1.58136168e+01 0.00000000e+00 0.00000000e+00

0.500000 -1.74237362e+01 2.09486257e+01 3.83718858e+01 1.89369219e+01 1.70466164e+00 2.23885055e-01 8.31571038e-02 1.89369219e+01 0.00000000e+00 0.00000000e+00

0.520000 -1.31185765e+01 2.52539123e+01 3.83718858e+01 2.32198267e+01 1.72656369e+00 2.24332906e-01 8.31890442e-02 2.32198267e+01 0.00000000e+00 0.00000000e+00

0.540000 -7.23748730e+00 3.11350300e+01 3.83718858e+01 2.90778957e+01 1.74909443e+00 2.24816325e-01 8.32234605e-02 2.90778957e+01 0.00000000e+00 0.00000000e+00

0.560000 7.78078733e-01 3.91505341e+01 3.83718858e+01 3.70696460e+01 1.77226602e+00 2.25361526e-01 8.32605758e-02 3.70696460e+01 0.00000000e+00 0.00000000e+00

0.580000 1.16984255e+01 5.00707898e+01 3.83718858e+01 4.79653791e+01 1.79609424e+00 2.26015866e-01 8.33005986e-02 4.79653791e+01 0.00000000e+00 0.00000000e+00

0.600000 2.66266732e+01 6.49988945e+01 3.83718858e+01 6.28680817e+01 1.82060313e+00 2.26865900e-01 8.33437145e-02 6.28680817e+01 0.00000000e+00 0.00000000e+00

0.620000 4.71997097e+01 8.55717548e+01 3.83718858e+01 8.34144617e+01 1.84583212e+00 2.28070935e-01 8.33900635e-02 8.34144617e+01 0.00000000e+00 0.00000000e+00

0.640000 7.58988358e+01 1.14270715e+02 3.83718858e+01 1.12085505e+02 1.87184620e+00 2.29924527e-01 8.34397088e-02 1.12085505e+02 0.00000000e+00 0.00000000e+00

0.660000 1.16501496e+02 1.54873251e+02 3.83718858e+01 1.52658044e+02 1.89874916e+00 2.32965055e-01 8.34926024e-02 1.52658044e+02 0.00000000e+00 0.00000000e+00

0.680000 1.74711280e+02 2.13082971e+02 3.83718858e+01 2.10834554e+02 1.92669856e+00 2.38170102e-01 8.35485772e-02 2.10834554e+02 0.00000000e+00 0.00000000e+00

0.700000 2.58986019e+02 2.97356790e+02 3.83718858e+01 2.95069967e+02 1.95592787e+00 2.47287344e-01 8.36072767e-02 2.95069967e+02 0.00000000e+00 0.00000000e+00

0.720000 3.81645961e+02 4.20015003e+02 3.83718858e+01 4.17681223e+02 1.98672377e+00 2.63387140e-01 8.36682483e-02 4.17681223e+02 0.00000000e+00 0.00000000e+00

0.740000 5.59860038e+02 5.98232690e+02 3.83718858e+01 5.95837757e+02 2.01948296e+00 2.91718940e-01 8.37307741e-02 5.95837757e+02 0.00000000e+00 0.00000000e+00

0.760000 8.16561163e+02 8.54941096e+02 3.83718858e+01 8.52461624e+02 2.05467288e+00 3.41005730e-01 8.37940194e-02 8.52461624e+02 0.00000000e+00 0.00000000e+00

0.780000 1.18046260e+03 1.21882328e+03 3.83718858e+01 1.21622133e+03 2.09281434e+00 4.25280044e-01 8.38570524e-02 1.21622133e+03 0.00000000e+00 0.00000000e+00

0.800000 1.68384361e+03 1.72225007e+03 3.83718858e+01 1.71946541e+03 2.13441438e+00 5.66330057e-01 8.39188445e-02 1.71946541e+03 0.00000000e+00 0.00000000e+00

solar cell parameters deduced from calculated IV-curve:

Voc = 0.558269 Volt

Jsc = 31.59707704 mA/cm2

FF = 61.4720 %

eta = 10.8435 %

V_MPP = 0.427246 Volt

J_MPP = 25.37990887 mA/cm2

SCAPS 3.3.07 ELIS-UGent: Version scaps3307.exe, dated 27-01-2018, 10:11:02

Simulation of I-V curves

Single shot simulation # 2

Calculation started : 16-9-2021 at 14:02:57

problem definition file : c:\Program Files (x86)\Scaps3307\def\example CIGS.def

last saved: 01-09-2015 at 07:44:06

Calculation under illumination

Optical generation from internal SCAPS calculation

Illumination spectrum read from file

spectrum file :

C:\Program Files (x86)\Scaps3307\spectrum\AM1_5G 1 sun.spe last saved: 22-05-2009 at 09:59:48

Neutral density filter: ND 0.0000

Transmission of ND filter: 1.0000e+00

Power from spectrum: 100.0000 mW/cm2

Incident power on solar cell structure 100.0000 mW/cm2

Working point conditions

Temperature 300.00 K

Workpoint bias voltage 0.0000 Volt

Frequency 1.0000e+06 Hz

Voltage reference convention: voltage referred to right contact, voltage V applied to left contact

Current reference convention: current positive when entering the left contact

Power generated by the cell: = -V*I; power consumed by the cell = +V*I

v(V) jtot(mA/cm2) j_total_rec(mA/cm2) j_total_gen(mA/cm2) jbulk(mA/cm2) jifr(mA/cm2) jminor_left(mA/cm2) jminor_right(mA/cm2) j_SRH(mA/cm2) j_Radiative(mA/cm2) j_Auger(mA/cm2)

0.000000 -3.20878560e+01 6.28386860e+00 3.83718858e+01 4.77275771e+00 1.21363681e+00 2.09154976e-01 8.83191043e-02 4.77275771e+00 0.00000000e+00 0.00000000e+00

0.020000 -3.19876472e+01 6.38426810e+00 3.83718858e+01 4.86632117e+00 1.22003556e+00 2.09592136e-01 8.83192325e-02 4.86632117e+00 0.00000000e+00 0.00000000e+00

0.040000 -3.18829362e+01 6.48898762e+00 3.83718858e+01 4.96413153e+00 1.22650947e+00 2.10027262e-01 8.83193585e-02 4.96413153e+00 0.00000000e+00 0.00000000e+00

0.060000 -3.17735717e+01 6.59836644e+00 3.83718858e+01 5.06652698e+00 1.23305952e+00 2.10460464e-01 8.83194828e-02 5.06652698e+00 0.00000000e+00 0.00000000e+00

0.080000 -3.16591678e+01 6.71277906e+00 3.83718858e+01 5.17388086e+00 1.23968675e+00 2.10891841e-01 8.83196052e-02 5.17388086e+00 0.00000000e+00 0.00000000e+00

0.100000 -3.15393208e+01 6.83264145e+00 3.83718858e+01 5.28660800e+00 1.24639223e+00 2.11321492e-01 8.83197257e-02 5.28660800e+00 0.00000000e+00 0.00000000e+00

0.120000 -3.14135511e+01 6.95842372e+00 3.83718858e+01 5.40517734e+00 1.25317701e+00 2.11749523e-01 8.83198445e-02 5.40517734e+00 0.00000000e+00 0.00000000e+00

0.140000 -3.12813252e+01 7.09066778e+00 3.83718858e+01 5.53012957e+00 1.26004220e+00 2.12176044e-01 8.83199616e-02 5.53012957e+00 0.00000000e+00 0.00000000e+00

0.160000 -3.11419993e+01 7.23000604e+00 3.83718858e+01 5.66209587e+00 1.26698892e+00 2.12601174e-01 8.83200769e-02 5.66209587e+00 0.00000000e+00 0.00000000e+00

0.180000 -3.09948363e+01 7.37718471e+00 3.83718858e+01 5.80182118e+00 1.27401830e+00 2.13025035e-01 8.83201904e-02 5.80182118e+00 0.00000000e+00 0.00000000e+00

0.200000 -3.08389397e+01 7.53310555e+00 3.83718858e+01 5.95020597e+00 1.28113152e+00 2.13447760e-01 8.83203021e-02 5.95020597e+00 0.00000000e+00 0.00000000e+00

0.220000 -3.06731681e+01 7.69890212e+00 3.83718858e+01 6.10838245e+00 1.28832976e+00 2.13869489e-01 8.83204120e-02 6.10838245e+00 0.00000000e+00 0.00000000e+00

0.240000 -3.04960232e+01 7.87606826e+00 3.83718858e+01 6.27784311e+00 1.29561425e+00 2.14290373e-01 8.83205203e-02 6.27784311e+00 0.00000000e+00 0.00000000e+00

0.260000 -3.03054383e+01 8.06667544e+00 3.83718858e+01 6.46065799e+00 1.30298624e+00 2.14710575e-01 8.83206271e-02 6.46065799e+00 0.00000000e+00 0.00000000e+00

0.280000 -3.00983991e+01 8.27375256e+00 3.83718858e+01 6.65985455e+00 1.31044700e+00 2.15130276e-01 8.83207323e-02 6.65985455e+00 0.00000000e+00 0.00000000e+00

0.300000 -2.98702320e+01 8.50193838e+00 3.83718858e+01 6.88007003e+00 1.31799784e+00 2.15549677e-01 8.83208360e-02 6.88007003e+00 0.00000000e+00 0.00000000e+00

0.320000 -2.96136537e+01 8.75854857e+00 3.83718858e+01 7.12861853e+00 1.32564009e+00 2.15969012e-01 8.83209381e-02 7.12861853e+00 0.00000000e+00 0.00000000e+00

0.340000 -2.93169878e+01 9.05524999e+00 3.83718858e+01 7.41716529e+00 1.33337511e+00 2.16388559e-01 8.83210386e-02 7.41716529e+00 0.00000000e+00 0.00000000e+00

0.360000 -2.89616740e+01 9.41059904e+00 3.83718858e+01 7.76426494e+00 1.34120429e+00 2.16808672e-01 8.83211367e-02 7.76426494e+00 0.00000000e+00 0.00000000e+00

0.380000 -2.85185271e+01 9.85376519e+00 3.83718858e+01 8.19908510e+00 1.34912904e+00 2.17229820e-01 8.83212344e-02 8.19908510e+00 0.00000000e+00 0.00000000e+00

0.400000 -2.79423994e+01 1.04299171e+01 3.83718858e+01 8.76679231e+00 1.35715078e+00 2.17652672e-01 8.83213303e-02 8.76679231e+00 0.00000000e+00 0.00000000e+00

0.420000 -2.71641616e+01 1.12081620e+01 3.83718858e+01 9.53649139e+00 1.36527095e+00 2.18078244e-01 8.83214256e-02 9.53649139e+00 0.00000000e+00 0.00000000e+00

0.440000 -2.60787029e+01 1.22936236e+01 3.83718858e+01 1.06133029e+01 1.37349094e+00 2.18508182e-01 8.83215200e-02 1.06133029e+01 0.00000000e+00 0.00000000e+00

0.460000 -2.45261941e+01 1.38461061e+01 3.83718858e+01 1.21570271e+01 1.38181214e+00 2.18945306e-01 8.83216132e-02 1.21570271e+01 0.00000000e+00 0.00000000e+00

0.480000 -2.22633318e+01 1.61089193e+01 3.83718858e+01 1.44109671e+01 1.39023582e+00 2.19394673e-01 8.83217056e-02 1.44109671e+01 0.00000000e+00 0.00000000e+00

0.500000 -1.89188818e+01 1.94532807e+01 3.83718858e+01 1.77463301e+01 1.39876311e+00 2.19865678e-01 8.83217990e-02 1.77463301e+01 0.00000000e+00 0.00000000e+00

0.520000 -1.39251245e+01 2.44469019e+01 3.83718858e+01 2.27308089e+01 1.40739486e+00 2.20376192e-01 8.83218926e-02 2.27308089e+01 0.00000000e+00 0.00000000e+00

0.540000 -6.41132778e+00 3.19604968e+01 3.83718858e+01 3.02350825e+01 1.41613148e+00 2.20960805e-01 8.83219891e-02 3.02350825e+01 0.00000000e+00 0.00000000e+00

0.560000 4.96213498e+00 4.33337663e+01 3.83718858e+01 4.15987844e+01 1.42497264e+00 2.21687191e-01 8.83220886e-02 4.15987844e+01 0.00000000e+00 0.00000000e+00

0.580000 2.22622549e+01 6.06338917e+01 3.83718858e+01 5.88889640e+01 1.43391688e+00 2.22688603e-01 8.83221941e-02 5.88889640e+01 0.00000000e+00 0.00000000e+00

0.600000 4.86842747e+01 8.70559702e+01 3.83718858e+01 8.53004592e+01 1.44296079e+00 2.24227944e-01 8.83223145e-02 8.53004592e+01 0.00000000e+00 0.00000000e+00

0.620000 8.91642488e+01 1.27535898e+02 3.83718858e+01 1.25768653e+02 1.45209835e+00 2.26824354e-01 8.83224540e-02 1.25768653e+02 0.00000000e+00 0.00000000e+00

0.640000 1.51297997e+02 1.89669358e+02 3.83718858e+01 1.87888219e+02 1.46131902e+00 2.31497811e-01 8.83226251e-02 1.87888219e+02 0.00000000e+00 0.00000000e+00

0.660000 2.46666241e+02 2.85035981e+02 3.83718858e+01 2.83236815e+02 1.47060585e+00 2.40237616e-01 8.83228434e-02 2.83236815e+02 0.00000000e+00 0.00000000e+00

0.680000 3.92611773e+02 4.30972884e+02 3.83718858e+01 4.29147753e+02 1.47993263e+00 2.56874895e-01 8.83231346e-02 4.29147753e+02 0.00000000e+00 0.00000000e+00

0.700000 6.14248126e+02 6.52622767e+02 3.83718858e+01 6.50756533e+02 1.48926058e+00 2.88650129e-01 8.83235376e-02 6.50756533e+02 0.00000000e+00 0.00000000e+00

0.720000 9.46224327e+02 9.84618688e+02 3.83718858e+01 9.82682943e+02 1.49853678e+00 3.48884058e-01 8.83241027e-02 9.82682943e+02 0.00000000e+00 0.00000000e+00

0.740000 1.43263699e+03 1.47098839e+03 3.83718858e+01 1.46893113e+03 1.50769529e+00 4.61230735e-01 8.83248937e-02 1.46893113e+03 0.00000000e+00 0.00000000e+00

0.760000 2.12324037e+03 2.16143146e+03 3.83718858e+01 2.15916059e+03 1.51666346e+00 6.65879208e-01 8.83259850e-02 2.15916059e+03 0.00000000e+00 0.00000000e+00

0.780000 3.06446114e+03 3.10316096e+03 3.83718858e+01 3.10051951e+03 1.52537290e+00 1.02775275e+00 8.83274524e-02 3.10051951e+03 0.00000000e+00 0.00000000e+00

0.800000 4.29385856e+03 4.33149091e+03 3.83718858e+01 4.32822149e+03 1.53378878e+00 1.64730288e+00 8.83293335e-02 4.32822149e+03 0.00000000e+00 0.00000000e+00

solar cell parameters deduced from calculated IV-curve:

Voc = 0.552086 Volt

Jsc = 32.08785596 mA/cm2

FF = 64.7972 %

eta = 11.4790 %

V_MPP = 0.436191 Volt

J_MPP = 26.31640275 mA/cm2

SCAPS 3.3.07 ELIS-UGent: Version scaps3307.exe, dated 27-01-2018, 10:11:02

Simulation of I-V curves

Single shot simulation # 3

Calculation started : 16-9-2021 at 14:03:25

problem definition file : c:\Program Files (x86)\Scaps3307\def\example CIGS.def

last saved: 01-09-2015 at 07:44:06

Calculation under illumination

Optical generation from internal SCAPS calculation

Illumination spectrum read from file

spectrum file :

C:\Program Files (x86)\Scaps3307\spectrum\AM1_5G 1 sun.spe last saved: 22-05-2009 at 09:59:48

Neutral density filter: ND 0.0000

Transmission of ND filter: 1.0000e+00

Power from spectrum: 100.0000 mW/cm2

Incident power on solar cell structure 100.0000 mW/cm2

Working point conditions

Temperature 300.00 K

Workpoint bias voltage 0.0000 Volt

Frequency 1.0000e+06 Hz

Voltage reference convention: voltage referred to right contact, voltage V applied to left contact

Current reference convention: current positive when entering the left contact

Power generated by the cell: = -V*I; power consumed by the cell = +V*I

v(V) jtot(mA/cm2) j_total_rec(mA/cm2) j_total_gen(mA/cm2) jbulk(mA/cm2) jifr(mA/cm2) jminor_left(mA/cm2) jminor_right(mA/cm2) j_SRH(mA/cm2) j_Radiative(mA/cm2) j_Auger(mA/cm2)

0.000000 -3.27657925e+01 5.60591774e+00 3.83718858e+01 4.88671545e+00 3.90478494e-01 2.07361534e-01 1.21362270e-01 4.88671545e+00 0.00000000e+00 0.00000000e+00

0.020000 -3.26681466e+01 5.70378723e+00 3.83718858e+01 4.98373962e+00 3.90849238e-01 2.07836101e-01 1.21362270e-01 4.98373962e+00 0.00000000e+00 0.00000000e+00

0.040000 -3.25658506e+01 5.80609485e+00 3.83718858e+01 5.08520253e+00 3.91222025e-01 2.08308020e-01 1.21362270e-01 5.08520253e+00 0.00000000e+00 0.00000000e+00

0.060000 -3.24587782e+01 5.91317993e+00 3.83718858e+01 5.19144338e+00 3.91596878e-01 2.08777408e-01 1.21362271e-01 5.19144338e+00 0.00000000e+00 0.00000000e+00

0.080000 -3.23465522e+01 6.02541948e+00 3.83718858e+01 5.30283903e+00 3.91973816e-01 2.09244368e-01 1.21362271e-01 5.30283903e+00 0.00000000e+00 0.00000000e+00

0.100000 -3.22287432e+01 6.14324329e+00 3.83718858e+01 5.41981915e+00 3.92352861e-01 2.09709008e-01 1.21362271e-01 5.41981915e+00 0.00000000e+00 0.00000000e+00

0.120000 -3.21048553e+01 6.26714732e+00 3.83718858e+01 5.54287957e+00 3.92734033e-01 2.10171442e-01 1.21362271e-01 5.54287957e+00 0.00000000e+00 0.00000000e+00

0.140000 -3.19743168e+01 6.39770331e+00 3.83718858e+01 5.67259189e+00 3.93117355e-01 2.10631786e-01 1.21362271e-01 5.67259189e+00 0.00000000e+00 0.00000000e+00

0.160000 -3.18364692e+01 6.53556998e+00 3.83718858e+01 5.80961470e+00 3.93502848e-01 2.11090166e-01 1.21362271e-01 5.80961470e+00 0.00000000e+00 0.00000000e+00

0.180000 -3.16905436e+01 6.68151646e+00 3.83718858e+01 5.95471694e+00 3.93890534e-01 2.11546714e-01 1.21362271e-01 5.95471694e+00 0.00000000e+00 0.00000000e+00

0.200000 -3.15356169e+01 6.83646603e+00 3.83718858e+01 6.10882176e+00 3.94280436e-01 2.12001568e-01 1.21362271e-01 6.10882176e+00 0.00000000e+00 0.00000000e+00

0.220000 -3.13705422e+01 7.00156589e+00 3.83718858e+01 6.27307616e+00 3.94672577e-01 2.12454876e-01 1.21362272e-01 6.27307616e+00 0.00000000e+00 0.00000000e+00

0.240000 -3.11938343e+01 7.17830118e+00 3.83718858e+01 6.44896513e+00 3.95066978e-01 2.12906798e-01 1.21362272e-01 6.44896513e+00 0.00000000e+00 0.00000000e+00

0.260000 -3.10034645e+01 7.36870116e+00 3.83718858e+01 6.63851772e+00 3.95463665e-01 2.13357506e-01 1.21362272e-01 6.63851772e+00 0.00000000e+00 0.00000000e+00

0.280000 -3.07964855e+01 7.57571290e+00 3.83718858e+01 6.84468078e+00 3.95862661e-01 2.13807186e-01 1.21362272e-01 6.84468078e+00 0.00000000e+00 0.00000000e+00

0.300000 -3.05683926e+01 7.80384074e+00 3.83718858e+01 7.07195843e+00 3.96263989e-01 2.14256049e-01 1.21362272e-01 7.07195843e+00 0.00000000e+00 0.00000000e+00

0.320000 -3.03120763e+01 8.06019371e+00 3.83718858e+01 7.32745943e+00 3.96667674e-01 2.14704338e-01 1.21362273e-01 7.32745943e+00 0.00000000e+00 0.00000000e+00

0.340000 -3.00161521e+01 8.35615504e+00 3.83718858e+01 7.62256668e+00 3.97073740e-01 2.15152341e-01 1.21362272e-01 7.62256668e+00 0.00000000e+00 0.00000000e+00

0.360000 -2.96623991e+01 8.70994409e+00 3.83718858e+01 7.97549918e+00 3.97482211e-01 2.15600425e-01 1.21362273e-01 7.97549918e+00 0.00000000e+00 0.00000000e+00

0.380000 -2.92220051e+01 9.15037013e+00 3.83718858e+01 8.41506567e+00 3.97893112e-01 2.16049080e-01 1.21362274e-01 8.41506567e+00 0.00000000e+00 0.00000000e+00

0.400000 -2.86501761e+01 9.72222454e+00 3.83718858e+01 8.98605679e+00 3.98306465e-01 2.16499010e-01 1.21362274e-01 8.98605679e+00 0.00000000e+00 0.00000000e+00

0.420000 -2.78781987e+01 1.04942168e+01 3.83718858e+01 9.75718098e+00 3.98722293e-01 2.16951297e-01 1.21362275e-01 9.75718098e+00 0.00000000e+00 0.00000000e+00

0.440000 -2.68013056e+01 1.15711097e+01 3.83718858e+01 1.08331991e+01 3.99140613e-01 2.17407713e-01 1.21362275e-01 1.08331991e+01 0.00000000e+00 0.00000000e+00

0.460000 -2.52598405e+01 1.31125523e+01 3.83718858e+01 1.23737572e+01 3.99561443e-01 2.17871336e-01 1.21362277e-01 1.23737572e+01 0.00000000e+00 0.00000000e+00

0.480000 -2.30102128e+01 1.53621249e+01 3.83718858e+01 1.46224301e+01 3.99984791e-01 2.18347747e-01 1.21362280e-01 1.46224301e+01 0.00000000e+00 0.00000000e+00

0.500000 -1.96803950e+01 1.86918404e+01 3.83718858e+01 1.79512201e+01 4.00410658e-01 2.18847399e-01 1.21362284e-01 1.79512201e+01 0.00000000e+00 0.00000000e+00

0.520000 -1.47015133e+01 2.36705552e+01 3.83718858e+01 2.29289635e+01 4.00839029e-01 2.19390320e-01 1.21362289e-01 2.29289635e+01 0.00000000e+00 0.00000000e+00

0.540000 -7.20172130e+00 3.11701045e+01 3.83718858e+01 3.04274568e+01 4.01269866e-01 2.20015466e-01 1.21362298e-01 3.04274568e+01 0.00000000e+00 0.00000000e+00

0.560000 4.15966560e+00 4.25312067e+01 3.83718858e+01 4.17873420e+01 4.01703093e-01 2.20799358e-01 1.21362310e-01 4.17873420e+01 0.00000000e+00 0.00000000e+00

0.580000 2.14517507e+01 5.98231715e+01 3.83718858e+01 5.90777774e+01 4.02138573e-01 2.21893146e-01 1.21362328e-01 5.90777774e+01 0.00000000e+00 0.00000000e+00

0.600000 4.78766660e+01 8.62483644e+01 3.83718858e+01 8.55008301e+01 4.02576084e-01 2.23595871e-01 1.21362357e-01 8.55008301e+01 0.00000000e+00 0.00000000e+00

0.620000 8.83926386e+01 1.26764297e+02 3.83718858e+01 1.26013420e+02 4.03015294e-01 2.26499416e-01 1.21362400e-01 1.26013420e+02 0.00000000e+00 0.00000000e+00

0.640000 1.50650161e+02 1.89021551e+02 3.83718858e+01 1.88264963e+02 4.03455583e-01 2.31769766e-01 1.21362467e-01 1.88264963e+02 0.00000000e+00 0.00000000e+00

0.660000 2.46357158e+02 2.84726994e+02 3.83718858e+01 2.83960047e+02 4.03896080e-01 2.41687995e-01 1.21362570e-01 2.83960047e+02 0.00000000e+00 0.00000000e+00

0.680000 3.93136760e+02 4.31498179e+02 3.83718858e+01 4.30711815e+02 4.04335452e-01 2.60664949e-01 1.21362728e-01 4.30711815e+02 0.00000000e+00 0.00000000e+00

0.700000 6.16695943e+02 6.55070564e+02 3.83718858e+01 6.54247346e+02 4.04771720e-01 2.97082758e-01 1.21362970e-01 6.54247346e+02 0.00000000e+00 0.00000000e+00

0.720000 9.52862684e+02 9.91257373e+02 3.83718858e+01 9.90364328e+02 4.05202195e-01 3.66480319e-01 1.21363332e-01 9.90364328e+02 0.00000000e+00 0.00000000e+00

0.740000 1.44787937e+03 1.48622948e+03 3.83718858e+01 1.48520576e+03 4.05623480e-01 4.96730341e-01 1.21363865e-01 1.48520576e+03 0.00000000e+00 0.00000000e+00

0.760000 2.15492308e+03 2.19309163e+03 3.83718858e+01 2.19182844e+03 4.06031766e-01 7.35796065e-01 1.21364625e-01 2.19182844e+03 0.00000000e+00 0.00000000e+00

0.780000 3.12475340e+03 3.16354077e+03 3.83718858e+01 3.16185069e+03 4.06423317e-01 1.16229354e+00 1.21365674e-01 3.16185069e+03 0.00000000e+00 0.00000000e+00

0.800000 4.39885951e+03 4.43764576e+03 3.83718858e+01 4.43521789e+03 4.06795956e-01 1.89971139e+00 1.21367046e-01 4.43521789e+03 0.00000000e+00 0.00000000e+00

solar cell parameters deduced from calculated IV-curve:

Voc = 0.553427 Volt

Jsc = 32.76579250 mA/cm2

FF = 65.0406 %

eta = 11.7941 %

V_MPP = 0.437754 Volt

J_MPP = 26.94236722 mA/cm2

# **CdS QDs (Eg = 2.65 eV)**

SCAPS 3.3.07 ELIS-UGent: Version scaps3307.exe, dated 27-01-2018, 10:11:02

Simulation of I-V curves

Single shot simulation # 1

Calculation started : 16-9-2021 at 14:06:44

problem definition file : c:\Program Files (x86)\Scaps3307\def\example CIGS.def

last saved: 01-09-2015 at 07:44:06

Calculation under illumination

Optical generation from internal SCAPS calculation

Illumination spectrum read from file

spectrum file :

C:\Program Files (x86)\Scaps3307\spectrum\AM1_5G 1 sun.spe last saved: 22-05-2009 at 09:59:48

Neutral density filter: ND 0.0000

Transmission of ND filter: 1.0000e+00

Power from spectrum: 100.0000 mW/cm2

Incident power on solar cell structure 100.0000 mW/cm2

Working point conditions

Temperature 300.00 K

Workpoint bias voltage 0.0000 Volt

Frequency 1.0000e+06 Hz

Voltage reference convention: voltage referred to right contact, voltage V applied to left contact

Current reference convention: current positive when entering the left contact

Power generated by the cell: = -V*I; power consumed by the cell = +V*I

v(V) jtot(mA/cm2) j_total_rec(mA/cm2) j_total_gen(mA/cm2) jbulk(mA/cm2) jifr(mA/cm2) jminor_left(mA/cm2) jminor_right(mA/cm2) j_SRH(mA/cm2) j_Radiative(mA/cm2) j_Auger(mA/cm2)

0.000000 -3.19773867e+01 6.39855581e+00 3.83752279e+01 5.23193237e+00 8.69712233e-01 2.14263866e-01 8.26473383e-02 5.23193237e+00 0.00000000e+00 0.00000000e+00

0.020000 -3.18627902e+01 6.51175886e+00 3.83752279e+01 5.33654838e+00 8.77880385e-01 2.14670871e-01 8.26592219e-02 5.33654838e+00 0.00000000e+00 0.00000000e+00

0.040000 -3.17445290e+01 6.63004310e+00 3.83752279e+01 5.44606533e+00 8.86229499e-01 2.15077274e-01 8.26709909e-02 5.44606533e+00 0.00000000e+00 0.00000000e+00

0.060000 -3.16206669e+01 6.75348941e+00 3.83752279e+01 5.56054202e+00 8.94782259e-01 2.15481959e-01 8.26831722e-02 5.56054202e+00 0.00000000e+00 0.00000000e+00

0.080000 -3.14915501e+01 6.88265207e+00 3.83752279e+01 5.68053026e+00 9.03540684e-01 2.15885571e-01 8.26955572e-02 5.68053026e+00 0.00000000e+00 0.00000000e+00

0.100000 -3.13563201e+01 7.01793111e+00 3.83752279e+01 5.80641867e+00 9.12516225e-01 2.16287926e-01 8.27082912e-02 5.80641867e+00 0.00000000e+00 0.00000000e+00

0.120000 -3.12144333e+01 7.15986907e+00 3.83752279e+01 5.93874096e+00 9.21717580e-01 2.16689123e-01 8.27214014e-02 5.93874096e+00 0.00000000e+00 0.00000000e+00

0.140000 -3.10652472e+01 7.30910512e+00 3.83752279e+01 6.07812708e+00 9.31153856e-01 2.17089268e-01 8.27349165e-02 6.07812708e+00 0.00000000e+00 0.00000000e+00

0.160000 -3.09079917e+01 7.46641318e+00 3.83752279e+01 6.22534123e+00 9.40834609e-01 2.17488469e-01 8.27488710e-02 6.22534123e+00 0.00000000e+00 0.00000000e+00

0.180000 -3.07416978e+01 7.63276107e+00 3.83752279e+01 6.38134108e+00 9.50769853e-01 2.17886838e-01 8.27633002e-02 6.38134108e+00 0.00000000e+00 0.00000000e+00

0.200000 -3.05651032e+01 7.80940674e+00 3.83752279e+01 6.54737394e+00 9.60970064e-01 2.18284488e-01 8.27782464e-02 6.54737394e+00 0.00000000e+00 0.00000000e+00

0.220000 -3.03765100e+01 7.99805395e+00 3.83752279e+01 6.72513248e+00 9.71446179e-01 2.18681539e-01 8.27937533e-02 6.72513248e+00 0.00000000e+00 0.00000000e+00

0.240000 -3.01730929e+01 8.20099732e+00 3.83752279e+01 6.91690048e+00 9.82208987e-01 2.19077879e-01 8.28099697e-02 6.91690048e+00 0.00000000e+00 0.00000000e+00

0.260000 -2.99522887e+01 8.42190884e+00 3.83752279e+01 7.12633676e+00 9.93271196e-01 2.19474132e-01 8.28267473e-02 7.12633676e+00 0.00000000e+00 0.00000000e+00

0.280000 -2.97085269e+01 8.66578542e+00 3.83752279e+01 7.35842622e+00 1.00464477e+00 2.19870171e-01 8.28442589e-02 7.35842622e+00 0.00000000e+00 0.00000000e+00

0.300000 -2.94342483e+01 8.94017654e+00 3.83752279e+01 7.62070562e+00 1.01634220e+00 2.20266144e-01 8.28625770e-02 7.62070562e+00 0.00000000e+00 0.00000000e+00

0.320000 -2.91183421e+01 9.25620586e+00 3.83752279e+01 7.92428567e+00 1.02837619e+00 2.20662215e-01 8.28817859e-02 7.92428567e+00 0.00000000e+00 0.00000000e+00

0.340000 -2.87441708e+01 9.62993305e+00 3.83752279e+01 8.28521557e+00 1.04075705e+00 2.21058370e-01 8.29020625e-02 8.28521557e+00 0.00000000e+00 0.00000000e+00

0.360000 -2.82893723e+01 1.00849974e+01 3.83752279e+01 8.72711579e+00 1.05350298e+00 2.21455330e-01 8.29233183e-02 8.72711579e+00 0.00000000e+00 0.00000000e+00

0.380000 -2.77203210e+01 1.06543164e+01 3.83752279e+01 9.28289350e+00 1.06662393e+00 2.21853151e-01 8.29457798e-02 9.28289350e+00 0.00000000e+00 0.00000000e+00

0.400000 -2.69904540e+01 1.13841915e+01 3.83752279e+01 9.99883926e+00 1.08013041e+00 2.22252188e-01 8.29696158e-02 9.99883926e+00 0.00000000e+00 0.00000000e+00

0.420000 -2.60358524e+01 1.23394147e+01 3.83752279e+01 1.09397265e+01 1.09403992e+00 2.22653430e-01 8.29948483e-02 1.09397265e+01 0.00000000e+00 0.00000000e+00

0.440000 -2.47676032e+01 1.36082742e+01 3.83752279e+01 1.21938352e+01 1.10835955e+00 2.23057685e-01 8.30217322e-02 1.21938352e+01 0.00000000e+00 0.00000000e+00

0.460000 -2.30649401e+01 1.53105633e+01 3.83752279e+01 1.38809554e+01 1.12309110e+00 2.23466297e-01 8.30505618e-02 1.38809554e+01 0.00000000e+00 0.00000000e+00

0.480000 -2.07667112e+01 1.76089088e+01 3.83752279e+01 1.61636897e+01 1.13825521e+00 2.23882556e-01 8.30813346e-02 1.61636897e+01 0.00000000e+00 0.00000000e+00

0.500000 -1.76569381e+01 2.07187388e+01 3.83752279e+01 1.92574575e+01 1.15385598e+00 2.24310968e-01 8.31143186e-02 1.92574575e+01 0.00000000e+00 0.00000000e+00

0.520000 -1.34491188e+01 2.49265535e+01 3.83752279e+01 2.34487425e+01 1.16990168e+00 2.24759638e-01 8.31497171e-02 2.34487425e+01 0.00000000e+00 0.00000000e+00

0.540000 -7.75982272e+00 3.06157750e+01 3.83752279e+01 2.91209426e+01 1.18640183e+00 2.25242813e-01 8.31877319e-02 2.91209426e+01 0.00000000e+00 0.00000000e+00

0.560000 -6.42262228e-02 3.83112621e+01 3.83752279e+01 3.67988782e+01 1.20336937e+00 2.25785994e-01 8.32285512e-02 3.67988782e+01 0.00000000e+00 0.00000000e+00

0.580000 1.03818161e+01 4.87571985e+01 3.83752279e+01 4.72266665e+01 1.22082408e+00 2.26435532e-01 8.32723402e-02 4.72266665e+01 0.00000000e+00 0.00000000e+00

0.600000 2.46728796e+01 6.30481365e+01 3.83752279e+01 6.14987428e+01 1.23879786e+00 2.27276628e-01 8.33192216e-02 6.14987428e+01 0.00000000e+00 0.00000000e+00

0.620000 4.44604042e+01 8.28355514e+01 3.83752279e+01 8.12663733e+01 1.25734210e+00 2.28466779e-01 8.33692554e-02 8.12663733e+01 0.00000000e+00 0.00000000e+00

0.640000 7.22604248e+01 1.10635493e+02 3.83752279e+01 1.09045236e+02 1.27653753e+00 2.30297022e-01 8.34224188e-02 1.09045236e+02 0.00000000e+00 0.00000000e+00

0.660000 1.11896171e+02 1.50271195e+02 3.83752279e+01 1.48657908e+02 1.29650593e+00 2.33302175e-01 8.34785614e-02 1.48657908e+02 0.00000000e+00 0.00000000e+00

0.680000 1.69119175e+02 2.07494198e+02 3.83752279e+01 2.05854783e+02 1.31742320e+00 2.38454928e-01 8.35374456e-02 2.05854783e+02 0.00000000e+00 0.00000000e+00

0.700000 2.52442267e+02 2.90816540e+02 3.83752279e+01 2.89145909e+02 1.33953500e+00 2.47497323e-01 8.35986694e-02 2.89145909e+02 0.00000000e+00 0.00000000e+00

0.720000 3.74207546e+02 4.12580045e+02 3.83752279e+01 4.10869735e+02 1.36315722e+00 2.63490863e-01 8.36617266e-02 4.10869735e+02 0.00000000e+00 0.00000000e+00

0.740000 5.51639547e+02 5.90015732e+02 3.83752279e+01 5.88251631e+02 1.38869724e+00 2.91678240e-01 8.37259308e-02 5.88251631e+02 0.00000000e+00 0.00000000e+00

0.760000 8.07719911e+02 8.46104388e+02 3.83752279e+01 8.44263177e+02 1.41664505e+00 3.40776100e-01 8.37904840e-02 8.44263177e+02 0.00000000e+00 0.00000000e+00

0.780000 1.17122404e+03 1.20958662e+03 3.83752279e+01 1.20763040e+03 1.44755614e+00 4.24812245e-01 8.38545076e-02 1.20763040e+03 0.00000000e+00 0.00000000e+00

0.800000 1.67440545e+03 1.71281858e+03 3.83752279e+01 1.71068710e+03 1.48199219e+00 5.65568677e-01 8.39170222e-02 1.71068710e+03 0.00000000e+00 0.00000000e+00

solar cell parameters deduced from calculated IV-curve:

Voc = 0.560141 Volt

Jsc = 31.97738675 mA/cm2

FF = 61.1050 %

eta = 10.9450 %

V_MPP = 0.426543 Volt

J_MPP = 25.65989630 mA/cm2

SCAPS 3.3.07 ELIS-UGent: Version scaps3307.exe, dated 27-01-2018, 10:11:02

Simulation of I-V curves

Single shot simulation # 2

Calculation started : 16-9-2021 at 14:07:09

problem definition file : c:\Program Files (x86)\Scaps3307\def\example CIGS.def

last saved: 01-09-2015 at 07:44:06

Calculation under illumination

Optical generation from internal SCAPS calculation

Illumination spectrum read from file

spectrum file :

C:\Program Files (x86)\Scaps3307\spectrum\AM1_5G 1 sun.spe last saved: 22-05-2009 at 09:59:48

Neutral density filter: ND 0.0000

Transmission of ND filter: 1.0000e+00

Power from spectrum: 100.0000 mW/cm2

Incident power on solar cell structure 100.0000 mW/cm2

Working point conditions

Temperature 300.00 K

Workpoint bias voltage 0.0000 Volt

Frequency 1.0000e+06 Hz

Voltage reference convention: voltage referred to right contact, voltage V applied to left contact

Current reference convention: current positive when entering the left contact

Power generated by the cell: = -V*I; power consumed by the cell = +V*I

v(V) jtot(mA/cm2) j_total_rec(mA/cm2) j_total_gen(mA/cm2) jbulk(mA/cm2) jifr(mA/cm2) jminor_left(mA/cm2) jminor_right(mA/cm2) j_SRH(mA/cm2) j_Radiative(mA/cm2) j_Auger(mA/cm2)

0.000000 -3.25293387e+01 5.84572600e+00 3.83752279e+01 4.76140106e+00 7.86705686e-01 2.09300154e-01 8.83190983e-02 4.76140106e+00 0.00000000e+00 0.00000000e+00

0.020000 -3.24304997e+01 5.94476490e+00 3.83752279e+01 4.85535068e+00 7.91355165e-01 2.09739833e-01 8.83192265e-02 4.85535068e+00 0.00000000e+00 0.00000000e+00

0.040000 -3.23271305e+01 6.04814619e+00 3.83752279e+01 4.95358666e+00 7.96062632e-01 2.10177542e-01 8.83193525e-02 4.95358666e+00 0.00000000e+00 0.00000000e+00

0.060000 -3.22190747e+01 6.15621327e+00 3.83752279e+01 5.05645148e+00 8.00828922e-01 2.10613399e-01 8.83194763e-02 5.05645148e+00 0.00000000e+00 0.00000000e+00

0.080000 -3.21059536e+01 6.26934537e+00 3.83752279e+01 5.16432338e+00 8.05654892e-01 2.11047503e-01 8.83195987e-02 5.16432338e+00 0.00000000e+00 0.00000000e+00

0.100000 -3.19873472e+01 6.38796374e+00 3.83752279e+01 5.27762264e+00 8.10541421e-01 2.11479959e-01 8.83197193e-02 5.27762264e+00 0.00000000e+00 0.00000000e+00

0.120000 -3.18627801e+01 6.51254434e+00 3.83752279e+01 5.39682422e+00 8.15489403e-01 2.11910875e-01 8.83198380e-02 5.39682422e+00 0.00000000e+00 0.00000000e+00

0.140000 -3.17317062e+01 6.64363568e+00 3.83752279e+01 5.52247561e+00 8.20499752e-01 2.12340365e-01 8.83199551e-02 5.52247561e+00 0.00000000e+00 0.00000000e+00

0.160000 -3.15934796e+01 6.78187792e+00 3.83752279e+01 5.65521589e+00 8.25573400e-01 2.12768553e-01 8.83200704e-02 5.65521589e+00 0.00000000e+00 0.00000000e+00

0.180000 -3.14473496e+01 6.92802626e+00 3.83752279e+01 5.79579921e+00 8.30711300e-01 2.13195565e-01 8.83201838e-02 5.79579921e+00 0.00000000e+00 0.00000000e+00

0.200000 -3.12924000e+01 7.08299272e+00 3.83752279e+01 5.94513646e+00 8.35914425e-01 2.13621539e-01 8.83202957e-02 5.94513646e+00 0.00000000e+00 0.00000000e+00

0.220000 -3.11274945e+01 7.24792237e+00 3.83752279e+01 6.10437158e+00 8.41183773e-01 2.14046620e-01 8.83204058e-02 6.10437158e+00 0.00000000e+00 0.00000000e+00

0.240000 -3.09511171e+01 7.42432208e+00 3.83752279e+01 6.27501024e+00 8.46520360e-01 2.14470965e-01 8.83205143e-02 6.27501024e+00 0.00000000e+00 0.00000000e+00

0.260000 -3.07611861e+01 7.61427743e+00 3.83752279e+01 6.45913684e+00 8.51925230e-01 2.14894744e-01 8.83206210e-02 6.45913684e+00 0.00000000e+00 0.00000000e+00

0.280000 -3.05546675e+01 7.82083142e+00 3.83752279e+01 6.65979311e+00 8.57399447e-01 2.15318142e-01 8.83207258e-02 6.65979311e+00 0.00000000e+00 0.00000000e+00

0.300000 -3.03268885e+01 8.04863520e+00 3.83752279e+01 6.88162890e+00 8.62944102e-01 2.15741371e-01 8.83208293e-02 6.88162890e+00 0.00000000e+00 0.00000000e+00

0.320000 -3.00705462e+01 8.30501319e+00 3.83752279e+01 7.13196728e+00 8.68560310e-01 2.16164670e-01 8.83209314e-02 7.13196728e+00 0.00000000e+00 0.00000000e+00

0.340000 -2.97739592e+01 8.60163530e+00 3.83752279e+01 7.42247674e+00 8.74249209e-01 2.16588328e-01 8.83210320e-02 7.42247674e+00 0.00000000e+00 0.00000000e+00

0.360000 -2.94185701e+01 8.95705449e+00 3.83752279e+01 7.77170869e+00 8.80011960e-01 2.17012706e-01 8.83211311e-02 7.77170869e+00 0.00000000e+00 0.00000000e+00

0.380000 -2.89752161e+01 9.40043305e+00 3.83752279e+01 8.20882379e+00 8.85849742e-01 2.17438287e-01 8.83212278e-02 8.20882379e+00 0.00000000e+00 0.00000000e+00

0.400000 -2.83987374e+01 9.97693510e+00 3.83752279e+01 8.77898428e+00 8.91763749e-01 2.17865751e-01 8.83213242e-02 8.77898428e+00 0.00000000e+00 0.00000000e+00

0.420000 -2.76200084e+01 1.07556721e+01 3.83752279e+01 9.55129933e+00 8.97755181e-01 2.18296128e-01 8.83214189e-02 9.55129933e+00 0.00000000e+00 0.00000000e+00

0.440000 -2.65338965e+01 1.18417831e+01 3.83752279e+01 1.06309052e+01 9.03825230e-01 2.18731079e-01 8.83215132e-02 1.06309052e+01 0.00000000e+00 0.00000000e+00

0.460000 -2.49805576e+01 1.33950996e+01 3.83752279e+01 1.21776295e+01 9.09975061e-01 2.19173442e-01 8.83216071e-02 1.21776295e+01 0.00000000e+00 0.00000000e+00

0.480000 -2.27166373e+01 1.56589707e+01 3.83752279e+01 1.44348150e+01 9.16205774e-01 2.19628300e-01 8.83216991e-02 1.44348150e+01 0.00000000e+00 0.00000000e+00

0.500000 -1.93708500e+01 1.90046692e+01 3.83752279e+01 1.77737240e+01 9.22518359e-01 2.20105070e-01 8.83217922e-02 1.77737240e+01 0.00000000e+00 0.00000000e+00

0.520000 -1.43753965e+01 2.39999810e+01 3.83752279e+01 2.27621238e+01 9.28913604e-01 2.20621660e-01 8.83218868e-02 2.27621238e+01 0.00000000e+00 0.00000000e+00

0.540000 -6.85945970e+00 3.15157196e+01 3.83752279e+01 3.02707929e+01 9.35391966e-01 2.21212702e-01 8.83219823e-02 3.02707929e+01 0.00000000e+00 0.00000000e+00

0.560000 4.51673561e+00 4.28917176e+01 3.83752279e+01 4.16394962e+01 9.41953367e-01 2.21945926e-01 8.83220818e-02 4.16394962e+01 0.00000000e+00 0.00000000e+00

0.580000 2.18203504e+01 6.01953334e+01 3.83752279e+01 5.89354596e+01 9.48596867e-01 2.22954667e-01 8.83221877e-02 5.89354596e+01 0.00000000e+00 0.00000000e+00

0.600000 4.82468666e+01 8.66219045e+01 3.83752279e+01 8.53537602e+01 9.55320103e-01 2.24501939e-01 8.83223079e-02 8.53537602e+01 0.00000000e+00 0.00000000e+00

0.620000 8.87326710e+01 1.27107663e+02 3.83752279e+01 1.25830115e+02 9.62118740e-01 2.27107048e-01 8.83224473e-02 1.25830115e+02 0.00000000e+00 0.00000000e+00

0.640000 1.50874051e+02 1.89248757e+02 3.83752279e+01 1.87959659e+02 9.68985106e-01 2.31790226e-01 8.83226186e-02 1.87959659e+02 0.00000000e+00 0.00000000e+00

0.660000 2.46252360e+02 2.84625448e+02 3.83752279e+01 2.83320678e+02 9.75906741e-01 2.40541167e-01 8.83228366e-02 2.83320678e+02 0.00000000e+00 0.00000000e+00

0.680000 3.92211205e+02 4.30575678e+02 3.83752279e+01 4.29247299e+02 9.82864361e-01 2.57191610e-01 8.83231282e-02 4.29247299e+02 0.00000000e+00 0.00000000e+00

0.700000 6.13865143e+02 6.52243122e+02 3.83752279e+01 6.50875986e+02 9.89829405e-01 2.88982987e-01 8.83235312e-02 6.50875986e+02 0.00000000e+00 0.00000000e+00

0.720000 9.45864088e+02 9.84261773e+02 3.83752279e+01 9.82827448e+02 9.96762996e-01 3.49237466e-01 8.83240962e-02 9.82827448e+02 0.00000000e+00 0.00000000e+00

0.740000 1.43230515e+03 1.47065991e+03 3.83752279e+01 1.46910636e+03 1.00361693e+00 4.61611144e-01 8.83248873e-02 1.46910636e+03 0.00000000e+00 0.00000000e+00

0.760000 2.12294230e+03 2.16113679e+03 3.83752279e+01 2.15937183e+03 1.01033848e+00 6.66295834e-01 8.83259785e-02 2.15937183e+03 0.00000000e+00 0.00000000e+00

0.780000 3.06420085e+03 3.10290396e+03 3.83752279e+01 3.10077053e+03 1.01687869e+00 1.02821836e+00 8.83274460e-02 3.10077053e+03 0.00000000e+00 0.00000000e+00

0.800000 4.29363714e+03 4.33127293e+03 3.83752279e+01 4.32851355e+03 1.02321410e+00 1.64783417e+00 8.83293271e-02 4.32851355e+03 0.00000000e+00 0.00000000e+00

solar cell parameters deduced from calculated IV-curve:

Voc = 0.552839 Volt

Jsc = 32.52933868 mA/cm2

FF = 64.9360 %

eta = 11.6778 %

V_MPP = 0.436934 Volt

J_MPP = 26.72664812 mA/cm2

SCAPS 3.3.07 ELIS-UGent: Version scaps3307.exe, dated 27-01-2018, 10:11:02

Simulation of I-V curves

Single shot simulation # 3

Calculation started : 16-9-2021 at 14:07:30

problem definition file : c:\Program Files (x86)\Scaps3307\def\example CIGS.def

last saved: 01-09-2015 at 07:44:06

Calculation under illumination

Optical generation from internal SCAPS calculation

Illumination spectrum read from file

spectrum file :

C:\Program Files (x86)\Scaps3307\spectrum\AM1_5G 1 sun.spe last saved: 22-05-2009 at 09:59:48

Neutral density filter: ND 0.0000

Transmission of ND filter: 1.0000e+00

Power from spectrum: 100.0000 mW/cm2

Incident power on solar cell structure 100.0000 mW/cm2

Working point conditions

Temperature 300.00 K

Workpoint bias voltage 0.0000 Volt

Frequency 1.0000e+06 Hz

Voltage reference convention: voltage referred to right contact, voltage V applied to left contact

Current reference convention: current positive when entering the left contact

Power generated by the cell: = -V*I; power consumed by the cell = +V*I

v(V) jtot(mA/cm2) j_total_rec(mA/cm2) j_total_gen(mA/cm2) jbulk(mA/cm2) jifr(mA/cm2) jminor_left(mA/cm2) jminor_right(mA/cm2) j_SRH(mA/cm2) j_Radiative(mA/cm2) j_Auger(mA/cm2)

0.000000 -3.29701158e+01 5.40493719e+00 3.83752279e+01 4.84569377e+00 2.30383205e-01 2.07497954e-01 1.21362269e-01 4.84569377e+00 0.00000000e+00 0.00000000e+00

0.020000 -3.28728578e+01 5.50241730e+00 3.83752279e+01 4.94243538e+00 2.30644698e-01 2.07974954e-01 1.21362269e-01 4.94243538e+00 0.00000000e+00 0.00000000e+00

0.040000 -3.27709494e+01 5.60433726e+00 3.83752279e+01 5.04361790e+00 2.30907726e-01 2.08449368e-01 1.21362269e-01 5.04361790e+00 0.00000000e+00 0.00000000e+00

0.060000 -3.26642616e+01 5.71103759e+00 3.83752279e+01 5.14958170e+00 2.31172304e-01 2.08921319e-01 1.21362269e-01 5.14958170e+00 0.00000000e+00 0.00000000e+00

0.080000 -3.25524161e+01 5.82289653e+00 3.83752279e+01 5.26070489e+00 2.31438450e-01 2.09390913e-01 1.21362269e-01 5.26070489e+00 0.00000000e+00 0.00000000e+00

0.100000 -3.24349820e+01 5.94034534e+00 3.83752279e+01 5.37741863e+00 2.31706179e-01 2.09858261e-01 1.21362269e-01 5.37741863e+00 0.00000000e+00 0.00000000e+00

0.120000 -3.23114616e+01 6.06388181e+00 3.83752279e+01 5.50022055e+00 2.31975508e-01 2.10323479e-01 1.21362270e-01 5.50022055e+00 0.00000000e+00 0.00000000e+00

0.140000 -3.21812808e+01 6.19407996e+00 3.83752279e+01 5.62968455e+00 2.32246452e-01 2.10786689e-01 1.21362270e-01 5.62968455e+00 0.00000000e+00 0.00000000e+00

0.160000 -3.20437786e+01 6.33160115e+00 3.83752279e+01 5.76647183e+00 2.32519029e-01 2.11248020e-01 1.21362269e-01 5.76647183e+00 0.00000000e+00 0.00000000e+00

0.180000 -3.18981828e+01 6.47721763e+00 3.83752279e+01 5.91135450e+00 2.32793256e-01 2.11707607e-01 1.21362270e-01 5.91135450e+00 0.00000000e+00 0.00000000e+00

0.200000 -3.17435665e+01 6.63185674e+00 3.83752279e+01 6.06525972e+00 2.33069150e-01 2.12165594e-01 1.21362270e-01 6.06525972e+00 0.00000000e+00 0.00000000e+00

0.220000 -3.15787772e+01 6.79667113e+00 3.83752279e+01 6.22934000e+00 2.33346728e-01 2.12622134e-01 1.21362270e-01 6.22934000e+00 0.00000000e+00 0.00000000e+00

0.240000 -3.14023223e+01 6.97315330e+00 3.83752279e+01 6.40508763e+00 2.33626009e-01 2.13077393e-01 1.21362271e-01 6.40508763e+00 0.00000000e+00 0.00000000e+00

0.260000 -3.12121635e+01 7.16334211e+00 3.83752279e+01 6.59454128e+00 2.33907010e-01 2.13531546e-01 1.21362271e-01 6.59454128e+00 0.00000000e+00 0.00000000e+00

0.280000 -3.10053412e+01 7.37019708e+00 3.83752279e+01 6.80066027e+00 2.34189749e-01 2.13984789e-01 1.21362271e-01 6.80066027e+00 0.00000000e+00 0.00000000e+00

0.300000 -3.07773345e+01 7.59823867e+00 3.83752279e+01 7.02796482e+00 2.34474246e-01 2.14437339e-01 1.21362271e-01 7.02796482e+00 0.00000000e+00 0.00000000e+00

0.320000 -3.05210141e+01 7.85459559e+00 3.83752279e+01 7.28358336e+00 2.34760518e-01 2.14889446e-01 1.21362271e-01 7.28358336e+00 0.00000000e+00 0.00000000e+00

0.340000 -3.02249735e+01 8.15067322e+00 3.83752279e+01 7.57892096e+00 2.35048584e-01 2.15341407e-01 1.21362271e-01 7.57892096e+00 0.00000000e+00 0.00000000e+00

0.360000 -2.98709688e+01 8.50471370e+00 3.83752279e+01 7.93221936e+00 2.35338464e-01 2.15793598e-01 1.21362271e-01 7.93221936e+00 0.00000000e+00 0.00000000e+00

0.380000 -2.94301666e+01 8.94554785e+00 3.83752279e+01 8.37230888e+00 2.35630175e-01 2.16246520e-01 1.21362272e-01 8.37230888e+00 0.00000000e+00 0.00000000e+00

0.400000 -2.88577543e+01 9.51798555e+00 3.83752279e+01 8.94399865e+00 2.35923736e-01 2.16700888e-01 1.21362273e-01 8.94399865e+00 0.00000000e+00 0.00000000e+00

0.420000 -2.80850041e+01 1.02907507e+01 3.83752279e+01 9.71601143e+00 2.36219163e-01 2.17157798e-01 1.21362274e-01 9.71601143e+00 0.00000000e+00 0.00000000e+00

0.440000 -2.70071381e+01 1.13686166e+01 3.83752279e+01 1.07931188e+01 2.36516472e-01 2.17619036e-01 1.21362274e-01 1.07931188e+01 0.00000000e+00 0.00000000e+00

0.460000 -2.54644898e+01 1.29112427e+01 3.83752279e+01 1.23349770e+01 2.36815676e-01 2.18087700e-01 1.21362276e-01 1.23349770e+01 0.00000000e+00 0.00000000e+00

0.480000 -2.32134559e+01 1.51622221e+01 3.83752279e+01 1.45851736e+01 2.37116782e-01 2.18569390e-01 1.21362278e-01 1.45851736e+01 0.00000000e+00 0.00000000e+00

0.500000 -1.98819894e+01 1.84935872e+01 3.83752279e+01 1.79157306e+01 2.37419793e-01 2.19074584e-01 1.21362283e-01 1.79157306e+01 0.00000000e+00 0.00000000e+00

0.520000 -1.49011878e+01 2.34742235e+01 3.83752279e+01 2.28955132e+01 2.37724699e-01 2.19623343e-01 1.21362288e-01 2.28955132e+01 0.00000000e+00 0.00000000e+00

0.540000 -7.39916462e+00 3.09760066e+01 3.83752279e+01 3.03963582e+01 2.38031475e-01 2.20254661e-01 1.21362296e-01 3.03963582e+01 0.00000000e+00 0.00000000e+00

0.560000 3.96481897e+00 4.23397099e+01 3.83752279e+01 4.17589624e+01 2.38340067e-01 2.21045112e-01 1.21362308e-01 4.17589624e+01 0.00000000e+00 0.00000000e+00

0.580000 2.12599397e+01 5.96347169e+01 3.83752279e+01 5.90525583e+01 2.38650380e-01 2.22145916e-01 1.21362327e-01 5.90525583e+01 0.00000000e+00 0.00000000e+00

0.600000 4.76884524e+01 8.60634933e+01 3.83752279e+01 8.54793124e+01 2.38962255e-01 2.23856216e-01 1.21362355e-01 8.54793124e+01 0.00000000e+00 0.00000000e+00

0.620000 8.82086871e+01 1.26583688e+02 3.83752279e+01 1.25996282e+02 2.39275450e-01 2.26768037e-01 1.21362399e-01 1.25996282e+02 0.00000000e+00 0.00000000e+00

0.640000 1.50471337e+02 1.88846069e+02 3.83752279e+01 1.88253070e+02 2.39589531e-01 2.32047579e-01 1.21362466e-01 1.88253070e+02 0.00000000e+00 0.00000000e+00

0.660000 2.46184572e+02 2.84557749e+02 3.83752279e+01 2.83954507e+02 2.39903870e-01 2.41976243e-01 1.21362569e-01 2.83954507e+02 0.00000000e+00 0.00000000e+00

0.680000 3.92971832e+02 4.31336591e+02 3.83752279e+01 4.30714045e+02 2.40217512e-01 2.60965374e-01 1.21362727e-01 4.30714045e+02 0.00000000e+00 0.00000000e+00

0.700000 6.16540445e+02 6.54918408e+02 3.83752279e+01 6.54259118e+02 2.40529037e-01 2.97397871e-01 1.21362968e-01 6.54259118e+02 0.00000000e+00 0.00000000e+00

0.720000 9.52718716e+02 9.91116743e+02 3.83752279e+01 9.90387729e+02 2.40836523e-01 3.66813773e-01 1.21363331e-01 9.90387729e+02 0.00000000e+00 0.00000000e+00

0.740000 1.44774916e+03 1.48610261e+03 3.83752279e+01 1.48524302e+03 2.41137540e-01 4.97087432e-01 1.21363864e-01 1.48524302e+03 0.00000000e+00 0.00000000e+00

0.760000 2.15480864e+03 2.19298056e+03 3.83752279e+01 2.19188158e+03 2.41429369e-01 7.36184346e-01 1.21364624e-01 2.19188158e+03 0.00000000e+00 0.00000000e+00

0.780000 3.12465622e+03 3.16344690e+03 3.83752279e+01 3.16192110e+03 2.41709350e-01 1.16272353e+00 1.21365673e-01 3.16192110e+03 0.00000000e+00 0.00000000e+00

0.800000 4.39877988e+03 4.43756943e+03 3.83752279e+01 4.43530589e+03 2.41975951e-01 1.90019705e+00 1.21367045e-01 4.43530589e+03 0.00000000e+00 0.00000000e+00

solar cell parameters deduced from calculated IV-curve:

Voc = 0.553752 Volt

Jsc = 32.97011577 mA/cm2

FF = 65.0936 %

eta = 11.8843 %

V_MPP = 0.438066 Volt

J_MPP = 27.12901534 mA/cm2

# **In_2_S_3_ nanoparticle (Eg = 2.32 eV)**

SCAPS 3.3.07 ELIS-UGent: Version scaps3307.exe, dated 27-01-2018, 10:11:02

Simulation of I-V curves

Single shot simulation # 1

Calculation started : 16-9-2021 at 15:02:57

problem definition file : c:\Program Files (x86)\Scaps3307\def\example CIGS.def

last saved: 01-09-2015 at 07:44:06

Calculation under illumination

Optical generation from internal SCAPS calculation

Illumination spectrum read from file

spectrum file :

C:\Program Files (x86)\Scaps3307\spectrum\AM1_5G 1 sun.spe last saved: 22-05-2009 at 09:59:48

Neutral density filter: ND 0.0000

Transmission of ND filter: 1.0000e+00

Power from spectrum: 100.0000 mW/cm2

Incident power on solar cell structure 100.0000 mW/cm2

Working point conditions

Temperature 300.00 K

Workpoint bias voltage 0.0000 Volt

Frequency 1.0000e+06 Hz

Voltage reference convention: voltage referred to right contact, voltage V applied to left contact

Current reference convention: current positive when entering the left contact

Power generated by the cell: = -V*I; power consumed by the cell = +V*I

v(V) jtot(mA/cm2) j_total_rec(mA/cm2) j_total_gen(mA/cm2) jbulk(mA/cm2) jifr(mA/cm2) jminor_left(mA/cm2) jminor_right(mA/cm2) j_SRH(mA/cm2) j_Radiative(mA/cm2) j_Auger(mA/cm2)

0.000000 -2.99264113e+01 8.44426382e+00 3.83705264e+01 6.45271738e+00 1.63050868e+00 2.18000186e-01 1.43037579e-01 6.45271738e+00 0.00000000e+00 0.00000000e+00

0.020000 -2.97471614e+01 8.62218581e+00 3.83705264e+01 6.61519861e+00 1.64556074e+00 2.18379089e-01 1.43047370e-01 6.61519861e+00 0.00000000e+00 0.00000000e+00

0.040000 -2.95580753e+01 8.81151617e+00 3.83705264e+01 6.78872605e+00 1.66097552e+00 2.18757246e-01 1.43057352e-01 6.78872605e+00 0.00000000e+00 0.00000000e+00

0.060000 -2.93554333e+01 9.01437916e+00 3.83705264e+01 6.97541120e+00 1.67676576e+00 2.19134627e-01 1.43067567e-01 6.97541120e+00 0.00000000e+00 0.00000000e+00

0.080000 -2.91361413e+01 9.23388868e+00 3.83705264e+01 7.17834815e+00 1.69295109e+00 2.19511435e-01 1.43078010e-01 7.17834815e+00 0.00000000e+00 0.00000000e+00

0.100000 -2.88952791e+01 9.47438335e+00 3.83705264e+01 7.40185550e+00 1.70955130e+00 2.19887859e-01 1.43088690e-01 7.40185550e+00 0.00000000e+00 0.00000000e+00

0.120000 -2.86282409e+01 9.74209294e+00 3.83705264e+01 7.65213167e+00 1.72659743e+00 2.20264266e-01 1.43099567e-01 7.65213167e+00 0.00000000e+00 0.00000000e+00

0.140000 -2.83251775e+01 1.00452704e+01 3.83705264e+01 7.93740805e+00 1.74411085e+00 2.20640850e-01 1.43110666e-01 7.93740805e+00 0.00000000e+00 0.00000000e+00

0.160000 -2.79753528e+01 1.03951578e+01 3.83705264e+01 8.26889270e+00 1.76212514e+00 2.21017993e-01 1.43121968e-01 8.26889270e+00 0.00000000e+00 0.00000000e+00

0.180000 -2.75643147e+01 1.08062669e+01 3.83705264e+01 8.66106120e+00 1.78067611e+00 2.21396103e-01 1.43133462e-01 8.66106120e+00 0.00000000e+00 0.00000000e+00

0.200000 -2.70739963e+01 1.12966303e+01 3.83705264e+01 9.13190479e+00 1.79980468e+00 2.21775658e-01 1.43145137e-01 9.13190479e+00 0.00000000e+00 0.00000000e+00

0.220000 -2.64832656e+01 1.18873703e+01 3.83705264e+01 9.70249839e+00 1.81955773e+00 2.22157227e-01 1.43156981e-01 9.70249839e+00 0.00000000e+00 0.00000000e+00

0.240000 -2.57696659e+01 1.26009659e+01 3.83705264e+01 1.03952664e+01 1.83998907e+00 2.22541479e-01 1.43168980e-01 1.03952664e+01 0.00000000e+00 0.00000000e+00

0.260000 -2.49134246e+01 1.34571933e+01 3.83705264e+01 1.12299226e+01 1.86116036e+00 2.22929203e-01 1.43181120e-01 1.12299226e+01 0.00000000e+00 0.00000000e+00

0.280000 -2.39049706e+01 1.44655935e+01 3.83705264e+01 1.22159370e+01 1.88314174e+00 2.23321312e-01 1.43193389e-01 1.22159370e+01 0.00000000e+00 0.00000000e+00

0.300000 -2.27560707e+01 1.56144194e+01 3.83705264e+01 1.33414829e+01 1.90601183e+00 2.23718850e-01 1.43205770e-01 1.33414829e+01 0.00000000e+00 0.00000000e+00

0.320000 -2.15103208e+01 1.68601905e+01 3.83705264e+01 1.45629920e+01 1.92985719e+00 2.24123043e-01 1.43218251e-01 1.45629920e+01 0.00000000e+00 0.00000000e+00

0.340000 -2.02412045e+01 1.81292795e+01 3.83705264e+01 1.58067401e+01 1.95477312e+00 2.24535464e-01 1.43230816e-01 1.58067401e+01 0.00000000e+00 0.00000000e+00

0.360000 -1.90271856e+01 1.93432771e+01 3.83705264e+01 1.69942047e+01 1.98087050e+00 2.24958441e-01 1.43243454e-01 1.69942047e+01 0.00000000e+00 0.00000000e+00

0.380000 -1.79132154e+01 2.04572321e+01 3.83705264e+01 1.80802890e+01 2.00829134e+00 2.25395616e-01 1.43256154e-01 1.80802890e+01 0.00000000e+00 0.00000000e+00

0.400000 -1.68942583e+01 2.14761778e+01 3.83705264e+01 1.90698273e+01 2.03722915e+00 2.25852472e-01 1.43268905e-01 1.90698273e+01 0.00000000e+00 0.00000000e+00

0.420000 -1.59322466e+01 2.24381610e+01 3.83705264e+01 2.00005928e+01 2.06794984e+00 2.26336680e-01 1.43281702e-01 2.00005928e+01 0.00000000e+00 0.00000000e+00

0.440000 -1.49820425e+01 2.33883640e+01 3.83705264e+01 2.09173912e+01 2.10081992e+00 2.26858350e-01 1.43294539e-01 2.09173912e+01 0.00000000e+00 0.00000000e+00

0.460000 -1.40062051e+01 2.43641824e+01 3.83705264e+01 2.18570901e+01 2.13635480e+00 2.27430052e-01 1.43307413e-01 2.18570901e+01 0.00000000e+00 0.00000000e+00

0.480000 -1.29795375e+01 2.53908338e+01 3.83705264e+01 2.28441461e+01 2.17530118e+00 2.28066241e-01 1.43320321e-01 2.28441461e+01 0.00000000e+00 0.00000000e+00

0.500000 -1.18893971e+01 2.64809648e+01 3.83705264e+01 2.38900785e+01 2.21877155e+00 2.28781579e-01 1.43333257e-01 2.38900785e+01 0.00000000e+00 0.00000000e+00

0.520000 -1.07356392e+01 2.76347196e+01 3.83705264e+01 2.49933261e+01 2.26845954e+00 2.29587797e-01 1.43346215e-01 2.49933261e+01 0.00000000e+00 0.00000000e+00

0.540000 -9.53019238e+00 2.88401634e+01 3.83705264e+01 2.61393346e+01 2.32698028e+00 2.30489374e-01 1.43359183e-01 2.61393346e+01 0.00000000e+00 0.00000000e+00

0.560000 -8.29462827e+00 3.00757247e+01 3.83705264e+01 2.73024701e+01 2.39840309e+00 2.31479359e-01 1.43372139e-01 2.73024701e+01 0.00000000e+00 0.00000000e+00

0.580000 -7.05496965e+00 3.13163896e+01 3.83705264e+01 2.84513896e+01 2.48907737e+00 2.32537683e-01 1.43385045e-01 2.84513896e+01 0.00000000e+00 0.00000000e+00

0.600000 -5.82752073e+00 3.25436661e+01 3.83705264e+01 2.95577427e+01 2.60889173e+00 2.33633827e-01 1.43397861e-01 2.95577427e+01 0.00000000e+00 0.00000000e+00

0.620000 -4.61346006e+00 3.37576162e+01 3.83705264e+01 3.06062735e+01 2.77319783e+00 2.34734397e-01 1.43410519e-01 3.06062735e+01 0.00000000e+00 0.00000000e+00

0.640000 -3.38336680e+00 3.49875981e+01 3.83705264e+01 3.16026732e+01 3.00568942e+00 2.35812513e-01 1.43422939e-01 3.16026732e+01 0.00000000e+00 0.00000000e+00

0.660000 -2.07003588e+00 3.63007984e+01 3.83705264e+01 3.25777956e+01 3.34271171e+00 2.36856087e-01 1.43435028e-01 3.25777956e+01 0.00000000e+00 0.00000000e+00

0.680000 -5.60718513e-01 3.78099734e+01 3.83705264e+01 3.35889903e+01 3.83966365e+00 2.37872737e-01 1.43446691e-01 3.35889903e+01 0.00000000e+00 0.00000000e+00

0.700000 1.31345363e+00 3.96840354e+01 3.83705264e+01 3.47212259e+01 4.58045991e+00 2.38891813e-01 1.43457834e-01 3.47212259e+01 0.00000000e+00 0.00000000e+00

0.720000 3.79500645e+00 4.21654638e+01 3.83705264e+01 3.60906583e+01 5.69137162e+00 2.39965551e-01 1.43468376e-01 3.60906583e+01 0.00000000e+00 0.00000000e+00

0.740000 7.22752567e+00 4.55978681e+01 3.83705264e+01 3.78523322e+01 7.36088617e+00 2.41171492e-01 1.43478251e-01 3.78523322e+01 0.00000000e+00 0.00000000e+00

0.760000 1.20958562e+01 5.04661470e+01 3.83705264e+01 4.02126532e+01 9.86738887e+00 2.42617447e-01 1.43487422e-01 4.02126532e+01 0.00000000e+00 0.00000000e+00

0.780000 1.90804558e+01 5.74507378e+01 3.83705264e+01 4.34465294e+01 1.36162630e+01 2.44449525e-01 1.43495874e-01 4.34465294e+01 0.00000000e+00 0.00000000e+00

0.800000 2.91255698e+01 6.74960438e+01 3.83705264e+01 4.79186018e+01 1.91870752e+01 2.46863253e-01 1.43503621e-01 4.79186018e+01 0.00000000e+00 0.00000000e+00

solar cell parameters deduced from calculated IV-curve:

Voc = 0.686408 Volt

Jsc = 29.92641129 mA/cm2

FF = 33.5414 %

eta = 6.8900 %

V_MPP = 0.329590 Volt

J_MPP = 20.90473213 mA/cm2

SCAPS 3.3.07 ELIS-UGent: Version scaps3307.exe, dated 27-01-2018, 10:11:02

Simulation of I-V curves

Single shot simulation # 2

Calculation started : 16-9-2021 at 15:03:23

problem definition file : c:\Program Files (x86)\Scaps3307\def\example CIGS.def

last saved: 01-09-2015 at 07:44:06

Calculation under illumination

Optical generation from internal SCAPS calculation

Illumination spectrum read from file

spectrum file :

C:\Program Files (x86)\Scaps3307\spectrum\AM1_5G 1 sun.spe last saved: 22-05-2009 at 09:59:48

Neutral density filter: ND 0.0000

Transmission of ND filter: 1.0000e+00

Power from spectrum: 100.0000 mW/cm2

Incident power on solar cell structure 100.0000 mW/cm2

Working point conditions

Temperature 300.00 K

Workpoint bias voltage 0.0000 Volt

Frequency 1.0000e+06 Hz

Voltage reference convention: voltage referred to right contact, voltage V applied to left contact

Current reference convention: current positive when entering the left contact

Power generated by the cell: = -V*I; power consumed by the cell = +V*I

v(V) jtot(mA/cm2) j_total_rec(mA/cm2) j_total_gen(mA/cm2) jbulk(mA/cm2) jifr(mA/cm2) jminor_left(mA/cm2) jminor_right(mA/cm2) j_SRH(mA/cm2) j_Radiative(mA/cm2) j_Auger(mA/cm2)

0.000000 -3.10345343e+01 7.33582158e+00 3.83705264e+01 5.44220630e+00 1.53314259e+00 2.14987359e-01 1.45485335e-01 5.44220630e+00 0.00000000e+00 0.00000000e+00

0.020000 -3.09058099e+01 7.46464102e+00 3.83705264e+01 5.55802166e+00 1.54573846e+00 2.15395482e-01 1.45485418e-01 5.55802166e+00 0.00000000e+00 0.00000000e+00

0.040000 -3.07709708e+01 7.59948470e+00 3.83705264e+01 5.67952941e+00 1.55866704e+00 2.15802751e-01 1.45485499e-01 5.67952941e+00 0.00000000e+00 0.00000000e+00

0.060000 -3.06295799e+01 7.74087854e+00 3.83705264e+01 5.80723864e+00 1.57194497e+00 2.16209344e-01 1.45485580e-01 5.80723864e+00 0.00000000e+00 0.00000000e+00

0.080000 -3.04810283e+01 7.88943021e+00 3.83705264e+01 5.94173883e+00 1.58559029e+00 2.16615428e-01 1.45485662e-01 5.94173883e+00 0.00000000e+00 0.00000000e+00

0.100000 -3.03246064e+01 8.04586037e+00 3.83705264e+01 6.08373092e+00 1.59962253e+00 2.17021179e-01 1.45485743e-01 6.08373092e+00 0.00000000e+00 0.00000000e+00

0.120000 -3.01594194e+01 8.21104715e+00 3.83705264e+01 6.23407164e+00 1.61406290e+00 2.17426792e-01 1.45485824e-01 6.23407164e+00 0.00000000e+00 0.00000000e+00

0.140000 -2.99843832e+01 8.38609633e+00 3.83705264e+01 6.39384349e+00 1.62893446e+00 2.17832472e-01 1.45485905e-01 6.39384349e+00 0.00000000e+00 0.00000000e+00

0.160000 -2.97980166e+01 8.57245606e+00 3.83705264e+01 6.56446919e+00 1.64426244e+00 2.18238446e-01 1.45485986e-01 6.56446919e+00 0.00000000e+00 0.00000000e+00

0.180000 -2.95983815e+01 8.77209871e+00 3.83705264e+01 6.74789324e+00 1.66007445e+00 2.18644960e-01 1.45486067e-01 6.74789324e+00 0.00000000e+00 0.00000000e+00

0.200000 -2.93826836e+01 8.98780440e+00 3.83705264e+01 6.94686512e+00 1.67640085e+00 2.19052288e-01 1.45486148e-01 6.94686512e+00 0.00000000e+00 0.00000000e+00

0.220000 -2.91469062e+01 9.22358814e+00 3.83705264e+01 7.16536601e+00 1.69327516e+00 2.19460733e-01 1.45486230e-01 7.16536601e+00 0.00000000e+00 0.00000000e+00

0.240000 -2.88851734e+01 9.48531289e+00 3.83705264e+01 7.40922136e+00 1.71073458e+00 2.19870638e-01 1.45486311e-01 7.40922136e+00 0.00000000e+00 0.00000000e+00

0.260000 -2.85889716e+01 9.78151922e+00 3.83705264e+01 7.68692992e+00 1.72882051e+00 2.20282394e-01 1.45486394e-01 7.68692992e+00 0.00000000e+00 0.00000000e+00

0.280000 -2.82459981e+01 1.01244714e+01 3.83705264e+01 8.01070911e+00 1.74757936e+00 2.20696457e-01 1.45486476e-01 8.01070911e+00 0.00000000e+00 0.00000000e+00

0.300000 -2.78391267e+01 1.05313618e+01 3.83705264e+01 8.39769849e+00 1.76706340e+00 2.21113362e-01 1.45486560e-01 8.39769849e+00 0.00000000e+00 0.00000000e+00

0.320000 -2.73449542e+01 1.10255044e+01 3.83705264e+01 8.87115216e+00 1.78733187e+00 2.21533752e-01 1.45486645e-01 8.87115216e+00 0.00000000e+00 0.00000000e+00

0.340000 -2.67333544e+01 1.16371070e+01 3.83705264e+01 9.46120944e+00 1.80845239e+00 2.21958407e-01 1.45486732e-01 9.46120944e+00 0.00000000e+00 0.00000000e+00

0.360000 -2.59677481e+01 1.24026980e+01 3.83705264e+01 1.02043203e+01 1.83050260e+00 2.22388290e-01 1.45486821e-01 1.02043203e+01 0.00000000e+00 0.00000000e+00

0.380000 -2.50091035e+01 1.33613530e+01 3.83705264e+01 1.11394692e+01 1.85357235e+00 2.22824590e-01 1.45486912e-01 1.11394692e+01 0.00000000e+00 0.00000000e+00

0.400000 -2.38257269e+01 1.45446775e+01 3.83705264e+01 1.22981555e+01 1.87776624e+00 2.23268775e-01 1.45487007e-01 1.22981555e+01 0.00000000e+00 0.00000000e+00

0.420000 -2.24128012e+01 1.59576275e+01 3.83705264e+01 1.36852109e+01 1.90320686e+00 2.23722645e-01 1.45487105e-01 1.36852109e+01 0.00000000e+00 0.00000000e+00

0.440000 -2.08166837e+01 1.75537129e+01 3.83705264e+01 1.52539982e+01 1.93003906e+00 2.24188450e-01 1.45487206e-01 1.52539982e+01 0.00000000e+00 0.00000000e+00

0.460000 -1.91434815e+01 1.92269013e+01 3.83705264e+01 1.68983073e+01 1.95843736e+00 2.24669282e-01 1.45487308e-01 1.68983073e+01 0.00000000e+00 0.00000000e+00

0.480000 -1.75155656e+01 2.08548070e+01 3.83705264e+01 1.84955285e+01 1.98862102e+00 2.25170083e-01 1.45487412e-01 1.84955285e+01 0.00000000e+00 0.00000000e+00

0.500000 -1.59932084e+01 2.23771550e+01 3.83705264e+01 1.99850871e+01 2.02088112e+00 2.25699279e-01 1.45487516e-01 1.99850871e+01 0.00000000e+00 0.00000000e+00

0.520000 -1.45449269e+01 2.38254515e+01 3.83705264e+01 2.13980714e+01 2.05562189e+00 2.26270587e-01 1.45487621e-01 2.13980714e+01 0.00000000e+00 0.00000000e+00

0.540000 -1.30878731e+01 2.52825104e+01 3.83705264e+01 2.28166919e+01 2.09342602e+00 2.26904810e-01 1.45487728e-01 2.28166919e+01 0.00000000e+00 0.00000000e+00

0.560000 -1.15345151e+01 2.68358703e+01 3.83705264e+01 2.43275799e+01 2.13517089e+00 2.27631613e-01 1.45487839e-01 2.43275799e+01 0.00000000e+00 0.00000000e+00

0.580000 -9.81247812e+00 2.85578974e+01 3.83705264e+01 2.60016766e+01 2.18224261e+00 2.28490298e-01 1.45487956e-01 2.60016766e+01 0.00000000e+00 0.00000000e+00

0.600000 -7.86939798e+00 3.05009734e+01 3.83705264e+01 2.78890305e+01 2.23692757e+00 2.29527327e-01 1.45488078e-01 2.78890305e+01 0.00000000e+00 0.00000000e+00

0.620000 -5.67543666e+00 3.26949296e+01 3.83705264e+01 3.00155293e+01 2.30312400e+00 2.30788122e-01 1.45488209e-01 3.00155293e+01 0.00000000e+00 0.00000000e+00

0.640000 -3.22385999e+00 3.51465006e+01 3.83705264e+01 3.23810870e+01 2.38762209e+00 2.32303206e-01 1.45488348e-01 3.23810870e+01 0.00000000e+00 0.00000000e+00

0.660000 -5.21137759e-01 3.78492172e+01 3.83705264e+01 3.49672816e+01 2.50237202e+00 2.34075029e-01 1.45488496e-01 3.49672816e+01 0.00000000e+00 0.00000000e+00

0.680000 2.44106471e+00 4.08114141e+01 3.83705264e+01 3.77614318e+01 2.66841631e+00 2.36077384e-01 1.45488654e-01 3.77614318e+01 0.00000000e+00 0.00000000e+00

0.700000 5.72865942e+00 4.40989923e+01 3.83705264e+01 4.07926996e+01 2.92252880e+00 2.38275066e-01 1.45488824e-01 4.07926996e+01 0.00000000e+00 0.00000000e+00

0.720000 9.50877789e+00 4.78790489e+01 3.83705264e+01 4.41648200e+01 3.32808320e+00 2.40656761e-01 1.45489006e-01 4.41648200e+01 0.00000000e+00 0.00000000e+00

0.740000 1.40843820e+01 5.24545788e+01 3.83705264e+01 4.80735787e+01 3.99224632e+00 2.43264495e-01 1.45489204e-01 4.80735787e+01 0.00000000e+00 0.00000000e+00

0.760000 1.99251890e+01 5.82953422e+01 3.83705264e+01 5.28114932e+01 5.09215037e+00 2.46209209e-01 1.45489423e-01 5.28114932e+01 0.00000000e+00 0.00000000e+00

0.780000 2.77085751e+01 6.60789121e+01 3.83705264e+01 5.87707613e+01 6.91298617e+00 2.49674962e-01 1.45489667e-01 5.87707613e+01 0.00000000e+00 0.00000000e+00

0.800000 3.83819783e+01 7.67523108e+01 3.83705264e+01 6.64524257e+01 9.90047401e+00 2.53921174e-01 1.45489944e-01 6.64524257e+01 0.00000000e+00 0.00000000e+00

solar cell parameters deduced from calculated IV-curve:

Voc = 0.663649 Volt

Jsc = 31.03453427 mA/cm2

FF = 46.3001 %

eta = 9.5360 %

V_MPP = 0.394238 Volt

J_MPP = 24.18841305 mA/cm2

SCAPS 3.3.07 ELIS-UGent: Version scaps3307.exe, dated 27-01-2018, 10:11:02

Simulation of I-V curves

Single shot simulation # 3

Calculation started : 16-9-2021 at 15:03:44

problem definition file : c:\Program Files (x86)\Scaps3307\def\example CIGS.def

last saved: 01-09-2015 at 07:44:06

Calculation under illumination

Optical generation from internal SCAPS calculation

Illumination spectrum read from file

spectrum file :

C:\Program Files (x86)\Scaps3307\spectrum\AM1_5G 1 sun.spe last saved: 22-05-2009 at 09:59:48

Neutral density filter: ND 0.0000

Transmission of ND filter: 1.0000e+00

Power from spectrum: 100.0000 mW/cm2

Incident power on solar cell structure 100.0000 mW/cm2

Working point conditions

Temperature 300.00 K

Workpoint bias voltage 0.0000 Volt

Frequency 1.0000e+06 Hz

Voltage reference convention: voltage referred to right contact, voltage V applied to left contact

Current reference convention: current positive when entering the left contact

Power generated by the cell: = -V*I; power consumed by the cell = +V*I

v(V) jtot(mA/cm2) j_total_rec(mA/cm2) j_total_gen(mA/cm2) jbulk(mA/cm2) jifr(mA/cm2) jminor_left(mA/cm2) jminor_right(mA/cm2) j_SRH(mA/cm2) j_Radiative(mA/cm2) j_Auger(mA/cm2)

0.000000 -3.17383538e+01 6.63199524e+00 3.83705264e+01 4.83102019e+00 1.43226687e+00 2.11514233e-01 1.57193945e-01 4.83102019e+00 0.00000000e+00 0.00000000e+00

0.020000 -3.16329883e+01 6.73747637e+00 3.83705264e+01 4.92536786e+00 1.44296248e+00 2.11952090e-01 1.57193945e-01 4.92536786e+00 0.00000000e+00 0.00000000e+00

0.040000 -3.15230514e+01 6.84741729e+00 3.83705264e+01 5.02392630e+00 1.45390903e+00 2.12388011e-01 1.57193945e-01 5.02392630e+00 0.00000000e+00 0.00000000e+00

0.060000 -3.14083146e+01 6.96215852e+00 3.83705264e+01 5.12702505e+00 1.46511738e+00 2.12822137e-01 1.57193945e-01 5.12702505e+00 0.00000000e+00 0.00000000e+00

0.080000 -3.12884029e+01 7.08207502e+00 3.83705264e+01 5.23502733e+00 1.47659916e+00 2.13254590e-01 1.57193945e-01 5.23502733e+00 0.00000000e+00 0.00000000e+00

0.100000 -3.11628956e+01 7.20758742e+00 3.83705264e+01 5.34834119e+00 1.48836679e+00 2.13685499e-01 1.57193945e-01 5.34834119e+00 0.00000000e+00 0.00000000e+00

0.120000 -3.10313128e+01 7.33917583e+00 3.83705264e+01 5.46743333e+00 1.50043356e+00 2.14115002e-01 1.57193945e-01 5.46743333e+00 0.00000000e+00 0.00000000e+00

0.140000 -3.08931029e+01 7.47739187e+00 3.83705264e+01 5.59284091e+00 1.51281377e+00 2.14543241e-01 1.57193945e-01 5.59284091e+00 0.00000000e+00 0.00000000e+00

0.160000 -3.07476267e+01 7.62287476e+00 3.83705264e+01 5.72518766e+00 1.52552279e+00 2.14970371e-01 1.57193945e-01 5.72518766e+00 0.00000000e+00 0.00000000e+00

0.180000 -3.05941246e+01 7.77638417e+00 3.83705264e+01 5.86521649e+00 1.53857718e+00 2.15396556e-01 1.57193945e-01 5.86521649e+00 0.00000000e+00 0.00000000e+00

0.200000 -3.04316630e+01 7.93885385e+00 3.83705264e+01 6.01384309e+00 1.55199485e+00 2.15821969e-01 1.57193945e-01 6.01384309e+00 0.00000000e+00 0.00000000e+00

0.220000 -3.02590500e+01 8.11147566e+00 3.83705264e+01 6.17223977e+00 1.56579515e+00 2.16246798e-01 1.57193945e-01 6.17223977e+00 0.00000000e+00 0.00000000e+00

0.240000 -3.00746897e+01 8.29584565e+00 3.83705264e+01 6.34198131e+00 1.57999915e+00 2.16671247e-01 1.57193945e-01 6.34198131e+00 0.00000000e+00 0.00000000e+00

0.260000 -2.98763272e+01 8.49421875e+00 3.83705264e+01 6.52529954e+00 1.59462973e+00 2.17095536e-01 1.57193946e-01 6.52529954e+00 0.00000000e+00 0.00000000e+00

0.280000 -2.96606209e+01 8.70993634e+00 3.83705264e+01 6.72551060e+00 1.60971189e+00 2.17519910e-01 1.57193946e-01 6.72551060e+00 0.00000000e+00 0.00000000e+00

0.300000 -2.94224379e+01 8.94813081e+00 3.83705264e+01 6.94771921e+00 1.62527301e+00 2.17944643e-01 1.57193946e-01 6.94771921e+00 0.00000000e+00 0.00000000e+00

0.320000 -2.91537343e+01 9.21684595e+00 3.83705264e+01 7.19993880e+00 1.64134315e+00 2.18370049e-01 1.57193946e-01 7.19993880e+00 0.00000000e+00 0.00000000e+00

0.340000 -2.88418461e+01 9.52874461e+00 3.83705264e+01 7.49479870e+00 1.65795546e+00 2.18796494e-01 1.57193946e-01 7.49479870e+00 0.00000000e+00 0.00000000e+00

0.360000 -2.84669759e+01 9.90362327e+00 3.83705264e+01 7.85205826e+00 1.67514663e+00 2.19224429e-01 1.57193946e-01 7.85205826e+00 0.00000000e+00 0.00000000e+00

0.380000 -2.79985588e+01 1.03720458e+01 3.83705264e+01 8.30224003e+00 1.69295740e+00 2.19654421e-01 1.57193947e-01 8.30224003e+00 0.00000000e+00 0.00000000e+00

0.400000 -2.73900001e+01 1.09806056e+01 3.83705264e+01 8.89189116e+00 1.71143324e+00 2.20087227e-01 1.57193947e-01 8.89189116e+00 0.00000000e+00 0.00000000e+00

0.420000 -2.65709310e+01 1.17996700e+01 3.83705264e+01 9.69132701e+00 1.73062511e+00 2.20523910e-01 1.57193948e-01 9.69132701e+00 0.00000000e+00 0.00000000e+00

0.440000 -2.54356192e+01 1.29349696e+01 3.83705264e+01 1.08062191e+01 1.75059042e+00 2.20966054e-01 1.57193948e-01 1.08062191e+01 0.00000000e+00 0.00000000e+00

0.460000 -2.38255871e+01 1.45449801e+01 3.83705264e+01 1.23949757e+01 1.77139423e+00 2.21416158e-01 1.57193949e-01 1.23949757e+01 0.00000000e+00 0.00000000e+00

0.480000 -2.15037580e+01 1.68667753e+01 3.83705264e+01 1.46945924e+01 1.79311061e+00 2.21878363e-01 1.57193950e-01 1.46945924e+01 0.00000000e+00 0.00000000e+00

0.500000 -1.81165205e+01 2.02539640e+01 3.83705264e+01 1.80585856e+01 1.81582457e+00 2.22359821e-01 1.57193952e-01 1.80585856e+01 0.00000000e+00 0.00000000e+00

0.520000 -1.31395255e+01 2.52308959e+01 3.83705264e+01 2.30111944e+01 1.83963430e+00 2.22873226e-01 1.57193954e-01 2.30111944e+01 0.00000000e+00 0.00000000e+00

0.540000 -5.80354119e+00 3.25668141e+01 3.83705264e+01 3.03215245e+01 1.86465423e+00 2.23441486e-01 1.57193958e-01 3.03215245e+01 0.00000000e+00 0.00000000e+00

0.560000 4.99969386e+00 4.33700297e+01 3.83705264e+01 4.10977103e+01 1.89101931e+00 2.24106136e-01 1.57193963e-01 4.10977103e+01 0.00000000e+00 0.00000000e+00

0.580000 2.08198220e+01 5.91903029e+01 3.83705264e+01 5.68892758e+01 1.91889116e+00 2.24941993e-01 1.57193971e-01 5.68892758e+01 0.00000000e+00 0.00000000e+00

0.600000 4.37185820e+01 8.20888963e+01 3.83705264e+01 7.97571536e+01 1.94846751e+00 2.26081210e-01 1.57193982e-01 7.97571536e+01 0.00000000e+00 0.00000000e+00

0.620000 7.62090039e+01 1.14579289e+02 3.83705264e+01 1.12214345e+02 1.97999982e+00 2.27750235e-01 1.57193999e-01 1.12214345e+02 0.00000000e+00 0.00000000e+00

0.640000 1.20890430e+02 1.59260635e+02 3.83705264e+01 1.56859299e+02 2.01382204e+00 2.30319768e-01 1.57194021e-01 1.56859299e+02 0.00000000e+00 0.00000000e+00

0.660000 1.79572260e+02 2.17942297e+02 3.83705264e+01 2.15500328e+02 2.05040740e+00 2.34367891e-01 1.57194050e-01 2.15500328e+02 0.00000000e+00 0.00000000e+00

0.680000 2.52002666e+02 2.90372383e+02 3.83705264e+01 2.87883946e+02 2.09047342e+00 2.40769381e-01 1.57194087e-01 2.87883946e+02 0.00000000e+00 0.00000000e+00

0.700000 3.35307672e+02 3.73676855e+02 3.83705264e+01 3.71133621e+02 2.13516941e+00 2.50869780e-01 1.57194128e-01 3.71133621e+02 0.00000000e+00 0.00000000e+00

0.720000 4.25894558e+02 4.64262944e+02 3.83705264e+01 4.61652553e+02 2.18640490e+00 2.66791486e-01 1.57194175e-01 4.61652553e+02 0.00000000e+00 0.00000000e+00

0.740000 5.22862088e+02 5.61229503e+02 3.83705264e+01 5.58533185e+02 2.24745428e+00 2.91669658e-01 1.57194223e-01 5.58533185e+02 0.00000000e+00 0.00000000e+00

0.760000 6.28660808e+02 6.67031844e+02 3.83705264e+01 6.64221132e+02 2.32413176e+00 3.29386725e-01 1.57194275e-01 6.64221132e+02 0.00000000e+00 0.00000000e+00

0.780000 7.46606231e+02 7.84977373e+02 3.83705264e+01 7.82009391e+02 2.42712149e+00 3.83666471e-01 1.57194332e-01 7.82009391e+02 0.00000000e+00 0.00000000e+00

0.800000 8.78774840e+02 9.17145960e+02 3.83705264e+01 9.13955493e+02 2.57630366e+00 4.56968365e-01 1.57194400e-01 9.13955493e+02 0.00000000e+00 0.00000000e+00

solar cell parameters deduced from calculated IV-curve:

Voc = 0.551528 Volt

Jsc = 31.73835382 mA/cm2

FF = 64.0148 %

eta = 11.2055 %

V_MPP = 0.433184 Volt

J_MPP = 25.86783048 mA/cm2

# **In_2_S_3_ QDs (Eg = 2.98 eV)**

SCAPS 3.3.07 ELIS-UGent: Version scaps3307.exe, dated 27-01-2018, 10:11:02

Simulation of I-V curves

Single shot simulation # 4

Calculation started : 16-9-2021 at 15:04:17

problem definition file : c:\Program Files (x86)\Scaps3307\def\example CIGS.def

last saved: 01-09-2015 at 07:44:06

Calculation under illumination

Optical generation from internal SCAPS calculation

Illumination spectrum read from file

spectrum file :

C:\Program Files (x86)\Scaps3307\spectrum\AM1_5G 1 sun.spe last saved: 22-05-2009 at 09:59:48

Neutral density filter: ND 0.0000

Transmission of ND filter: 1.0000e+00

Power from spectrum: 100.0000 mW/cm2

Incident power on solar cell structure 100.0000 mW/cm2

Working point conditions

Temperature 300.00 K

Workpoint bias voltage 0.0000 Volt

Frequency 1.0000e+06 Hz

Voltage reference convention: voltage referred to right contact, voltage V applied to left contact

Current reference convention: current positive when entering the left contact

Power generated by the cell: = -V*I; power consumed by the cell = +V*I

v(V) jtot(mA/cm2) j_total_rec(mA/cm2) j_total_gen(mA/cm2) jbulk(mA/cm2) jifr(mA/cm2) jminor_left(mA/cm2) jminor_right(mA/cm2) j_SRH(mA/cm2) j_Radiative(mA/cm2) j_Auger(mA/cm2)

0.000000 -3.07987256e+01 7.57926823e+00 3.83776600e+01 6.60989222e+00 6.07778267e-01 2.18577108e-01 1.43020637e-01 6.60989222e+00 0.00000000e+00 0.00000000e+00

0.020000 -3.06209814e+01 7.75653533e+00 3.83776600e+01 6.77964912e+00 6.14897311e-01 2.18956136e-01 1.43032764e-01 6.77964912e+00 0.00000000e+00 0.00000000e+00

0.040000 -3.04314853e+01 7.94606356e+00 3.83776600e+01 6.96147691e+00 6.22206806e-01 2.19334799e-01 1.43045042e-01 6.96147691e+00 0.00000000e+00 0.00000000e+00

0.060000 -3.02272066e+01 8.15036877e+00 3.83776600e+01 7.15788160e+00 6.29716428e-01 2.19713262e-01 1.43057477e-01 7.15788160e+00 0.00000000e+00 0.00000000e+00

0.080000 -3.00045877e+01 8.37299626e+00 3.83776600e+01 7.37239644e+00 6.37437987e-01 2.20091779e-01 1.43070056e-01 7.37239644e+00 0.00000000e+00 0.00000000e+00

0.100000 -2.97587211e+01 8.61887638e+00 3.83776600e+01 7.60993845e+00 6.45384530e-01 2.20470633e-01 1.43082768e-01 7.60993845e+00 0.00000000e+00 0.00000000e+00

0.120000 -2.94827895e+01 8.89480223e+00 3.83776600e+01 7.87728598e+00 6.53570507e-01 2.20850140e-01 1.43095602e-01 7.87728598e+00 0.00000000e+00 0.00000000e+00

0.140000 -2.91676156e+01 9.20997765e+00 3.83776600e+01 8.18362648e+00 6.62011973e-01 2.21230657e-01 1.43108546e-01 8.18362648e+00 0.00000000e+00 0.00000000e+00

0.160000 -2.88009898e+01 9.57658839e+00 3.83776600e+01 8.54112738e+00 6.70726831e-01 2.21612590e-01 1.43121587e-01 8.54112738e+00 0.00000000e+00 0.00000000e+00

0.180000 -2.83672589e+01 1.00102991e+01 3.83776600e+01 8.96543288e+00 6.79735133e-01 2.21996409e-01 1.43134714e-01 8.96543288e+00 0.00000000e+00 0.00000000e+00

0.200000 -2.78470691e+01 1.05304824e+01 3.83776600e+01 9.47589234e+00 6.89059449e-01 2.22382658e-01 1.43147915e-01 9.47589234e+00 0.00000000e+00 0.00000000e+00

0.220000 -2.72177421e+01 1.11598006e+01 3.83776600e+01 1.00951421e+01 6.98725309e-01 2.22771976e-01 1.43161179e-01 1.00951421e+01 0.00000000e+00 0.00000000e+00

0.240000 -2.64550664e+01 1.19224612e+01 3.83776600e+01 1.08473598e+01 7.08761758e-01 2.23165115e-01 1.43174496e-01 1.08473598e+01 0.00000000e+00 0.00000000e+00

0.260000 -2.55374290e+01 1.28400786e+01 3.83776600e+01 1.17541258e+01 7.19201988e-01 2.23562954e-01 1.43187855e-01 1.17541258e+01 0.00000000e+00 0.00000000e+00

0.280000 -2.44535901e+01 1.39239122e+01 3.83776600e+01 1.28266604e+01 7.30084031e-01 2.23966516e-01 1.43201248e-01 1.28266604e+01 0.00000000e+00 0.00000000e+00

0.300000 -2.32142105e+01 1.51632651e+01 3.83776600e+01 1.40542219e+01 7.41451461e-01 2.24376978e-01 1.43214666e-01 1.40542219e+01 0.00000000e+00 0.00000000e+00

0.320000 -2.18635547e+01 1.65139189e+01 3.83776600e+01 1.53925409e+01 7.53354168e-01 2.24795719e-01 1.43228102e-01 1.53925409e+01 0.00000000e+00 0.00000000e+00

0.340000 -2.04793524e+01 1.78981165e+01 3.83776600e+01 1.67638005e+01 7.65849922e-01 2.25224507e-01 1.43241551e-01 1.67638005e+01 0.00000000e+00 0.00000000e+00

0.360000 -1.91480812e+01 1.92293915e+01 3.83776600e+01 1.80814620e+01 7.79008568e-01 2.25665919e-01 1.43255006e-01 1.80814620e+01 0.00000000e+00 0.00000000e+00

0.380000 -1.79236534e+01 2.04538276e+01 3.83776600e+01 1.92915142e+01 7.92920934e-01 2.26123975e-01 1.43268465e-01 1.92915142e+01 0.00000000e+00 0.00000000e+00

0.400000 -1.68058482e+01 2.15716421e+01 3.83776600e+01 2.03940429e+01 8.07712561e-01 2.26604734e-01 1.43281928e-01 2.03940429e+01 0.00000000e+00 0.00000000e+00

0.420000 -1.57555967e+01 2.26219006e+01 3.83776600e+01 2.14279261e+01 8.23562394e-01 2.27116708e-01 1.43295399e-01 2.14279261e+01 0.00000000e+00 0.00000000e+00

0.440000 -1.47233447e+01 2.36541561e+01 3.83776600e+01 2.24424451e+01 8.40731061e-01 2.27671124e-01 1.43308880e-01 2.24424451e+01 0.00000000e+00 0.00000000e+00

0.460000 -1.36669518e+01 2.47105496e+01 3.83776600e+01 2.34793372e+01 8.59608162e-01 2.28281945e-01 1.43322378e-01 2.34793372e+01 0.00000000e+00 0.00000000e+00

0.480000 -1.25579972e+01 2.58198861e+01 3.83776600e+01 2.45667931e+01 8.80791848e-01 2.28965244e-01 1.43335898e-01 2.45667931e+01 0.00000000e+00 0.00000000e+00

0.500000 -1.13811696e+01 2.69966763e+01 3.83776600e+01 2.57183712e+01 9.05218379e-01 2.29737262e-01 1.43349450e-01 2.57183712e+01 0.00000000e+00 0.00000000e+00

0.520000 -1.01365066e+01 2.82412177e+01 3.83776600e+01 2.69328688e+01 9.34374832e-01 2.30611059e-01 1.43363037e-01 2.69328688e+01 0.00000000e+00 0.00000000e+00

0.540000 -8.83779861e+00 2.95399346e+01 3.83776600e+01 2.81943313e+01 9.70635066e-01 2.31591631e-01 1.43376659e-01 2.81943313e+01 0.00000000e+00 0.00000000e+00

0.560000 -7.50969065e+00 3.08680162e+01 3.83776600e+01 2.94741641e+01 1.01779048e+00 2.32671270e-01 1.43390308e-01 2.94741641e+01 0.00000000e+00 0.00000000e+00

0.580000 -6.18160222e+00 3.21960800e+01 3.83776600e+01 3.07369745e+01 1.08187392e+00 2.33827572e-01 1.43403966e-01 3.07369745e+01 0.00000000e+00 0.00000000e+00

0.600000 -4.87661662e+00 3.35010443e+01 3.83776600e+01 3.19501765e+01 1.17242380e+00 2.35026412e-01 1.43417601e-01 3.19501765e+01 0.00000000e+00 0.00000000e+00

0.620000 -3.59865042e+00 3.47789529e+01 3.83776600e+01 3.30948882e+01 1.30440359e+00 2.36229973e-01 1.43431168e-01 3.30948882e+01 0.00000000e+00 0.00000000e+00

0.640000 -2.32106630e+00 3.60565208e+01 3.83776600e+01 3.41745778e+01 1.50109126e+00 2.37407175e-01 1.43444608e-01 3.41745778e+01 0.00000000e+00 0.00000000e+00

0.660000 -9.77653954e-01 3.73998737e+01 3.83776600e+01 3.52194759e+01 1.79839736e+00 2.38542677e-01 1.43457851e-01 3.52194759e+01 0.00000000e+00 0.00000000e+00

0.680000 5.44597546e-01 3.89220771e+01 3.83776600e+01 3.62876835e+01 2.25128062e+00 2.39642166e-01 1.43470825e-01 3.62876835e+01 0.00000000e+00 0.00000000e+00

0.700000 2.41578963e+00 4.07932673e+01 3.83776600e+01 3.74658197e+01 2.94322979e+00 2.40734330e-01 1.43483461e-01 3.74658197e+01 0.00000000e+00 0.00000000e+00

0.720000 4.88010786e+00 4.32575839e+01 3.83776600e+01 3.88720739e+01 4.00014264e+00 2.41871676e-01 1.43495703e-01 3.88720739e+01 0.00000000e+00 0.00000000e+00

0.740000 8.28291343e+00 4.66603881e+01 3.83776600e+01 4.06634975e+01 5.61025050e+00 2.43132581e-01 1.43507512e-01 4.06634975e+01 0.00000000e+00 0.00000000e+00

0.760000 1.31107958e+01 5.14882694e+01 3.83776600e+01 4.30482409e+01 8.05188363e+00 2.44625979e-01 1.43518874e-01 4.30482409e+01 0.00000000e+00 0.00000000e+00

0.780000 2.00457498e+01 5.84233057e+01 3.83776600e+01 4.63026146e+01 1.17306621e+01 2.46499199e-01 1.43529798e-01 4.63026146e+01 0.00000000e+00 0.00000000e+00

0.800000 3.00336033e+01 6.84115876e+01 3.83776600e+01 5.07923295e+01 1.72267689e+01 2.48948988e-01 1.43540318e-01 5.07923295e+01 0.00000000e+00 0.00000000e+00

solar cell parameters deduced from calculated IV-curve:

Voc = 0.673110 Volt

Jsc = 30.79872557 mA/cm2

FF = 33.7483 %

eta = 6.9963 %

V_MPP = 0.319902 Volt

J_MPP = 21.87023057 mA/cm2

SCAPS 3.3.07 ELIS-UGent: Version scaps3307.exe, dated 27-01-2018, 10:11:02

Simulation of I-V curves

Single shot simulation # 5

Calculation started : 16-9-2021 at 15:04:39

problem definition file : c:\Program Files (x86)\Scaps3307\def\example CIGS.def

last saved: 01-09-2015 at 07:44:06

Calculation under illumination

Optical generation from internal SCAPS calculation

Illumination spectrum read from file

spectrum file :

C:\Program Files (x86)\Scaps3307\spectrum\AM1_5G 1 sun.spe last saved: 22-05-2009 at 09:59:48

Neutral density filter: ND 0.0000

Transmission of ND filter: 1.0000e+00

Power from spectrum: 100.0000 mW/cm2

Incident power on solar cell structure 100.0000 mW/cm2

Working point conditions

Temperature 300.00 K

Workpoint bias voltage 0.0000 Volt

Frequency 1.0000e+06 Hz

Voltage reference convention: voltage referred to right contact, voltage V applied to left contact

Current reference convention: current positive when entering the left contact

Power generated by the cell: = -V*I; power consumed by the cell = +V*I

v(V) jtot(mA/cm2) j_total_rec(mA/cm2) j_total_gen(mA/cm2) jbulk(mA/cm2) jifr(mA/cm2) jminor_left(mA/cm2) jminor_right(mA/cm2) j_SRH(mA/cm2) j_Radiative(mA/cm2) j_Auger(mA/cm2)

0.000000 -3.19636510e+01 6.41383319e+00 3.83776600e+01 5.49729725e+00 5.55623594e-01 2.15361824e-01 1.45550521e-01 5.49729725e+00 0.00000000e+00 0.00000000e+00

0.020000 -3.18377297e+01 6.53988021e+00 3.83776600e+01 5.61685708e+00 5.61694723e-01 2.15777476e-01 1.45550933e-01 5.61685708e+00 0.00000000e+00 0.00000000e+00

0.040000 -3.17055029e+01 6.67210847e+00 3.83776600e+01 5.74242535e+00 5.67939240e-01 2.16192529e-01 1.45551350e-01 5.74242535e+00 0.00000000e+00 0.00000000e+00

0.060000 -3.15665342e+01 6.81107770e+00 3.83776600e+01 5.87455267e+00 5.74366078e-01 2.16607176e-01 1.45551773e-01 5.87455267e+00 0.00000000e+00 0.00000000e+00

0.080000 -3.14201789e+01 6.95743691e+00 3.83776600e+01 6.01387818e+00 5.80984924e-01 2.17021602e-01 1.45552202e-01 6.01387818e+00 0.00000000e+00 0.00000000e+00

0.100000 -3.12656636e+01 7.11195874e+00 3.83776600e+01 6.16116382e+00 5.87806276e-01 2.17436003e-01 1.45552638e-01 6.16116382e+00 0.00000000e+00 0.00000000e+00

0.120000 -3.11020335e+01 7.27558822e+00 3.83776600e+01 6.31734301e+00 5.94841539e-01 2.17850589e-01 1.45553080e-01 6.31734301e+00 0.00000000e+00 0.00000000e+00

0.140000 -3.09281065e+01 7.44951918e+00 3.83776600e+01 6.48359692e+00 6.02103140e-01 2.18265590e-01 1.45553530e-01 6.48359692e+00 0.00000000e+00 0.00000000e+00

0.160000 -3.07422985e+01 7.63531834e+00 3.83776600e+01 6.66147845e+00 6.09604655e-01 2.18681255e-01 1.45553988e-01 6.66147845e+00 0.00000000e+00 0.00000000e+00

0.180000 -3.05425074e+01 7.83512112e+00 3.83776600e+01 6.85310784e+00 6.17360972e-01 2.19097858e-01 1.45554453e-01 6.85310784e+00 0.00000000e+00 0.00000000e+00

0.200000 -3.03256910e+01 8.05193495e+00 3.83776600e+01 7.06147586e+00 6.25388463e-01 2.19515701e-01 1.45554928e-01 7.06147586e+00 0.00000000e+00 0.00000000e+00

0.220000 -3.00875369e+01 8.29009366e+00 3.83776600e+01 7.29089791e+00 6.33705212e-01 2.19935121e-01 1.45555412e-01 7.29089791e+00 0.00000000e+00 0.00000000e+00

0.240000 -2.98217143e+01 8.55590644e+00 3.83776600e+01 7.54766277e+00 6.42331269e-01 2.20356498e-01 1.45555906e-01 7.54766277e+00 0.00000000e+00 0.00000000e+00

0.260000 -2.95190853e+01 8.85853384e+00 3.83776600e+01 7.84090818e+00 6.51288972e-01 2.20780269e-01 1.45556411e-01 7.84090818e+00 0.00000000e+00 0.00000000e+00

0.280000 -2.91665348e+01 9.21109378e+00 3.83776600e+01 8.18372659e+00 6.60603330e-01 2.21206937e-01 1.45556928e-01 8.18372659e+00 0.00000000e+00 0.00000000e+00

0.300000 -2.87456624e+01 9.63194745e+00 3.83776600e+01 8.59445039e+00 6.70302501e-01 2.21637098e-01 1.45557457e-01 8.59445039e+00 0.00000000e+00 0.00000000e+00

0.320000 -2.82315944e+01 1.01460072e+01 3.83776600e+01 9.09795930e+00 6.80418386e-01 2.22071464e-01 1.45558001e-01 9.09795930e+00 0.00000000e+00 0.00000000e+00

0.340000 -2.75919258e+01 1.07856674e+01 3.83776600e+01 9.72661057e+00 6.90987381e-01 2.22510905e-01 1.45558560e-01 9.72661057e+00 0.00000000e+00 0.00000000e+00

0.360000 -2.67871434e+01 1.15904332e+01 3.83776600e+01 1.05198663e+01 7.02051349e-01 2.22956487e-01 1.45559137e-01 1.05198663e+01 0.00000000e+00 0.00000000e+00

0.380000 -2.57742225e+01 1.26033375e+01 3.83776600e+01 1.15207094e+01 7.13658888e-01 2.23409540e-01 1.45559733e-01 1.15207094e+01 0.00000000e+00 0.00000000e+00

0.400000 -2.45166333e+01 1.38609078e+01 3.83776600e+01 1.27656087e+01 7.25867043e-01 2.23871708e-01 1.45560351e-01 1.27656087e+01 0.00000000e+00 0.00000000e+00

0.420000 -2.30043353e+01 1.53731855e+01 3.83776600e+01 1.42645358e+01 7.38743669e-01 2.24345021e-01 1.45560992e-01 1.42645358e+01 0.00000000e+00 0.00000000e+00

0.440000 -2.12813495e+01 1.70961592e+01 3.83776600e+01 1.59733947e+01 7.52370921e-01 2.24832009e-01 1.45561658e-01 1.59733947e+01 0.00000000e+00 0.00000000e+00

0.460000 -1.94591392e+01 1.89183499e+01 3.83776600e+01 1.77806004e+01 7.66850997e-01 2.25336106e-01 1.45562352e-01 1.77806004e+01 0.00000000e+00 0.00000000e+00

0.480000 -1.76764069e+01 2.07010803e+01 3.83776600e+01 1.95473379e+01 7.82316600e-01 2.25862700e-01 1.45563074e-01 1.95473379e+01 0.00000000e+00 0.00000000e+00

0.500000 -1.60118464e+01 2.23656486e+01 3.83776600e+01 2.11947138e+01 7.98950010e-01 2.26420938e-01 1.45563829e-01 2.11947138e+01 0.00000000e+00 0.00000000e+00

0.520000 -1.44409805e+01 2.39365243e+01 3.83776600e+01 2.27469177e+01 8.17016198e-01 2.27025749e-01 1.45564621e-01 2.27469177e+01 0.00000000e+00 0.00000000e+00

0.540000 -1.28757724e+01 2.55017384e+01 3.83776600e+01 2.42915520e+01 8.36921191e-01 2.27699785e-01 1.45565459e-01 2.42915520e+01 0.00000000e+00 0.00000000e+00

0.560000 -1.12194512e+01 2.71580609e+01 3.83776600e+01 2.59246996e+01 8.59319726e-01 2.28475241e-01 1.45566351e-01 2.59246996e+01 0.00000000e+00 0.00000000e+00

0.580000 -9.39223724e+00 2.89852727e+01 3.83776600e+01 2.77249940e+01 8.85316784e-01 2.29394533e-01 1.45567309e-01 2.77249940e+01 0.00000000e+00 0.00000000e+00

0.600000 -7.33806682e+00 3.10394388e+01 3.83776600e+01 2.97465199e+01 9.16843209e-01 2.30507376e-01 1.45568346e-01 2.97465199e+01 0.00000000e+00 0.00000000e+00

0.620000 -5.02761048e+00 3.33498897e+01 3.83776600e+01 3.20151094e+01 9.57349236e-01 2.31861568e-01 1.45569478e-01 3.20151094e+01 0.00000000e+00 0.00000000e+00

0.640000 -2.45881896e+00 3.59186754e+01 3.83776600e+01 3.45265497e+01 1.01306730e+00 2.33487650e-01 1.45570725e-01 3.45265497e+01 0.00000000e+00 0.00000000e+00

0.660000 3.53842043e-01 3.87313306e+01 3.83776600e+01 3.72551063e+01 1.09526776e+00 2.35384455e-01 1.45572109e-01 3.72551063e+01 0.00000000e+00 0.00000000e+00

0.680000 3.40959037e+00 4.17870735e+01 3.83776600e+01 4.01797898e+01 1.22419152e+00 2.37518587e-01 1.45573658e-01 4.01797898e+01 0.00000000e+00 0.00000000e+00

0.700000 6.76694110e+00 4.51444194e+01 3.83776600e+01 4.33232855e+01 1.43571250e+00 2.39845939e-01 1.45575400e-01 4.33232855e+01 0.00000000e+00 0.00000000e+00

0.720000 1.05893075e+01 4.89667759e+01 3.83776600e+01 4.67865853e+01 1.79226592e+00 2.42347350e-01 1.45577366e-01 4.67865853e+01 0.00000000e+00 0.00000000e+00

0.740000 1.51798255e+01 5.35572622e+01 3.83776600e+01 5.07664767e+01 2.40014592e+00 2.45060016e-01 1.45579588e-01 5.07664767e+01 0.00000000e+00 0.00000000e+00

0.760000 2.10108832e+01 5.93883252e+01 3.83776600e+01 5.55588257e+01 3.43582401e+00 2.48093397e-01 1.45582093e-01 5.55588257e+01 0.00000000e+00 0.00000000e+00

0.780000 2.87638454e+01 6.71413147e+01 3.83776600e+01 6.15598820e+01 5.18421524e+00 2.51632549e-01 1.45584905e-01 6.15598820e+01 0.00000000e+00 0.00000000e+00

0.800000 3.93897409e+01 7.77672056e+01 3.83776600e+01 6.92743498e+01 8.09132858e+00 2.55939197e-01 1.45588050e-01 6.92743498e+01 0.00000000e+00 0.00000000e+00

solar cell parameters deduced from calculated IV-curve:

Voc = 0.657576 Volt

Jsc = 31.96365096 mA/cm2

FF = 46.7144 %

eta = 9.8187 %

V_MPP = 0.391934 Volt

J_MPP = 25.05190003 mA/cm2

SCAPS 3.3.07 ELIS-UGent: Version scaps3307.exe, dated 27-01-2018, 10:11:02

Simulation of I-V curves

Single shot simulation # 6

Calculation started : 16-9-2021 at 15:05:04

problem definition file : c:\Program Files (x86)\Scaps3307\def\example CIGS.def

last saved: 01-09-2015 at 07:44:06

Calculation under illumination

Optical generation from internal SCAPS calculation

Illumination spectrum read from file

spectrum file :

C:\Program Files (x86)\Scaps3307\spectrum\AM1_5G 1 sun.spe last saved: 22-05-2009 at 09:59:48

Neutral density filter: ND 0.0000

Transmission of ND filter: 1.0000e+00

Power from spectrum: 100.0000 mW/cm2

Incident power on solar cell structure 100.0000 mW/cm2

Working point conditions

Temperature 300.00 K

Workpoint bias voltage 0.0000 Volt

Frequency 1.0000e+06 Hz

Voltage reference convention: voltage referred to right contact, voltage V applied to left contact

Current reference convention: current positive when entering the left contact

Power generated by the cell: = -V*I; power consumed by the cell = +V*I

v(V) jtot(mA/cm2) j_total_rec(mA/cm2) j_total_gen(mA/cm2) jbulk(mA/cm2) jifr(mA/cm2) jminor_left(mA/cm2) jminor_right(mA/cm2) j_SRH(mA/cm2) j_Radiative(mA/cm2) j_Auger(mA/cm2)

0.000000 -3.26688881e+01 5.70859448e+00 3.83776600e+01 4.83263743e+00 5.05740180e-01 2.11833606e-01 1.58383267e-01 4.83263743e+00 0.00000000e+00 0.00000000e+00

0.020000 -3.25676086e+01 5.80999415e+00 3.83776600e+01 4.92853169e+00 5.10801563e-01 2.12277551e-01 1.58383347e-01 4.92853169e+00 0.00000000e+00 0.00000000e+00

0.040000 -3.24617539e+01 5.91585292e+00 3.83776600e+01 5.02875781e+00 5.15991939e-01 2.12719743e-01 1.58383427e-01 5.02875781e+00 0.00000000e+00 0.00000000e+00

0.060000 -3.23510919e+01 6.02651942e+00 3.83776600e+01 5.13365851e+00 5.21317075e-01 2.13160332e-01 1.58383509e-01 5.13365851e+00 0.00000000e+00 0.00000000e+00

0.080000 -3.22352381e+01 6.14237802e+00 3.83776600e+01 5.24361183e+00 5.26783145e-01 2.13599451e-01 1.58383591e-01 5.24361183e+00 0.00000000e+00 0.00000000e+00

0.100000 -3.21137611e+01 6.26386014e+00 3.83776600e+01 5.35904246e+00 5.32396758e-01 2.14037239e-01 1.58383675e-01 5.35904246e+00 0.00000000e+00 0.00000000e+00

0.120000 -3.19861680e+01 6.39145891e+00 3.83776600e+01 5.48043630e+00 5.38165002e-01 2.14473844e-01 1.58383760e-01 5.48043630e+00 0.00000000e+00 0.00000000e+00

0.140000 -3.18518910e+01 6.52574191e+00 3.83776600e+01 5.60835314e+00 5.44095492e-01 2.14909425e-01 1.58383847e-01 5.60835314e+00 0.00000000e+00 0.00000000e+00

0.160000 -3.17102719e+01 6.66736763e+00 3.83776600e+01 5.74344312e+00 5.50196430e-01 2.15344147e-01 1.58383934e-01 5.74344312e+00 0.00000000e+00 0.00000000e+00

0.180000 -3.15605282e+01 6.81711871e+00 3.83776600e+01 5.88647984e+00 5.56476660e-01 2.15778188e-01 1.58384023e-01 5.88647984e+00 0.00000000e+00 0.00000000e+00

0.200000 -3.14016980e+01 6.97595694e+00 3.83776600e+01 6.03841533e+00 5.62945749e-01 2.16211740e-01 1.58384114e-01 6.03841533e+00 0.00000000e+00 0.00000000e+00

0.220000 -3.12325552e+01 7.14510846e+00 3.83776600e+01 6.20046518e+00 5.69614063e-01 2.16645007e-01 1.58384206e-01 6.20046518e+00 0.00000000e+00 0.00000000e+00

0.240000 -3.10514625e+01 7.32621091e+00 3.83776600e+01 6.37425553e+00 5.76492868e-01 2.17078211e-01 1.58384300e-01 6.37425553e+00 0.00000000e+00 0.00000000e+00

0.260000 -3.08561143e+01 7.52156960e+00 3.83776600e+01 6.56207917e+00 5.83594436e-01 2.17511596e-01 1.58384395e-01 6.56207917e+00 0.00000000e+00 0.00000000e+00

0.280000 -3.06431085e+01 7.73458652e+00 3.83776600e+01 6.76732443e+00 5.90932178e-01 2.17945429e-01 1.58384492e-01 6.76732443e+00 0.00000000e+00 0.00000000e+00

0.300000 -3.04072407e+01 7.97046581e+00 3.83776600e+01 6.99518042e+00 5.98520790e-01 2.18380008e-01 1.58384591e-01 6.99518042e+00 0.00000000e+00 0.00000000e+00

0.320000 -3.01403829e+01 8.23733506e+00 3.83776600e+01 7.25375826e+00 6.06376433e-01 2.18815677e-01 1.58384692e-01 7.25375826e+00 0.00000000e+00 0.00000000e+00

0.340000 -2.98297739e+01 8.54795446e+00 3.83776600e+01 7.55579989e+00 6.14516936e-01 2.19252836e-01 1.58384795e-01 7.55579989e+00 0.00000000e+00 0.00000000e+00

0.360000 -2.94555016e+01 8.92223519e+00 3.83776600e+01 7.92119627e+00 6.22962050e-01 2.19691968e-01 1.58384901e-01 7.92119627e+00 0.00000000e+00 0.00000000e+00

0.380000 -2.89868604e+01 9.39088166e+00 3.83776600e+01 8.38062923e+00 6.31733734e-01 2.20133684e-01 1.58385009e-01 8.38062923e+00 0.00000000e+00 0.00000000e+00

0.400000 -2.83770744e+01 1.00006686e+01 3.83776600e+01 8.98084817e+00 6.40856517e-01 2.20578787e-01 1.58385120e-01 8.98084817e+00 0.00000000e+00 0.00000000e+00

0.420000 -2.75555308e+01 1.08222073e+01 3.83776600e+01 9.79243574e+00 6.50357924e-01 2.21028395e-01 1.58385233e-01 9.79243574e+00 0.00000000e+00 0.00000000e+00

0.440000 -2.64161623e+01 1.19615635e+01 3.83776600e+01 1.09214250e+01 6.60269001e-01 2.21484155e-01 1.58385351e-01 1.09214250e+01 0.00000000e+00 0.00000000e+00

0.460000 -2.48000241e+01 1.35776799e+01 3.83776600e+01 1.25267208e+01 6.70624964e-01 2.21948645e-01 1.58385471e-01 1.25267208e+01 0.00000000e+00 0.00000000e+00

0.480000 -2.24693838e+01 1.59082860e+01 3.83776600e+01 1.48460083e+01 6.81466009e-01 2.22426102e-01 1.58385595e-01 1.48460083e+01 0.00000000e+00 0.00000000e+00

0.500000 -1.90697072e+01 1.93079137e+01 3.83776600e+01 1.82337658e+01 6.92838345e-01 2.22923802e-01 1.58385723e-01 1.82337658e+01 0.00000000e+00 0.00000000e+00

0.520000 -1.40753392e+01 2.43022180e+01 3.83776600e+01 2.32155820e+01 7.04795556e-01 2.23454604e-01 1.58385857e-01 2.32155820e+01 0.00000000e+00 0.00000000e+00

0.540000 -6.71520004e+00 3.16622904e+01 3.83776600e+01 3.05624623e+01 7.17400436e-01 2.24041642e-01 1.58385997e-01 3.05624623e+01 0.00000000e+00 0.00000000e+00

0.560000 4.12157990e+00 4.24990492e+01 3.83776600e+01 4.13852086e+01 7.30727675e-01 2.24726780e-01 1.58386143e-01 4.13852086e+01 0.00000000e+00 0.00000000e+00

0.580000 1.99882754e+01 5.83658928e+01 3.83776600e+01 5.72370532e+01 7.44867969e-01 2.25585319e-01 1.58386298e-01 5.72370532e+01 0.00000000e+00 0.00000000e+00

0.600000 4.29515910e+01 8.13290392e+01 3.83776600e+01 8.01839678e+01 7.59934789e-01 2.26750137e-01 1.58386462e-01 8.01839678e+01 0.00000000e+00 0.00000000e+00

0.620000 7.55309749e+01 1.13908394e+02 3.83776600e+01 1.12745482e+02 7.76077166e-01 2.28448749e-01 1.58386639e-01 1.12745482e+02 0.00000000e+00 0.00000000e+00

0.640000 1.20333271e+02 1.58710610e+02 3.83776600e+01 1.57527667e+02 7.93502269e-01 2.31053374e-01 1.58386831e-01 1.57527667e+02 0.00000000e+00 0.00000000e+00

0.660000 1.79174526e+02 2.17551695e+02 3.83776600e+01 2.16345643e+02 8.12520837e-01 2.35144065e-01 1.58387040e-01 2.16345643e+02 0.00000000e+00 0.00000000e+00

0.680000 2.51803744e+02 2.90180588e+02 3.83776600e+01 2.88946968e+02 8.33634626e-01 2.41597843e-01 1.58387267e-01 2.88946968e+02 0.00000000e+00 0.00000000e+00

0.700000 3.35334511e+02 3.73710809e+02 3.83776600e+01 3.72442957e+02 8.57702404e-01 2.51762539e-01 1.58387514e-01 3.72442957e+02 0.00000000e+00 0.00000000e+00

0.720000 4.26147828e+02 4.64523307e+02 3.83776600e+01 4.63210906e+02 8.86249576e-01 2.67763198e-01 1.58387782e-01 4.63210906e+02 0.00000000e+00 0.00000000e+00

0.740000 5.23318432e+02 5.61692908e+02 3.83776600e+01 5.60319722e+02 9.22058650e-01 2.92738701e-01 1.58388073e-01 5.60319722e+02 0.00000000e+00 0.00000000e+00

0.760000 6.29289173e+02 6.67667347e+02 3.83776600e+01 6.66208057e+02 9.70326027e-01 3.30575395e-01 1.58388395e-01 6.66208057e+02 0.00000000e+00 0.00000000e+00

0.780000 7.47378057e+02 7.85756348e+02 3.83776600e+01 7.84172016e+02 1.04094462e+00 3.84998181e-01 1.58388755e-01 7.84172016e+02 0.00000000e+00 0.00000000e+00

0.800000 8.79662368e+02 9.18040654e+02 3.83776600e+01 9.16270985e+02 1.15281819e+00 4.58462556e-01 1.58389165e-01 9.16270985e+02 0.00000000e+00 0.00000000e+00

solar cell parameters deduced from calculated IV-curve:

Voc = 0.553117 Volt

Jsc = 32.66888807 mA/cm2

FF = 64.3709 %

eta = 11.6316 %

V_MPP = 0.434707 Volt

J_MPP = 26.75743212 mA/cm2

# **Ag_2_S nanoparticle (Eg = 1.1 eV)**

SCAPS 3.3.07 ELIS-UGent: Version scaps3307.exe, dated 27-01-2018, 10:11:02

Simulation of I-V curves

Single shot simulation # 1

Calculation started : 20-9-2021 at 1:52:56

problem definition file : c:\Program Files (x86)\Scaps3307\def\example CIGS.def

last saved: 01-09-2015 at 07:44:06

Calculation under illumination

Optical generation from internal SCAPS calculation

Illumination spectrum read from file

spectrum file :

C:\Program Files (x86)\Scaps3307\spectrum\AM1_5G 1 sun.spe last saved: 22-05-2009 at 09:59:48

Neutral density filter: ND 0.0000

Transmission of ND filter: 1.0000e+00

Power from spectrum: 100.0000 mW/cm2

Incident power on solar cell structure 100.0000 mW/cm2

Working point conditions

Temperature 300.00 K

Workpoint bias voltage 0.0000 Volt

Frequency 1.0000e+06 Hz

Voltage reference convention: voltage referred to right contact, voltage V applied to left contact

Current reference convention: current positive when entering the left contact

Power generated by the cell: = -V*I; power consumed by the cell = +V*I

v(V) jtot(mA/cm2) j_total_rec(mA/cm2) j_total_gen(mA/cm2) jbulk(mA/cm2) jifr(mA/cm2) jminor_left(mA/cm2) jminor_right(mA/cm2) j_SRH(mA/cm2) j_Radiative(mA/cm2) j_Auger(mA/cm2)

0.000000 -9.43817953e+00 3.06162290e+01 4.00438207e+01 1.76876249e+00 2.86605438e+01 1.01119910e-01 8.58027676e-02 1.76876249e+00 0.00000000e+00 0.00000000e+00

0.020000 -9.39232038e+00 3.06507097e+01 4.00438207e+01 1.80195013e+00 2.86616623e+01 1.01294478e-01 8.58028131e-02 1.80195013e+00 0.00000000e+00 0.00000000e+00

0.040000 -9.35641528e+00 3.06866699e+01 4.00438207e+01 1.83660256e+00 2.86627965e+01 1.01467757e-01 8.58031486e-02 1.83660256e+00 0.00000000e+00 0.00000000e+00

0.060000 -9.31890352e+00 3.07242313e+01 4.00438207e+01 1.87284118e+00 2.86639475e+01 1.01639775e-01 8.58028978e-02 1.87284118e+00 0.00000000e+00 0.00000000e+00

0.080000 -9.27965062e+00 3.07635312e+01 4.00438207e+01 1.91080286e+00 2.86651146e+01 1.01810567e-01 8.58032323e-02 1.91080286e+00 0.00000000e+00 0.00000000e+00

0.100000 -9.23849785e+00 3.08047270e+01 4.00438207e+01 1.95064453e+00 2.86662993e+01 1.01980172e-01 8.58029807e-02 1.95064453e+00 0.00000000e+00 0.00000000e+00

0.120000 -9.19525566e+00 3.08480077e+01 4.00438207e+01 1.99255471e+00 2.86675011e+01 1.02148627e-01 8.58033145e-02 1.99255471e+00 0.00000000e+00 0.00000000e+00

0.140000 -9.14969081e+00 3.08936089e+01 4.00438207e+01 2.03676853e+00 2.86687213e+01 1.02315972e-01 8.58030621e-02 2.03676853e+00 0.00000000e+00 0.00000000e+00

0.160000 -9.10149850e+00 3.09418342e+01 4.00438207e+01 2.08358916e+00 2.86699594e+01 1.02482250e-01 8.58033950e-02 2.08358916e+00 0.00000000e+00 0.00000000e+00

0.180000 -9.05026651e+00 3.09930955e+01 4.00438207e+01 2.13342787e+00 2.86712170e+01 1.02647504e-01 8.58031416e-02 2.13342787e+00 0.00000000e+00 0.00000000e+00

0.200000 -8.99540476e+00 3.10479866e+01 4.00438207e+01 2.18687795e+00 2.86724933e+01 1.02811781e-01 8.58034736e-02 2.18687795e+00 0.00000000e+00 0.00000000e+00

0.220000 -8.93600826e+00 3.11074063e+01 4.00438207e+01 2.24483782e+00 2.86737902e+01 1.02975131e-01 8.58032196e-02 2.24483782e+00 0.00000000e+00 0.00000000e+00

0.240000 -8.87068023e+00 3.11727579e+01 4.00438207e+01 2.30870994e+00 2.86751068e+01 1.03137605e-01 8.58035507e-02 2.30870994e+00 0.00000000e+00 0.00000000e+00

0.260000 -8.79719468e+00 3.12462671e+01 4.00438207e+01 2.38071957e+00 2.86764450e+01 1.03299263e-01 8.58032959e-02 2.38071957e+00 0.00000000e+00 0.00000000e+00

0.280000 -8.69894057e+00 3.13314700e+01 4.00438207e+01 2.46440212e+00 2.86778040e+01 1.03460168e-01 8.58036310e-02 2.46440212e+00 0.00000000e+00 0.00000000e+00

0.300000 -8.59715014e+00 3.14339274e+01 4.00438207e+01 2.56531779e+00 2.86791858e+01 1.03620398e-01 8.58033707e-02 2.56531779e+00 0.00000000e+00 0.00000000e+00

0.320000 -8.46945312e+00 3.15622551e+01 4.00438207e+01 2.69208168e+00 2.86805900e+01 1.03780044e-01 8.58034075e-02 2.69208168e+00 0.00000000e+00 0.00000000e+00

0.340000 -8.30267622e+00 3.17296469e+01 4.00438207e+01 2.85788679e+00 2.86820174e+01 1.03939219e-01 8.58034441e-02 2.85788679e+00 0.00000000e+00 0.00000000e+00

0.360000 -8.07677932e+00 3.19561306e+01 4.00438207e+01 3.08276021e+00 2.86834688e+01 1.04098080e-01 8.58034804e-02 3.08276021e+00 0.00000000e+00 0.00000000e+00

0.380000 -7.76153339e+00 3.22719371e+01 4.00438207e+01 3.39693186e+00 2.86849448e+01 1.04256857e-01 8.58035158e-02 3.39693186e+00 0.00000000e+00 0.00000000e+00

0.400000 -7.31141631e+00 3.27225991e+01 4.00438207e+01 3.84593353e+00 2.86864461e+01 1.04415910e-01 8.58035517e-02 3.84593353e+00 0.00000000e+00 0.00000000e+00

0.420000 -6.65774785e+00 3.33767874e+01 4.00438207e+01 4.49843462e+00 2.86879734e+01 1.04575847e-01 8.58035874e-02 4.49843462e+00 0.00000000e+00 0.00000000e+00

0.440000 -5.69657198e+00 3.43384343e+01 4.00438207e+01 5.45836558e+00 2.86895273e+01 1.04737749e-01 8.58036232e-02 5.45836558e+00 0.00000000e+00 0.00000000e+00

0.460000 -4.26999000e+00 3.57654692e+01 4.00438207e+01 6.88365321e+00 2.86911087e+01 1.04903620e-01 8.58036581e-02 6.88365321e+00 0.00000000e+00 0.00000000e+00

0.480000 -2.13720504e+00 3.78986779e+01 4.00438207e+01 9.01507868e+00 2.86927183e+01 1.05077286e-01 8.58036949e-02 9.01507868e+00 0.00000000e+00 0.00000000e+00

0.500000 1.06983199e+00 4.11061081e+01 4.00438207e+01 1.22206815e+01 2.86943567e+01 1.05266178e-01 8.58037316e-02 1.22206815e+01 0.00000000e+00 0.00000000e+00

0.520000 5.91490038e+00 4.59515384e+01 4.00438207e+01 1.70642249e+01 2.86960248e+01 1.05484926e-01 8.58037703e-02 1.70642249e+01 0.00000000e+00 0.00000000e+00

0.540000 1.32634589e+01 5.33004228e+01 4.00438207e+01 2.44111333e+01 2.86977232e+01 1.05762529e-01 8.58038111e-02 2.44111333e+01 0.00000000e+00 0.00000000e+00

0.560000 2.44469545e+01 6.44842375e+01 4.00438207e+01 3.55928244e+01 2.86994525e+01 1.06156719e-01 8.58038563e-02 3.55928244e+01 0.00000000e+00 0.00000000e+00

0.580000 4.15116777e+01 8.15552901e+01 4.00438207e+01 5.26614903e+01 2.87012131e+01 1.06782513e-01 8.58042009e-02 5.26614903e+01 0.00000000e+00 0.00000000e+00

0.600000 6.76358888e+01 1.07679493e+02 4.00438207e+01 7.87828141e+01 2.87030059e+01 1.07868998e-01 8.58042582e-02 7.87828141e+01 0.00000000e+00 0.00000000e+00

0.620000 1.07690921e+02 1.47734437e+02 4.00438207e+01 1.18833931e+02 2.87048314e+01 1.09870465e-01 8.58043382e-02 1.18833931e+02 0.00000000e+00 0.00000000e+00

0.640000 1.69154991e+02 2.09198135e+02 4.00438207e+01 1.80291958e+02 2.87066903e+01 1.13682160e-01 8.58044398e-02 1.80291958e+02 0.00000000e+00 0.00000000e+00

0.660000 2.63371790e+02 3.03413071e+02 4.00438207e+01 2.74497631e+02 2.87085848e+01 1.21050238e-01 8.58042889e-02 2.74497631e+02 0.00000000e+00 0.00000000e+00

0.680000 4.07211257e+02 4.47243479e+02 4.00438207e+01 4.18311827e+02 2.87105180e+01 1.35329266e-01 8.58047810e-02 4.18311827e+02 0.00000000e+00 0.00000000e+00

0.700000 6.24870372e+02 6.64916978e+02 4.00438207e+01 6.35955849e+02 2.87124994e+01 1.62824582e-01 8.58050580e-02 6.35955849e+02 0.00000000e+00 0.00000000e+00

0.720000 9.49329626e+02 9.89394698e+02 4.00438207e+01 9.60379307e+02 2.87145464e+01 2.15038406e-01 8.58054654e-02 9.60379307e+02 0.00000000e+00 0.00000000e+00

0.740000 1.42170759e+03 1.46189278e+03 4.00438207e+01 1.43277814e+03 2.87166905e+01 3.12142152e-01 8.58057741e-02 1.43277814e+03 0.00000000e+00 0.00000000e+00

0.760000 2.08823176e+03 2.12813202e+03 4.00438207e+01 2.09883931e+03 2.87189823e+01 4.87918354e-01 8.58068560e-02 2.09883931e+03 0.00000000e+00 0.00000000e+00

0.780000 2.99012181e+03 3.03038507e+03 4.00438207e+01 3.00078185e+03 2.87214934e+01 7.95919045e-01 8.58079260e-02 3.00078185e+03 0.00000000e+00 0.00000000e+00

0.800000 4.15950118e+03 4.19915970e+03 4.00438207e+01 4.16903225e+03 2.87243134e+01 1.31733061e+00 8.58093338e-02 4.16903225e+03 0.00000000e+00 0.00000000e+00

solar cell parameters deduced from calculated IV-curve:

Voc = 0.33850505 Volt

Jsc = 6.46695928 mA/cm2

FF = 43.3592535 %

eta = 2.02172573 %

V_MPP = 0.383770 Volt

J_MPP = 7.62848514 mA/cm2

SCAPS 3.3.07 ELIS-UGent: Version scaps3307.exe, dated 27-01-2018, 10:11:02

Simulation of I-V curves

Single shot simulation # 2

Calculation started : 20-9-2021 at 1:55:56

problem definition file : c:\Program Files (x86)\Scaps3307\def\example CIGS.def

last saved: 01-09-2015 at 07:44:06

Calculation under illumination

Optical generation from internal SCAPS calculation

Illumination spectrum read from file

spectrum file :

C:\Program Files (x86)\Scaps3307\spectrum\AM1_5G 1 sun.spe last saved: 22-05-2009 at 09:59:48

Neutral density filter: ND 0.0000

Transmission of ND filter: 1.0000e+00

Power from spectrum: 100.0000 mW/cm2

Incident power on solar cell structure 100.0000 mW/cm2

Working point conditions

Temperature 300.00 K

Workpoint bias voltage 0.0000 Volt

Frequency 1.0000e+06 Hz

Voltage reference convention: voltage referred to right contact, voltage V applied to left contact

Current reference convention: current positive when entering the left contact

Power generated by the cell: = -V*I; power consumed by the cell = +V*I

v(V) jtot(mA/cm2) j_total_rec(mA/cm2) j_total_gen(mA/cm2) jbulk(mA/cm2) jifr(mA/cm2) jminor_left(mA/cm2) jminor_right(mA/cm2) j_SRH(mA/cm2) j_Radiative(mA/cm2) j_Auger(mA/cm2)

0.000000 -9.43817953e+00 3.06162290e+01 4.00438207e+01 1.76876249e+00 2.86605438e+01 1.01119910e-01 8.58027676e-02 1.76876249e+00 0.00000000e+00 0.00000000e+00

0.020000 -9.39232038e+00 3.06507097e+01 4.00438207e+01 1.80195013e+00 2.86616623e+01 1.01294478e-01 8.58028131e-02 1.80195013e+00 0.00000000e+00 0.00000000e+00

0.040000 -9.35641528e+00 3.06866699e+01 4.00438207e+01 1.83660256e+00 2.86627965e+01 1.01467757e-01 8.58031486e-02 1.83660256e+00 0.00000000e+00 0.00000000e+00

0.060000 -9.31890352e+00 3.07242313e+01 4.00438207e+01 1.87284118e+00 2.86639475e+01 1.01639775e-01 8.58028978e-02 1.87284118e+00 0.00000000e+00 0.00000000e+00

0.080000 -9.27965062e+00 3.07635312e+01 4.00438207e+01 1.91080286e+00 2.86651146e+01 1.01810567e-01 8.58032323e-02 1.91080286e+00 0.00000000e+00 0.00000000e+00

0.100000 -9.23849785e+00 3.08047270e+01 4.00438207e+01 1.95064453e+00 2.86662993e+01 1.01980172e-01 8.58029807e-02 1.95064453e+00 0.00000000e+00 0.00000000e+00

0.120000 -9.19525566e+00 3.08480077e+01 4.00438207e+01 1.99255471e+00 2.86675011e+01 1.02148627e-01 8.58033145e-02 1.99255471e+00 0.00000000e+00 0.00000000e+00

0.140000 -9.14969081e+00 3.08936089e+01 4.00438207e+01 2.03676853e+00 2.86687213e+01 1.02315972e-01 8.58030621e-02 2.03676853e+00 0.00000000e+00 0.00000000e+00

0.160000 -9.10149850e+00 3.09418342e+01 4.00438207e+01 2.08358916e+00 2.86699594e+01 1.02482250e-01 8.58033950e-02 2.08358916e+00 0.00000000e+00 0.00000000e+00

0.180000 -9.05026651e+00 3.09930955e+01 4.00438207e+01 2.13342787e+00 2.86712170e+01 1.02647504e-01 8.58031416e-02 2.13342787e+00 0.00000000e+00 0.00000000e+00

0.200000 -8.99540476e+00 3.10479866e+01 4.00438207e+01 2.18687795e+00 2.86724933e+01 1.02811781e-01 8.58034736e-02 2.18687795e+00 0.00000000e+00 0.00000000e+00

0.220000 -8.93600826e+00 3.11074063e+01 4.00438207e+01 2.24483782e+00 2.86737902e+01 1.02975131e-01 8.58032196e-02 2.24483782e+00 0.00000000e+00 0.00000000e+00

0.240000 -8.87068023e+00 3.11727579e+01 4.00438207e+01 2.30870994e+00 2.86751068e+01 1.03137605e-01 8.58035507e-02 2.30870994e+00 0.00000000e+00 0.00000000e+00

0.260000 -8.79719468e+00 3.12462671e+01 4.00438207e+01 2.38071957e+00 2.86764450e+01 1.03299263e-01 8.58032959e-02 2.38071957e+00 0.00000000e+00 0.00000000e+00

0.280000 -8.69894057e+00 3.13314700e+01 4.00438207e+01 2.46440212e+00 2.86778040e+01 1.03460168e-01 8.58036310e-02 2.46440212e+00 0.00000000e+00 0.00000000e+00

0.300000 -8.59715014e+00 3.14339274e+01 4.00438207e+01 2.56531779e+00 2.86791858e+01 1.03620398e-01 8.58033707e-02 2.56531779e+00 0.00000000e+00 0.00000000e+00

0.320000 -8.46945312e+00 3.15622551e+01 4.00438207e+01 2.69208168e+00 2.86805900e+01 1.03780044e-01 8.58034075e-02 2.69208168e+00 0.00000000e+00 0.00000000e+00

0.340000 -8.30267622e+00 3.17296469e+01 4.00438207e+01 2.85788679e+00 2.86820174e+01 1.03939219e-01 8.58034441e-02 2.85788679e+00 0.00000000e+00 0.00000000e+00

0.360000 -8.07677932e+00 3.19561306e+01 4.00438207e+01 3.08276021e+00 2.86834688e+01 1.04098080e-01 8.58034804e-02 3.08276021e+00 0.00000000e+00 0.00000000e+00

0.380000 -7.76153339e+00 3.22719371e+01 4.00438207e+01 3.39693186e+00 2.86849448e+01 1.04256857e-01 8.58035158e-02 3.39693186e+00 0.00000000e+00 0.00000000e+00

0.400000 -7.31141631e+00 3.27225991e+01 4.00438207e+01 3.84593353e+00 2.86864461e+01 1.04415910e-01 8.58035517e-02 3.84593353e+00 0.00000000e+00 0.00000000e+00

0.420000 -6.65774785e+00 3.33767874e+01 4.00438207e+01 4.49843462e+00 2.86879734e+01 1.04575847e-01 8.58035874e-02 4.49843462e+00 0.00000000e+00 0.00000000e+00

0.440000 -5.69657198e+00 3.43384343e+01 4.00438207e+01 5.45836558e+00 2.86895273e+01 1.04737749e-01 8.58036232e-02 5.45836558e+00 0.00000000e+00 0.00000000e+00

0.460000 -4.26999000e+00 3.57654692e+01 4.00438207e+01 6.88365321e+00 2.86911087e+01 1.04903620e-01 8.58036581e-02 6.88365321e+00 0.00000000e+00 0.00000000e+00

0.480000 -2.13720504e+00 3.78986779e+01 4.00438207e+01 9.01507868e+00 2.86927183e+01 1.05077286e-01 8.58036949e-02 9.01507868e+00 0.00000000e+00 0.00000000e+00

0.500000 1.06983199e+00 4.11061081e+01 4.00438207e+01 1.22206815e+01 2.86943567e+01 1.05266178e-01 8.58037316e-02 1.22206815e+01 0.00000000e+00 0.00000000e+00

0.520000 5.91490038e+00 4.59515384e+01 4.00438207e+01 1.70642249e+01 2.86960248e+01 1.05484926e-01 8.58037703e-02 1.70642249e+01 0.00000000e+00 0.00000000e+00

0.540000 1.32634589e+01 5.33004228e+01 4.00438207e+01 2.44111333e+01 2.86977232e+01 1.05762529e-01 8.58038111e-02 2.44111333e+01 0.00000000e+00 0.00000000e+00

0.560000 2.44469545e+01 6.44842375e+01 4.00438207e+01 3.55928244e+01 2.86994525e+01 1.06156719e-01 8.58038563e-02 3.55928244e+01 0.00000000e+00 0.00000000e+00

0.580000 4.15116777e+01 8.15552901e+01 4.00438207e+01 5.26614903e+01 2.87012131e+01 1.06782513e-01 8.58042009e-02 5.26614903e+01 0.00000000e+00 0.00000000e+00

0.600000 6.76358888e+01 1.07679493e+02 4.00438207e+01 7.87828141e+01 2.87030059e+01 1.07868998e-01 8.58042582e-02 7.87828141e+01 0.00000000e+00 0.00000000e+00

0.620000 1.07690921e+02 1.47734437e+02 4.00438207e+01 1.18833931e+02 2.87048314e+01 1.09870465e-01 8.58043382e-02 1.18833931e+02 0.00000000e+00 0.00000000e+00

0.640000 1.69154991e+02 2.09198135e+02 4.00438207e+01 1.80291958e+02 2.87066903e+01 1.13682160e-01 8.58044398e-02 1.80291958e+02 0.00000000e+00 0.00000000e+00

0.660000 2.63371790e+02 3.03413071e+02 4.00438207e+01 2.74497631e+02 2.87085848e+01 1.21050238e-01 8.58042889e-02 2.74497631e+02 0.00000000e+00 0.00000000e+00

0.680000 4.07211257e+02 4.47243479e+02 4.00438207e+01 4.18311827e+02 2.87105180e+01 1.35329266e-01 8.58047810e-02 4.18311827e+02 0.00000000e+00 0.00000000e+00

0.700000 6.24870372e+02 6.64916978e+02 4.00438207e+01 6.35955849e+02 2.87124994e+01 1.62824582e-01 8.58050580e-02 6.35955849e+02 0.00000000e+00 0.00000000e+00

0.720000 9.49329626e+02 9.89394698e+02 4.00438207e+01 9.60379307e+02 2.87145464e+01 2.15038406e-01 8.58054654e-02 9.60379307e+02 0.00000000e+00 0.00000000e+00

0.740000 1.42170759e+03 1.46189278e+03 4.00438207e+01 1.43277814e+03 2.87166905e+01 3.12142152e-01 8.58057741e-02 1.43277814e+03 0.00000000e+00 0.00000000e+00

0.760000 2.08823176e+03 2.12813202e+03 4.00438207e+01 2.09883931e+03 2.87189823e+01 4.87918354e-01 8.58068560e-02 2.09883931e+03 0.00000000e+00 0.00000000e+00

0.780000 2.99012181e+03 3.03038507e+03 4.00438207e+01 3.00078185e+03 2.87214934e+01 7.95919045e-01 8.58079260e-02 3.00078185e+03 0.00000000e+00 0.00000000e+00

0.800000 4.15950118e+03 4.19915970e+03 4.00438207e+01 4.16903225e+03 2.87243134e+01 1.31733061e+00 8.58093338e-02 4.16903225e+03 0.00000000e+00 0.00000000e+00

solar cell parameters deduced from calculated IV-curve:

Voc = 0.494030 Volt

Jsc = 9.43817953 mA/cm2

FF = 63.2805 %

eta = 2.9506 %

V_MPP = 0.383770 Volt

J_MPP = 7.68848514 mA/cm2

SCAPS 3.3.07 ELIS-UGent: Version scaps3307.exe, dated 27-01-2018, 10:11:02

Simulation of I-V curves

Single shot simulation # 3

Calculation started : 20-9-2021 at 1:58:16

problem definition file : c:\Program Files (x86)\Scaps3307\def\example CIGS.def

last saved: 01-09-2015 at 07:44:06

Calculation under illumination

Optical generation from internal SCAPS calculation

Illumination spectrum read from file

spectrum file :

C:\Program Files (x86)\Scaps3307\spectrum\AM1_5G 1 sun.spe last saved: 22-05-2009 at 09:59:48

Neutral density filter: ND 0.0000

Transmission of ND filter: 1.0000e+00

Power from spectrum: 100.0000 mW/cm2

Incident power on solar cell structure 100.0000 mW/cm2

Working point conditions

Temperature 300.00 K

Workpoint bias voltage 0.0000 Volt

Frequency 1.0000e+06 Hz

Voltage reference convention: voltage referred to right contact, voltage V applied to left contact

Current reference convention: current positive when entering the left contact

Power generated by the cell: = -V*I; power consumed by the cell = +V*I

v(V) jtot(mA/cm2) j_total_rec(mA/cm2) j_total_gen(mA/cm2) jbulk(mA/cm2) jifr(mA/cm2) jminor_left(mA/cm2) jminor_right(mA/cm2) j_SRH(mA/cm2) j_Radiative(mA/cm2) j_Auger(mA/cm2)

0.000000 -9.53656037e+00 3.05071190e+01 4.00438207e+01 1.74483658e+00 2.85527638e+01 9.99850404e-02 1.09533565e-01 1.74483658e+00 0.00000000e+00 0.00000000e+00

0.020000 -9.50232419e+00 3.05413623e+01 4.00438207e+01 1.77855315e+00 2.85530942e+01 1.00183539e-01 1.09531412e-01 1.77855315e+00 0.00000000e+00 0.00000000e+00

0.040000 -9.46657064e+00 3.05771169e+01 4.00438207e+01 1.81378036e+00 2.85534248e+01 1.00380410e-01 1.09531412e-01 1.81378036e+00 0.00000000e+00 0.00000000e+00

0.060000 -9.42917613e+00 3.06145126e+01 4.00438207e+01 1.85064805e+00 2.85537575e+01 1.00575692e-01 1.09531412e-01 1.85064805e+00 0.00000000e+00 0.00000000e+00

0.080000 -9.38999573e+00 3.06536943e+01 4.00438207e+01 1.88930107e+00 2.85540924e+01 1.00769423e-01 1.09531412e-01 1.88930107e+00 0.00000000e+00 0.00000000e+00

0.100000 -9.34886881e+00 3.06948226e+01 4.00438207e+01 1.92990000e+00 2.85544295e+01 1.00961645e-01 1.09531412e-01 1.92990000e+00 0.00000000e+00 0.00000000e+00

0.120000 -9.30561006e+00 3.07380828e+01 4.00438207e+01 1.97263012e+00 2.85547689e+01 1.01152397e-01 1.09531412e-01 1.97263012e+00 0.00000000e+00 0.00000000e+00

0.140000 -9.25999146e+00 3.07837031e+01 4.00438207e+01 2.01771939e+00 2.85551106e+01 1.01341724e-01 1.09531412e-01 2.01771939e+00 0.00000000e+00 0.00000000e+00

0.160000 -9.21171778e+00 3.08319786e+01 4.00438207e+01 2.06546299e+00 2.85554545e+01 1.01529670e-01 1.09531412e-01 2.06546299e+00 0.00000000e+00 0.00000000e+00

0.180000 -9.16039052e+00 3.08833078e+01 4.00438207e+01 2.11625934e+00 2.85558007e+01 1.01716283e-01 1.09531412e-01 2.11625934e+00 0.00000000e+00 0.00000000e+00

0.200000 -9.10544192e+00 3.09382585e+01 4.00438207e+01 2.17067614e+00 2.85561494e+01 1.01901612e-01 1.09531412e-01 2.17067614e+00 0.00000000e+00 0.00000000e+00

0.220000 -9.04601436e+00 3.09976883e+01 4.00438207e+01 2.22957087e+00 2.85565003e+01 1.02085710e-01 1.09531412e-01 2.22957087e+00 0.00000000e+00 0.00000000e+00

0.240000 -8.98076173e+00 3.10629433e+01 4.00438207e+01 2.29428962e+00 2.85568537e+01 1.02268634e-01 1.09531412e-01 2.29428962e+00 0.00000000e+00 0.00000000e+00

0.260000 -8.90753753e+00 3.11361700e+01 4.00438207e+01 2.36697870e+00 2.85572094e+01 1.02450446e-01 1.09531412e-01 2.36697870e+00 0.00000000e+00 0.00000000e+00

0.280000 -8.82290792e+00 3.12208020e+01 4.00438207e+01 2.45107176e+00 2.85575676e+01 1.02631216e-01 1.09531412e-01 2.45107176e+00 0.00000000e+00 0.00000000e+00

0.300000 -8.72140928e+00 3.13223029e+01 4.00438207e+01 2.55203217e+00 2.85579283e+01 1.02811023e-01 1.09531412e-01 2.55203217e+00 0.00000000e+00 0.00000000e+00

0.320000 -8.59447877e+00 3.14492353e+01 4.00438207e+01 2.67842248e+00 2.85582914e+01 1.02989966e-01 1.09531413e-01 2.67842248e+00 0.00000000e+00 0.00000000e+00

0.340000 -8.42896495e+00 3.16147504e+01 4.00438207e+01 2.84339378e+00 2.85586571e+01 1.03168168e-01 1.09531413e-01 2.84339378e+00 0.00000000e+00 0.00000000e+00

0.360000 -8.20499176e+00 3.18387242e+01 4.00438207e+01 3.06682168e+00 2.85590253e+01 1.03345801e-01 1.09531413e-01 3.06682168e+00 0.00000000e+00 0.00000000e+00

0.380000 -7.89269591e+00 3.21510195e+01 4.00438207e+01 3.37856895e+00 2.85593960e+01 1.03523121e-01 1.09531414e-01 3.37856895e+00 0.00000000e+00 0.00000000e+00

0.400000 -7.44706767e+00 3.25966458e+01 4.00438207e+01 3.82364446e+00 2.85597694e+01 1.03700537e-01 1.09531414e-01 3.82364446e+00 0.00000000e+00 0.00000000e+00

0.420000 -6.79984317e+00 3.32438662e+01 4.00438207e+01 4.47031073e+00 2.85601453e+01 1.03878754e-01 1.09531415e-01 4.47031073e+00 0.00000000e+00 0.00000000e+00

0.440000 -5.84702189e+00 3.41966804e+01 4.00438207e+01 5.42256609e+00 2.85605239e+01 1.04059054e-01 1.09531416e-01 5.42256609e+00 0.00000000e+00 0.00000000e+00

0.460000 -4.42990657e+00 3.56137846e+01 4.00438207e+01 6.83910430e+00 2.85609051e+01 1.04243845e-01 1.09531419e-01 6.83910430e+00 0.00000000e+00 0.00000000e+00

0.480000 -2.30633292e+00 3.77373423e+01 4.00438207e+01 8.96208415e+00 2.85612889e+01 1.04437772e-01 1.09531421e-01 8.96208415e+00 0.00000000e+00 0.00000000e+00

0.500000 8.93328763e-01 4.09369876e+01 4.00438207e+01 1.21611308e+01 2.85616754e+01 1.04649936e-01 1.09531425e-01 1.21611308e+01 0.00000000e+00 0.00000000e+00

0.520000 5.73422015e+00 4.57778690e+01 4.00438207e+01 1.70013748e+01 2.85620645e+01 1.04898362e-01 1.09531430e-01 1.70013748e+01 0.00000000e+00 0.00000000e+00

0.540000 1.30831373e+01 5.31268067e+01 4.00438207e+01 2.43496001e+01 2.85624561e+01 1.05218952e-01 1.09531440e-01 2.43496001e+01 0.00000000e+00 0.00000000e+00

0.560000 2.42747078e+01 6.43185585e+01 4.00438207e+01 3.55404934e+01 2.85628502e+01 1.05683461e-01 1.09531453e-01 3.55404934e+01 0.00000000e+00 0.00000000e+00

0.580000 4.13721628e+01 8.14158229e+01 4.00438207e+01 5.26366093e+01 2.85632445e+01 1.06435367e-01 1.09533673e-01 5.26366093e+01 0.00000000e+00 0.00000000e+00

0.600000 6.75694388e+01 1.07613084e+02 4.00438207e+01 7.88321457e+01 2.85636432e+01 1.07761437e-01 1.09533702e-01 7.88321457e+01 0.00000000e+00 0.00000000e+00

0.620000 1.07813432e+02 1.47857003e+02 4.00438207e+01 1.19073191e+02 2.85640450e+01 1.10232681e-01 1.09533747e-01 1.19073191e+02 0.00000000e+00 0.00000000e+00

0.640000 1.69737643e+02 2.09780835e+02 4.00438207e+01 1.80991873e+02 2.85644476e+01 1.14980098e-01 1.09533817e-01 1.80991873e+02 0.00000000e+00 0.00000000e+00

0.660000 2.65022037e+02 3.05063340e+02 4.00438207e+01 2.76264731e+02 2.85648504e+01 1.24224884e-01 1.09533925e-01 2.76264731e+02 0.00000000e+00 0.00000000e+00

0.680000 4.11246223e+02 4.51278121e+02 4.00438207e+01 4.22461058e+02 2.85652550e+01 1.42275056e-01 1.09532152e-01 4.22461058e+02 0.00000000e+00 0.00000000e+00

0.700000 6.34047698e+02 6.74094557e+02 4.00438207e+01 6.45242033e+02 2.85656542e+01 1.77335663e-01 1.09534137e-01 6.45242033e+02 0.00000000e+00 0.00000000e+00

0.720000 9.69163080e+02 1.00923096e+03 4.00438207e+01 9.80310724e+02 2.85660513e+01 2.44646337e-01 1.09534522e-01 9.80310724e+02 0.00000000e+00 0.00000000e+00

0.740000 1.46270580e+03 1.50272716e+03 4.00438207e+01 1.47367960e+03 2.85664429e+01 3.71581228e-01 1.09534922e-01 1.47367960e+03 0.00000000e+00 0.00000000e+00

0.760000 2.16768299e+03 2.20752030e+03 4.00438207e+01 2.17823864e+03 2.85668280e+01 6.05293115e-01 1.09534566e-01 2.17823864e+03 0.00000000e+00 0.00000000e+00

0.780000 3.13465965e+03 3.17512055e+03 4.00438207e+01 3.14542070e+03 2.85672033e+01 1.02311779e+00 1.09535822e-01 3.14542070e+03 0.00000000e+00 0.00000000e+00

0.800000 4.40496739e+03 4.44542116e+03 4.00438207e+01 4.41499746e+03 2.85675710e+01 1.74659518e+00 1.09537506e-01 4.41499746e+03 0.00000000e+00 0.00000000e+00

solar cell parameters deduced from calculated IV-curve:

Voc = 0.495043 Volt

Jsc = 9.53656037 mA/cm2

FF = 63.5740 %

eta = 3.0013 %

V_MPP = 0.384941 Volt

J_MPP = 7.79686198 mA/cm2

# **Ag_2_S QDs (Eg = 1.82 eV)**

SCAPS 3.3.07 ELIS-UGent: Version scaps3307.exe, dated 27-01-2018, 10:11:02

Simulation of I-V curves

Single shot simulation # 1

Calculation started : 20-9-2021 at 2:06:53

problem definition file : c:\Program Files (x86)\Scaps3307\def\example CIGS.def

last saved: 01-09-2015 at 07:44:06

Calculation under illumination

Optical generation from internal SCAPS calculation

Illumination spectrum read from file

spectrum file :

C:\Program Files (x86)\Scaps3307\spectrum\AM1_5G 1 sun.spe last saved: 22-05-2009 at 09:59:48

Neutral density filter: ND 0.0000

Transmission of ND filter: 1.0000e+00

Power from spectrum: 100.0000 mW/cm2

Incident power on solar cell structure 100.0000 mW/cm2

Working point conditions

Temperature 300.00 K

Workpoint bias voltage 0.0000 Volt

Frequency 1.0000e+06 Hz

Voltage reference convention: voltage referred to right contact, voltage V applied to left contact

Current reference convention: current positive when entering the left contact

Power generated by the cell: = -V*I; power consumed by the cell = +V*I

v(V) jtot(mA/cm2) j_total_rec(mA/cm2) j_total_gen(mA/cm2) jbulk(mA/cm2) jifr(mA/cm2) jminor_left(mA/cm2) jminor_right(mA/cm2) j_SRH(mA/cm2) j_Radiative(mA/cm2) j_Auger(mA/cm2)

0.000000 -2.97869966e+01 8.57245792e+00 3.83586519e+01 4.92993873e+00 3.34866936e+00 2.11091740e-01 8.27580885e-02 4.92993873e+00 0.00000000e+00 0.00000000e+00

0.020000 -2.96636529e+01 8.69295601e+00 3.83586519e+01 5.02397790e+00 3.37473707e+00 2.11477838e-01 8.27632092e-02 5.02397790e+00 0.00000000e+00 0.00000000e+00

0.040000 -2.95385122e+01 8.81810681e+00 3.83586519e+01 5.12219437e+00 3.40128179e+00 2.11862359e-01 8.27682885e-02 5.12219437e+00 0.00000000e+00 0.00000000e+00

0.060000 -2.94084155e+01 8.94822777e+00 3.83586519e+01 5.22478273e+00 3.42842673e+00 2.12244802e-01 8.27735112e-02 5.22478273e+00 0.00000000e+00 0.00000000e+00

0.080000 -2.92729833e+01 9.08368724e+00 3.83586519e+01 5.33208623e+00 3.45619686e+00 2.12625256e-01 8.27788861e-02 5.33208623e+00 0.00000000e+00 0.00000000e+00

0.100000 -2.91317967e+01 9.22490224e+00 3.83586519e+01 5.44449574e+00 3.48461826e+00 2.13003814e-01 8.27844223e-02 5.44449574e+00 0.00000000e+00 0.00000000e+00

0.120000 -2.89843824e+01 9.37234951e+00 3.83586519e+01 5.56246018e+00 3.51371863e+00 2.13380573e-01 8.27901312e-02 5.56246018e+00 0.00000000e+00 0.00000000e+00

0.140000 -2.88301837e+01 9.52658179e+00 3.83586519e+01 5.68650277e+00 3.54352736e+00 2.13755634e-01 8.27960236e-02 5.68650277e+00 0.00000000e+00 0.00000000e+00

0.160000 -2.86685480e+01 9.68825675e+00 3.83586519e+01 5.81724976e+00 3.57407578e+00 2.14129103e-01 8.28021124e-02 5.81724976e+00 0.00000000e+00 0.00000000e+00

0.180000 -2.84986628e+01 9.85818257e+00 3.83586519e+01 5.95547589e+00 3.60539718e+00 2.14501090e-01 8.28084112e-02 5.95547589e+00 0.00000000e+00 0.00000000e+00

0.200000 -2.83194974e+01 1.00373921e+01 3.83586519e+01 6.10217842e+00 3.63752706e+00 2.14871712e-01 8.28149357e-02 6.10217842e+00 0.00000000e+00 0.00000000e+00

0.220000 -2.81296786e+01 1.02272682e+01 3.83586519e+01 6.25870209e+00 3.67050329e+00 2.15241091e-01 8.28217018e-02 6.25870209e+00 0.00000000e+00 0.00000000e+00

0.240000 -2.79272484e+01 1.04297504e+01 3.83586519e+01 6.42694608e+00 3.70436624e+00 2.15609357e-01 8.28287304e-02 6.42694608e+00 0.00000000e+00 0.00000000e+00

0.260000 -2.77093846e+01 1.06476712e+01 3.83586519e+01 6.60969952e+00 3.73915903e+00 2.15976651e-01 8.28360417e-02 6.60969952e+00 0.00000000e+00 0.00000000e+00

0.280000 -2.74718381e+01 1.08852845e+01 3.83586519e+01 6.81117002e+00 3.77492771e+00 2.16343126e-01 8.28436599e-02 6.81117002e+00 0.00000000e+00 0.00000000e+00

0.300000 -2.72081409e+01 1.11490642e+01 3.83586519e+01 7.03778218e+00 3.81172143e+00 2.16708955e-01 8.28516112e-02 7.03778218e+00 0.00000000e+00 0.00000000e+00

0.320000 -2.69084239e+01 1.14488585e+01 3.83586519e+01 7.29933156e+00 3.84959267e+00 2.17074334e-01 8.28599266e-02 7.29933156e+00 0.00000000e+00 0.00000000e+00

0.340000 -2.65565090e+01 1.17993878e+01 3.83586519e+01 7.61047296e+00 3.88860687e+00 2.17439283e-01 8.28687028e-02 7.61047296e+00 0.00000000e+00 0.00000000e+00

0.360000 -2.61333044e+01 1.22227869e+01 3.83586519e+01 7.99330269e+00 3.92880186e+00 2.17804549e-01 8.28778465e-02 7.99330269e+00 0.00000000e+00 0.00000000e+00

0.380000 -2.56054710e+01 1.27508628e+01 3.83586519e+01 8.47955146e+00 3.97025360e+00 2.18170279e-01 8.28874717e-02 8.47955146e+00 0.00000000e+00 0.00000000e+00

0.400000 -2.49274161e+01 1.34291742e+01 3.83586519e+01 9.11471024e+00 4.01302937e+00 2.18536995e-01 8.28976284e-02 9.11471024e+00 0.00000000e+00 0.00000000e+00

0.420000 -2.40351992e+01 1.43216783e+01 3.83586519e+01 9.96266431e+00 4.05720014e+00 2.18905441e-01 8.29083736e-02 9.96266431e+00 0.00000000e+00 0.00000000e+00

0.440000 -2.28406645e+01 1.55164912e+01 3.83586519e+01 1.11114543e+01 4.10284043e+00 2.19276732e-01 8.29197768e-02 1.11114543e+01 0.00000000e+00 0.00000000e+00

0.460000 -2.12238924e+01 1.71320239e+01 3.83586519e+01 1.26794165e+01 4.15002307e+00 2.19652384e-01 8.29319698e-02 1.26794165e+01 0.00000000e+00 0.00000000e+00

0.480000 -1.90328170e+01 1.93238292e+01 3.83586519e+01 1.48220110e+01 4.19883772e+00 2.20035650e-01 8.29449080e-02 1.48220110e+01 0.00000000e+00 0.00000000e+00

0.500000 -1.60709094e+01 2.22864897e+01 3.83586519e+01 1.77337360e+01 4.24936408e+00 2.20430792e-01 8.29587659e-02 1.77337360e+01 0.00000000e+00 0.00000000e+00

0.520000 -1.21032035e+01 2.62543438e+01 3.83586519e+01 2.16488438e+01 4.30168105e+00 2.20845292e-01 8.29736782e-02 2.16488438e+01 0.00000000e+00 0.00000000e+00

0.540000 -6.86192423e+00 3.14974871e+01 3.83586519e+01 2.68373116e+01 4.35589342e+00 2.21292353e-01 8.29896921e-02 2.68373116e+01 0.00000000e+00 0.00000000e+00

0.560000 -2.82417554e-02 3.83311728e+01 3.83586519e+01 3.36143196e+01 4.41205203e+00 2.21794058e-01 8.30070753e-02 3.36143196e+01 0.00000000e+00 0.00000000e+00

0.580000 8.79040501e+00 4.71503360e+01 3.83586519e+01 4.23746553e+01 4.47026362e+00 2.22391140e-01 8.30259041e-02 4.23746553e+01 0.00000000e+00 0.00000000e+00

0.600000 2.01769480e+01 5.85372973e+01 3.83586519e+01 5.37004876e+01 4.53060586e+00 2.23157479e-01 8.30463685e-02 5.37004876e+01 0.00000000e+00 0.00000000e+00

0.620000 3.50892905e+01 7.34484042e+01 3.83586519e+01 6.85479637e+01 4.59314253e+00 2.24229258e-01 8.30686786e-02 6.85479637e+01 0.00000000e+00 0.00000000e+00

0.640000 5.50721062e+01 9.34312248e+01 3.83586519e+01 8.84642498e+01 4.65802589e+00 2.25856202e-01 8.30929007e-02 8.84642498e+01 0.00000000e+00 0.00000000e+00

0.660000 8.26216239e+01 1.20982314e+02 3.83586519e+01 1.15945294e+02 4.72540967e+00 2.28491196e-01 8.31191474e-02 1.15945294e+02 0.00000000e+00 0.00000000e+00

0.680000 1.21696751e+02 1.60057106e+02 3.83586519e+01 1.54945549e+02 4.79546047e+00 2.32948467e-01 8.31474952e-02 1.54945549e+02 0.00000000e+00 0.00000000e+00

0.700000 1.78311012e+02 2.16670879e+02 3.83586519e+01 2.11478470e+02 4.86856280e+00 2.40668373e-01 8.31778058e-02 2.11478470e+02 0.00000000e+00 0.00000000e+00

0.720000 2.61564363e+02 2.99923746e+02 3.83586519e+01 2.94641107e+02 4.94526822e+00 2.54161482e-01 8.32097915e-02 2.94641107e+02 0.00000000e+00 0.00000000e+00

0.740000 3.84865495e+02 4.23224556e+02 3.83586519e+01 4.17837214e+02 5.02637754e+00 2.77721116e-01 8.32429914e-02 4.17837214e+02 0.00000000e+00 0.00000000e+00

0.760000 5.67266904e+02 6.05626160e+02 3.83586519e+01 6.00111394e+02 5.11296450e+00 3.18524519e-01 8.32767822e-02 6.00111394e+02 0.00000000e+00 0.00000000e+00

0.780000 8.34430770e+02 8.72792698e+02 3.83586519e+01 8.67114793e+02 5.20634058e+00 3.88254096e-01 8.33104351e-02 8.67114793e+02 0.00000000e+00 0.00000000e+00

0.800000 1.21873813e+03 1.25709078e+03 3.83586519e+01 1.25119404e+03 5.30802549e+00 5.05376392e-01 8.33432153e-02 1.25119404e+03 0.00000000e+00 0.00000000e+00

solar cell parameters deduced from calculated IV-curve:

Voc = 0.560072 Volt

Jsc = 29.78699657 mA/cm2

FF = 60.5510 %

eta = 10.1016 %

V_MPP = 0.425566 Volt

J_MPP = 23.73693943 mA/cm2

SCAPS 3.3.07 ELIS-UGent: Version scaps3307.exe, dated 27-01-2018, 10:11:02

Simulation of I-V curves

Single shot simulation # 2

Calculation started : 20-9-2021 at 2:07:23

problem definition file : c:\Program Files (x86)\Scaps3307\def\example CIGS.def

last saved: 01-09-2015 at 07:44:06

Calculation under illumination

Optical generation from internal SCAPS calculation

Illumination spectrum read from file

spectrum file :

C:\Program Files (x86)\Scaps3307\spectrum\AM1_5G 1 sun.spe last saved: 22-05-2009 at 09:59:48

Neutral density filter: ND 0.0000

Transmission of ND filter: 1.0000e+00

Power from spectrum: 100.0000 mW/cm2

Incident power on solar cell structure 100.0000 mW/cm2

Working point conditions

Temperature 300.00 K

Workpoint bias voltage 0.0000 Volt

Frequency 1.0000e+06 Hz

Voltage reference convention: voltage referred to right contact, voltage V applied to left contact

Current reference convention: current positive when entering the left contact

Power generated by the cell: = -V*I; power consumed by the cell = +V*I

v(V) jtot(mA/cm2) j_total_rec(mA/cm2) j_total_gen(mA/cm2) jbulk(mA/cm2) jifr(mA/cm2) jminor_left(mA/cm2) jminor_right(mA/cm2) j_SRH(mA/cm2) j_Radiative(mA/cm2) j_Auger(mA/cm2)

0.000000 -3.00535855e+01 8.30496011e+00 3.83586519e+01 4.73185799e+00 3.27920201e+00 2.07797226e-01 8.61028765e-02 4.73185799e+00 0.00000000e+00 0.00000000e+00

0.020000 -2.99447973e+01 8.41329151e+00 3.83586519e+01 4.82152434e+00 3.29746949e+00 2.08194749e-01 8.61029234e-02 4.82152434e+00 0.00000000e+00 0.00000000e+00

0.040000 -2.98322265e+01 8.52589951e+00 3.83586519e+01 4.91515399e+00 3.31605258e+00 2.08589966e-01 8.61029694e-02 4.91515399e+00 0.00000000e+00 0.00000000e+00

0.060000 -2.97150648e+01 8.64309730e+00 3.83586519e+01 5.01305140e+00 3.33495992e+00 2.08982972e-01 8.61030147e-02 5.01305140e+00 0.00000000e+00 0.00000000e+00

0.080000 -2.95929624e+01 8.76523510e+00 3.83586519e+01 5.11555771e+00 3.35420049e+00 2.09373846e-01 8.61030595e-02 5.11555771e+00 0.00000000e+00 0.00000000e+00

0.100000 -2.94655193e+01 8.89270920e+00 3.83586519e+01 5.22305981e+00 3.37378362e+00 2.09762672e-01 8.61031038e-02 5.22305981e+00 0.00000000e+00 0.00000000e+00

0.120000 -2.93322897e+01 9.02596865e+00 3.83586519e+01 5.33599701e+00 3.39371896e+00 2.10149536e-01 8.61031473e-02 5.33599701e+00 0.00000000e+00 0.00000000e+00

0.140000 -2.91927559e+01 9.16552441e+00 3.83586519e+01 5.45487018e+00 3.41401651e+00 2.10534531e-01 8.61031905e-02 5.45487018e+00 0.00000000e+00 0.00000000e+00

0.160000 -2.90463396e+01 9.31196747e+00 3.83586519e+01 5.58025983e+00 3.43468665e+00 2.10917753e-01 8.61032331e-02 5.58025983e+00 0.00000000e+00 0.00000000e+00

0.180000 -2.88923411e+01 9.46599920e+00 3.83586519e+01 5.71285649e+00 3.45574013e+00 2.11299302e-01 8.61032752e-02 5.71285649e+00 0.00000000e+00 0.00000000e+00

0.200000 -2.87298847e+01 9.62847608e+00 3.83586519e+01 5.85350537e+00 3.47718810e+00 2.11679287e-01 8.61033167e-02 5.85350537e+00 0.00000000e+00 0.00000000e+00

0.220000 -2.85579072e+01 9.80048140e+00 3.83586519e+01 6.00327810e+00 3.49904212e+00 2.12057820e-01 8.61033577e-02 6.00327810e+00 0.00000000e+00 0.00000000e+00

0.240000 -2.83749617e+01 9.98345407e+00 3.83586519e+01 6.16360145e+00 3.52131420e+00 2.12435026e-01 8.61033983e-02 6.16360145e+00 0.00000000e+00 0.00000000e+00

0.260000 -2.81790223e+01 1.01794178e+01 3.83586519e+01 6.33648660e+00 3.54401677e+00 2.12811037e-01 8.61034383e-02 6.33648660e+00 0.00000000e+00 0.00000000e+00

0.280000 -2.79670939e+01 1.03913741e+01 3.83586519e+01 6.52492183e+00 3.56716277e+00 2.13186000e-01 8.61034779e-02 6.52492183e+00 0.00000000e+00 0.00000000e+00

0.300000 -2.77345490e+01 1.06239611e+01 3.83586519e+01 6.73353190e+00 3.59076559e+00 2.13560081e-01 8.61035165e-02 6.73353190e+00 0.00000000e+00 0.00000000e+00

0.320000 -2.74740325e+01 1.08845318e+01 3.83586519e+01 6.96965567e+00 3.61483915e+00 2.13933472e-01 8.61035550e-02 6.96965567e+00 0.00000000e+00 0.00000000e+00

0.340000 -2.71737781e+01 1.11848395e+01 3.83586519e+01 7.24503157e+00 3.63939789e+00 2.14306407e-01 8.61035932e-02 7.24503157e+00 0.00000000e+00 0.00000000e+00

0.360000 -2.68151009e+01 1.15435675e+01 3.83586519e+01 7.57832794e+00 3.66445676e+00 2.14679188e-01 8.61036301e-02 7.57832794e+00 0.00000000e+00 0.00000000e+00

0.380000 -2.63686778e+01 1.19900371e+01 3.83586519e+01 7.99884994e+00 3.69003126e+00 2.15052222e-01 8.61036675e-02 7.99884994e+00 0.00000000e+00 0.00000000e+00

0.400000 -2.57891554e+01 1.25695991e+01 3.83586519e+01 8.55193185e+00 3.71613744e+00 2.15426099e-01 8.61037044e-02 8.55193185e+00 0.00000000e+00 0.00000000e+00

0.420000 -2.50072144e+01 1.33515696e+01 3.83586519e+01 9.30687234e+00 3.74279183e+00 2.15801730e-01 8.61037413e-02 9.30687234e+00 0.00000000e+00 0.00000000e+00

0.440000 -2.39175924e+01 1.44412066e+01 3.83586519e+01 1.03689108e+01 3.77001147e+00 2.16180598e-01 8.61037782e-02 1.03689108e+01 0.00000000e+00 0.00000000e+00

0.460000 -2.23606175e+01 1.59981758e+01 3.83586519e+01 1.18976929e+01 3.79781379e+00 2.16565266e-01 8.61038151e-02 1.18976929e+01 0.00000000e+00 0.00000000e+00

0.480000 -2.00935422e+01 1.82652154e+01 3.83586519e+01 1.41359347e+01 3.82621646e+00 2.16960338e-01 8.61038523e-02 1.41359347e+01 0.00000000e+00 0.00000000e+00

0.500000 -1.67461466e+01 2.16125330e+01 3.83586519e+01 1.74538176e+01 3.85523717e+00 2.17374371e-01 8.61038896e-02 1.74538176e+01 0.00000000e+00 0.00000000e+00

0.520000 -1.17521151e+01 2.66064301e+01 3.83586519e+01 2.24176093e+01 3.88489324e+00 2.17823642e-01 8.61039292e-02 2.24176093e+01 0.00000000e+00 0.00000000e+00

0.540000 -4.24292419e+00 3.41154994e+01 3.83586519e+01 2.98958549e+01 3.91520094e+00 2.18339608e-01 8.61039722e-02 2.98958549e+01 0.00000000e+00 0.00000000e+00

0.560000 7.11705651e+00 4.54752968e+01 3.83586519e+01 4.12240346e+01 3.94617447e+00 2.18983709e-01 8.61040176e-02 4.12240346e+01 0.00000000e+00 0.00000000e+00

0.580000 2.43851103e+01 6.27435767e+01 3.83586519e+01 5.84597718e+01 3.97782419e+00 2.19876624e-01 8.61040696e-02 5.84597718e+01 0.00000000e+00 0.00000000e+00

0.600000 5.07352579e+01 8.90937166e+01 3.83586519e+01 8.47762022e+01 4.01015423e+00 2.21256133e-01 8.61041296e-02 8.47762022e+01 0.00000000e+00 0.00000000e+00

0.620000 9.10552110e+01 1.29413620e+02 3.83586519e+01 1.25060767e+02 4.04315839e+00 2.23589995e-01 8.61042072e-02 1.25060767e+02 0.00000000e+00 0.00000000e+00

0.640000 1.52837422e+02 1.91195535e+02 3.83586519e+01 1.86804822e+02 4.07681407e+00 2.27794278e-01 8.61043111e-02 1.86804822e+02 0.00000000e+00 0.00000000e+00

0.660000 2.47444594e+02 2.85801087e+02 3.83586519e+01 2.81368262e+02 4.11107360e+00 2.35647166e-01 8.61044519e-02 2.81368262e+02 0.00000000e+00 0.00000000e+00

0.680000 3.91770653e+02 4.30118717e+02 3.83586519e+01 4.25636207e+02 4.14585297e+00 2.50551692e-01 8.61046524e-02 4.25636207e+02 0.00000000e+00 0.00000000e+00

0.700000 6.10042126e+02 6.48403352e+02 3.83586519e+01 6.43857345e+02 4.18101779e+00 2.78885170e-01 8.61049400e-02 6.43857345e+02 0.00000000e+00 0.00000000e+00

0.720000 9.35260215e+02 9.73639351e+02 3.83586519e+01 9.69004620e+02 4.21637581e+00 3.32249633e-01 8.61053536e-02 9.69004620e+02 0.00000000e+00 0.00000000e+00

0.740000 1.40855290e+03 1.44705012e+03 3.83586519e+01 1.44228139e+03 4.25167540e+00 4.30947903e-01 8.61059428e-02 1.44228139e+03 0.00000000e+00 0.00000000e+00

0.760000 2.07611542e+03 2.11433230e+03 3.83586519e+01 2.10935063e+03 4.28664614e+00 6.08912076e-01 8.61067570e-02 2.10935063e+03 0.00000000e+00 0.00000000e+00

0.780000 2.97912252e+03 3.01769895e+03 3.83586519e+01 3.01237198e+03 4.32100479e+00 9.19858822e-01 8.61078551e-02 3.01237198e+03 0.00000000e+00 0.00000000e+00

0.800000 4.14959845e+03 4.18757320e+03 3.83586519e+01 4.18168739e+03 4.35459062e+00 1.44510339e+00 8.61092630e-02 4.18168739e+03 0.00000000e+00 0.00000000e+00

solar cell parameters deduced from calculated IV-curve:

Voc = 0.548293 Volt

Jsc = 30.05358546 mA/cm2

FF = 63.9698 %

eta = 10.5411 %

V_MPP = 0.432246 Volt

J_MPP = 24.38669244 mA/cm2

SCAPS 3.3.07 ELIS-UGent: Version scaps3307.exe, dated 27-01-2018, 10:11:02

Simulation of I-V curves

Single shot simulation # 3

Calculation started : 20-9-2021 at 2:07:47

problem definition file : c:\Program Files (x86)\Scaps3307\def\example CIGS.def

last saved: 01-09-2015 at 07:44:06

Calculation under illumination

Optical generation from internal SCAPS calculation

Illumination spectrum read from file

spectrum file :

C:\Program Files (x86)\Scaps3307\spectrum\AM1_5G 1 sun.spe last saved: 22-05-2009 at 09:59:48

Neutral density filter: ND 0.0000

Transmission of ND filter: 1.0000e+00

Power from spectrum: 100.0000 mW/cm2

Incident power on solar cell structure 100.0000 mW/cm2

Working point conditions

Temperature 300.00 K

Workpoint bias voltage 0.0000 Volt

Frequency 1.0000e+06 Hz

Voltage reference convention: voltage referred to right contact, voltage V applied to left contact

Current reference convention: current positive when entering the left contact

Power generated by the cell: = -V*I; power consumed by the cell = +V*I

v(V) jtot(mA/cm2) j_total_rec(mA/cm2) j_total_gen(mA/cm2) jbulk(mA/cm2) jifr(mA/cm2) jminor_left(mA/cm2) jminor_right(mA/cm2) j_SRH(mA/cm2) j_Radiative(mA/cm2) j_Auger(mA/cm2)

0.000000 -3.14659536e+01 6.89252095e+00 3.83586519e+01 4.97834360e+00 1.59671225e+00 2.05203082e-01 1.12262009e-01 4.97834360e+00 0.00000000e+00 0.00000000e+00

0.020000 -3.13671377e+01 6.99156094e+00 3.83586519e+01 5.07480221e+00 1.59884485e+00 2.05651882e-01 1.12262010e-01 5.07480221e+00 0.00000000e+00 0.00000000e+00

0.040000 -3.12637376e+01 7.09497289e+00 3.83586519e+01 5.17562329e+00 1.60098990e+00 2.06097685e-01 1.12262010e-01 5.17562329e+00 0.00000000e+00 0.00000000e+00

0.060000 -3.11556360e+01 7.20308728e+00 3.83586519e+01 5.28113712e+00 1.60314756e+00 2.06540592e-01 1.12262010e-01 5.28113712e+00 0.00000000e+00 0.00000000e+00

0.080000 -3.10424659e+01 7.31627113e+00 3.83586519e+01 5.39171048e+00 1.60531795e+00 2.06980691e-01 1.12262010e-01 5.39171048e+00 0.00000000e+00 0.00000000e+00

0.100000 -3.09238103e+01 7.43494170e+00 3.83586519e+01 5.50776041e+00 1.60750121e+00 2.07418072e-01 1.12262010e-01 5.50776041e+00 0.00000000e+00 0.00000000e+00

0.120000 -3.07991901e+01 7.55957815e+00 3.83586519e+01 5.62976584e+00 1.60969747e+00 2.07852832e-01 1.12262010e-01 5.62976584e+00 0.00000000e+00 0.00000000e+00

0.140000 -3.06680545e+01 7.69073137e+00 3.83586519e+01 5.75827741e+00 1.61190688e+00 2.08285070e-01 1.12262010e-01 5.75827741e+00 0.00000000e+00 0.00000000e+00

0.160000 -3.05297674e+01 7.82903773e+00 3.83586519e+01 5.89393125e+00 1.61412957e+00 2.08714890e-01 1.12262010e-01 5.89393125e+00 0.00000000e+00 0.00000000e+00

0.180000 -3.03835814e+01 7.97524480e+00 3.83586519e+01 6.03747469e+00 1.61636570e+00 2.09142402e-01 1.12262011e-01 6.03747469e+00 0.00000000e+00 0.00000000e+00

0.200000 -3.02285953e+01 8.13025381e+00 3.83586519e+01 6.18980868e+00 1.61861540e+00 2.09567722e-01 1.12262011e-01 6.18980868e+00 0.00000000e+00 0.00000000e+00

0.220000 -3.00636897e+01 8.29518460e+00 3.83586519e+01 6.35205280e+00 1.62087882e+00 2.09990974e-01 1.12262011e-01 6.35205280e+00 0.00000000e+00 0.00000000e+00

0.240000 -2.98874162e+01 8.47148556e+00 3.83586519e+01 6.52565514e+00 1.62315612e+00 2.10412289e-01 1.12262011e-01 6.52565514e+00 0.00000000e+00 0.00000000e+00

0.260000 -2.96977947e+01 8.66113720e+00 3.83586519e+01 6.71259594e+00 1.62544744e+00 2.10831810e-01 1.12262012e-01 6.71259594e+00 0.00000000e+00 0.00000000e+00

0.280000 -2.94919426e+01 8.86702199e+00 3.83586519e+01 6.91575735e+00 1.62775294e+00 2.11249694e-01 1.12262012e-01 6.91575735e+00 0.00000000e+00 0.00000000e+00

0.300000 -2.92654402e+01 9.09355928e+00 3.83586519e+01 7.13955838e+00 1.63007277e+00 2.11666114e-01 1.12262012e-01 7.13955838e+00 0.00000000e+00 0.00000000e+00

0.320000 -2.90112813e+01 9.34775469e+00 3.83586519e+01 7.39100429e+00 1.63240711e+00 2.12081276e-01 1.12262013e-01 7.39100429e+00 0.00000000e+00 0.00000000e+00

0.340000 -2.87181926e+01 9.64088042e+00 3.83586519e+01 7.68136689e+00 1.63475609e+00 2.12495427e-01 1.12262013e-01 7.68136689e+00 0.00000000e+00 0.00000000e+00

0.360000 -2.83680649e+01 9.99104384e+00 3.83586519e+01 8.02875306e+00 1.63711989e+00 2.12908885e-01 1.12262013e-01 8.02875306e+00 0.00000000e+00 0.00000000e+00

0.380000 -2.79322039e+01 1.04269367e+01 3.83586519e+01 8.46185392e+00 1.63949865e+00 2.13322087e-01 1.12262013e-01 8.46185392e+00 0.00000000e+00 0.00000000e+00

0.400000 -2.73659274e+01 1.09932381e+01 3.83586519e+01 9.02534787e+00 1.64189254e+00 2.13735676e-01 1.12262014e-01 9.02534787e+00 0.00000000e+00 0.00000000e+00

0.420000 -2.66005660e+01 1.17586141e+01 3.83586519e+01 9.78789971e+00 1.64430170e+00 2.14150659e-01 1.12262015e-01 9.78789971e+00 0.00000000e+00 0.00000000e+00

0.440000 -2.55312381e+01 1.28279410e+01 3.83586519e+01 1.08543840e+01 1.64672626e+00 2.14568721e-01 1.12262016e-01 1.08543840e+01 0.00000000e+00 0.00000000e+00

0.460000 -2.39980135e+01 1.43611415e+01 3.83586519e+01 1.23847203e+01 1.64916634e+00 2.14992826e-01 1.12262018e-01 1.23847203e+01 0.00000000e+00 0.00000000e+00

0.480000 -2.17570476e+01 1.66020499e+01 3.83586519e+01 1.46227374e+01 1.65162202e+00 2.15428397e-01 1.12262021e-01 1.46227374e+01 0.00000000e+00 0.00000000e+00

0.500000 -1.84363912e+01 1.99226002e+01 3.83586519e+01 1.79403592e+01 1.65409331e+00 2.15885654e-01 1.12262024e-01 1.79403592e+01 0.00000000e+00 0.00000000e+00

0.520000 -1.34678216e+01 2.48909972e+01 3.83586519e+01 2.29057708e+01 1.65658015e+00 2.16384257e-01 1.12262030e-01 2.29057708e+01 0.00000000e+00 0.00000000e+00

0.540000 -5.98064583e+00 3.23779219e+01 3.83586519e+01 3.03896150e+01 1.65908236e+00 2.16962545e-01 1.12262038e-01 3.03896150e+01 0.00000000e+00 0.00000000e+00

0.560000 5.36461619e+00 4.37228878e+01 3.83586519e+01 4.17313303e+01 1.66159952e+00 2.17695952e-01 1.12262052e-01 4.17313303e+01 0.00000000e+00 0.00000000e+00

0.580000 2.26363956e+01 6.09945290e+01 3.83586519e+01 5.89994024e+01 1.66413089e+00 2.18733644e-01 1.12262072e-01 5.89994024e+01 0.00000000e+00 0.00000000e+00

0.600000 4.90367844e+01 8.73952452e+01 3.83586519e+01 8.53959369e+01 1.66667520e+00 2.20370940e-01 1.12262103e-01 8.53959369e+01 0.00000000e+00 0.00000000e+00

0.620000 8.95241185e+01 1.27882540e+02 3.83586519e+01 1.25877854e+02 1.66923051e+00 2.23192601e-01 1.12262149e-01 1.25877854e+02 0.00000000e+00 0.00000000e+00

0.640000 1.51746999e+02 1.90105151e+02 3.83586519e+01 1.88092745e+02 1.67179343e+00 2.28350851e-01 1.12262221e-01 1.88092745e+02 0.00000000e+00 0.00000000e+00

0.660000 2.47406993e+02 2.85763582e+02 3.83586519e+01 2.83738861e+02 1.67435889e+00 2.38100109e-01 1.12262332e-01 2.83738861e+02 0.00000000e+00 0.00000000e+00

0.680000 3.94112574e+02 4.32460709e+02 3.83586519e+01 4.30414727e+02 1.67691917e+00 2.56799739e-01 1.12262502e-01 4.30414727e+02 0.00000000e+00 0.00000000e+00

0.700000 6.17540908e+02 6.55902307e+02 3.83586519e+01 6.53817848e+02 1.67946294e+00 2.92733234e-01 1.12262761e-01 6.53817848e+02 0.00000000e+00 0.00000000e+00

0.720000 9.53462938e+02 9.91844488e+02 3.83586519e+01 9.89688997e+02 1.68197462e+00 3.61253495e-01 1.12263151e-01 9.89688997e+02 0.00000000e+00 0.00000000e+00

0.740000 1.44801746e+03 1.48635426e+03 3.83586519e+01 1.48406767e+03 1.68443458e+00 4.89890345e-01 1.12263724e-01 1.48406767e+03 0.00000000e+00 0.00000000e+00

0.760000 2.15421197e+03 2.19236723e+03 3.83586519e+01 2.18984215e+03 1.68682062e+00 7.25994888e-01 1.12264541e-01 2.18984215e+03 0.00000000e+00 0.00000000e+00

0.780000 3.12257598e+03 3.16134848e+03 3.83586519e+01 3.15839997e+03 1.68911093e+00 1.14713571e+00 1.12265668e-01 3.15839997e+03 0.00000000e+00 0.00000000e+00

0.800000 4.39433976e+03 4.43310752e+03 3.83586519e+01 4.42942888e+03 1.69129252e+00 1.87508122e+00 1.12267142e-01 4.42942888e+03 0.00000000e+00 0.00000000e+00

solar cell parameters deduced from calculated IV-curve:

Voc = 0.551377 Volt

Jsc = 31.46595361 mA/cm2

FF = 64.7782 %

eta = 11.2388 %

V_MPP = 0.435918 Volt

J_MPP = 25.78184033 mA/cm2

# **PbS nanoparticle (Eg = 1.22 eV)**

SCAPS 3.3.07 ELIS-UGent: Version scaps3307.exe, dated 27-01-2018, 10:11:02

Simulation of I-V curves

Single shot simulation # 1

Calculation started : 16-9-2021 at 14:29:41

problem definition file : c:\Program Files (x86)\Scaps3307\def\example CIGS.def

last saved: 01-09-2015 at 07:44:06

Calculation under illumination

Optical generation from internal SCAPS calculation

Illumination spectrum read from file

spectrum file :

C:\Program Files (x86)\Scaps3307\spectrum\AM1_5G 1 sun.spe last saved: 22-05-2009 at 09:59:48

Neutral density filter: ND 0.0000

Transmission of ND filter: 1.0000e+00

Power from spectrum: 100.0000 mW/cm2

Incident power on solar cell structure 100.0000 mW/cm2

Working point conditions

Temperature 300.00 K

Workpoint bias voltage 0.0000 Volt

Frequency 1.0000e+06 Hz

Voltage reference convention: voltage referred to right contact, voltage V applied to left contact

Current reference convention: current positive when entering the left contact

Power generated by the cell: = -V*I; power consumed by the cell = +V*I

v(V) jtot(mA/cm2) j_total_rec(mA/cm2) j_total_gen(mA/cm2) jbulk(mA/cm2) jifr(mA/cm2) jminor_left(mA/cm2) jminor_right(mA/cm2) j_SRH(mA/cm2) j_Radiative(mA/cm2) j_Auger(mA/cm2)

0.000000 -1.14354567e+01 2.70192210e+01 3.84517532e+01 2.30385141e+00 2.44969519e+01 1.34309856e-01 8.41077750e-02 2.30385141e+00 0.00000000e+00 0.00000000e+00

0.020000 -1.13862475e+01 2.70640427e+01 3.84517532e+01 2.34690784e+00 2.44984909e+01 1.34531885e-01 8.41120318e-02 2.34690784e+00 0.00000000e+00 0.00000000e+00

0.040000 -1.13395516e+01 2.71107251e+01 3.84517532e+01 2.39179847e+00 2.45000578e+01 1.34752217e-01 8.41165734e-02 2.39179847e+00 0.00000000e+00 0.00000000e+00

0.060000 -1.12889275e+01 2.71593785e+01 3.84517532e+01 2.43863425e+00 2.45016516e+01 1.34970686e-01 8.41219703e-02 2.43863425e+00 0.00000000e+00 0.00000000e+00

0.080000 -1.12380950e+01 2.72101857e+01 3.84517532e+01 2.48759570e+00 2.45032755e+01 1.35187467e-01 8.41270825e-02 2.48759570e+00 0.00000000e+00 0.00000000e+00

0.100000 -1.11849405e+01 2.72633196e+01 3.84517532e+01 2.53885507e+00 2.45049298e+01 1.35402549e-01 8.41321222e-02 2.53885507e+00 0.00000000e+00 0.00000000e+00

0.120000 -1.11292456e+01 2.73189964e+01 3.84517532e+01 2.59262756e+00 2.45066157e+01 1.35615981e-01 8.41371094e-02 2.59262756e+00 0.00000000e+00 0.00000000e+00

0.140000 -1.10707478e+01 2.73774789e+01 3.84517532e+01 2.64917460e+00 2.45083339e+01 1.35827818e-01 8.41425435e-02 2.64917460e+00 0.00000000e+00 0.00000000e+00

0.160000 -1.10091227e+01 2.74390952e+01 3.84517532e+01 2.70882303e+00 2.45100859e+01 1.36038114e-01 8.41481902e-02 2.70882303e+00 0.00000000e+00 0.00000000e+00

0.180000 -1.09439277e+01 2.75042769e+01 3.84517532e+01 2.77200302e+00 2.45118733e+01 1.36246926e-01 8.41537210e-02 2.77200302e+00 0.00000000e+00 0.00000000e+00

0.200000 -1.08745741e+01 2.75736247e+01 3.84517532e+01 2.83931365e+00 2.45136968e+01 1.36454314e-01 8.41598609e-02 2.83931365e+00 0.00000000e+00 0.00000000e+00

0.220000 -1.08001828e+01 2.76480157e+01 3.84517532e+01 2.91163072e+00 2.45155580e+01 1.36660338e-01 8.41666222e-02 2.91163072e+00 0.00000000e+00 0.00000000e+00

0.240000 -1.07194221e+01 2.77287843e+01 3.84517532e+01 2.99028668e+00 2.45174590e+01 1.36865062e-01 8.41734957e-02 2.99028668e+00 0.00000000e+00 0.00000000e+00

0.260000 -1.06302122e+01 2.78180085e+01 3.84517532e+01 3.07735799e+00 2.45194019e+01 1.37068551e-01 8.41800425e-02 3.07735799e+00 0.00000000e+00 0.00000000e+00

0.280000 -1.05293055e+01 2.79189486e+01 3.84517532e+01 3.17610304e+00 2.45213873e+01 1.37270874e-01 8.41874570e-02 3.17610304e+00 0.00000000e+00 0.00000000e+00

0.300000 -1.04116056e+01 2.80366916e+01 3.84517532e+01 3.29160692e+00 2.45234170e+01 1.37472101e-01 8.41956236e-02 3.29160692e+00 0.00000000e+00 0.00000000e+00

0.320000 -1.02692895e+01 2.81790764e+01 3.84517532e+01 3.43170665e+00 2.45254934e+01 1.37672310e-01 8.42040713e-02 3.43170665e+00 0.00000000e+00 0.00000000e+00

0.340000 -1.00922970e+01 2.83580383e+01 3.84517532e+01 3.60833495e+00 2.45276197e+01 1.37871635e-01 8.42120593e-02 3.60833495e+00 0.00000000e+00 0.00000000e+00

0.360000 -9.85720549e+00 2.85914042e+01 3.84517532e+01 3.83931889e+00 2.45297930e+01 1.38070030e-01 8.42223281e-02 3.83931889e+00 0.00000000e+00 0.00000000e+00

0.380000 -9.53874041e+00 2.89058383e+01 3.84517532e+01 4.15132064e+00 2.45320170e+01 1.38267668e-01 8.42329331e-02 4.15132064e+00 0.00000000e+00 0.00000000e+00

0.400000 -9.10417632e+00 2.93409801e+01 3.84517532e+01 4.58397543e+00 2.45342959e+01 1.38464920e-01 8.42438029e-02 4.58397543e+00 0.00000000e+00 0.00000000e+00

0.420000 -8.49094259e+00 2.99550004e+01 3.84517532e+01 5.19545467e+00 2.45366271e+01 1.38661987e-01 8.42566636e-02 5.19545467e+00 0.00000000e+00 0.00000000e+00

0.440000 -7.61391864e+00 3.08330346e+01 3.84517532e+01 6.07089305e+00 2.45390119e+01 1.38859423e-01 8.42702740e-02 6.07089305e+00 0.00000000e+00 0.00000000e+00

0.460000 -6.35003617e+00 3.20981598e+01 3.84517532e+01 7.33336748e+00 2.45414483e+01 1.39058231e-01 8.42858584e-02 7.33336748e+00 0.00000000e+00 0.00000000e+00

0.480000 -4.52131520e+00 3.39252678e+01 3.84517532e+01 9.15777102e+00 2.45439324e+01 1.39260177e-01 8.43042663e-02 9.15777102e+00 0.00000000e+00 0.00000000e+00

0.500000 -1.89279143e+00 3.65581064e+01 3.84517532e+01 1.17878477e+01 2.45464646e+01 1.39468842e-01 8.43252122e-02 1.17878477e+01 0.00000000e+00 0.00000000e+00

0.520000 1.86936458e+00 4.03248283e+01 3.84517532e+01 1.55517506e+01 2.45490390e+01 1.39690115e-01 8.43485796e-02 1.55517506e+01 0.00000000e+00 0.00000000e+00

0.540000 7.19805762e+00 4.56520250e+01 3.84517532e+01 2.08760660e+01 2.45516476e+01 1.39934350e-01 8.43770607e-02 2.08760660e+01 0.00000000e+00 0.00000000e+00

0.560000 1.46174841e+01 5.30721765e+01 3.84517532e+01 2.82932567e+01 2.45542909e+01 1.40219122e-01 8.44097082e-02 2.82932567e+01 0.00000000e+00 0.00000000e+00

0.580000 2.47606843e+01 6.32154965e+01 3.84517532e+01 3.84335075e+01 2.45569690e+01 1.40572280e-01 8.44476575e-02 3.84335075e+01 0.00000000e+00 0.00000000e+00

0.600000 3.83377801e+01 7.67922158e+01 3.84517532e+01 5.20069968e+01 2.45596919e+01 1.41036555e-01 8.44904793e-02 5.20069968e+01 0.00000000e+00 0.00000000e+00

0.620000 5.61094194e+01 9.45632370e+01 3.84517532e+01 6.97745426e+01 2.45624805e+01 1.41675085e-01 8.45387916e-02 6.97745426e+01 0.00000000e+00 0.00000000e+00

0.640000 7.88530348e+01 1.17306289e+02 3.84517532e+01 9.25137436e+01 2.45653756e+01 1.42578840e-01 8.45913722e-02 9.25137436e+01 0.00000000e+00 0.00000000e+00

0.660000 1.07329680e+02 1.45782541e+02 3.84517532e+01 1.20985572e+02 2.45684432e+01 1.43877727e-01 8.46479098e-02 1.20985572e+02 0.00000000e+00 0.00000000e+00

0.680000 1.42257077e+02 1.80709742e+02 3.84517532e+01 1.55907487e+02 2.45717886e+01 1.45758773e-01 8.47076470e-02 1.55907487e+02 0.00000000e+00 0.00000000e+00

0.700000 1.84304467e+02 2.22757118e+02 3.84517532e+01 1.97948274e+02 2.45755768e+01 1.48497078e-01 8.47700611e-02 1.97948274e+02 0.00000000e+00 0.00000000e+00

0.720000 2.34142839e+02 2.72595788e+02 3.84517532e+01 2.47778380e+02 2.45800675e+01 1.52507103e-01 8.48331855e-02 2.47778380e+02 0.00000000e+00 0.00000000e+00

0.740000 2.92596887e+02 3.31050489e+02 3.84517532e+01 3.06221509e+02 2.45856627e+01 1.58420374e-01 8.48976582e-02 3.06221509e+02 0.00000000e+00 0.00000000e+00

0.760000 3.60953082e+02 3.99405277e+02 3.84517532e+01 3.74560144e+02 2.45929824e+01 1.67188842e-01 8.49620268e-02 3.74560144e+02 0.00000000e+00 0.00000000e+00

0.780000 4.41038281e+02 4.79494141e+02 3.84517532e+01 4.54625961e+02 2.46029736e+01 1.80178450e-01 8.50285221e-02 4.54625961e+02 0.00000000e+00 0.00000000e+00

0.800000 5.35917564e+02 5.74370637e+02 3.84517532e+01 5.49469259e+02 2.46169949e+01 1.99285102e-01 8.50980211e-02 5.49469259e+02 0.00000000e+00 0.00000000e+00

solar cell parameters deduced from calculated IV-curve:

Voc = 0.510811 Volt

Jsc = 11.43545674 mA/cm2

FF = 62.3960 %

eta = 3.6448 %

V_MPP = 0.394590 Volt

J_MPP = 9.23685536 mA/cm2

SCAPS 3.3.07 ELIS-UGent: Version scaps3307.exe, dated 27-01-2018, 10:11:02

Simulation of I-V curves

Single shot simulation # 2

Calculation started : 16-9-2021 at 14:31:14

problem definition file : c:\Program Files (x86)\Scaps3307\def\example CIGS.def

last saved: 01-09-2015 at 07:44:06

Calculation under illumination

Optical generation from internal SCAPS calculation

Illumination spectrum read from file

spectrum file :

C:\Program Files (x86)\Scaps3307\spectrum\AM1_5G 1 sun.spe last saved: 22-05-2009 at 09:59:48

Neutral density filter: ND 0.0000

Transmission of ND filter: 1.0000e+00

Power from spectrum: 100.0000 mW/cm2

Incident power on solar cell structure 100.0000 mW/cm2

Working point conditions

Temperature 300.00 K

Workpoint bias voltage 0.0000 Volt

Frequency 1.0000e+06 Hz

Voltage reference convention: voltage referred to right contact, voltage V applied to left contact

Current reference convention: current positive when entering the left contact

Power generated by the cell: = -V*I; power consumed by the cell = +V*I

v(V) jtot(mA/cm2) j_total_rec(mA/cm2) j_total_gen(mA/cm2) jbulk(mA/cm2) jifr(mA/cm2) jminor_left(mA/cm2) jminor_right(mA/cm2) j_SRH(mA/cm2) j_Radiative(mA/cm2) j_Auger(mA/cm2)

0.000000 -1.15951177e+01 2.68611123e+01 3.84517532e+01 2.18272834e+00 2.44530483e+01 1.32217760e-01 9.31179750e-02 2.18272834e+00 0.00000000e+00 0.00000000e+00

0.020000 -1.15483358e+01 2.69031191e+01 3.84517532e+01 2.22373815e+00 2.44537981e+01 1.32462129e-01 9.31207659e-02 2.22373815e+00 0.00000000e+00 0.00000000e+00

0.040000 -1.15045505e+01 2.69469193e+01 3.84517532e+01 2.26653806e+00 2.44545557e+01 1.32704474e-01 9.31210152e-02 2.26653806e+00 0.00000000e+00 0.00000000e+00

0.060000 -1.14588284e+01 2.69926556e+01 3.84517532e+01 2.31127079e+00 2.44553187e+01 1.32944830e-01 9.31212921e-02 2.31127079e+00 0.00000000e+00 0.00000000e+00

0.080000 -1.14110180e+01 2.70404867e+01 3.84517532e+01 2.35809488e+00 2.44560870e+01 1.33183242e-01 9.31215658e-02 2.35809488e+00 0.00000000e+00 0.00000000e+00

0.100000 -1.13609205e+01 2.70905941e+01 3.84517532e+01 2.40719162e+00 2.44568609e+01 1.33419759e-01 9.31218365e-02 2.40719162e+00 0.00000000e+00 0.00000000e+00

0.120000 -1.13083362e+01 2.71431951e+01 3.84517532e+01 2.45877821e+00 2.44576403e+01 1.33654429e-01 9.31221041e-02 2.45877821e+00 0.00000000e+00 0.00000000e+00

0.140000 -1.12529849e+01 2.71985568e+01 3.84517532e+01 2.51312174e+00 2.44584254e+01 1.33887306e-01 9.31223689e-02 2.51312174e+00 0.00000000e+00 0.00000000e+00

0.160000 -1.11945439e+01 2.72570122e+01 3.84517532e+01 2.57055501e+00 2.44592161e+01 1.34118442e-01 9.31226308e-02 2.57055501e+00 0.00000000e+00 0.00000000e+00

0.180000 -1.11325817e+01 2.73189908e+01 3.84517532e+01 2.63150745e+00 2.44600126e+01 1.34347896e-01 9.31228898e-02 2.63150745e+00 0.00000000e+00 0.00000000e+00

0.200000 -1.10665006e+01 2.73850835e+01 3.84517532e+01 2.69656971e+00 2.44608149e+01 1.34575726e-01 9.31231458e-02 2.69656971e+00 0.00000000e+00 0.00000000e+00

0.220000 -1.09954316e+01 2.74561565e+01 3.84517532e+01 2.76660796e+00 2.44616232e+01 1.34801997e-01 9.31233992e-02 2.76660796e+00 0.00000000e+00 0.00000000e+00

0.240000 -1.09180756e+01 2.75335381e+01 3.84517532e+01 2.84295028e+00 2.44624374e+01 1.35026776e-01 9.31236500e-02 2.84295028e+00 0.00000000e+00 0.00000000e+00

0.260000 -1.08323156e+01 2.76193240e+01 3.84517532e+01 2.92769230e+00 2.44632577e+01 1.35250139e-01 9.31238982e-02 2.92769230e+00 0.00000000e+00 0.00000000e+00

0.280000 -1.07347929e+01 2.77168677e+01 3.84517532e+01 3.02418729e+00 2.44640841e+01 1.35472168e-01 9.31241437e-02 3.02418729e+00 0.00000000e+00 0.00000000e+00

0.300000 -1.06201523e+01 2.78315158e+01 3.84517532e+01 3.13778176e+00 2.44649167e+01 1.35692959e-01 9.31243867e-02 3.13778176e+00 0.00000000e+00 0.00000000e+00

0.320000 -1.04800195e+01 2.79716610e+01 3.84517532e+01 3.27686816e+00 2.44657556e+01 1.35912628e-01 9.31246276e-02 3.27686816e+00 0.00000000e+00 0.00000000e+00

0.340000 -1.03014304e+01 2.81502653e+01 3.84517532e+01 3.45440829e+00 2.44666008e+01 1.36131319e-01 9.31248662e-02 3.45440829e+00 0.00000000e+00 0.00000000e+00

0.360000 -1.00645667e+01 2.83871492e+01 3.84517532e+01 3.69022237e+00 2.44674525e+01 1.36349230e-01 9.31251030e-02 3.69022237e+00 0.00000000e+00 0.00000000e+00

0.380000 -9.73925525e+00 2.87124787e+01 3.84517532e+01 4.01447604e+00 2.44683106e+01 1.36566644e-01 9.31253378e-02 4.01447604e+00 0.00000000e+00 0.00000000e+00

0.400000 -9.27973213e+00 2.91720264e+01 3.84517532e+01 4.47294143e+00 2.44691754e+01 1.36784009e-01 9.31255718e-02 4.47294143e+00 0.00000000e+00 0.00000000e+00

0.420000 -8.61674038e+00 2.98350603e+01 3.84517532e+01 5.13488564e+00 2.44700468e+01 1.37002069e-01 9.31258047e-02 5.13488564e+00 0.00000000e+00 0.00000000e+00

0.440000 -7.64557907e+00 3.08062713e+01 3.84517532e+01 6.10499819e+00 2.44709250e+01 1.37222141e-01 9.31260389e-02 6.10499819e+00 0.00000000e+00 0.00000000e+00

0.460000 -6.20780467e+00 3.22440863e+01 3.84517532e+01 7.54170352e+00 2.44718099e+01 1.37446653e-01 9.31262750e-02 7.54170352e+00 0.00000000e+00 0.00000000e+00

0.480000 -4.06290479e+00 3.43891187e+01 3.84517532e+01 9.68561031e+00 2.44727017e+01 1.37680208e-01 9.31265158e-02 9.68561031e+00 0.00000000e+00 0.00000000e+00

0.500000 -8.43601442e-01 3.76085582e+01 3.84517532e+01 1.29038994e+01 2.44736003e+01 1.37931728e-01 9.31267640e-02 1.29038994e+01 0.00000000e+00 0.00000000e+00

0.520000 4.01294986e+00 4.24653399e+01 3.84517532e+01 1.77594884e+01 2.44745058e+01 1.38218732e-01 9.31270247e-02 1.77594884e+01 0.00000000e+00 0.00000000e+00

0.540000 1.13728436e+01 4.98255820e+01 3.84517532e+01 2.51184607e+01 2.44754181e+01 1.38575899e-01 9.31273069e-02 2.51184607e+01 0.00000000e+00 0.00000000e+00

0.560000 2.25715701e+01 6.10250780e+01 3.84517532e+01 3.63165411e+01 2.44763371e+01 1.39072197e-01 9.31276228e-02 3.63165411e+01 0.00000000e+00 0.00000000e+00

0.580000 3.96758414e+01 7.81269785e+01 3.84517532e+01 5.34167430e+01 2.44772650e+01 1.39844975e-01 9.31255274e-02 5.34167430e+01 0.00000000e+00 0.00000000e+00

0.600000 6.58683832e+01 1.04319429e+02 3.84517532e+01 7.96069386e+01 2.44781966e+01 1.41167737e-01 9.31259110e-02 7.96069386e+01 0.00000000e+00 0.00000000e+00

0.620000 1.06073148e+02 1.44524068e+02 3.84517532e+01 1.19808224e+02 2.44791336e+01 1.43583218e-01 9.31264300e-02 1.19808224e+02 0.00000000e+00 0.00000000e+00

0.640000 1.67857493e+02 2.06307991e+02 3.84517532e+01 1.81586626e+02 2.44800751e+01 1.48162255e-01 9.31271143e-02 1.81586626e+02 0.00000000e+00 0.00000000e+00

0.660000 2.62746789e+02 3.01195246e+02 3.84517532e+01 2.76464100e+02 2.44810171e+01 1.56997811e-01 9.31306248e-02 2.76464100e+02 0.00000000e+00 0.00000000e+00

0.680000 4.07971935e+02 4.46411170e+02 3.84517532e+01 4.21661952e+02 2.44819628e+01 1.74122955e-01 9.31321145e-02 4.21661952e+02 0.00000000e+00 0.00000000e+00

0.700000 6.28409872e+02 6.66864383e+02 3.84517532e+01 6.42081191e+02 2.44829075e+01 2.07149897e-01 9.31341773e-02 6.42081191e+02 0.00000000e+00 0.00000000e+00

0.720000 9.58226002e+02 9.96698861e+02 3.84517532e+01 9.71851838e+02 2.44838486e+01 2.70037332e-01 9.31371974e-02 9.71851838e+02 0.00000000e+00 0.00000000e+00

0.740000 1.44036563e+03 1.47895840e+03 3.84517532e+01 1.45399306e+03 2.44847873e+01 3.87414467e-01 9.31390498e-02 1.45399306e+03 0.00000000e+00 0.00000000e+00

0.760000 2.12344860e+03 2.16175600e+03 3.84517532e+01 2.13657635e+03 2.44857166e+01 6.00791654e-01 9.31474544e-02 2.13657635e+03 0.00000000e+00 0.00000000e+00

0.780000 3.05116886e+03 3.08984220e+03 3.84517532e+01 3.06428601e+03 2.44866488e+01 9.76380141e-01 9.31540744e-02 3.06428601e+03 0.00000000e+00 0.00000000e+00

0.800000 4.25745265e+03 4.29552717e+03 3.84517532e+01 4.26933139e+03 2.44875857e+01 1.61503034e+00 9.31648683e-02 4.26933139e+03 0.00000000e+00 0.00000000e+00

solar cell parameters deduced from calculated IV-curve:

Voc = 0.504015 Volt

Jsc = 11.59511768 mA/cm2

FF = 63.6160 %

eta = 3.7178 %

V_MPP = 0.392871 Volt

J_MPP = 9.46313556 mA/cm2

SCAPS 3.3.07 ELIS-UGent: Version scaps3307.exe, dated 27-01-2018, 10:11:02

Simulation of I-V curves

Single shot simulation # 3

Calculation started : 16-9-2021 at 14:31:51

problem definition file : c:\Program Files (x86)\Scaps3307\def\example CIGS.def

last saved: 01-09-2015 at 07:44:06

Calculation under illumination

Optical generation from internal SCAPS calculation

Illumination spectrum read from file

spectrum file :

C:\Program Files (x86)\Scaps3307\spectrum\AM1_5G 1 sun.spe last saved: 22-05-2009 at 09:59:48

Neutral density filter: ND 0.0000

Transmission of ND filter: 1.0000e+00

Power from spectrum: 100.0000 mW/cm2

Incident power on solar cell structure 100.0000 mW/cm2

Working point conditions

Temperature 300.00 K

Workpoint bias voltage 0.0000 Volt

Frequency 1.0000e+06 Hz

Voltage reference convention: voltage referred to right contact, voltage V applied to left contact

Current reference convention: current positive when entering the left contact

Power generated by the cell: = -V*I; power consumed by the cell = +V*I

v(V) jtot(mA/cm2) j_total_rec(mA/cm2) j_total_gen(mA/cm2) jbulk(mA/cm2) jifr(mA/cm2) jminor_left(mA/cm2) jminor_right(mA/cm2) j_SRH(mA/cm2) j_Radiative(mA/cm2) j_Auger(mA/cm2)

0.000000 -1.17281393e+01 2.67234657e+01 3.84517532e+01 2.19133590e+00 2.42538868e+01 1.31727731e-01 1.46515242e-01 2.19133590e+00 0.00000000e+00 0.00000000e+00

0.020000 -1.16862199e+01 2.67654016e+01 3.84517532e+01 2.23286964e+00 2.42540333e+01 1.31983429e-01 1.46515244e-01 2.23286964e+00 0.00000000e+00 0.00000000e+00

0.040000 -1.16424644e+01 2.68091586e+01 3.84517532e+01 2.27622580e+00 2.42541807e+01 1.32236889e-01 1.46515244e-01 2.27622580e+00 0.00000000e+00 0.00000000e+00

0.060000 -1.15967387e+01 2.68548861e+01 3.84517532e+01 2.32155369e+00 2.42543290e+01 1.32488161e-01 1.46515244e-01 2.32155369e+00 0.00000000e+00 0.00000000e+00

0.080000 -1.15488724e+01 2.69027543e+01 3.84517532e+01 2.36902356e+00 2.42544782e+01 1.32737295e-01 1.46515244e-01 2.36902356e+00 0.00000000e+00 0.00000000e+00

0.100000 -1.14986750e+01 2.69529537e+01 3.84517532e+01 2.41882585e+00 2.42546283e+01 1.32984342e-01 1.46515244e-01 2.41882585e+00 0.00000000e+00 0.00000000e+00

0.120000 -1.14459323e+01 2.70056986e+01 3.84517532e+01 2.47117479e+00 2.42547792e+01 1.33229355e-01 1.46515244e-01 2.47117479e+00 0.00000000e+00 0.00000000e+00

0.140000 -1.13903907e+01 2.70612426e+01 3.84517532e+01 2.52632390e+00 2.42549311e+01 1.33472390e-01 1.46515244e-01 2.52632390e+00 0.00000000e+00 0.00000000e+00

0.160000 -1.13317293e+01 2.71199064e+01 3.84517532e+01 2.58459379e+00 2.42550839e+01 1.33713504e-01 1.46515244e-01 2.58459379e+00 0.00000000e+00 0.00000000e+00

0.180000 -1.12695228e+01 2.71821159e+01 3.84517532e+01 2.64641031e+00 2.42552376e+01 1.33952757e-01 1.46515244e-01 2.64641031e+00 0.00000000e+00 0.00000000e+00

0.200000 -1.12031811e+01 2.72484607e+01 3.84517532e+01 2.71236296e+00 2.42553923e+01 1.34190213e-01 1.46515244e-01 2.71236296e+00 0.00000000e+00 0.00000000e+00

0.220000 -1.11318453e+01 2.73197998e+01 3.84517532e+01 2.78331079e+00 2.42555479e+01 1.34425938e-01 1.46515244e-01 2.78331079e+00 0.00000000e+00 0.00000000e+00

0.240000 -1.10541983e+01 2.73974503e+01 3.84517532e+01 2.86057063e+00 2.42557044e+01 1.34660004e-01 1.46515244e-01 2.86057063e+00 0.00000000e+00 0.00000000e+00

0.260000 -1.09681596e+01 2.74834928e+01 3.84517532e+01 2.94622311e+00 2.42558620e+01 1.34892489e-01 1.46515244e-01 2.94622311e+00 0.00000000e+00 0.00000000e+00

0.280000 -1.08704069e+01 2.75812492e+01 3.84517532e+01 3.04359005e+00 2.42560204e+01 1.35123480e-01 1.46515244e-01 3.04359005e+00 0.00000000e+00 0.00000000e+00

0.300000 -1.07556380e+01 2.76960216e+01 3.84517532e+01 3.15797337e+00 2.42561799e+01 1.35353075e-01 1.46515244e-01 3.15797337e+00 0.00000000e+00 0.00000000e+00

0.320000 -1.06154617e+01 2.78362012e+01 3.84517532e+01 3.29776417e+00 2.42563404e+01 1.35581396e-01 1.46515244e-01 3.29776417e+00 0.00000000e+00 0.00000000e+00

0.340000 -1.04368121e+01 2.80148533e+01 3.84517532e+01 3.47602757e+00 2.42565019e+01 1.35808591e-01 1.46515244e-01 3.47602757e+00 0.00000000e+00 0.00000000e+00

0.360000 -1.01997282e+01 2.82519388e+01 3.84517532e+01 3.71272435e+00 2.42566643e+01 1.36034866e-01 1.46515244e-01 3.71272435e+00 0.00000000e+00 0.00000000e+00

0.380000 -9.87412052e+00 2.85775466e+01 3.84517532e+01 4.03794304e+00 2.42568278e+01 1.36260516e-01 1.46515244e-01 4.03794304e+00 0.00000000e+00 0.00000000e+00

0.400000 -9.41479215e+00 2.90368731e+01 3.84517532e+01 4.49687954e+00 2.42569924e+01 1.36486004e-01 1.46515244e-01 4.49687954e+00 0.00000000e+00 0.00000000e+00

0.420000 -8.75355671e+00 2.96981040e+01 3.84517532e+01 5.15771878e+00 2.42571579e+01 1.36712111e-01 1.46515245e-01 5.15771878e+00 0.00000000e+00 0.00000000e+00

0.440000 -7.78685226e+00 3.06648002e+01 3.84517532e+01 6.12402024e+00 2.42573245e+01 1.36940221e-01 1.46515246e-01 6.12402024e+00 0.00000000e+00 0.00000000e+00

0.460000 -6.35662220e+00 3.20950166e+01 3.84517532e+01 7.55383633e+00 2.42574921e+01 1.37172902e-01 1.46515246e-01 7.55383633e+00 0.00000000e+00 0.00000000e+00

0.480000 -4.22113723e+00 3.42304809e+01 3.84517532e+01 9.68888983e+00 2.42576608e+01 1.37415047e-01 1.46515248e-01 9.68888983e+00 0.00000000e+00 0.00000000e+00

0.500000 -1.01049641e+00 3.74410933e+01 3.84517532e+01 1.28990714e+01 2.42578305e+01 1.37676169e-01 1.46515250e-01 1.28990714e+01 0.00000000e+00 0.00000000e+00

0.520000 3.84168145e+00 4.22932662e+01 3.84517532e+01 1.77507747e+01 2.42580012e+01 1.37974977e-01 1.46515253e-01 1.77507747e+01 0.00000000e+00 0.00000000e+00

0.540000 1.12042922e+01 4.96558804e+01 3.84517532e+01 2.51128436e+01 2.42581729e+01 1.38348567e-01 1.46515258e-01 2.51128436e+01 0.00000000e+00 0.00000000e+00

0.560000 2.24140643e+01 6.08657749e+01 3.84517532e+01 3.63220432e+01 2.42583457e+01 1.38870806e-01 1.46515263e-01 3.63220432e+01 0.00000000e+00 0.00000000e+00

0.580000 3.95351800e+01 7.79867651e+01 3.84517532e+01 5.34420416e+01 2.42585192e+01 1.39688998e-01 1.46515272e-01 5.34420416e+01 0.00000000e+00 0.00000000e+00

0.600000 6.57615433e+01 1.04213115e+02 3.84517532e+01 7.96668091e+01 2.42586934e+01 1.41096993e-01 1.46515289e-01 7.96668091e+01 0.00000000e+00 0.00000000e+00

0.620000 1.06039491e+02 1.44490992e+02 3.84517532e+01 1.19941928e+02 2.42588697e+01 1.43679056e-01 1.46515312e-01 1.19941928e+02 0.00000000e+00 0.00000000e+00

0.640000 1.68004577e+02 2.06455711e+02 3.84517532e+01 1.81901559e+02 2.42590454e+01 1.48591241e-01 1.46515350e-01 1.81901559e+02 0.00000000e+00 0.00000000e+00

0.660000 2.63345638e+02 3.01794923e+02 3.84517532e+01 2.77231085e+02 2.42592209e+01 1.58102287e-01 1.46515407e-01 2.77231085e+02 0.00000000e+00 0.00000000e+00

0.680000 4.09663827e+02 4.48103844e+02 3.84517532e+01 4.23521322e+02 2.42593967e+01 1.76610280e-01 1.46515496e-01 4.23521322e+02 0.00000000e+00 0.00000000e+00

0.700000 6.32641288e+02 6.71096028e+02 3.84517532e+01 6.46477452e+02 2.42595710e+01 2.12489860e-01 1.46515630e-01 6.46477452e+02 0.00000000e+00 0.00000000e+00

0.720000 9.68098934e+02 1.00657450e+03 3.84517532e+01 9.81886946e+02 2.42597433e+01 2.81294846e-01 1.46515832e-01 9.81886946e+02 0.00000000e+00 0.00000000e+00

0.740000 1.46230291e+03 1.50073236e+03 3.84517532e+01 1.47591496e+03 2.42599126e+01 4.10969110e-01 1.46516129e-01 1.47591496e+03 0.00000000e+00 0.00000000e+00

0.760000 2.16850960e+03 2.20675544e+03 3.84517532e+01 2.18169918e+03 2.42600774e+01 6.49666593e-01 1.46516554e-01 2.18169918e+03 0.00000000e+00 0.00000000e+00

0.780000 3.13762443e+03 3.17649449e+03 3.84517532e+01 3.15101133e+03 2.42602369e+01 1.07641189e+00 1.46517139e-01 3.15101133e+03 0.00000000e+00 0.00000000e+00

0.800000 4.41135457e+03 4.45022230e+03 3.84517532e+01 4.42399991e+03 2.42603910e+01 1.81548104e+00 1.46517905e-01 4.42399991e+03 0.00000000e+00 0.00000000e+00

solar cell parameters deduced from calculated IV-curve:

Voc = 0.504780 Volt

Jsc = 11.72813931 mA/cm2

FF = 63.6949 %

eta = 3.7708 %

V_MPP = 0.393535 Volt

J_MPP = 9.58191870 mA/cm2

# **PbS QDs (Eg = 1.61 eV)**

SCAPS 3.3.07 ELIS-UGent: Version scaps3307.exe, dated 27-01-2018, 10:11:02

Simulation of I-V curves

Single shot simulation # 4

Calculation started : 16-9-2021 at 14:32:34

problem definition file : c:\Program Files (x86)\Scaps3307\def\example CIGS.def

last saved: 01-09-2015 at 07:44:06

Calculation under illumination

Optical generation from internal SCAPS calculation

Illumination spectrum read from file

spectrum file :

C:\Program Files (x86)\Scaps3307\spectrum\AM1_5G 1 sun.spe last saved: 22-05-2009 at 09:59:48

Neutral density filter: ND 0.0000

Transmission of ND filter: 1.0000e+00

Power from spectrum: 100.0000 mW/cm2

Incident power on solar cell structure 100.0000 mW/cm2

Working point conditions

Temperature 300.00 K

Workpoint bias voltage 0.0000 Volt

Frequency 1.0000e+06 Hz

Voltage reference convention: voltage referred to right contact, voltage V applied to left contact

Current reference convention: current positive when entering the left contact

Power generated by the cell: = -V*I; power consumed by the cell = +V*I

v(V) jtot(mA/cm2) j_total_rec(mA/cm2) j_total_gen(mA/cm2) jbulk(mA/cm2) jifr(mA/cm2) jminor_left(mA/cm2) jminor_right(mA/cm2) j_SRH(mA/cm2) j_Radiative(mA/cm2) j_Auger(mA/cm2)

0.000000 -2.72356984e+01 1.11204480e+01 3.83531211e+01 4.73035690e+00 6.09951491e+00 2.06496713e-01 8.40794318e-02 4.73035690e+00 0.00000000e+00 0.00000000e+00

0.020000 -2.71149494e+01 1.12364020e+01 3.83531211e+01 4.81167442e+00 6.13379813e+00 2.06815482e-01 8.41139642e-02 4.81167442e+00 0.00000000e+00 0.00000000e+00

0.040000 -2.69919951e+01 1.13574504e+01 3.83531211e+01 4.89718946e+00 6.16897853e+00 2.07136013e-01 8.41463615e-02 4.89718946e+00 0.00000000e+00 0.00000000e+00

0.060000 -2.68663240e+01 1.14834075e+01 3.83531211e+01 4.98675224e+00 6.20502102e+00 2.07456231e-01 8.41779907e-02 4.98675224e+00 0.00000000e+00 0.00000000e+00

0.080000 -2.67353405e+01 1.16143363e+01 3.83531211e+01 5.08045267e+00 6.24189891e+00 2.07775250e-01 8.42094694e-02 5.08045267e+00 0.00000000e+00 0.00000000e+00

0.100000 -2.65988959e+01 1.17506744e+01 3.83531211e+01 5.17869112e+00 6.27964928e+00 2.08093292e-01 8.42407028e-02 5.17869112e+00 0.00000000e+00 0.00000000e+00

0.120000 -2.64566073e+01 1.18928782e+01 3.83531211e+01 5.28189205e+00 6.31830408e+00 2.08410431e-01 8.42716929e-02 5.28189205e+00 0.00000000e+00 0.00000000e+00

0.140000 -2.63079709e+01 1.20414726e+01 3.83531211e+01 5.39054852e+00 6.35789494e+00 2.08726713e-01 8.43024658e-02 5.39054852e+00 0.00000000e+00 0.00000000e+00

0.160000 -2.61523569e+01 1.21970899e+01 3.83531211e+01 5.50526023e+00 6.39845440e+00 2.09042178e-01 8.43330561e-02 5.50526023e+00 0.00000000e+00 0.00000000e+00

0.180000 -2.59889761e+01 1.23605231e+01 3.83531211e+01 5.62678664e+00 6.44001607e+00 2.09356864e-01 8.43635055e-02 5.62678664e+00 0.00000000e+00 0.00000000e+00

0.200000 -2.58167914e+01 1.25328109e+01 3.83531211e+01 5.75613176e+00 6.48261451e+00 2.09670810e-01 8.43938605e-02 5.75613176e+00 0.00000000e+00 0.00000000e+00

0.220000 -2.56343862e+01 1.27153777e+01 3.83531211e+01 5.89468451e+00 6.52628492e+00 2.09984054e-01 8.44241743e-02 5.89468451e+00 0.00000000e+00 0.00000000e+00

0.240000 -2.54397235e+01 1.29102606e+01 3.83531211e+01 6.04444705e+00 6.57106244e+00 2.10296630e-01 8.44545141e-02 6.04444705e+00 0.00000000e+00 0.00000000e+00

0.260000 -2.52288656e+01 1.31203341e+01 3.83531211e+01 6.20827604e+00 6.61696463e+00 2.10608265e-01 8.44851640e-02 6.20827604e+00 0.00000000e+00 0.00000000e+00

0.280000 -2.49991210e+01 1.33504066e+01 3.83531211e+01 6.39091535e+00 6.66405581e+00 2.10919599e-01 8.45158045e-02 6.39091535e+00 0.00000000e+00 0.00000000e+00

0.300000 -2.47428802e+01 1.36072566e+01 3.83531211e+01 6.59913045e+00 6.71234901e+00 2.11230374e-01 8.45467384e-02 6.59913045e+00 0.00000000e+00 0.00000000e+00

0.320000 -2.44483237e+01 1.39009172e+01 3.83531211e+01 6.84296687e+00 6.76183179e+00 2.11539972e-01 8.45785388e-02 6.84296687e+00 0.00000000e+00 0.00000000e+00

0.340000 -2.41031257e+01 1.42475724e+01 3.83531211e+01 7.13853076e+00 6.81258139e+00 2.11849716e-01 8.46104978e-02 7.13853076e+00 0.00000000e+00 0.00000000e+00

0.360000 -2.36838426e+01 1.46695878e+01 3.83531211e+01 7.50821475e+00 6.86457080e+00 2.12159054e-01 8.46432224e-02 7.50821475e+00 0.00000000e+00 0.00000000e+00

0.380000 -2.31577580e+01 1.51993157e+01 3.83531211e+01 7.98439643e+00 6.91777432e+00 2.12467903e-01 8.46770731e-02 7.98439643e+00 0.00000000e+00 0.00000000e+00

0.400000 -2.24750230e+01 1.58811166e+01 3.83531211e+01 8.61154560e+00 6.97208258e+00 2.12775408e-01 8.47130292e-02 8.61154560e+00 0.00000000e+00 0.00000000e+00

0.420000 -2.15771027e+01 1.67786584e+01 3.83531211e+01 9.45332058e+00 7.02750420e+00 2.13082746e-01 8.47508555e-02 9.45332058e+00 0.00000000e+00 0.00000000e+00

0.440000 -2.03792111e+01 1.79774175e+01 3.83531211e+01 1.05952623e+01 7.08397376e+00 2.13390639e-01 8.47908691e-02 1.05952623e+01 0.00000000e+00 0.00000000e+00

0.460000 -1.87659383e+01 1.95893828e+01 3.83531211e+01 1.21494884e+01 7.14136091e+00 2.13699703e-01 8.48338320e-02 1.21494884e+01 0.00000000e+00 0.00000000e+00

0.480000 -1.65939560e+01 2.17615042e+01 3.83531211e+01 1.42629999e+01 7.19961200e+00 2.14012436e-01 8.48798336e-02 1.42629999e+01 0.00000000e+00 0.00000000e+00

0.500000 -1.36793113e+01 2.46760963e+01 3.83531211e+01 1.71182118e+01 7.25862302e+00 2.14331999e-01 8.49294033e-02 1.71182118e+01 0.00000000e+00 0.00000000e+00

0.520000 -9.80073633e+00 2.85544204e+01 3.83531211e+01 2.09364323e+01 7.31834145e+00 2.14663913e-01 8.49828134e-02 2.09364323e+01 0.00000000e+00 0.00000000e+00

0.540000 -4.70012328e+00 3.36546417e+01 3.83531211e+01 2.59758099e+01 7.37877479e+00 2.15016829e-01 8.50401872e-02 2.59758099e+01 0.00000000e+00 0.00000000e+00

0.560000 1.90967068e+00 4.02663181e+01 3.83531211e+01 3.25257765e+01 7.44003625e+00 2.15404056e-01 8.51013423e-02 3.25257765e+01 0.00000000e+00 0.00000000e+00

0.580000 1.03481488e+01 4.87040371e+01 3.83531211e+01 4.09007330e+01 7.50229305e+00 2.15844798e-01 8.51662526e-02 4.09007330e+01 0.00000000e+00 0.00000000e+00

0.600000 2.09423930e+01 5.92976135e+01 3.83531211e+01 5.14301109e+01 7.56589994e+00 2.16368313e-01 8.52342929e-02 5.14301109e+01 0.00000000e+00 0.00000000e+00

0.620000 3.40300166e+01 7.23848254e+01 3.83531211e+01 6.44511869e+01 7.63131571e+00 2.17018006e-01 8.53048344e-02 6.44511869e+01 0.00000000e+00 0.00000000e+00

0.640000 4.99582372e+01 8.83127884e+01 3.83531211e+01 8.03104466e+01 7.69910449e+00 2.17860128e-01 8.53771912e-02 8.03104466e+01 0.00000000e+00 0.00000000e+00

0.660000 6.91068354e+01 1.07461287e+02 3.83531211e+01 9.93869467e+01 7.76989067e+00 2.18998366e-01 8.54507577e-02 9.93869467e+01 0.00000000e+00 0.00000000e+00

0.680000 9.19526823e+01 1.30307143e+02 3.83531211e+01 1.22156730e+02 7.84429011e+00 2.20597780e-01 8.55251867e-02 1.22156730e+02 0.00000000e+00 0.00000000e+00

0.700000 1.19184105e+02 1.57538654e+02 3.83531211e+01 1.49307292e+02 7.92284045e+00 2.22920886e-01 8.56005145e-02 1.49307292e+02 0.00000000e+00 0.00000000e+00

0.720000 1.51843555e+02 1.90198195e+02 3.83531211e+01 1.81880166e+02 8.00597602e+00 2.26376519e-01 8.56772055e-02 1.81880166e+02 0.00000000e+00 0.00000000e+00

0.740000 1.91453784e+02 2.29808452e+02 3.83531211e+01 2.21397033e+02 8.09408280e+00 2.31579742e-01 8.57560054e-02 2.21397033e+02 0.00000000e+00 0.00000000e+00

0.760000 2.40104674e+02 2.78459324e+02 3.83531211e+01 2.69946448e+02 8.18761767e+00 2.39420375e-01 8.58377698e-02 2.69946448e+02 0.00000000e+00 0.00000000e+00

0.780000 3.00520347e+02 3.38874889e+02 3.83531211e+01 3.30250589e+02 8.28723781e+00 2.51138747e-01 8.59232804e-02 3.30250589e+02 0.00000000e+00 0.00000000e+00

0.800000 3.76135828e+02 4.14490178e+02 3.83531211e+01 4.05741858e+02 8.39389755e+00 2.68409043e-01 8.60131587e-02 4.05741858e+02 0.00000000e+00 0.00000000e+00

solar cell parameters deduced from calculated IV-curve:

Voc = 0.554668 Volt

Jsc = 27.23569841 mA/cm2

FF = 59.9890 %

eta = 9.0624 %

V_MPP = 0.419707 Volt

J_MPP = 21.59221047 mA/cm2

SCAPS 3.3.07 ELIS-UGent: Version scaps3307.exe, dated 27-01-2018, 10:11:02

Simulation of I-V curves

Single shot simulation # 5

Calculation started : 16-9-2021 at 14:32:58

problem definition file : c:\Program Files (x86)\Scaps3307\def\example CIGS.def

last saved: 01-09-2015 at 07:44:06

Calculation under illumination

Optical generation from internal SCAPS calculation

Illumination spectrum read from file

spectrum file :

C:\Program Files (x86)\Scaps3307\spectrum\AM1_5G 1 sun.spe last saved: 22-05-2009 at 09:59:48

Neutral density filter: ND 0.0000

Transmission of ND filter: 1.0000e+00

Power from spectrum: 100.0000 mW/cm2

Incident power on solar cell structure 100.0000 mW/cm2

Working point conditions

Temperature 300.00 K

Workpoint bias voltage 0.0000 Volt

Frequency 1.0000e+06 Hz

Voltage reference convention: voltage referred to right contact, voltage V applied to left contact

Current reference convention: current positive when entering the left contact

Power generated by the cell: = -V*I; power consumed by the cell = +V*I

v(V) jtot(mA/cm2) j_total_rec(mA/cm2) j_total_gen(mA/cm2) jbulk(mA/cm2) jifr(mA/cm2) jminor_left(mA/cm2) jminor_right(mA/cm2) j_SRH(mA/cm2) j_Radiative(mA/cm2) j_Auger(mA/cm2)

0.000000 -2.82692135e+01 1.00841736e+01 3.83531211e+01 4.65137369e+00 5.13537973e+00 2.01518109e-01 9.59020290e-02 4.65137369e+00 0.00000000e+00 0.00000000e+00

0.020000 -2.81686853e+01 1.01843835e+01 3.83531211e+01 4.73926454e+00 5.14729181e+00 2.01924737e-01 9.59023849e-02 4.73926454e+00 0.00000000e+00 0.00000000e+00

0.040000 -2.80645169e+01 1.02885634e+01 3.83531211e+01 4.83105967e+00 5.15927253e+00 2.02328437e-01 9.59027302e-02 4.83105967e+00 0.00000000e+00 0.00000000e+00

0.060000 -2.79560706e+01 1.03970218e+01 3.83531211e+01 4.92706604e+00 5.17132335e+00 2.02729290e-01 9.59030717e-02 4.92706604e+00 0.00000000e+00 0.00000000e+00

0.080000 -2.78430003e+01 1.05101051e+01 3.83531211e+01 5.02762894e+00 5.18344534e+00 2.03127371e-01 9.59034092e-02 5.02762894e+00 0.00000000e+00 0.00000000e+00

0.100000 -2.77249178e+01 1.06282014e+01 3.83531211e+01 5.13313538e+00 5.19563956e+00 2.03522755e-01 9.59037431e-02 5.13313538e+00 0.00000000e+00 0.00000000e+00

0.120000 -2.76013899e+01 1.07517442e+01 3.83531211e+01 5.24401752e+00 5.20790710e+00 2.03915523e-01 9.59040730e-02 5.24401752e+00 0.00000000e+00 0.00000000e+00

0.140000 -2.74719277e+01 1.08812226e+01 3.83531211e+01 5.36076336e+00 5.22024906e+00 2.04305760e-01 9.59043993e-02 5.36076336e+00 0.00000000e+00 0.00000000e+00

0.160000 -2.73359648e+01 1.10172027e+01 3.83531211e+01 5.48393791e+00 5.23266655e+00 2.04693553e-01 9.59047222e-02 5.48393791e+00 0.00000000e+00 0.00000000e+00

0.180000 -2.71928278e+01 1.11603586e+01 3.83531211e+01 5.61421380e+00 5.24516072e+00 2.05078996e-01 9.59050416e-02 5.61421380e+00 0.00000000e+00 0.00000000e+00

0.200000 -2.70416927e+01 1.13115139e+01 3.83531211e+01 5.75241366e+00 5.25773271e+00 2.05462186e-01 9.59053573e-02 5.75241366e+00 0.00000000e+00 0.00000000e+00

0.220000 -2.68815166e+01 1.14717121e+01 3.83531211e+01 5.89957952e+00 5.27038369e+00 2.05843227e-01 9.59056698e-02 5.89957952e+00 0.00000000e+00 0.00000000e+00

0.240000 -2.67109121e+01 1.16423407e+01 3.83531211e+01 6.05709763e+00 5.28311486e+00 2.06222231e-01 9.59059780e-02 6.05709763e+00 0.00000000e+00 0.00000000e+00

0.260000 -2.65279280e+01 1.18253509e+01 3.83531211e+01 6.22691789e+00 5.29592741e+00 2.06599319e-01 9.59062840e-02 6.22691789e+00 0.00000000e+00 0.00000000e+00

0.280000 -2.63296730e+01 1.20236341e+01 3.83531211e+01 6.41193029e+00 5.30882258e+00 2.06974624e-01 9.59065869e-02 6.41193029e+00 0.00000000e+00 0.00000000e+00

0.300000 -2.61116721e+01 1.22416651e+01 3.83531211e+01 6.61660837e+00 5.32180160e+00 2.07348298e-01 9.59068867e-02 6.61660837e+00 0.00000000e+00 0.00000000e+00

0.320000 -2.58667935e+01 1.24865755e+01 3.83531211e+01 6.84808204e+00 5.33486572e+00 2.07720516e-01 9.59071837e-02 6.84808204e+00 0.00000000e+00 0.00000000e+00

0.340000 -2.55835536e+01 1.27698481e+01 3.83531211e+01 7.11783288e+00 5.34801623e+00 2.08091496e-01 9.59074780e-02 7.11783288e+00 0.00000000e+00 0.00000000e+00

0.360000 -2.52435700e+01 1.31098645e+01 3.83531211e+01 7.44424087e+00 5.36125439e+00 2.08461523e-01 9.59077694e-02 7.44424087e+00 0.00000000e+00 0.00000000e+00

0.380000 -2.48178224e+01 1.35356412e+01 3.83531211e+01 7.85632063e+00 5.37458149e+00 2.08830991e-01 9.59080587e-02 7.85632063e+00 0.00000000e+00 0.00000000e+00

0.400000 -2.42611802e+01 1.40923047e+01 3.83531211e+01 8.39919704e+00 5.38799882e+00 2.09200491e-01 9.59083461e-02 8.39919704e+00 0.00000000e+00 0.00000000e+00

0.420000 -2.35043348e+01 1.48491662e+01 3.83531211e+01 9.14217900e+00 5.40150762e+00 2.09570957e-01 9.59086335e-02 9.14217900e+00 0.00000000e+00 0.00000000e+00

0.440000 -2.24417532e+01 1.59117498e+01 3.83531211e+01 1.01907878e+01 5.41510910e+00 2.09943967e-01 9.59089196e-02 1.01907878e+01 0.00000000e+00 0.00000000e+00

0.460000 -2.09134675e+01 1.74400378e+01 3.83531211e+01 1.17050019e+01 5.42880439e+00 2.10322305e-01 9.59092091e-02 1.17050019e+01 0.00000000e+00 0.00000000e+00

0.480000 -1.86768200e+01 1.96766897e+01 3.83531211e+01 1.39274746e+01 5.44259450e+00 2.10711083e-01 9.59095022e-02 1.39274746e+01 0.00000000e+00 0.00000000e+00

0.500000 -1.53622869e+01 2.29911681e+01 3.83531211e+01 1.72276582e+01 5.45648017e+00 2.11119951e-01 9.59098041e-02 1.72276582e+01 0.00000000e+00 0.00000000e+00

0.520000 -1.04046566e+01 2.79487825e+01 3.83531211e+01 2.21708430e+01 5.47046184e+00 2.11567502e-01 9.59101174e-02 2.21708430e+01 0.00000000e+00 0.00000000e+00

0.540000 -2.93512575e+00 3.54182469e+01 3.83531211e+01 2.96257071e+01 5.48453930e+00 2.12090025e-01 9.59104581e-02 2.96257071e+01 0.00000000e+00 0.00000000e+00

0.560000 8.38515045e+00 4.67385381e+01 3.83531211e+01 4.09311568e+01 5.49871148e+00 2.12758936e-01 9.59108366e-02 4.09311568e+01 0.00000000e+00 0.00000000e+00

0.580000 2.56242263e+01 6.39771274e+01 3.83531211e+01 5.81545255e+01 5.51297526e+00 2.13715349e-01 9.59112646e-02 5.81545255e+01 0.00000000e+00 0.00000000e+00

0.600000 5.19754462e+01 9.03283466e+01 3.83531211e+01 8.44898694e+01 5.52732675e+00 2.15238606e-01 9.59117897e-02 8.44898694e+01 0.00000000e+00 0.00000000e+00

0.620000 9.23698804e+01 1.30722723e+02 3.83531211e+01 1.24867173e+02 5.54175717e+00 2.17880572e-01 9.59124577e-02 1.24867173e+02 0.00000000e+00 0.00000000e+00

0.640000 1.54387466e+02 1.92739998e+02 3.83531211e+01 1.86865106e+02 5.55625282e+00 2.22726375e-01 9.59133450e-02 1.86865106e+02 0.00000000e+00 0.00000000e+00

0.660000 2.49570819e+02 2.87921747e+02 3.83531211e+01 2.82023149e+02 5.57079207e+00 2.31891302e-01 9.59145713e-02 2.82023149e+02 0.00000000e+00 0.00000000e+00

0.680000 3.95173689e+02 4.33516430e+02 3.83531211e+01 4.27585729e+02 5.58534196e+00 2.49442833e-01 9.59163133e-02 4.27585729e+02 0.00000000e+00 0.00000000e+00

0.700000 6.16104945e+02 6.54460535e+02 3.83531211e+01 6.48481715e+02 5.59985497e+00 2.83045371e-01 9.59188374e-02 6.48481715e+02 0.00000000e+00 0.00000000e+00

0.720000 9.46552528e+02 9.84925643e+02 3.83531211e+01 9.78868723e+02 5.61426539e+00 3.46732114e-01 9.59224913e-02 9.78868723e+02 0.00000000e+00 0.00000000e+00

0.740000 1.42948231e+03 1.46797271e+03 3.83531211e+01 1.46178305e+03 5.62849226e+00 4.65233578e-01 9.59277384e-02 1.46178305e+03 0.00000000e+00 0.00000000e+00

0.760000 2.11349650e+03 2.15170724e+03 3.83531211e+01 2.14528868e+03 5.64244817e+00 6.80181736e-01 9.59350472e-02 2.14528868e+03 0.00000000e+00 0.00000000e+00

0.780000 3.04226505e+03 3.08083848e+03 3.83531211e+01 3.07402855e+03 5.65605402e+00 1.05792894e+00 9.59449889e-02 3.07402855e+03 0.00000000e+00 0.00000000e+00

0.800000 4.24963458e+03 4.28761026e+03 3.83531211e+01 4.28014559e+03 5.66926069e+00 1.69945616e+00 9.59578505e-02 4.28014559e+03 0.00000000e+00 0.00000000e+00

solar cell parameters deduced from calculated IV-curve:

Voc = 0.545893 Volt

Jsc = 28.26921348 mA/cm2

FF = 64.1474 %

eta = 9.8992 %

V_MPP = 0.430566 Volt

J_MPP = 22.99110312 mA/cm2

SCAPS 3.3.07 ELIS-UGent: Version scaps3307.exe, dated 27-01-2018, 10:11:02

Simulation of I-V curves

Single shot simulation # 6

Calculation started : 16-9-2021 at 14:33:21

problem definition file : c:\Program Files (x86)\Scaps3307\def\example CIGS.def

last saved: 01-09-2015 at 07:44:06

Calculation under illumination

Optical generation from internal SCAPS calculation

Illumination spectrum read from file

spectrum file :

C:\Program Files (x86)\Scaps3307\spectrum\AM1_5G 1 sun.spe last saved: 22-05-2009 at 09:59:48

Neutral density filter: ND 0.0000

Transmission of ND filter: 1.0000e+00

Power from spectrum: 100.0000 mW/cm2

Incident power on solar cell structure 100.0000 mW/cm2

Working point conditions

Temperature 300.00 K

Workpoint bias voltage 0.0000 Volt

Frequency 1.0000e+06 Hz

Voltage reference convention: voltage referred to right contact, voltage V applied to left contact

Current reference convention: current positive when entering the left contact

Power generated by the cell: = -V*I; power consumed by the cell = +V*I

v(V) jtot(mA/cm2) j_total_rec(mA/cm2) j_total_gen(mA/cm2) jbulk(mA/cm2) jifr(mA/cm2) jminor_left(mA/cm2) jminor_right(mA/cm2) j_SRH(mA/cm2) j_Radiative(mA/cm2) j_Auger(mA/cm2)

0.000000 -3.16421306e+01 6.71081002e+00 3.83531211e+01 5.08251153e+00 1.28109060e+00 2.00692764e-01 1.46515123e-01 5.08251153e+00 0.00000000e+00 0.00000000e+00

0.020000 -3.15431733e+01 6.81000938e+00 3.83531211e+01 5.18070017e+00 1.28167686e+00 2.01117221e-01 1.46515123e-01 5.18070017e+00 0.00000000e+00 0.00000000e+00

0.040000 -3.14395490e+01 6.91364674e+00 3.83531211e+01 5.28332748e+00 1.28226570e+00 2.01538444e-01 1.46515123e-01 5.28332748e+00 0.00000000e+00 0.00000000e+00

0.060000 -3.13311507e+01 7.02205915e+00 3.83531211e+01 5.39073037e+00 1.28285713e+00 2.01956526e-01 1.46515124e-01 5.39073037e+00 0.00000000e+00 0.00000000e+00

0.080000 -3.12176023e+01 7.13562281e+00 3.83531211e+01 5.50328496e+00 1.28345118e+00 2.02371548e-01 1.46515123e-01 5.50328496e+00 0.00000000e+00 0.00000000e+00

0.100000 -3.10984754e+01 7.25476612e+00 3.83531211e+01 5.62141953e+00 1.28404788e+00 2.02783590e-01 1.46515123e-01 5.62141953e+00 0.00000000e+00 0.00000000e+00

0.120000 -3.09732773e+01 7.37998206e+00 3.83531211e+01 5.74562695e+00 1.28464724e+00 2.03192738e-01 1.46515123e-01 5.74562695e+00 0.00000000e+00 0.00000000e+00

0.140000 -3.08414406e+01 7.51183837e+00 3.83531211e+01 5.87647487e+00 1.28524930e+00 2.03599083e-01 1.46515124e-01 5.87647487e+00 0.00000000e+00 0.00000000e+00

0.160000 -3.07023114e+01 7.65098864e+00 3.83531211e+01 6.01461673e+00 1.28585407e+00 2.04002717e-01 1.46515124e-01 6.01461673e+00 0.00000000e+00 0.00000000e+00

0.180000 -3.05551303e+01 7.79819266e+00 3.83531211e+01 6.16081222e+00 1.28646158e+00 2.04403738e-01 1.46515124e-01 6.16081222e+00 0.00000000e+00 0.00000000e+00

0.200000 -3.03989937e+01 7.95435454e+00 3.83531211e+01 6.31596531e+00 1.28707186e+00 2.04802251e-01 1.46515124e-01 6.31596531e+00 0.00000000e+00 0.00000000e+00

0.220000 -3.02327888e+01 8.12058709e+00 3.83531211e+01 6.48118867e+00 1.28768493e+00 2.05198364e-01 1.46515124e-01 6.48118867e+00 0.00000000e+00 0.00000000e+00

0.240000 -3.00550845e+01 8.29832144e+00 3.83531211e+01 6.65791330e+00 1.28830082e+00 2.05592195e-01 1.46515125e-01 6.65791330e+00 0.00000000e+00 0.00000000e+00

0.260000 -2.98639337e+01 8.48950525e+00 3.83531211e+01 6.84808670e+00 1.28891955e+00 2.05983869e-01 1.46515124e-01 6.84808670e+00 0.00000000e+00 0.00000000e+00

0.280000 -2.96565133e+01 8.69696147e+00 3.83531211e+01 7.05453167e+00 1.28954115e+00 2.06373526e-01 1.46515124e-01 7.05453167e+00 0.00000000e+00 0.00000000e+00

0.300000 -2.94285022e+01 8.92501089e+00 3.83531211e+01 7.28156880e+00 1.29016564e+00 2.06761320e-01 1.46515125e-01 7.28156880e+00 0.00000000e+00 0.00000000e+00

0.320000 -2.91730443e+01 9.18050913e+00 3.83531211e+01 7.53605351e+00 1.29079306e+00 2.07147434e-01 1.46515125e-01 7.53605351e+00 0.00000000e+00 0.00000000e+00

0.340000 -2.88790755e+01 9.47451905e+00 3.83531211e+01 7.82904841e+00 1.29142343e+00 2.07532091e-01 1.46515125e-01 7.82904841e+00 0.00000000e+00 0.00000000e+00

0.360000 -2.85287372e+01 9.82489692e+00 3.83531211e+01 8.17840945e+00 1.29205677e+00 2.07915581e-01 1.46515126e-01 8.17840945e+00 0.00000000e+00 0.00000000e+00

0.380000 -2.80935692e+01 1.02601010e+01 3.83531211e+01 8.61259440e+00 1.29269311e+00 2.08298310e-01 1.46515126e-01 8.61259440e+00 0.00000000e+00 0.00000000e+00

0.400000 -2.75290509e+01 1.08246479e+01 3.83531211e+01 9.17611946e+00 1.29333248e+00 2.08680886e-01 1.46515126e-01 9.17611946e+00 0.00000000e+00 0.00000000e+00

0.420000 -2.67666526e+01 1.15870635e+01 3.83531211e+01 9.93750924e+00 1.29397489e+00 2.09064280e-01 1.46515127e-01 9.93750924e+00 0.00000000e+00 0.00000000e+00

0.440000 -2.57017702e+01 1.26519464e+01 3.83531211e+01 1.10013607e+01 1.29462037e+00 2.09450136e-01 1.46515127e-01 1.10013607e+01 0.00000000e+00 0.00000000e+00

0.460000 -2.41749832e+01 1.41787094e+01 3.83531211e+01 1.25270840e+01 1.29526893e+00 2.09841378e-01 1.46515128e-01 1.25270840e+01 0.00000000e+00 0.00000000e+00

0.480000 -2.19430030e+01 1.64106292e+01 3.83531211e+01 1.47579500e+01 1.29592057e+00 2.10243408e-01 1.46515130e-01 1.47579500e+01 0.00000000e+00 0.00000000e+00

0.500000 -1.86340221e+01 1.97194963e+01 3.83531211e+01 1.80657394e+01 1.29657528e+00 2.10666468e-01 1.46515132e-01 1.80657394e+01 0.00000000e+00 0.00000000e+00

0.520000 -1.36791074e+01 2.46742230e+01 3.83531211e+01 2.30193445e+01 1.29723303e+00 2.11130345e-01 1.46515135e-01 2.30193445e+01 0.00000000e+00 0.00000000e+00

0.540000 -6.20601880e+00 3.21470329e+01 3.83531211e+01 3.04909503e+01 1.29789374e+00 2.11673747e-01 1.46515138e-01 3.04909503e+01 0.00000000e+00 0.00000000e+00

0.560000 5.12642737e+00 4.34791353e+01 3.83531211e+01 4.18216899e+01 1.29855728e+00 2.12372989e-01 1.46515145e-01 4.18216899e+01 0.00000000e+00 0.00000000e+00

0.580000 2.23874872e+01 6.07399758e+01 3.83531211e+01 5.90808580e+01 1.29922346e+00 2.13379198e-01 1.46515156e-01 5.90808580e+01 0.00000000e+00 0.00000000e+00

0.600000 4.87783986e+01 8.71313252e+01 3.83531211e+01 8.54699262e+01 1.29989191e+00 2.14991906e-01 1.46515172e-01 8.54699262e+01 0.00000000e+00 0.00000000e+00

0.620000 8.92565841e+01 1.27609474e+02 3.83531211e+01 1.25944592e+02 1.30056212e+00 2.17804697e-01 1.46515196e-01 1.25944592e+02 0.00000000e+00 0.00000000e+00

0.640000 1.51471398e+02 1.89824028e+02 3.83531211e+01 1.88153291e+02 1.30123321e+00 2.22988011e-01 1.46515234e-01 1.88153291e+02 0.00000000e+00 0.00000000e+00

0.660000 2.47131548e+02 2.85482648e+02 3.83531211e+01 2.83801396e+02 1.30190377e+00 2.32833267e-01 1.46515291e-01 2.83801396e+02 0.00000000e+00 0.00000000e+00

0.680000 3.93864846e+02 4.32207600e+02 3.83531211e+01 4.30506739e+02 1.30257184e+00 2.51773851e-01 1.46515379e-01 4.30506739e+02 0.00000000e+00 0.00000000e+00

0.700000 6.17390497e+02 6.55746329e+02 3.83531211e+01 6.54008341e+02 1.30323444e+00 2.88238682e-01 1.46515514e-01 6.54008341e+02 0.00000000e+00 0.00000000e+00

0.720000 9.53561687e+02 9.91937519e+02 3.83531211e+01 9.90129255e+02 1.30388751e+00 3.57860526e-01 1.46515717e-01 9.90129255e+02 0.00000000e+00 0.00000000e+00

0.740000 1.44866925e+03 1.48700064e+03 3.83531211e+01 1.48506090e+03 1.30452596e+00 4.88692464e-01 1.46516014e-01 1.48506090e+03 0.00000000e+00 0.00000000e+00

0.760000 2.15597168e+03 2.19412177e+03 3.83531211e+01 2.19194108e+03 1.30514403e+00 7.29028491e-01 1.46516439e-01 2.19194108e+03 0.00000000e+00 0.00000000e+00

0.780000 3.12634278e+03 3.16511133e+03 3.83531211e+01 3.16250102e+03 1.30573607e+00 1.15805877e+00 1.46517026e-01 3.16250102e+03 0.00000000e+00 0.00000000e+00

0.800000 4.40139717e+03 4.44016576e+03 3.83531211e+01 4.43681272e+03 1.30629875e+00 1.90022408e+00 1.46517793e-01 4.43681272e+03 0.00000000e+00 0.00000000e+00

solar cell parameters deduced from calculated IV-curve:

Voc = 0.551777 Volt

Jsc = 31.64213058 mA/cm2

FF = 64.7944 %

eta = 11.3127 %

V_MPP = 0.436387 Volt

J_MPP = 25.92359346 mA/cm2

# **ZnS nanoparticle (Eg = 3.54 eV)**

SCAPS 3.3.07 ELIS-UGent: Version scaps3307.exe, dated 27-01-2018, 10:11:02

Simulation of I-V curves

Single shot simulation # 1

Calculation started : 16-9-2021 at 14:15:22

problem definition file : c:\Program Files (x86)\Scaps3307\def\example CIGS.def

last saved: 01-09-2015 at 07:44:06

Calculation under illumination

Optical generation from internal SCAPS calculation

Illumination spectrum read from file

spectrum file :

C:\Program Files (x86)\Scaps3307\spectrum\AM1_5G 1 sun.spe last saved: 22-05-2009 at 09:59:48

Neutral density filter: ND 0.0000

Transmission of ND filter: 1.0000e+00

Power from spectrum: 100.0000 mW/cm2

Incident power on solar cell structure 100.0000 mW/cm2

Working point conditions

Temperature 300.00 K

Workpoint bias voltage 0.0000 Volt

Frequency 1.0000e+06 Hz

Voltage reference convention: voltage referred to right contact, voltage V applied to left contact

Current reference convention: current positive when entering the left contact

Power generated by the cell: = -V*I; power consumed by the cell = +V*I

v(V) jtot(mA/cm2) j_total_rec(mA/cm2) j_total_gen(mA/cm2) jbulk(mA/cm2) jifr(mA/cm2) jminor_left(mA/cm2) jminor_right(mA/cm2) j_SRH(mA/cm2) j_Radiative(mA/cm2) j_Auger(mA/cm2)

0.000000 -3.22872277e+01 6.09215769e+00 3.83790080e+01 5.36826404e+00 3.78511206e-01 2.14883373e-01 1.30499075e-01 5.36826404e+00 0.00000000e+00 0.00000000e+00

0.020000 -3.21703885e+01 6.20805566e+00 3.83790080e+01 5.47874482e+00 3.83342451e-01 2.15288789e-01 1.30679604e-01 5.47874482e+00 0.00000000e+00 0.00000000e+00

0.040000 -3.20491824e+01 6.32928128e+00 3.83790080e+01 5.59442848e+00 3.88297496e-01 2.15693418e-01 1.30861880e-01 5.59442848e+00 0.00000000e+00 0.00000000e+00

0.060000 -3.19224451e+01 6.45604340e+00 3.83790080e+01 5.71551599e+00 3.93380322e-01 2.16096633e-01 1.31050452e-01 5.71551599e+00 0.00000000e+00 0.00000000e+00

0.080000 -3.17897224e+01 6.58879135e+00 3.83790080e+01 5.84245126e+00 3.98595736e-01 2.16498522e-01 1.31245833e-01 5.84245126e+00 0.00000000e+00 0.00000000e+00

0.100000 -3.16502046e+01 6.72796263e+00 3.83790080e+01 5.97566601e+00 4.03947726e-01 2.16898938e-01 1.31449951e-01 5.97566601e+00 0.00000000e+00 0.00000000e+00

0.120000 -3.15038915e+01 6.87432429e+00 3.83790080e+01 6.11592185e+00 4.09443447e-01 2.17298467e-01 1.31660530e-01 6.11592185e+00 0.00000000e+00 0.00000000e+00

0.140000 -3.13497511e+01 7.02852421e+00 3.83790080e+01 6.26386016e+00 4.15087369e-01 2.17696946e-01 1.31879741e-01 6.26386016e+00 0.00000000e+00 0.00000000e+00

0.160000 -3.11868580e+01 7.19146716e+00 3.83790080e+01 6.42037931e+00 4.20885040e-01 2.18094465e-01 1.32108353e-01 6.42037931e+00 0.00000000e+00 0.00000000e+00

0.180000 -3.10140635e+01 7.36431851e+00 3.83790080e+01 6.58663801e+00 4.26842176e-01 2.18491119e-01 1.32347209e-01 6.58663801e+00 0.00000000e+00 0.00000000e+00

0.200000 -3.08298055e+01 7.54863561e+00 3.83790080e+01 6.76418672e+00 4.32964635e-01 2.18887001e-01 1.32597255e-01 6.76418672e+00 0.00000000e+00 0.00000000e+00

0.220000 -3.06319338e+01 7.74656989e+00 3.83790080e+01 6.95516975e+00 4.39258389e-01 2.19282206e-01 1.32859551e-01 6.95516975e+00 0.00000000e+00 0.00000000e+00

0.240000 -3.04170855e+01 7.96106130e+00 3.83790080e+01 7.16252082e+00 4.45727335e-01 2.19676621e-01 1.33136523e-01 7.16252082e+00 0.00000000e+00 0.00000000e+00

0.260000 -3.01815661e+01 8.19672046e+00 3.83790080e+01 7.39084073e+00 4.52382148e-01 2.20070807e-01 1.33426777e-01 7.39084073e+00 0.00000000e+00 0.00000000e+00

0.280000 -2.99186904e+01 8.45974060e+00 3.83790080e+01 7.64631613e+00 4.59226532e-01 2.20464618e-01 1.33733311e-01 7.64631613e+00 0.00000000e+00 0.00000000e+00

0.300000 -2.96191202e+01 8.75901885e+00 3.83790080e+01 7.93783812e+00 4.66263732e-01 2.20857963e-01 1.34059032e-01 7.93783812e+00 0.00000000e+00 0.00000000e+00

0.320000 -2.92708099e+01 9.10769440e+00 3.83790080e+01 8.27853387e+00 4.73506221e-01 2.21251486e-01 1.34402830e-01 8.27853387e+00 0.00000000e+00 0.00000000e+00

0.340000 -2.88554762e+01 9.52338545e+00 3.83790080e+01 8.68601594e+00 4.80955822e-01 2.21645044e-01 1.34768649e-01 8.68601594e+00 0.00000000e+00 0.00000000e+00

0.360000 -2.83487303e+01 1.00304800e+01 3.83790080e+01 9.18466452e+00 4.88617681e-01 2.22038843e-01 1.35158919e-01 9.18466452e+00 0.00000000e+00 0.00000000e+00

0.380000 -2.77174368e+01 1.06616066e+01 3.83790080e+01 9.80710310e+00 4.96493055e-01 2.22432965e-01 1.35577509e-01 9.80710310e+00 0.00000000e+00 0.00000000e+00

0.400000 -2.69182428e+01 1.14608731e+01 3.83790080e+01 1.05974275e+01 5.04591982e-01 2.22828169e-01 1.36025403e-01 1.05974275e+01 0.00000000e+00 0.00000000e+00

0.420000 -2.58936415e+01 1.24855106e+01 3.83790080e+01 1.16128622e+01 5.12917058e-01 2.23224988e-01 1.36506436e-01 1.16128622e+01 0.00000000e+00 0.00000000e+00

0.440000 -2.45694403e+01 1.38097277e+01 3.83790080e+01 1.29276064e+01 5.21472792e-01 2.23624425e-01 1.37024029e-01 1.29276064e+01 0.00000000e+00 0.00000000e+00

0.460000 -2.28506801e+01 1.55284713e+01 3.83790080e+01 1.46365971e+01 5.30264265e-01 2.24028081e-01 1.37581814e-01 1.46365971e+01 0.00000000e+00 0.00000000e+00

0.480000 -2.06164487e+01 1.77626610e+01 3.83790080e+01 1.68607410e+01 5.39297934e-01 2.24438568e-01 1.38183578e-01 1.68607410e+01 0.00000000e+00 0.00000000e+00

0.500000 -1.77113838e+01 2.06676720e+01 3.83790080e+01 1.97553957e+01 5.48582870e-01 2.24860260e-01 1.38833196e-01 1.97553957e+01 0.00000000e+00 0.00000000e+00

0.520000 -1.39299725e+01 2.44490124e+01 3.83790080e+01 2.35260447e+01 5.58132564e-01 2.25300674e-01 1.39534534e-01 2.35260447e+01 0.00000000e+00 0.00000000e+00

0.540000 -8.98733504e+00 2.93915806e+01 3.83790080e+01 2.84575487e+01 5.67967438e-01 2.25773078e-01 1.40291371e-01 2.84575487e+01 0.00000000e+00 0.00000000e+00

0.560000 -2.46843706e+00 3.59104391e+01 3.83790080e+01 3.49649121e+01 5.78118270e-01 2.26301391e-01 1.41107296e-01 3.49649121e+01 0.00000000e+00 0.00000000e+00

0.580000 6.25216604e+00 4.46310082e+01 3.83790080e+01 4.36734623e+01 5.88630820e-01 2.26929402e-01 1.41985661e-01 4.36734623e+01 0.00000000e+00 0.00000000e+00

0.600000 1.81283744e+01 5.65071869e+01 3.83790080e+01 5.55369474e+01 5.99572073e-01 2.27737903e-01 1.42929525e-01 5.55369474e+01 0.00000000e+00 0.00000000e+00

0.620000 3.46100828e+01 7.29890440e+01 3.83790080e+01 7.20051854e+01 6.11040957e-01 2.28876269e-01 1.43941324e-01 7.20051854e+01 0.00000000e+00 0.00000000e+00

0.640000 5.79012732e+01 9.62801627e+01 3.83790080e+01 9.52813470e+01 6.23170712e-01 2.30620597e-01 1.45024402e-01 9.52813470e+01 0.00000000e+00 0.00000000e+00

0.660000 9.13061531e+01 1.29685029e+02 3.83790080e+01 1.28669210e+02 6.36161205e-01 2.33477053e-01 1.46180362e-01 1.28669210e+02 0.00000000e+00 0.00000000e+00

0.680000 1.39765758e+02 1.78144636e+02 3.83790080e+01 1.77108571e+02 6.50289643e-01 2.38363965e-01 1.47410922e-01 1.77108571e+02 0.00000000e+00 0.00000000e+00

0.700000 2.10585558e+02 2.48964288e+02 3.83790080e+01 2.47902706e+02 6.65943746e-01 2.46921249e-01 1.48717044e-01 2.47902706e+02 0.00000000e+00 0.00000000e+00

0.720000 3.14384235e+02 3.52761851e+02 3.83790080e+01 3.51666076e+02 6.83658693e-01 2.62017803e-01 1.50098274e-01 3.51666076e+02 0.00000000e+00 0.00000000e+00

0.740000 4.66159527e+02 5.04539194e+02 3.83790080e+01 5.03394938e+02 7.04154805e-01 2.88550217e-01 1.51551677e-01 5.03394938e+02 0.00000000e+00 0.00000000e+00

0.760000 6.86314999e+02 7.24698289e+02 3.83790080e+01 7.23482207e+02 7.28380364e-01 3.34630767e-01 1.53070981e-01 7.23482207e+02 0.00000000e+00 0.00000000e+00

0.780000 1.00117273e+03 1.03954725e+03 3.83790080e+01 1.03822178e+03 7.57520259e-01 4.13294548e-01 1.54645662e-01 1.03822178e+03 0.00000000e+00 0.00000000e+00

0.800000 1.44174870e+03 1.48014063e+03 3.83790080e+01 1.47864660e+03 7.92970612e-01 5.44801521e-01 1.56258387e-01 1.47864660e+03 0.00000000e+00 0.00000000e+00

solar cell parameters deduced from calculated IV-curve:

Voc = 0.566201 Volt

Jsc = 32.28722768 mA/cm2

FF = 59.5037 %

eta = 10.8779 %

V_MPP = 0.423418 Volt

J_MPP = 25.69070683 mA/cm2

SCAPS 3.3.07 ELIS-UGent: Version scaps3307.exe, dated 27-01-2018, 10:11:02

Simulation of I-V curves

Single shot simulation # 2

Calculation started : 16-9-2021 at 14:15:50

problem definition file : c:\Program Files (x86)\Scaps3307\def\example CIGS.def

last saved: 01-09-2015 at 07:44:06

Calculation under illumination

Optical generation from internal SCAPS calculation

Illumination spectrum read from file

spectrum file :

C:\Program Files (x86)\Scaps3307\spectrum\AM1_5G 1 sun.spe last saved: 22-05-2009 at 09:59:48

Neutral density filter: ND 0.0000

Transmission of ND filter: 1.0000e+00

Power from spectrum: 100.0000 mW/cm2

Incident power on solar cell structure 100.0000 mW/cm2

Working point conditions

Temperature 300.00 K

Workpoint bias voltage 0.0000 Volt

Frequency 1.0000e+06 Hz

Voltage reference convention: voltage referred to right contact, voltage V applied to left contact

Current reference convention: current positive when entering the left contact

Power generated by the cell: = -V*I; power consumed by the cell = +V*I

v(V) jtot(mA/cm2) j_total_rec(mA/cm2) j_total_gen(mA/cm2) jbulk(mA/cm2) jifr(mA/cm2) jminor_left(mA/cm2) jminor_right(mA/cm2) j_SRH(mA/cm2) j_Radiative(mA/cm2) j_Auger(mA/cm2)

0.000000 -3.29216942e+01 5.45714569e+00 3.83790080e+01 4.74374665e+00 3.18645497e-01 2.10117066e-01 1.84636484e-01 4.74374665e+00 0.00000000e+00 0.00000000e+00

0.020000 -3.28246128e+01 5.55441929e+00 3.83790080e+01 4.83729469e+00 3.21870146e-01 2.10548948e-01 1.84705503e-01 4.83729469e+00 0.00000000e+00 0.00000000e+00

0.040000 -3.27230076e+01 5.65603401e+00 3.83790080e+01 4.93512098e+00 3.25159145e-01 2.10978944e-01 1.84774944e-01 4.93512098e+00 0.00000000e+00 0.00000000e+00

0.060000 -3.26167225e+01 5.76232939e+00 3.83790080e+01 5.03756307e+00 3.28514333e-01 2.11407177e-01 1.84844812e-01 5.03756307e+00 0.00000000e+00 0.00000000e+00

0.080000 -3.25053818e+01 5.87368131e+00 3.83790080e+01 5.14499482e+00 3.31937631e-01 2.11833749e-01 1.84915109e-01 5.14499482e+00 0.00000000e+00 0.00000000e+00

0.100000 -3.23885606e+01 5.99051523e+00 3.83790080e+01 5.25783959e+00 3.35431029e-01 2.12258767e-01 1.84985843e-01 5.25783959e+00 0.00000000e+00 0.00000000e+00

0.120000 -3.22657721e+01 6.11331779e+00 3.83790080e+01 5.37658184e+00 3.38996590e-01 2.12682345e-01 1.85057016e-01 5.37658184e+00 0.00000000e+00 0.00000000e+00

0.140000 -3.21364577e+01 6.24264650e+00 3.83790080e+01 5.50177680e+00 3.42636456e-01 2.13104602e-01 1.85128633e-01 5.50177680e+00 0.00000000e+00 0.00000000e+00

0.160000 -3.19999749e+01 6.37914404e+00 3.83790080e+01 5.63406483e+00 3.46352847e-01 2.13525664e-01 1.85200698e-01 5.63406483e+00 0.00000000e+00 0.00000000e+00

0.180000 -3.18555698e+01 6.52356742e+00 3.83790080e+01 5.77420048e+00 3.50148066e-01 2.13945664e-01 1.85273216e-01 5.77420048e+00 0.00000000e+00 0.00000000e+00

0.200000 -3.17023182e+01 6.67683748e+00 3.83790080e+01 5.92310204e+00 3.54024506e-01 2.14364743e-01 1.85346190e-01 5.92310204e+00 0.00000000e+00 0.00000000e+00

0.220000 -3.15390606e+01 6.84011533e+00 3.83790080e+01 6.08192801e+00 3.57984649e-01 2.14783054e-01 1.85419624e-01 6.08192801e+00 0.00000000e+00 0.00000000e+00

0.240000 -3.13642713e+01 7.01492941e+00 3.83790080e+01 6.25220406e+00 3.62031073e-01 2.15200759e-01 1.85493523e-01 6.25220406e+00 0.00000000e+00 0.00000000e+00

0.260000 -3.11758246e+01 7.20340071e+00 3.83790080e+01 6.43604833e+00 3.66166454e-01 2.15618034e-01 1.85567890e-01 6.43604833e+00 0.00000000e+00 0.00000000e+00

0.280000 -3.09706164e+01 7.40863295e+00 3.83790080e+01 6.63656158e+00 3.70393574e-01 2.16035073e-01 1.85642729e-01 6.63656158e+00 0.00000000e+00 0.00000000e+00

0.300000 -3.07439155e+01 7.63536351e+00 3.83790080e+01 6.85847806e+00 3.74715321e-01 2.16452093e-01 1.85718043e-01 6.85847806e+00 0.00000000e+00 0.00000000e+00

0.320000 -3.04882891e+01 7.89102379e+00 3.83790080e+01 7.10922591e+00 3.79134694e-01 2.16869344e-01 1.85793836e-01 7.10922591e+00 0.00000000e+00 0.00000000e+00

0.340000 -3.01919337e+01 8.18741052e+00 3.83790080e+01 7.40059848e+00 3.83654809e-01 2.17287123e-01 1.85870111e-01 7.40059848e+00 0.00000000e+00 0.00000000e+00

0.360000 -2.98361619e+01 8.54320986e+00 3.83790080e+01 7.75127829e+00 3.88278901e-01 2.17705798e-01 1.85946870e-01 7.75127829e+00 0.00000000e+00 0.00000000e+00

0.380000 -2.93916913e+01 8.98770756e+00 3.83790080e+01 8.19054726e+00 3.93010325e-01 2.18125858e-01 1.86024117e-01 8.19054726e+00 0.00000000e+00 0.00000000e+00

0.400000 -2.88131956e+01 9.56622426e+00 3.83790080e+01 8.76372187e+00 3.97852557e-01 2.18547985e-01 1.86101852e-01 8.76372187e+00 0.00000000e+00 0.00000000e+00

0.420000 -2.80312784e+01 1.03481496e+01 3.83790080e+01 9.54018718e+00 4.02809192e-01 2.18973197e-01 1.86180080e-01 9.54018718e+00 0.00000000e+00 0.00000000e+00

0.440000 -2.69404367e+01 1.14389922e+01 3.83790080e+01 1.06254464e+01 4.07883937e-01 2.19403118e-01 1.86258801e-01 1.06254464e+01 0.00000000e+00 0.00000000e+00

0.460000 -2.53806249e+01 1.29987809e+01 3.83790080e+01 1.21795218e+01 4.13080597e-01 2.19840493e-01 1.86338014e-01 1.21795218e+01 0.00000000e+00 0.00000000e+00

0.480000 -2.31087972e+01 1.52705662e+01 3.83790080e+01 1.44454552e+01 4.18403052e-01 2.20290184e-01 1.86417719e-01 1.44454552e+01 0.00000000e+00 0.00000000e+00

0.500000 -1.97545021e+01 1.86247735e+01 3.83790080e+01 1.77936592e+01 4.23855207e-01 2.20761143e-01 1.86497917e-01 1.77936592e+01 0.00000000e+00 0.00000000e+00

0.520000 -1.47510342e+01 2.36281095e+01 3.83790080e+01 2.27908197e+01 4.29440923e-01 2.21270293e-01 1.86578598e-01 2.27908197e+01 0.00000000e+00 0.00000000e+00

0.540000 -7.22858636e+00 3.11503762e+01 3.83790080e+01 3.03067023e+01 4.35163892e-01 2.21850227e-01 1.86659754e-01 3.03067023e+01 0.00000000e+00 0.00000000e+00

0.560000 4.15117411e+00 4.25299627e+01 3.83790080e+01 4.16796294e+01 4.41027443e-01 2.22564467e-01 1.86741372e-01 4.16796294e+01 0.00000000e+00 0.00000000e+00

0.580000 2.14513224e+01 5.98301597e+01 3.83790080e+01 5.89727644e+01 4.47034234e-01 2.23537701e-01 1.86823430e-01 5.89727644e+01 0.00000000e+00 0.00000000e+00

0.600000 4.78559281e+01 8.62347453e+01 3.83790080e+01 8.53696385e+01 4.53185721e-01 2.25015215e-01 1.86905885e-01 8.53696385e+01 0.00000000e+00 0.00000000e+00

0.620000 8.82727406e+01 1.26651511e+02 3.83790080e+01 1.25777560e+02 4.59481598e-01 2.27480060e-01 1.86988697e-01 1.25777560e+02 0.00000000e+00 0.00000000e+00

0.640000 1.50234255e+02 1.88612736e+02 3.83790080e+01 1.87727867e+02 4.65918534e-01 2.31878453e-01 1.87071770e-01 1.87727867e+02 0.00000000e+00 0.00000000e+00

0.660000 2.45184503e+02 2.83561372e+02 3.83790080e+01 2.82661679e+02 4.72488778e-01 2.40049298e-01 1.87154982e-01 2.82661679e+02 0.00000000e+00 0.00000000e+00

0.680000 3.90179945e+02 4.28548312e+02 3.83790080e+01 4.27626377e+02 4.79178298e-01 2.55518125e-01 1.87238138e-01 4.27626377e+02 0.00000000e+00 0.00000000e+00

0.700000 6.09758623e+02 6.48140269e+02 3.83790080e+01 6.47182075e+02 4.85964626e-01 2.84908808e-01 1.87320975e-01 6.47182075e+02 0.00000000e+00 0.00000000e+00

0.720000 9.37482677e+02 9.75882897e+02 3.83790080e+01 9.74862364e+02 4.92816303e-01 3.40314011e-01 1.87403129e-01 9.74862364e+02 0.00000000e+00 0.00000000e+00

0.740000 1.41539005e+03 1.45391474e+03 3.83790080e+01 1.45278458e+03 4.99693627e-01 4.42990134e-01 1.87484156e-01 1.45278458e+03 0.00000000e+00 0.00000000e+00

0.760000 2.09101635e+03 2.12924047e+03 3.83790080e+01 2.12791769e+03 5.06557666e-01 6.28659239e-01 1.87563590e-01 2.12791769e+03 0.00000000e+00 0.00000000e+00

0.780000 3.00695245e+03 3.04558342e+03 3.83790080e+01 3.04392820e+03 5.13370937e-01 9.54210507e-01 1.87640987e-01 3.04392820e+03 0.00000000e+00 0.00000000e+00

0.800000 4.19691664e+03 4.23481266e+03 3.83790080e+01 4.23259846e+03 5.20124050e-01 1.50635912e+00 1.87716100e-01 4.23259846e+03 0.00000000e+00 0.00000000e+00

solar cell parameters deduced from calculated IV-curve:

Voc = 0.553451 Volt

Jsc = 32.92169419 mA/cm2

FF = 65.0685 %

eta = 11.8558 %

V_MPP = 0.437441 Volt

J_MPP = 27.10268490 mA/cm2

SCAPS 3.3.07 ELIS-UGent: Version scaps3307.exe, dated 27-01-2018, 10:11:02

Simulation of I-V curves

Single shot simulation # 3

Calculation started : 16-9-2021 at 14:16:10

problem definition file : c:\Program Files (x86)\Scaps3307\def\example CIGS.def

last saved: 01-09-2015 at 07:44:06

Calculation under illumination

Optical generation from internal SCAPS calculation

Illumination spectrum read from file

spectrum file :

C:\Program Files (x86)\Scaps3307\spectrum\AM1_5G 1 sun.spe last saved: 22-05-2009 at 09:59:48

Neutral density filter: ND 0.0000

Transmission of ND filter: 1.0000e+00

Power from spectrum: 100.0000 mW/cm2

Incident power on solar cell structure 100.0000 mW/cm2

Working point conditions

Temperature 300.00 K

Workpoint bias voltage 0.0000 Volt

Frequency 1.0000e+06 Hz

Voltage reference convention: voltage referred to right contact, voltage V applied to left contact

Current reference convention: current positive when entering the left contact

Power generated by the cell: = -V*I; power consumed by the cell = +V*I

v(V) jtot(mA/cm2) j_total_rec(mA/cm2) j_total_gen(mA/cm2) jbulk(mA/cm2) jifr(mA/cm2) jminor_left(mA/cm2) jminor_right(mA/cm2) j_SRH(mA/cm2) j_Radiative(mA/cm2) j_Auger(mA/cm2)

0.000000 -3.31084266e+01 5.27040711e+00 3.83790080e+01 4.78715779e+00 8.51591037e-02 2.07655826e-01 1.90434394e-01 4.78715779e+00 0.00000000e+00 0.00000000e+00

0.020000 -3.30117591e+01 5.36729315e+00 3.83790080e+01 4.88327539e+00 8.54502654e-02 2.08132865e-01 1.90434627e-01 4.88327539e+00 0.00000000e+00 0.00000000e+00

0.040000 -3.29104524e+01 5.46861120e+00 3.83790080e+01 4.98382529e+00 8.57436620e-02 2.08607387e-01 1.90434859e-01 4.98382529e+00 0.00000000e+00 0.00000000e+00

0.060000 -3.28043740e+01 5.57470190e+00 3.83790080e+01 5.08914797e+00 8.60393196e-02 2.09079517e-01 1.90435092e-01 5.08914797e+00 0.00000000e+00 0.00000000e+00

0.080000 -3.26931461e+01 5.68594319e+00 3.83790080e+01 5.19962124e+00 8.63372646e-02 2.09549363e-01 1.90435324e-01 5.19962124e+00 0.00000000e+00 0.00000000e+00

0.100000 -3.25763381e+01 5.80276564e+00 3.83790080e+01 5.31567552e+00 8.66375237e-02 2.10017040e-01 1.90435557e-01 5.31567552e+00 0.00000000e+00 0.00000000e+00

0.120000 -3.24534540e+01 5.92566547e+00 3.83790080e+01 5.43780689e+00 8.69401240e-02 2.10482665e-01 1.90435790e-01 5.43780689e+00 0.00000000e+00 0.00000000e+00

0.140000 -3.23239225e+01 6.05521414e+00 3.83790080e+01 5.56658666e+00 8.72450931e-02 2.10946366e-01 1.90436022e-01 5.56658666e+00 0.00000000e+00 0.00000000e+00

0.160000 -3.21870843e+01 6.19207100e+00 3.83790080e+01 5.70267401e+00 8.75524588e-02 2.11408273e-01 1.90436255e-01 5.70267401e+00 0.00000000e+00 0.00000000e+00

0.180000 -3.20421665e+01 6.33700923e+00 3.83790080e+01 5.84684197e+00 8.78622496e-02 2.11868526e-01 1.90436488e-01 5.84684197e+00 0.00000000e+00 0.00000000e+00

0.200000 -3.18882369e+01 6.49096139e+00 3.83790080e+01 6.00002290e+00 8.81744945e-02 2.12327273e-01 1.90436720e-01 6.00002290e+00 0.00000000e+00 0.00000000e+00

0.220000 -3.17241341e+01 6.65508868e+00 3.83790080e+01 6.16337784e+00 8.84892226e-02 2.12784672e-01 1.90436953e-01 6.16337784e+00 0.00000000e+00 0.00000000e+00

0.240000 -3.15483558e+01 6.83089411e+00 3.83790080e+01 6.33840957e+00 8.88064638e-02 2.13240893e-01 1.90437186e-01 6.33840957e+00 0.00000000e+00 0.00000000e+00

0.260000 -3.13588504e+01 7.02042916e+00 3.83790080e+01 6.52716937e+00 8.91262483e-02 2.13696117e-01 1.90437419e-01 6.52716937e+00 0.00000000e+00 0.00000000e+00

0.280000 -3.11526426e+01 7.22666920e+00 3.83790080e+01 6.73263239e+00 8.94486069e-02 2.14150545e-01 1.90437652e-01 6.73263239e+00 0.00000000e+00 0.00000000e+00

0.300000 -3.09251924e+01 7.45415381e+00 3.83790080e+01 6.95933795e+00 8.97735707e-02 2.14604401e-01 1.90437885e-01 6.95933795e+00 0.00000000e+00 0.00000000e+00

0.320000 -3.06693477e+01 7.71003469e+00 3.83790080e+01 7.21443746e+00 9.01011712e-02 2.15057941e-01 1.90438118e-01 7.21443746e+00 0.00000000e+00 0.00000000e+00

0.340000 -3.03736731e+01 8.00574592e+00 3.83790080e+01 7.50936465e+00 9.04314402e-02 2.15511472e-01 1.90438351e-01 7.50936465e+00 0.00000000e+00 0.00000000e+00

0.360000 -3.00198941e+01 8.35956024e+00 3.83790080e+01 7.86239187e+00 9.07644096e-02 2.15965376e-01 1.90438584e-01 7.86239187e+00 0.00000000e+00 0.00000000e+00

0.380000 -2.95791589e+01 8.80032709e+00 3.83790080e+01 8.30236799e+00 9.11001115e-02 2.16420165e-01 1.90438818e-01 8.30236799e+00 0.00000000e+00 0.00000000e+00

0.400000 -2.90066519e+01 9.37285912e+00 3.83790080e+01 8.87410493e+00 9.14385771e-02 2.16876561e-01 1.90439051e-01 8.87410493e+00 0.00000000e+00 0.00000000e+00

0.420000 -2.82336184e+01 1.01459074e+01 3.83790080e+01 9.64635266e+00 9.17798371e-02 2.17335670e-01 1.90439285e-01 9.64635266e+00 0.00000000e+00 0.00000000e+00

0.440000 -2.71551551e+01 1.12243708e+01 3.83790080e+01 1.07240080e+01 9.21239203e-02 2.17799288e-01 1.90439519e-01 1.07240080e+01 0.00000000e+00 0.00000000e+00

0.460000 -2.56113469e+01 1.27681571e+01 3.83790080e+01 1.22669760e+01 9.24708526e-02 2.18270511e-01 1.90439751e-01 1.22669760e+01 0.00000000e+00 0.00000000e+00

0.480000 -2.33583229e+01 1.50211273e+01 3.83790080e+01 1.45191118e+01 9.28206550e-02 2.18754931e-01 1.90439984e-01 1.45191118e+01 0.00000000e+00 0.00000000e+00

0.500000 -2.00239648e+01 1.83553856e+01 3.83790080e+01 1.78525090e+01 9.31733404e-02 2.19262988e-01 1.90440218e-01 1.78525090e+01 0.00000000e+00 0.00000000e+00

0.520000 -1.50396753e+01 2.33395126e+01 3.83790080e+01 2.28357286e+01 9.35289096e-02 2.19814639e-01 1.90440449e-01 2.28357286e+01 0.00000000e+00 0.00000000e+00

0.540000 -7.53420941e+00 3.08447435e+01 3.83790080e+01 3.03399668e+01 9.38873431e-02 2.20448648e-01 1.90440684e-01 3.03399668e+01 0.00000000e+00 0.00000000e+00

0.560000 3.83252580e+00 4.22112069e+01 3.83790080e+01 4.17052763e+01 9.42485904e-02 2.21241097e-01 1.90440917e-01 4.17052763e+01 0.00000000e+00 0.00000000e+00

0.580000 2.11295183e+01 5.95081000e+01 3.83790080e+01 5.90007041e+01 9.46125517e-02 2.22342181e-01 1.90441150e-01 5.90007041e+01 0.00000000e+00 0.00000000e+00

0.600000 4.75595431e+01 8.59383645e+01 3.83790080e+01 8.54288952e+01 9.49790469e-02 2.24048939e-01 1.90441385e-01 8.54288952e+01 0.00000000e+00 0.00000000e+00

0.620000 8.80815614e+01 1.26460342e+02 3.83790080e+01 1.25947604e+02 9.53477859e-02 2.26949107e-01 1.90441617e-01 1.25947604e+02 0.00000000e+00 0.00000000e+00

0.640000 1.50345960e+02 1.88724471e+02 3.83790080e+01 1.88206111e+02 9.57182898e-02 2.32200224e-01 1.90441850e-01 1.88206111e+02 0.00000000e+00 0.00000000e+00

0.660000 2.46057643e+02 2.84434592e+02 3.83790080e+01 2.83905994e+02 9.60898112e-02 2.42066360e-01 1.90442083e-01 2.83905994e+02 0.00000000e+00 0.00000000e+00

0.680000 3.92831078e+02 4.31199584e+02 3.83790080e+01 4.30651756e+02 9.64612159e-02 2.60924554e-01 1.90442315e-01 4.30651756e+02 0.00000000e+00 0.00000000e+00

0.700000 6.16353898e+02 6.54735650e+02 3.83790080e+01 6.54151286e+02 9.68308347e-02 2.97090768e-01 1.90442546e-01 6.54151286e+02 0.00000000e+00 0.00000000e+00

0.720000 9.52414918e+02 9.90816779e+02 3.83790080e+01 9.90163163e+02 9.71963924e-02 3.65976812e-01 1.90442776e-01 9.90163163e+02 0.00000000e+00 0.00000000e+00

0.740000 1.44718270e+03 1.48553991e+03 3.83790080e+01 1.48475669e+03 9.75550317e-02 4.95217674e-01 1.90443005e-01 1.48475669e+03 0.00000000e+00 0.00000000e+00

0.760000 2.15371097e+03 2.19188673e+03 3.83790080e+01 2.19086604e+03 9.79035596e-02 7.32347218e-01 1.90443231e-01 2.19086604e+03 0.00000000e+00 0.00000000e+00

0.780000 3.12259015e+03 3.16138325e+03 3.83790080e+01 3.15993933e+03 9.82388826e-02 1.15524032e+00 1.90443455e-01 3.15993933e+03 0.00000000e+00 0.00000000e+00

0.800000 4.39511564e+03 4.43390501e+03 3.83790080e+01 4.43172985e+03 9.85592720e-02 1.88615822e+00 1.90443677e-01 4.43172985e+03 0.00000000e+00 0.00000000e+00

solar cell parameters deduced from calculated IV-curve:

Voc = 0.553972 Volt

Jsc = 33.10842657 mA/cm2

FF = 65.1497 %

eta = 11.9492 %

V_MPP = 0.438262 Volt

J_MPP = 27.26498252 mA/cm2

# **ZnS QDs (Eg = 3.95 eV)**

SCAPS 3.3.07 ELIS-UGent: Version scaps3307.exe, dated 27-01-2018, 10:11:02

Simulation of I-V curves

Single shot simulation # 4

Calculation started : 16-9-2021 at 14:17:08

problem definition file : c:\Program Files (x86)\Scaps3307\def\example CIGS.def

last saved: 01-09-2015 at 07:44:06

Calculation under illumination

Optical generation from internal SCAPS calculation

Illumination spectrum read from file

spectrum file :

C:\Program Files (x86)\Scaps3307\spectrum\AM1_5G 1 sun.spe last saved: 22-05-2009 at 09:59:48

Neutral density filter: ND 0.0000

Transmission of ND filter: 1.0000e+00

Power from spectrum: 100.0000 mW/cm2

Incident power on solar cell structure 100.0000 mW/cm2

Working point conditions

Temperature 300.00 K

Workpoint bias voltage 0.0000 Volt

Frequency 1.0000e+06 Hz

Voltage reference convention: voltage referred to right contact, voltage V applied to left contact

Current reference convention: current positive when entering the left contact

Power generated by the cell: = -V*I; power consumed by the cell = +V*I

v(V) jtot(mA/cm2) j_total_rec(mA/cm2) j_total_gen(mA/cm2) jbulk(mA/cm2) jifr(mA/cm2) jminor_left(mA/cm2) jminor_right(mA/cm2) j_SRH(mA/cm2) j_Radiative(mA/cm2) j_Auger(mA/cm2)

0.000000 -3.22774775e+01 6.10205703e+00 3.83791535e+01 5.37588318e+00 3.51642333e-01 2.14918948e-01 1.59612566e-01 5.37588318e+00 0.00000000e+00 0.00000000e+00

0.020000 -3.21607334e+01 6.21786509e+00 3.83791535e+01 5.48658193e+00 3.56346595e-01 2.15323578e-01 1.59612984e-01 5.48658193e+00 0.00000000e+00 0.00000000e+00

0.040000 -3.20398264e+01 6.33907312e+00 3.83791535e+01 5.60255912e+00 3.61172950e-01 2.15727647e-01 1.59613406e-01 5.60255912e+00 0.00000000e+00 0.00000000e+00

0.060000 -3.19129399e+01 6.46570455e+00 3.83791535e+01 5.72383510e+00 3.66125700e-01 2.16129907e-01 1.59613852e-01 5.72383510e+00 0.00000000e+00 0.00000000e+00

0.080000 -3.17804157e+01 6.59842382e+00 3.83791535e+01 5.85106780e+00 3.71210542e-01 2.16531159e-01 1.59614313e-01 5.85106780e+00 0.00000000e+00 0.00000000e+00

0.100000 -3.16411155e+01 6.73757987e+00 3.83791535e+01 5.98460246e+00 3.76431657e-01 2.16930952e-01 1.59614799e-01 5.98460246e+00 0.00000000e+00 0.00000000e+00

0.120000 -3.14948304e+01 6.88389091e+00 3.83791535e+01 6.12515079e+00 3.81795096e-01 2.17329715e-01 1.59615307e-01 6.12515079e+00 0.00000000e+00 0.00000000e+00

0.140000 -3.13404586e+01 7.03798408e+00 3.83791535e+01 6.27333565e+00 3.87305347e-01 2.17727241e-01 1.59615842e-01 6.27333565e+00 0.00000000e+00 0.00000000e+00

0.160000 -3.11775808e+01 7.20091651e+00 3.83791535e+01 6.43020641e+00 3.92969688e-01 2.18124012e-01 1.59616402e-01 6.43020641e+00 0.00000000e+00 0.00000000e+00

0.180000 -3.10047611e+01 7.37379077e+00 3.83791535e+01 6.59686055e+00 3.98793313e-01 2.18519915e-01 1.59616995e-01 6.59686055e+00 0.00000000e+00 0.00000000e+00

0.200000 -3.08204365e+01 7.55818094e+00 3.83791535e+01 6.77486596e+00 4.04782316e-01 2.18915039e-01 1.59617622e-01 6.77486596e+00 0.00000000e+00 0.00000000e+00

0.220000 -3.06224068e+01 7.75626401e+00 3.83791535e+01 6.96639329e+00 4.10942957e-01 2.19309476e-01 1.59618286e-01 6.96639329e+00 0.00000000e+00 0.00000000e+00

0.240000 -3.04076063e+01 7.97112412e+00 3.83791535e+01 7.17452018e+00 4.17281626e-01 2.19703320e-01 1.59618993e-01 7.17452018e+00 0.00000000e+00 0.00000000e+00

0.260000 -3.01713854e+01 8.20710314e+00 3.83791535e+01 7.40358345e+00 4.23803428e-01 2.20096517e-01 1.59619750e-01 7.40358345e+00 0.00000000e+00 0.00000000e+00

0.280000 -2.99078650e+01 8.47076224e+00 3.83791535e+01 7.66013421e+00 4.30517965e-01 2.20489512e-01 1.59620556e-01 7.66013421e+00 0.00000000e+00 0.00000000e+00

0.300000 -2.96076619e+01 8.77110681e+00 3.83791535e+01 7.95317300e+00 4.37430165e-01 2.20882223e-01 1.59621421e-01 7.95317300e+00 0.00000000e+00 0.00000000e+00

0.320000 -2.92579593e+01 9.12100891e+00 3.83791535e+01 8.29556488e+00 4.44546870e-01 2.21274811e-01 1.59622353e-01 8.29556488e+00 0.00000000e+00 0.00000000e+00

0.340000 -2.88408377e+01 9.53827079e+00 3.83791535e+01 8.70510619e+00 4.51873865e-01 2.21667384e-01 1.59623358e-01 8.70510619e+00 0.00000000e+00 0.00000000e+00

0.360000 -2.83320518e+01 1.00473815e+01 3.83791535e+01 9.20627801e+00 4.59418805e-01 2.22060260e-01 1.59624446e-01 9.20627801e+00 0.00000000e+00 0.00000000e+00

0.380000 -2.76983511e+01 1.06806242e+01 3.83791535e+01 9.83136376e+00 4.67181543e-01 2.22453299e-01 1.59625635e-01 9.83136376e+00 0.00000000e+00 0.00000000e+00

0.400000 -2.68969008e+01 1.14823665e+01 3.83791535e+01 1.06247138e+01 4.75178135e-01 2.22847628e-01 1.59626928e-01 1.06247138e+01 0.00000000e+00 0.00000000e+00

0.420000 -2.58702807e+01 1.25087803e+01 3.83791535e+01 1.16425025e+01 4.83406083e-01 2.23243328e-01 1.59628349e-01 1.16425025e+01 0.00000000e+00 0.00000000e+00

0.440000 -2.45451603e+01 1.38338114e+01 3.83791535e+01 1.29586638e+01 4.91875967e-01 2.23641681e-01 1.59629909e-01 1.29586638e+01 0.00000000e+00 0.00000000e+00

0.460000 -2.28274978e+01 1.55514688e+01 3.83791535e+01 1.46671980e+01 5.00594823e-01 2.24044276e-01 1.59631628e-01 1.46671980e+01 0.00000000e+00 0.00000000e+00

0.480000 -2.05975887e+01 1.77816521e+01 3.83791535e+01 1.68879914e+01 5.09573325e-01 2.24453820e-01 1.59633525e-01 1.68879914e+01 0.00000000e+00 0.00000000e+00

0.500000 -1.77011974e+01 2.06777628e+01 3.83791535e+01 1.97744374e+01 5.18815412e-01 2.24874300e-01 1.59635633e-01 1.97744374e+01 0.00000000e+00 0.00000000e+00

0.520000 -1.39339849e+01 2.44449976e+01 3.83791535e+01 2.35317009e+01 5.28345119e-01 2.25313633e-01 1.59637970e-01 2.35317009e+01 0.00000000e+00 0.00000000e+00

0.540000 -9.01123296e+00 2.93677173e+01 3.83791535e+01 2.84441097e+01 5.38182164e-01 2.25784917e-01 1.59640569e-01 2.84441097e+01 0.00000000e+00 0.00000000e+00

0.560000 -2.51734730e+00 3.58616030e+01 3.83791535e+01 3.49272863e+01 5.48361113e-01 2.26312118e-01 1.59643458e-01 3.49272863e+01 0.00000000e+00 0.00000000e+00

0.580000 6.17445308e+00 4.45534297e+01 3.83791535e+01 4.36079136e+01 5.58930355e-01 2.26939008e-01 1.59646676e-01 4.36079136e+01 0.00000000e+00 0.00000000e+00

0.600000 1.80208920e+01 5.63998447e+01 3.83791535e+01 5.54424901e+01 5.69958074e-01 2.27746350e-01 1.59650258e-01 5.54424901e+01 0.00000000e+00 0.00000000e+00

0.620000 3.44750884e+01 7.28540546e+01 3.83791535e+01 7.18839718e+01 5.81544997e-01 2.28883587e-01 1.59654245e-01 7.18839718e+01 0.00000000e+00 0.00000000e+00

0.640000 5.77358198e+01 9.61148490e+01 3.83791535e+01 9.51307322e+01 5.93831605e-01 2.30626489e-01 1.59658683e-01 9.51307322e+01 0.00000000e+00 0.00000000e+00

0.660000 9.11149230e+01 1.29493945e+02 3.83791535e+01 1.28493786e+02 6.07013809e-01 2.33481370e-01 1.59663612e-01 1.28493786e+02 0.00000000e+00 0.00000000e+00

0.680000 1.39551684e+02 1.77930705e+02 3.83791535e+01 1.76911298e+02 6.21371982e-01 2.38366326e-01 1.59669064e-01 1.76911298e+02 0.00000000e+00 0.00000000e+00

0.700000 2.10351964e+02 2.48730836e+02 3.83791535e+01 2.47686945e+02 6.37294939e-01 2.46921099e-01 1.59675104e-01 2.47686945e+02 0.00000000e+00 0.00000000e+00

0.720000 3.14129630e+02 3.52508889e+02 3.83791535e+01 3.51431875e+02 6.55317648e-01 2.62014970e-01 1.59681760e-01 3.51431875e+02 0.00000000e+00 0.00000000e+00

0.740000 4.65898545e+02 5.04278364e+02 3.83791535e+01 5.03153969e+02 6.76163222e-01 2.88542689e-01 1.59689055e-01 5.03153969e+02 0.00000000e+00 0.00000000e+00

0.760000 6.86047256e+02 7.24430716e+02 3.83791535e+01 7.23235625e+02 7.00776200e-01 3.34618095e-01 1.59696987e-01 7.23235625e+02 0.00000000e+00 0.00000000e+00

0.780000 1.00090442e+03 1.03927906e+03 3.83791535e+01 1.03797574e+03 7.30339922e-01 4.13275736e-01 1.59705522e-01 1.03797574e+03 0.00000000e+00 0.00000000e+00

0.800000 1.44148496e+03 1.47987706e+03 3.83791535e+01 1.47840632e+03 7.66245808e-01 5.44775567e-01 1.59714577e-01 1.47840632e+03 0.00000000e+00 0.00000000e+00

solar cell parameters deduced from calculated IV-curve:

Voc = 0.566340 Volt

Jsc = 32.27747751 mA/cm2

FF = 59.4524 %

eta = 10.8679 %

V_MPP = 0.423301 Volt

J_MPP = 25.67420664 mA/cm2

SCAPS 3.3.07 ELIS-UGent: Version scaps3307.exe, dated 27-01-2018, 10:11:02

Simulation of I-V curves

Single shot simulation # 5

Calculation started : 16-9-2021 at 14:18:14

problem definition file : c:\Program Files (x86)\Scaps3307\def\example CIGS.def

last saved: 01-09-2015 at 07:44:06

Calculation under illumination

Optical generation from internal SCAPS calculation

Illumination spectrum read from file

spectrum file :

C:\Program Files (x86)\Scaps3307\spectrum\AM1_5G 1 sun.spe last saved: 22-05-2009 at 09:59:48

Neutral density filter: ND 0.0000

Transmission of ND filter: 1.0000e+00

Power from spectrum: 100.0000 mW/cm2

Incident power on solar cell structure 100.0000 mW/cm2

Working point conditions

Temperature 300.00 K

Workpoint bias voltage 0.0000 Volt

Frequency 1.0000e+06 Hz

Voltage reference convention: voltage referred to right contact, voltage V applied to left contact

Current reference convention: current positive when entering the left contact

Power generated by the cell: = -V*I; power consumed by the cell = +V*I

v(V) jtot(mA/cm2) j_total_rec(mA/cm2) j_total_gen(mA/cm2) jbulk(mA/cm2) jifr(mA/cm2) jminor_left(mA/cm2) jminor_right(mA/cm2) j_SRH(mA/cm2) j_Radiative(mA/cm2) j_Auger(mA/cm2)

0.000000 -3.29544727e+01 5.42451219e+00 3.83791535e+01 4.74481642e+00 3.09736829e-01 2.10117736e-01 1.59841207e-01 4.74481642e+00 0.00000000e+00 0.00000000e+00

0.020000 -3.28572243e+01 5.52174693e+00 3.83791535e+01 4.83841142e+00 3.12943978e-01 2.10549619e-01 1.59841910e-01 4.83841142e+00 0.00000000e+00 0.00000000e+00

0.040000 -3.27556443e+01 5.62332698e+00 3.83791535e+01 4.93628948e+00 3.16215248e-01 2.10979643e-01 1.59842616e-01 4.93628948e+00 0.00000000e+00 0.00000000e+00

0.060000 -3.26493807e+01 5.72959061e+00 3.83791535e+01 5.03878689e+00 3.19552489e-01 2.11407904e-01 1.59843327e-01 5.03878689e+00 0.00000000e+00 0.00000000e+00

0.080000 -3.25382938e+01 5.84091543e+00 3.83791535e+01 5.14627926e+00 3.22957604e-01 2.11834528e-01 1.59844041e-01 5.14627926e+00 0.00000000e+00 0.00000000e+00

0.100000 -3.24212483e+01 5.95772312e+00 3.83791535e+01 5.25918618e+00 3.26432619e-01 2.12259554e-01 1.59844761e-01 5.25918618e+00 0.00000000e+00 0.00000000e+00

0.120000 -3.22987288e+01 6.08050616e+00 3.83791535e+01 5.37799795e+00 3.29979534e-01 2.12683188e-01 1.59845484e-01 5.37799795e+00 0.00000000e+00 0.00000000e+00

0.140000 -3.21691542e+01 6.20981732e+00 3.83791535e+01 5.50326511e+00 3.33600549e-01 2.13105455e-01 1.59846211e-01 5.50326511e+00 0.00000000e+00 0.00000000e+00

0.160000 -3.20326678e+01 6.34630369e+00 3.83791535e+01 5.63563236e+00 3.37297833e-01 2.13526553e-01 1.59846943e-01 5.63563236e+00 0.00000000e+00 0.00000000e+00

0.180000 -3.18882500e+01 6.49072159e+00 3.83791535e+01 5.77585362e+00 3.41073701e-01 2.13946591e-01 1.59847680e-01 5.77585362e+00 0.00000000e+00 0.00000000e+00

0.200000 -3.17353053e+01 6.64399351e+00 3.83791535e+01 5.92484883e+00 3.44930520e-01 2.14365734e-01 1.59848420e-01 5.92484883e+00 0.00000000e+00 0.00000000e+00

0.220000 -3.15720431e+01 6.80727912e+00 3.83791535e+01 6.08377507e+00 3.48870805e-01 2.14784087e-01 1.59849165e-01 6.08377507e+00 0.00000000e+00 0.00000000e+00

0.240000 -3.13972338e+01 6.98210857e+00 3.83791535e+01 6.25415971e+00 3.52897112e-01 2.15201836e-01 1.59849914e-01 6.25415971e+00 0.00000000e+00 0.00000000e+00

0.260000 -3.12087682e+01 7.17060329e+00 3.83791535e+01 6.43812135e+00 3.57012111e-01 2.15619158e-01 1.59850668e-01 6.43812135e+00 0.00000000e+00 0.00000000e+00

0.280000 -3.10035305e+01 7.37586717e+00 3.83791535e+01 6.63876092e+00 3.61218579e-01 2.16036247e-01 1.59851426e-01 6.63876092e+00 0.00000000e+00 0.00000000e+00

0.300000 -3.07767901e+01 7.60263718e+00 3.83791535e+01 6.86081228e+00 3.65519398e-01 2.16453319e-01 1.59852189e-01 6.86081228e+00 0.00000000e+00 0.00000000e+00

0.320000 -3.05206296e+01 7.85834282e+00 3.83791535e+01 7.11170166e+00 3.69917598e-01 2.16870602e-01 1.59852956e-01 7.11170166e+00 0.00000000e+00 0.00000000e+00

0.340000 -3.02247066e+01 8.15478106e+00 3.83791535e+01 7.40322269e+00 3.74416185e-01 2.17288463e-01 1.59853727e-01 7.40322269e+00 0.00000000e+00 0.00000000e+00

0.360000 -2.98688885e+01 8.51063308e+00 3.83791535e+01 7.75405288e+00 3.79018492e-01 2.17707201e-01 1.59854503e-01 7.75405288e+00 0.00000000e+00 0.00000000e+00

0.380000 -2.94237911e+01 8.95518178e+00 3.83791535e+01 8.19347131e+00 3.83727879e-01 2.18127305e-01 1.59855283e-01 8.19347131e+00 0.00000000e+00 0.00000000e+00

0.400000 -2.88458162e+01 9.53374766e+00 3.83791535e+01 8.76679439e+00 3.88547681e-01 2.18549525e-01 1.59856067e-01 8.76679439e+00 0.00000000e+00 0.00000000e+00

0.420000 -2.80632567e+01 1.03157164e+01 3.83791535e+01 9.54340307e+00 3.93481676e-01 2.18974791e-01 1.59856858e-01 9.54340307e+00 0.00000000e+00 0.00000000e+00

0.440000 -2.69729732e+01 1.14066000e+01 3.83791535e+01 1.06288042e+01 3.98533359e-01 2.19404814e-01 1.59857651e-01 1.06288042e+01 0.00000000e+00 0.00000000e+00

0.460000 -2.54131252e+01 1.29664276e+01 3.83791535e+01 1.21830202e+01 4.03706692e-01 2.19842275e-01 1.59858449e-01 1.21830202e+01 0.00000000e+00 0.00000000e+00

0.480000 -2.31412538e+01 1.52382544e+01 3.83791535e+01 1.44490976e+01 4.09005495e-01 2.20292059e-01 1.59859252e-01 1.44490976e+01 0.00000000e+00 0.00000000e+00

0.500000 -1.97869122e+01 1.85925103e+01 3.83791535e+01 1.77974535e+01 4.14433669e-01 2.20763119e-01 1.59860061e-01 1.77974535e+01 0.00000000e+00 0.00000000e+00

0.520000 -1.47833823e+01 2.35959081e+01 3.83791535e+01 2.27947798e+01 4.19995071e-01 2.21272377e-01 1.59860872e-01 2.27947798e+01 0.00000000e+00 0.00000000e+00

0.540000 -7.26071492e+00 3.11182564e+01 3.83791535e+01 3.03108488e+01 4.25693427e-01 2.21852428e-01 1.59861689e-01 3.03108488e+01 0.00000000e+00 0.00000000e+00

0.560000 4.11901940e+00 4.24979537e+01 3.83791535e+01 4.16839925e+01 4.31531963e-01 2.22566797e-01 1.59862506e-01 4.16839925e+01 0.00000000e+00 0.00000000e+00

0.580000 2.14193204e+01 5.97983026e+01 3.83791535e+01 5.89773856e+01 4.37513445e-01 2.23540172e-01 1.59863331e-01 5.89773856e+01 0.00000000e+00 0.00000000e+00

0.600000 4.78241350e+01 8.62030977e+01 3.83791535e+01 8.53745764e+01 4.43639305e-01 2.25017846e-01 1.59864150e-01 8.53745764e+01 0.00000000e+00 0.00000000e+00

0.620000 8.82412383e+01 1.26620154e+02 3.83791535e+01 1.25782897e+02 4.49909264e-01 2.27482870e-01 1.59864980e-01 1.25782897e+02 0.00000000e+00 0.00000000e+00

0.640000 1.50203158e+02 1.88581784e+02 3.83791535e+01 1.87733717e+02 4.56320026e-01 2.31881470e-01 1.59865811e-01 1.87733717e+02 0.00000000e+00 0.00000000e+00

0.660000 2.45153970e+02 2.83530985e+02 3.83791535e+01 2.82668201e+02 4.62863906e-01 2.40052563e-01 1.59866644e-01 2.82668201e+02 0.00000000e+00 0.00000000e+00

0.680000 3.90150192e+02 4.28518707e+02 3.83791535e+01 4.27633791e+02 4.69526971e-01 2.55521697e-01 1.59867475e-01 4.27633791e+02 0.00000000e+00 0.00000000e+00

0.700000 6.09729944e+02 6.48111735e+02 3.83791535e+01 6.47190667e+02 4.76286899e-01 2.84912775e-01 1.59868300e-01 6.47190667e+02 0.00000000e+00 0.00000000e+00

0.720000 9.37455426e+02 9.75855790e+02 3.83791535e+01 9.74872490e+02 4.83112439e-01 3.40318507e-01 1.59869120e-01 9.74872490e+02 0.00000000e+00 0.00000000e+00

0.740000 1.41536463e+03 1.45388946e+03 3.83791535e+01 1.45279663e+03 4.89964148e-01 4.42995359e-01 1.59869926e-01 1.45279663e+03 0.00000000e+00 0.00000000e+00

0.760000 2.09099312e+03 2.12921739e+03 3.83791535e+01 2.12793205e+03 4.96803375e-01 6.28665479e-01 1.59870714e-01 2.12793205e+03 0.00000000e+00 0.00000000e+00

0.780000 3.00693171e+03 3.04556282e+03 3.83791535e+01 3.04394514e+03 5.03592941e-01 9.54218157e-01 1.59871482e-01 3.04394514e+03 0.00000000e+00 0.00000000e+00

0.800000 4.19689848e+03 4.23479465e+03 3.83791535e+01 4.23261808e+03 5.10323623e-01 1.50636870e+00 1.59872224e-01 4.23261808e+03 0.00000000e+00 0.00000000e+00

solar cell parameters deduced from calculated IV-curve:

Voc = 0.553504 Volt

Jsc = 32.95447273 mA/cm2

FF = 65.0755 %

eta = 11.8700 %

V_MPP = 0.437480 Volt

J_MPP = 27.13275282 mA/cm2

SCAPS 3.3.07 ELIS-UGent: Version scaps3307.exe, dated 27-01-2018, 10:11:02

Simulation of I-V curves

Single shot simulation # 6

Calculation started : 16-9-2021 at 14:18:58

problem definition file : c:\Program Files (x86)\Scaps3307\def\example CIGS.def

last saved: 01-09-2015 at 07:44:06

Calculation under illumination

Optical generation from internal SCAPS calculation

Illumination spectrum read from file

spectrum file :

C:\Program Files (x86)\Scaps3307\spectrum\AM1_5G 1 sun.spe last saved: 22-05-2009 at 09:59:48

Neutral density filter: ND 0.0000

Transmission of ND filter: 1.0000e+00

Power from spectrum: 100.0000 mW/cm2

Incident power on solar cell structure 100.0000 mW/cm2

Working point conditions

Temperature 300.00 K

Workpoint bias voltage 0.0000 Volt

Frequency 1.0000e+06 Hz

Voltage reference convention: voltage referred to right contact, voltage V applied to left contact

Current reference convention: current positive when entering the left contact

Power generated by the cell: = -V*I; power consumed by the cell = +V*I

v(V) jtot(mA/cm2) j_total_rec(mA/cm2) j_total_gen(mA/cm2) jbulk(mA/cm2) jifr(mA/cm2) jminor_left(mA/cm2) jminor_right(mA/cm2) j_SRH(mA/cm2) j_Radiative(mA/cm2) j_Auger(mA/cm2)

0.000000 -3.31411625e+01 5.23781670e+00 3.83791535e+01 4.78870840e+00 8.15682193e-02 2.07656365e-01 1.59883713e-01 4.78870840e+00 0.00000000e+00 0.00000000e+00

0.020000 -3.30444472e+01 5.33475090e+00 3.83791535e+01 4.88487754e+00 8.18562163e-02 2.08133427e-01 1.59883718e-01 4.88487754e+00 0.00000000e+00 0.00000000e+00

0.040000 -3.29430892e+01 5.43612031e+00 3.83791535e+01 4.98548219e+00 8.21464314e-02 2.08607972e-01 1.59883720e-01 4.98548219e+00 0.00000000e+00 0.00000000e+00

0.060000 -3.28369559e+01 5.54226590e+00 3.83791535e+01 5.09086316e+00 8.24388927e-02 2.09080126e-01 1.59883722e-01 5.09086316e+00 0.00000000e+00 0.00000000e+00

0.080000 -3.27256693e+01 5.65356589e+00 3.83791535e+01 5.20139854e+00 8.27336262e-02 2.09549998e-01 1.59883724e-01 5.20139854e+00 0.00000000e+00 0.00000000e+00

0.100000 -3.26087985e+01 5.77045120e+00 3.83791535e+01 5.31751912e+00 8.30306586e-02 2.10017701e-01 1.59883726e-01 5.31751912e+00 0.00000000e+00 0.00000000e+00

0.120000 -3.24858470e+01 5.89341842e+00 3.83791535e+01 5.43972132e+00 8.33300149e-02 2.10483354e-01 1.59883725e-01 5.43972132e+00 0.00000000e+00 0.00000000e+00

0.140000 -3.23562432e+01 6.02303943e+00 3.83791535e+01 5.56857690e+00 8.36317262e-02 2.10947084e-01 1.59883727e-01 5.56857690e+00 0.00000000e+00 0.00000000e+00

0.160000 -3.22193273e+01 6.15997409e+00 3.83791535e+01 5.70474552e+00 8.39358186e-02 2.11409022e-01 1.59883729e-01 5.70474552e+00 0.00000000e+00 0.00000000e+00

0.180000 -3.20743257e+01 6.30499613e+00 3.83791535e+01 5.84900078e+00 8.42423202e-02 2.11869308e-01 1.59883731e-01 5.84900078e+00 0.00000000e+00 0.00000000e+00

0.200000 -3.19203057e+01 6.45903873e+00 3.83791535e+01 6.00227565e+00 8.45512599e-02 2.12328089e-01 1.59883733e-01 6.00227565e+00 0.00000000e+00 0.00000000e+00

0.220000 -3.17561053e+01 6.62326373e+00 3.83791535e+01 6.16573181e+00 8.48626667e-02 2.12785525e-01 1.59883734e-01 6.16573181e+00 0.00000000e+00 0.00000000e+00

0.240000 -3.15802214e+01 6.79917475e+00 3.83791535e+01 6.34087266e+00 8.51765703e-02 2.13241783e-01 1.59883736e-01 6.34087266e+00 0.00000000e+00 0.00000000e+00

0.260000 -3.13906022e+01 6.98882374e+00 3.83791535e+01 6.52974995e+00 8.54930008e-02 2.13697047e-01 1.59883738e-01 6.52974995e+00 0.00000000e+00 0.00000000e+00

0.280000 -3.11842720e+01 7.19518615e+00 3.83791535e+01 6.73533890e+00 8.58119887e-02 2.14151518e-01 1.59883740e-01 6.73533890e+00 0.00000000e+00 0.00000000e+00

0.300000 -3.09566917e+01 7.42280105e+00 3.83791535e+01 6.96217833e+00 8.61335650e-02 2.14605419e-01 1.59883742e-01 6.96217833e+00 0.00000000e+00 0.00000000e+00

0.320000 -3.07007102e+01 7.67881882e+00 3.83791535e+01 7.21741831e+00 8.64577611e-02 2.15059007e-01 1.59883744e-01 7.21741831e+00 0.00000000e+00 0.00000000e+00

0.340000 -3.04048945e+01 7.97467119e+00 3.83791535e+01 7.51249025e+00 8.67846086e-02 2.15512588e-01 1.59883746e-01 7.51249025e+00 0.00000000e+00 0.00000000e+00

0.360000 -3.00509733e+01 8.32862775e+00 3.83791535e+01 7.86566332e+00 8.71141393e-02 2.15966547e-01 1.59883748e-01 7.86566332e+00 0.00000000e+00 0.00000000e+00

0.380000 -2.96100983e+01 8.76953451e+00 3.83791535e+01 8.30578299e+00 8.74463850e-02 2.16421392e-01 1.59883750e-01 8.30578299e+00 0.00000000e+00 0.00000000e+00

0.400000 -2.90374564e+01 9.34220150e+00 3.83791535e+01 8.87765852e+00 8.77813769e-02 2.16877850e-01 1.59883752e-01 8.87765852e+00 0.00000000e+00 0.00000000e+00

0.420000 -2.82642942e+01 1.01153786e+01 3.83791535e+01 9.65003867e+00 8.81191454e-02 2.17337023e-01 1.59883755e-01 9.65003867e+00 0.00000000e+00 0.00000000e+00

0.440000 -2.71857081e+01 1.11939648e+01 3.83791535e+01 1.07278206e+01 8.84597194e-02 2.17800710e-01 1.59883757e-01 1.07278206e+01 0.00000000e+00 0.00000000e+00

0.460000 -2.56417815e+01 1.27378695e+01 3.83791535e+01 1.22709106e+01 8.88031245e-02 2.18272007e-01 1.59883758e-01 1.22709106e+01 0.00000000e+00 0.00000000e+00

0.480000 -2.33886410e+01 1.49909563e+01 3.83791535e+01 1.45231666e+01 8.91493817e-02 2.18756507e-01 1.59883759e-01 1.45231666e+01 0.00000000e+00 0.00000000e+00

0.500000 -2.00541645e+01 1.83253329e+01 3.83791535e+01 1.78566859e+01 8.94985041e-02 2.19264650e-01 1.59883761e-01 1.78566859e+01 0.00000000e+00 0.00000000e+00

0.520000 -1.50697493e+01 2.33095852e+01 3.83791535e+01 2.28400346e+01 8.98504924e-02 2.19816393e-01 1.59883761e-01 2.28400346e+01 0.00000000e+00 0.00000000e+00

0.540000 -7.56414417e+00 3.08149551e+01 3.83791535e+01 3.03444155e+01 9.02053277e-02 2.20450503e-01 1.59883765e-01 3.03444155e+01 0.00000000e+00 0.00000000e+00

0.560000 3.80275273e+00 4.21815796e+01 3.83791535e+01 4.17098898e+01 9.05629600e-02 2.21243062e-01 1.59883767e-01 4.17098898e+01 0.00000000e+00 0.00000000e+00

0.580000 2.10999399e+01 5.94786663e+01 3.83791535e+01 5.90055149e+01 9.09232906e-02 2.22344268e-01 1.59883768e-01 5.90055149e+01 0.00000000e+00 0.00000000e+00

0.600000 4.75302036e+01 8.59091706e+01 3.83791535e+01 8.54339495e+01 9.12861433e-02 2.24051161e-01 1.59883773e-01 8.54339495e+01 0.00000000e+00 0.00000000e+00

0.620000 8.80525267e+01 1.26431453e+02 3.83791535e+01 1.25952967e+02 9.16512271e-02 2.26951482e-01 1.59883774e-01 1.25952967e+02 0.00000000e+00 0.00000000e+00

0.640000 1.50317322e+02 1.88695978e+02 3.83791535e+01 1.88211874e+02 9.20180696e-02 2.32202777e-01 1.59883776e-01 1.88211874e+02 0.00000000e+00 0.00000000e+00

0.660000 2.46029528e+02 2.84406623e+02 3.83791535e+01 2.83912284e+02 9.23859308e-02 2.42069124e-01 1.59883778e-01 2.83912284e+02 0.00000000e+00 0.00000000e+00

0.680000 3.92803663e+02 4.31172318e+02 3.83791535e+01 4.30658753e+02 9.27536857e-02 2.60927580e-01 1.59883778e-01 4.30658753e+02 0.00000000e+00 0.00000000e+00

0.700000 6.16327430e+02 6.54709326e+02 3.83791535e+01 6.54159229e+02 9.31196844e-02 2.97094132e-01 1.59883780e-01 6.54159229e+02 0.00000000e+00 0.00000000e+00

0.720000 9.52389696e+02 9.90791701e+02 3.83791535e+01 9.90172355e+02 9.34816723e-02 3.65980633e-01 1.59883782e-01 9.90172355e+02 0.00000000e+00 0.00000000e+00

0.740000 1.44715908e+03 1.48551643e+03 3.83791535e+01 1.48476748e+03 9.38368196e-02 4.95222135e-01 1.59883784e-01 1.48476748e+03 0.00000000e+00 0.00000000e+00

0.760000 2.15368929e+03 2.19186520e+03 3.83791535e+01 2.19087878e+03 9.41819662e-02 7.32352591e-01 1.59883787e-01 2.19087878e+03 0.00000000e+00 0.00000000e+00

0.780000 3.12257072e+03 3.16136395e+03 3.83791535e+01 3.15995430e+03 9.45140449e-02 1.15524700e+00 1.59883787e-01 3.15995430e+03 0.00000000e+00 0.00000000e+00

0.800000 4.39509857e+03 4.43388809e+03 3.83791535e+01 4.43174720e+03 9.48313497e-02 1.88616675e+00 1.59883789e-01 4.43174720e+03 0.00000000e+00 0.00000000e+00

solar cell parameters deduced from calculated IV-curve:

Voc = 0.554021 Volt

Jsc = 33.14116250 mA/cm2

FF = 65.1525 %

eta = 11.9626 %

V_MPP = 0.438301 Volt

J_MPP = 27.29311545 mA/cm2
