# Supplementary material for: Numerical simulation of quantum dots as a buffer layer in CIGS solar cells: a comparative study
Source: Sci Rep. 2022 May 16;12:8099. doi: 10.1038/s41598-022-12234-0 (PMC9110751; doi:10.1038/s41598-022-12234-0)
Supplement: Supplementary file 17 — Supplementary Information 17. [file 41598_2022_12234_MOESM17_ESM.docx]

**Numerical simulation of Quantum Dots as a buffer layer in CIGS Solar Cells: A Comparative study**

Zuhair R. Abdulghani**^a^**, Asmaa Soheil Najm^*^**^b^**, Araa Mebdir Holi ^*^**^c^**, Asla Abdullah Al-Zahrani^*^**^d^**, Khaled S Al-Zahrani^e^, Hazim Moria ^f^

^a^ Department of Mechanical Engineering Technology, Yanbu Industrial College, Yanbu Al-Sinaiyah City, 41912, Saudi Arabia

^b^ Department of Electrical Electronic & Systems Engineering, Faculty of

Engineering and Built Environment, Universiti Kebangsaan Malaysia, 43600

UKM Bangi, Selangor, Malaysia

^c^ Department of Physics, College of Education, University of Al-Qadisiyah, Al-

Diwaniyah, Al-Qadisiyah 58002, Iraq

^d^ Imam Abdulrahman bin Faisal University, Eastern Region, Dammam, Kingdom

of Saudi

^e^ Department of Mechanical Engineering Technology, Yanbu Industrial College, Yanbu Al-Sinaiyah City, 41912, Saudi Arabia

^f^ Department of Mechanical Engineering Technology, Yanbu Industrial College, Yanbu Al-Sinaiyah City, 41912, Saudi Arabia

*Corresponding Authors: [asmaa.soheil@yahoo.com](mailto:asmaa.soheil@yahoo.com); [araa.holi@qu.edu.iq](mailto:araa.holi@qu.edu.iq), [aaalzahrani@iau.edu.sa](mailto:aaalzahrani@iau.edu.sa)

**Supplementary data**


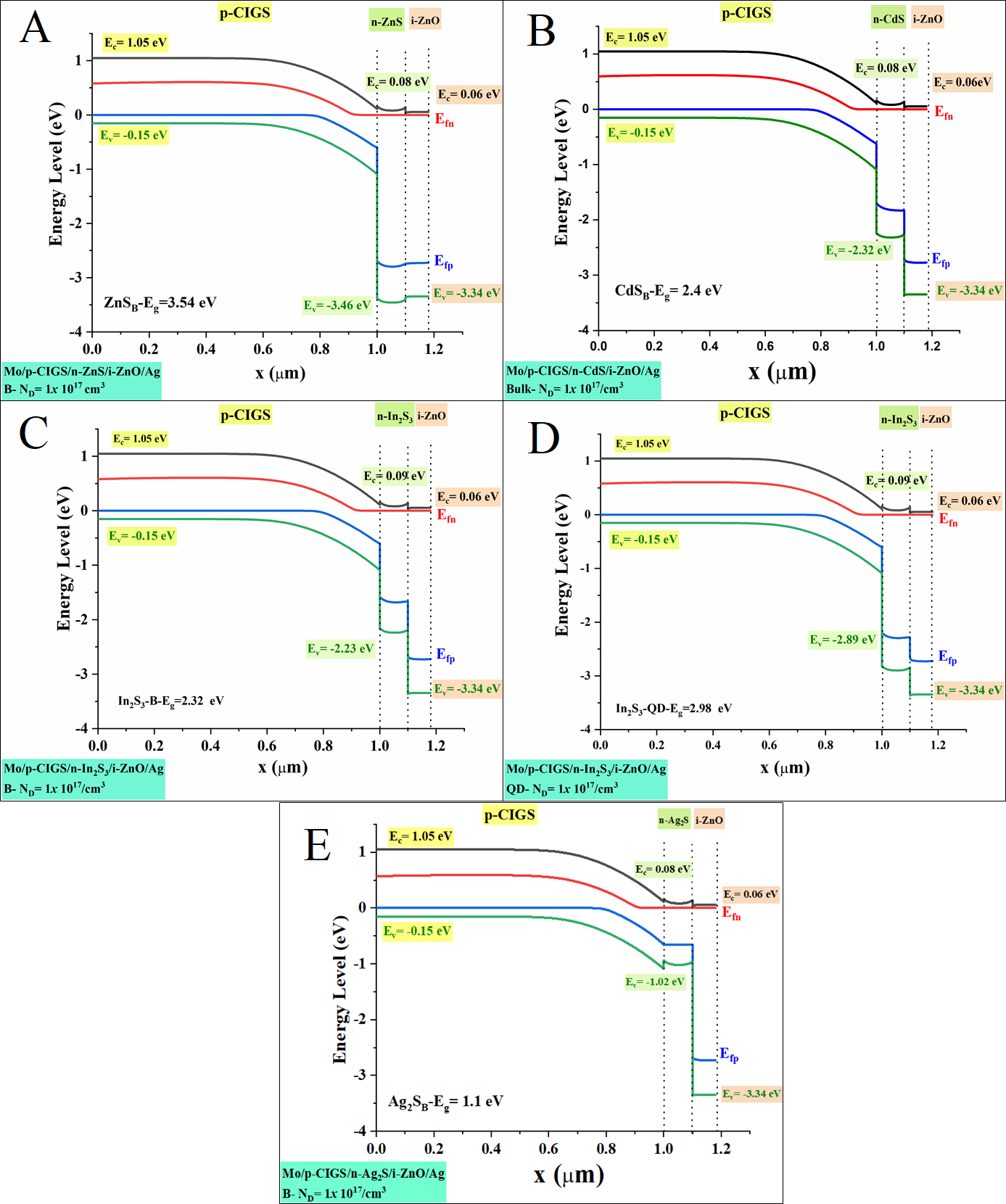


**Fig A1.** Band diagram at Bulk bandgap at N_D_=10^17^


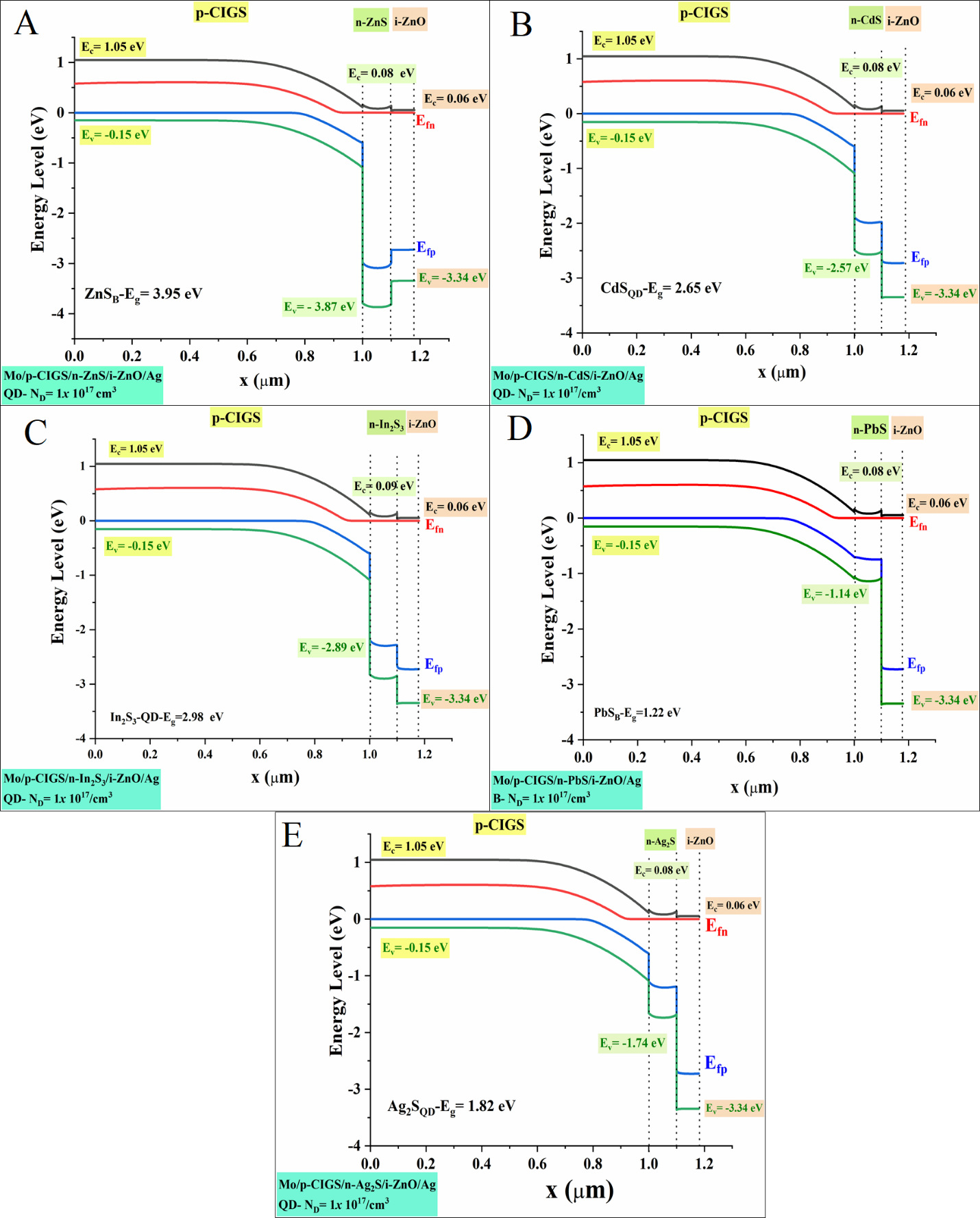


**Fig B1.** Band diagram at Quantum bandgap at N_D_=10^17^


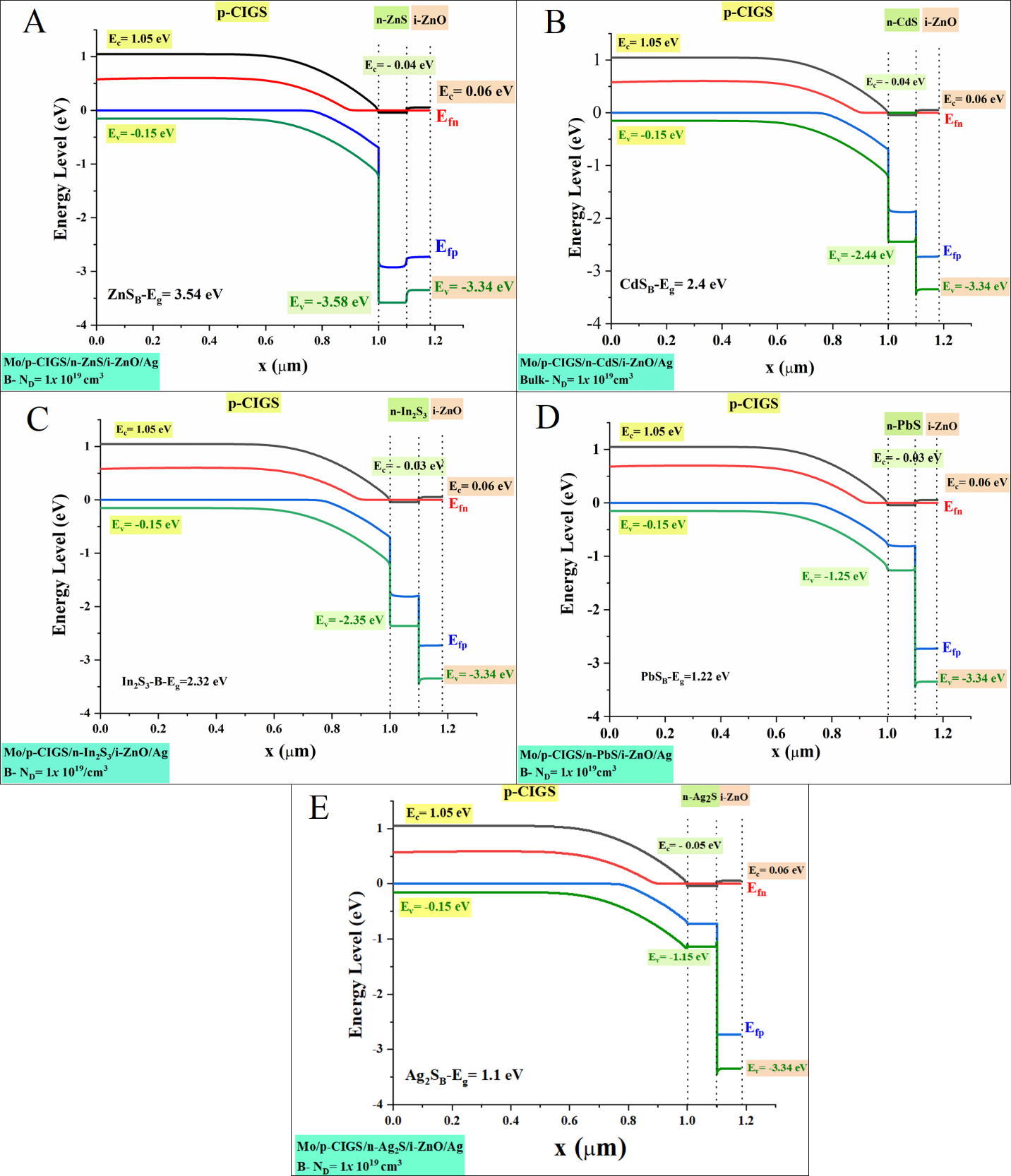


**Fig A2.** Band diagram at Bulk bandgap at N_D_=10^19^


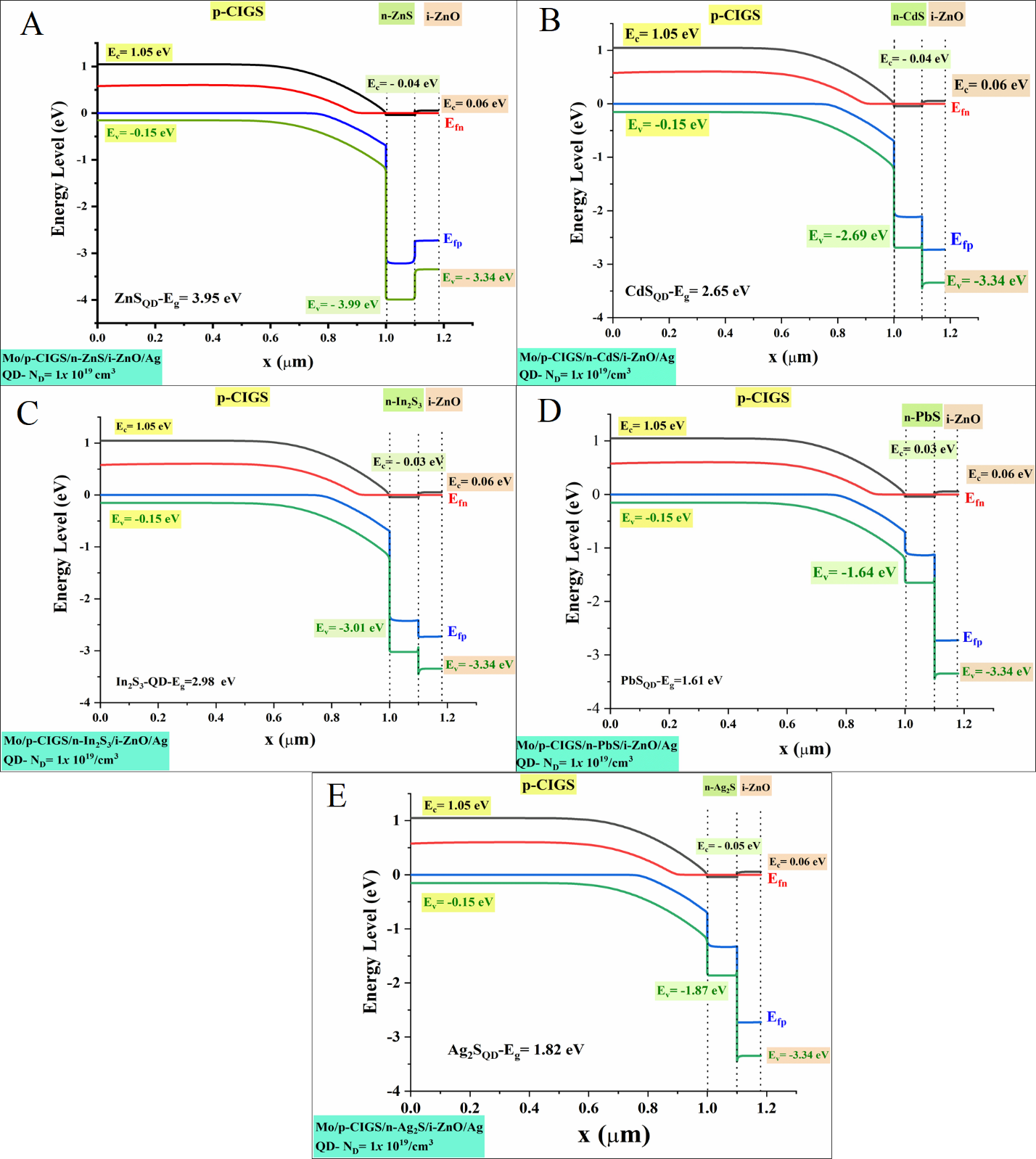


**Fig B2.** Band diagram at Quantum bandgap at N_D_=10^19^
